# Organophosphine-Promoted Decarbynylative Hydrocarbenylation of the Carbon–Carbon Triple Bond

**DOI:** 10.1021/acs.orglett.6c02063

**Published:** 2026-07-03

**Authors:** Mohd Yeshab Ansari, Bang-Zhen Chen, Ting-Jyun Wang, Li-Ching Shen, Shih-Ching Chuang

**Affiliations:** Department of Applied Chemistry, 34914 National Yang Ming Chiao Tung University, Hsinchu 300093, Taiwan

## Abstract

Herein, an innovative illustration of organophosphine-promoted decarbynylative hydrocarbenylation of diynoates with 2-oxindoles has been uncovered, affording (*E*)-3-alkyl/aryl propynylidene/allylidene oxindoles regio- and stereoselectively through the unique C≡C bond scission of diynoates. This tandem reaction proceeded via the initial nucleophilic α-attack of phosphines on diynoates, followed by a sequential process involving proton abstraction/nucleophilic attack, H-shift, and subsequent removal of the phosphorus ylide, enabling the formation of (*E*)-3-alkyl/aryl propynylidene/allylidene oxindoles with broad functional group tolerance. Additionally, the wide substrate scope (>50 entries), scalability, novel reaction mechanism, late-stage functionalization of some FDA-approved marketed drugs, metal-free C≡C bond scission, downstream transformations (>14 examples), and good to excellent yields (up to 92%) with excellent stereo- and regioselectivity are the remarkable features of this strategy.

## Graphical abstract

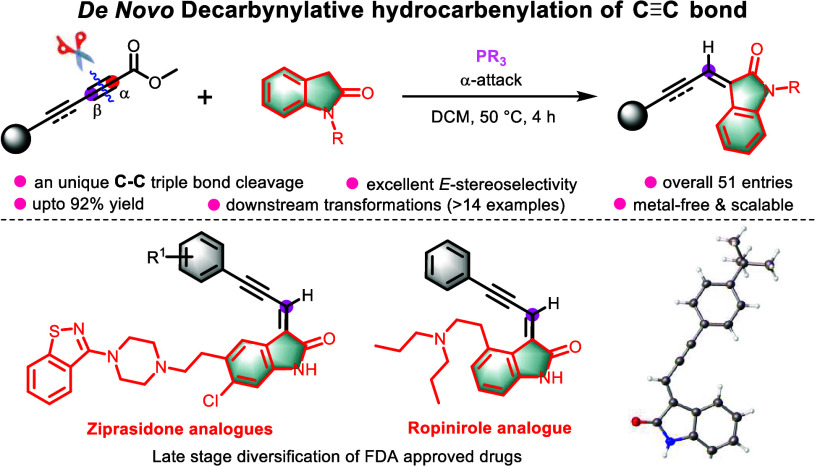

Nitrogen-containing molecules have extensive applications in the chemical and pharmaceutical industries and are widely present in bioactive natural products. Analysis of U.S. FDA-approved small-molecule pharmaceuticals revealed that approximately 60% contain *N*-heterocycles.^1^ One such class of privileged scaffolds, 2-oxindole, constitutes the core of many biologically active compounds, natural products, and commercialized drugs, and is considered one of the most enticing therapeutic components in the realm of drug design and development.^2^ Moreover, 3-alkylidene oxindoles with a specific configuration (*E* or *Z*) are pivotal structural motifs frequently encountered in pharmaceutically active compounds, natural products, and optoelectronic materials due to their remarkable electronic and physical properties.^2,3^ On the other hand, conjugated enynes are important building blocks in contemporary organic synthesis and are extensively employed in various organic reactions, particularly when incorporated into high-value structures.^4^ Due to their substantial therapeutic benefits, the stereoselective construction of these privileged architectures, specifically 3-alkylidene oxindole incorporating the aforementioned building blocks conjugated enynes with structural diversity, is highly prioritized. Over the last few decades, significant advancements have been made in the cleavage of carbon–carbon single and double bonds for molecular transformation.^5^ In contrast, the cleavage of C≡C bonds remains a daunting task, with only a few strategies reported.^6^ This difficulty stems from the exceptionally high bond dissociation energy (>200 kcal/mol) needed to break C≡C bonds.^7^ To accomplish this task, many noble-metal catalysts and stoichiometric amounts of nonrenewable oxidizing agents are frequently employed for alkyne cleavage or C≡C bond activation, which, in turn, limits the appeal and practicality of these strategies.^5a,7b,8^

Besides the well-known alkyne metathesis by using metal–alkylidyne complexes (Scheme 1A),^9^ examples of other metal-catalyzed cleavage of C≡C bonds can still be counted on fingers, and these reactions usually rely on costly and toxic transition metals, including palladium,^7b,8c^ gold,^10^ ruthenium,^11^ platinum, or rhodium,^12^ thereby constraining their practical applicability. Consequently, there remains an opportunity to expand the range of environmentally friendly, cost-effective methodologies for activating the carbon–carbon triple bond, especially developed without using transition metals. In recent decades, two primary synthetic methods have been devised for the synthesis of 3-alkylidene oxindoles and 3-propynylidene oxindoles. The first approach is a metal-free, classical method that installs C3-alkenyl/enyne functionality via a Knoevenagel condensation reaction between 2-oxindoles and carbonyl substrates in a basic medium (Scheme 1B, eq 1).^13^ Another strategy for preparing involves the formation of a five-membered ring using an aniline derivative and an isocyanate as reactants, typically catalyzed by palladium (Scheme 1B, eq 2).,^6^ ^27^ This method often requires multiple steps to synthesize acyclic precursors. However, the above-reported protocols are associated with certain limitations, including a narrow substrate scope, poor stereoselectivity (*E/Z*) at the newly formed olefin, prolonged reaction times, low yields, and the use of an expensive metal catalyst. Moreover, this process shows incompatibility with *N*-substituted 2-oxindoles, making the synthesis of thermal- and alkali-sensitive *N*-substituted 3-alkylideneoxindoles challenging.^14^ On the other hand, nucleophilic phosphine-mediated reactions have emerged as one of the most expeditious synthetic methodologies in contemporary organic synthesis.^15^ Nucleophilic conjugate addition to α,β-unsaturated systems is a fundamental technique, with 1,*n*-addition (*n* = even) patterns like 1,2-, 1,4- or (β), 1,6- or (δ), and 1,8-additions widely studied due to resonance stabilization by the carbonyl functionality. Notably, our research group discovered a rare anti-Michael (α-addition) pathway, in which the α -carbon of α, β-unsaturated species serves as an electrophilic center susceptible to nucleophilic attack by phosphines.^16^ This unique phosphine-catalyzed α-addition, rarely explored in conventional organophosphine chemistry, was demonstrated across a variety of α,β-unsaturated frameworks, including enynoates,^17^ enyndioates,^18^ DMAD,^18b^ oligoynoates,^16a^ diynedioates,^19^ and diynoates.^20^

**Scheme 1:**
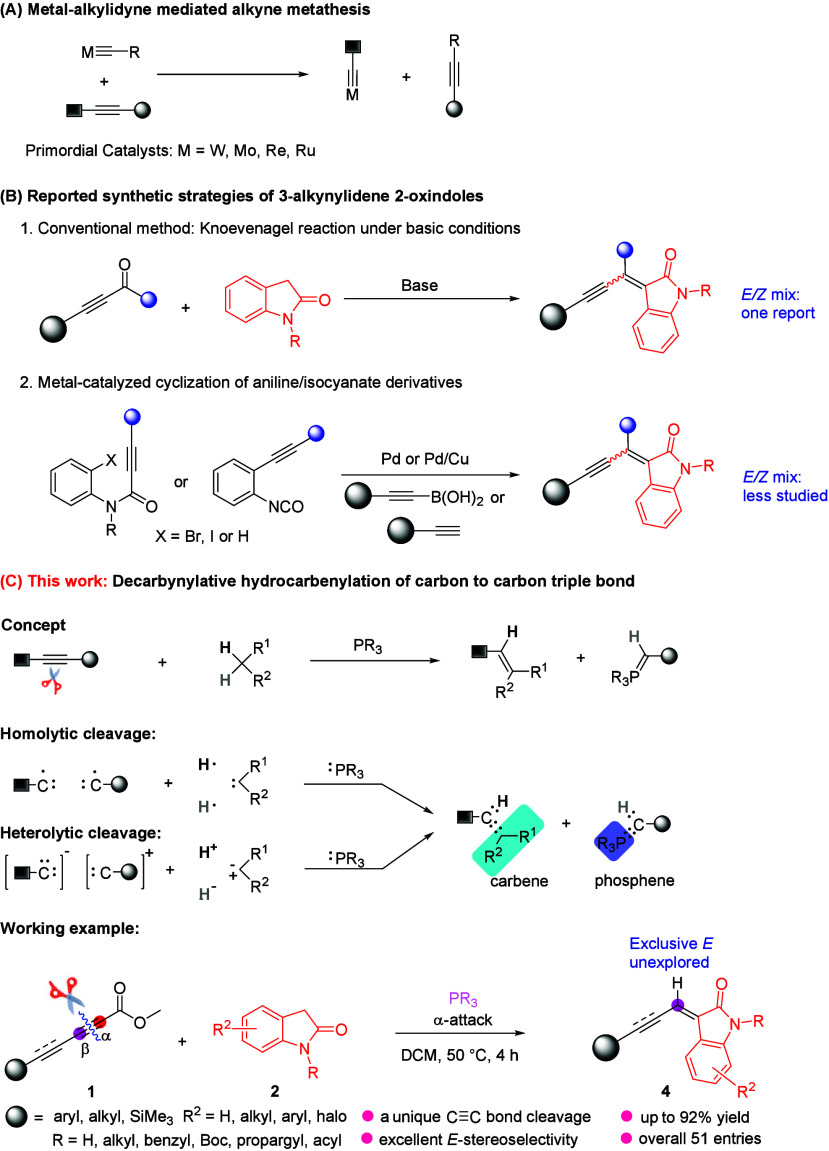
Literature Background and Proposal

Considering the superior pharmaceutical activities, the increasing research importance of 3-alkylidene oxindoles with defined geometry in chemical biology and organic synthesis, and the highly demanded green protocol, we herein disclose a hitherto *de novo* concept for decarbynylative hydrocarbenylation of the C≡C bond without the use of transition metals. This novel concept involves splitting the C≡C bond and the methylene moiety of the reactants homolytically or heterolytically, followed by recombination with phosphines, yielding hydrocarbenylation and hydrophosphenation products, i.e., alkenes and phosphorus ylides, respectively. In real operation, this concept can be achieved by organophosphine-promoted regio- and stereoselective decarbynylative hydrocarbenylation of the C≡C bond of diynoates with 2-oxindole, giving (*E*)-3-alkyl/aryl propynylidene/allylidene oxindoles with exclusive *E* regioselectivity (Scheme 1C).

For optimization studies, we carried out the test reaction using methyl 5-phenylpenta-2,4-diynoate (**1a**), 2-oxindole (**2a**), and triphenylphosphine (**3a**) as model substrates under various conditions, and the outcomes are showcased in Table 1. Initially, the model reaction was conducted at room temperature with a molar ratio of **1a:2a:3a** (1:1:0.5) in different solvents, *viz*., THF, DCM, MeCN, DMF, and *o-DCB.* The best yield of 40% was observed in DCM (Table 1, entry 2). Next, the test reaction was conducted in DCM at room temperature in the absence of PPh_3_, and no product formation was detected (Table 1, entry 6). We then turned our focus to screening the molar ratio of **1a**, **2a**, and PPh_3_ in DCM. The loading of PPh_3_ was evaluated, and 1.2 equiv was found to be the best and provided the target product in 84% yield (Table 1, entry 8), and those with lower or higher than 1.2 equiv of PPh_3_ furnished lower or comparable yields (Table 1, entries 7, 9–10). Subsequently, the reaction temperature and time were tuned under the same molar ratio. Gratifyingly, the best product was generated in 90% yield when the model reaction was tested at 50 °C in DCM for 4 h (Table 1, entry 14).

**Table 1:**
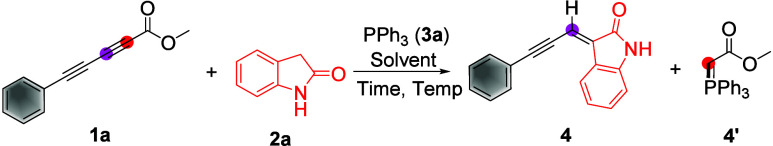
Model Reaction: Optimizations^a^

Entry	Molar ratio **1a**:**2a**:**3a**	Solvent	Time (h)	Temp (°C)	Yield^b^ (%)
1	1:1:0.5	THF	24	rt	24
2	1:1:0.5	DCM	24	rt	40
3	1:1:0.5	ACN	24	rt	37
4	1:1:0.5	DMF	24	rt	23
5	1:1:0.5	*o*-DCB	24	rt	30
6	1:1:0	DCM	24	rt	n.d.^c^
7	1:1:1	DCM	24	rt	79
8	1:1:1.2	DCM	24	rt	84
9	1:1:1.5	DCM	24	rt	82
10	1:1:2	DCM	24	rt	84
11	1:1:1.2	DCM	12	40	84
12	1:1:1.2	DCM	10	50	87
13	1:1:1.2	DCM	6	50	87
14	1:1:1.2	DCM	4	50	90
15	1:1:1.2	DCM	2	50	71
16	1:1:1.2	DCM	4	60	84
17	1:1:1.2	DCM	4	70	81
18	1:1:1.2	DCM	4	80	78
19^d^	1:1:1.2	DCM	4	50	90
20	1:1:1.2	DCE	4	50	84
21	1:1:1.2	EtOH	4	50	76
22	1:1:1.2	1,4-Dioxane	4	50	68
23	1:1:1.2	Toluene	4	50	70

a Reaction conditions: **1a** (0.10 mmol), **2a** (0.10 mmol), and **3a** (0.12 mmol) in anhydrous solvents at specified reaction temperature unless otherwise noted.

b Yields were determined using ^1^H NMR with mesitylene as an internal standard.

c n.d.: not detected.

d Reaction was carried out with 4 Å MS (50 mg).

Encouraged by these optimized results, we applied this novel transformation to a range of diynoates and 2-oxindoles; the outcomes are summarized in Scheme 2. A series of substituted diynoates bearing electron-neutral (H), electron-donating substituents (4-Me, 4-OMe, and 4-^*t*^Bu), and electron-withdrawing substituents (3-F and 4-F) on the phenyl ring were well tolerated and afforded the corresponding products **4** in good to excellent yields. The nature of the groups on the phenyl ring had a slight influence on the transformation’s efficacy. In general, the aromatic ring of diynoates with electron-neutral (H) or electron-donating groups (EDGs) furnished slightly better yields (**4a** and **4c**–**4e**) than those with electron-withdrawing groups (EWGs) (**4b** and **4f**), but were nearly comparable in reactivity. Furthermore, a series of 2-oxindoles with electron-neutral (H), electron-donating (5-Me, 5-Ph, and 5-Cy), and electron-withdrawing substituents (5-F, 7-F, 5-Cl, 6-Cl, and 5-Br) on the aryl ring underwent the reaction smoothly under optimal reaction conditions, delivering the requisite products in good to excellent yields (80–92%), indicating their good reactivities in this decarbynylative hydrocarbenylation. The structure of *E*-olefins **4e** was unequivocally established by X-ray crystallographic investigations. However, the incorporation of a highly electron-withdrawing substituent in 2-oxindole, such as the nitro (-NO_2_) substituent, on the aryl ring did not yield the desired products, likely due to the formation of deactivated intermediate by this nitro moiety. The installation of electronically distinct groups on the phenyl ring of 2-oxindole had an impact, as 2-oxindole substituted with EWGs furnished the target products **4g**-**4n**, **4y**-**4ad**, **4ag**-**4ai**, and **4al** in good yields, while those with EDGs resulted in excellent yields **4u**-**4x** and **4af**. Next, we aimed to evaluate the reactivity of C-5 functionalized 2-oxindole with different aryl hydrocarbons, including phenyl, 2-chlorophenyl, 4-methylphenyl, and 2-naphthyl, all of which were tolerable and yielded the exclusive regioselective products **4o**-**4t** with excellent yields. The reaction proceeded well when the diynoate was equipped with a heterocyclic aryl moiety, such as methyl 5-(thiophen-3-yl)­penta-2,4-diynoate, and the targeted analogs **4ae**-**4ai** were obtained without any deterioration in chemical yields. However, aliphatic diynoates such as methyl undeca-2,4-diynoate and methyl 5-(trimethylsilyl)­penta-2,4-diynoate were also compatible with this transformation and furnished the target molecules **4aj**-**4al**, albeit with moderate yields due to the less stabilized 1,3-dipoles. Notably, a variety of *N*-substituted 2-oxindoles, including *N*-benzyl, *N*-Me, *N*-Et, *N*-acyl, *N*-Boc, and *N*-propargyl, also efficaciously participated in this transformation, affording the corresponding products **5a**-**5g** in good yields. Furthermore, the scope of this decarbynylative hydrocarbenylation was checked using various enynoates under the optimized conditions with P­(cy)_3_. Gratifyingly, all substrates delivered the corresponding products **6a**-**6c** in 30–40% yields with exclusive *E* regiochemistry. The relatively poor yields were ascribed to the presence of dienoate moiety that may further react with phosphines. Furthermore, the substrates bearing a single alkyne unit found to be incompatible under optimal reaction conditions.

**Scheme 2:**
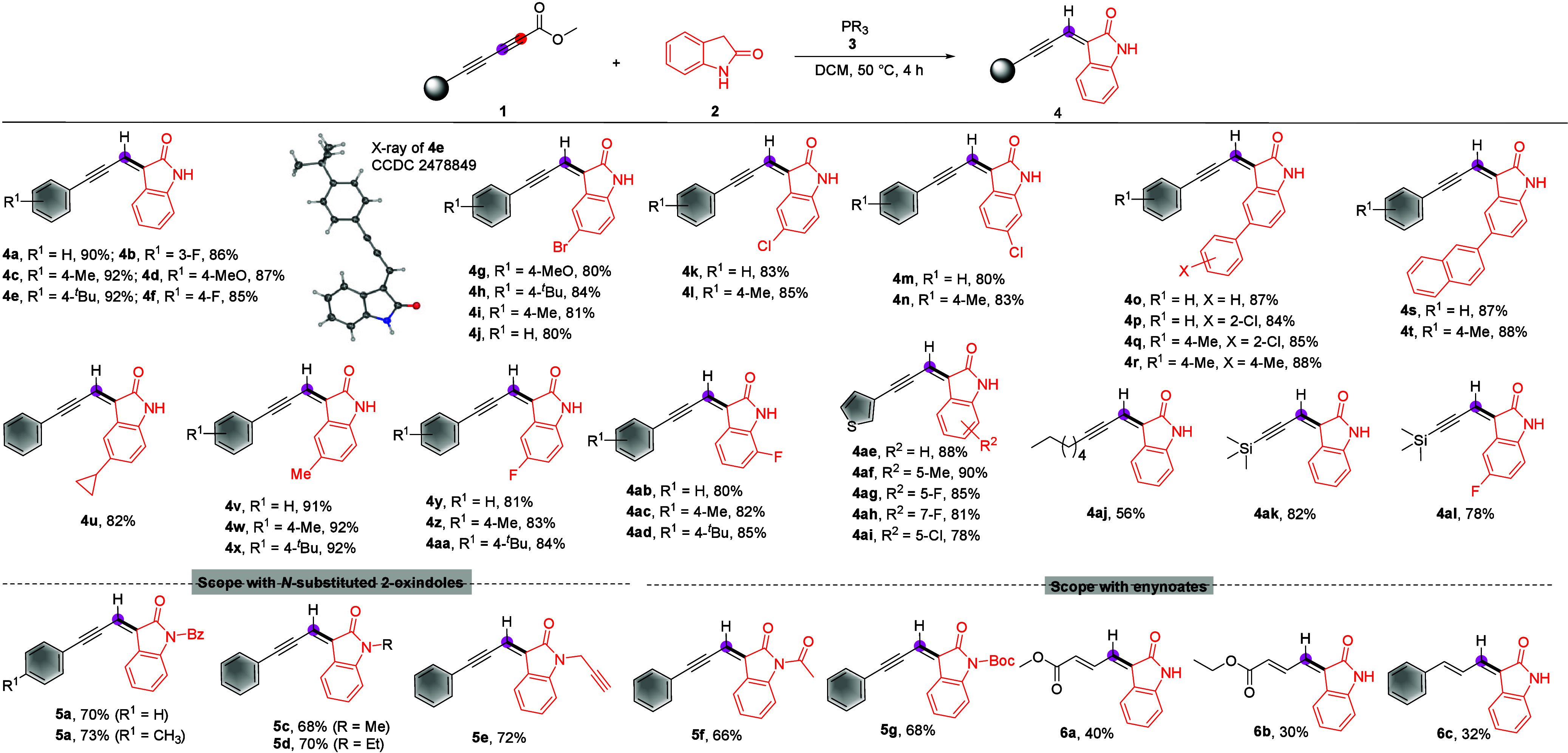
Substrate Scopes with Diynoates/Enynoates and 2-Oxindoles^a^ ^,^ ^b^

a Reaction conditions: **1** (0.10 mmol), **2** (0.10 mmol), and **3** (0.12 mmol) in dry DCM (3.0 mL) at 50 °C for 4 h.
b Yields were determined using ^1^H NMR with mesitylene as an internal standard.

Moreover, we aimed to utilize this highly robust strategy, decarbynylative hydrocarbenylation, for late-stage functionalization of FDA-approved drugs with intrinsic 2-oxindole pharmacophores. As shown in Scheme 3A, Ziprasidone (a psychotropic agent, FDA approval: 2001) and Ropinirole (an anti-Parkinson agent, FDA approval: 1997) were both well tolerated under the reaction conditions and converted into the target analogs **7a**-**7c** in good yields with excellent regioselectivity. To showcase the robustness and synthetic application, a metal-free regioselective reaction of diynoates **1a** (5.4 mmol) with 2-oxindole **2a** (5.4 mmol) and PPh_3_ **3a** (6.5 mmol) was conducted on a large scale. The reaction proceeded efficiently, yielding **4a** in 88% under the optimized conditions, with no significant loss in the target product yield (Scheme 3B, a). This demonstrates its considerable potential for future industrial applications. Next, the postmodification of compound **4a** was explored. First, the reduction of product **4a** using LiAlH_4_ in THF was carried out at room temperature. It selectively reduced the conjugated olefin, furnishing product **8** in 58% yield, while the carbonyl group of the amide remained unaffected. After that, allylation of product **4a** was conducted with allyl bromide under basic conditions, yielding the desired compound **9** in 75% yield. Then, the unsaturation, *viz*., alkyne and olefin functionalities in product **4a**, were efficiently reduced upon hydrogenation to furnish **10** in 68% yield. In addition to these synthetic modifications, we have also carried out several renowned name reactions, including the Curtius reaction, Scholl reaction, Ullmann coupling, Chan-Lam coupling of compound **4a**, and Suzuki cross-coupling of compound **4j** with suitable reaction partners, as shown in Scheme 3B. All could afford the corresponding products in good yields. Interestingly, the skeleton of compound **12**, *viz*., benzo­[*cd*]­indol-2­(1*H*)-one, exhibits promising biological activity as BET bromodomain inhibitors with therapeutic potential in various cancers and inflammatory diseases.^21^ Therefore, the approaches outlined in Scheme 3B not only provide a simple and efficient route to obtaining both medicinally and synthetically significant compounds but also broaden the synthetic versatility of this strategy.

**Scheme 3:**
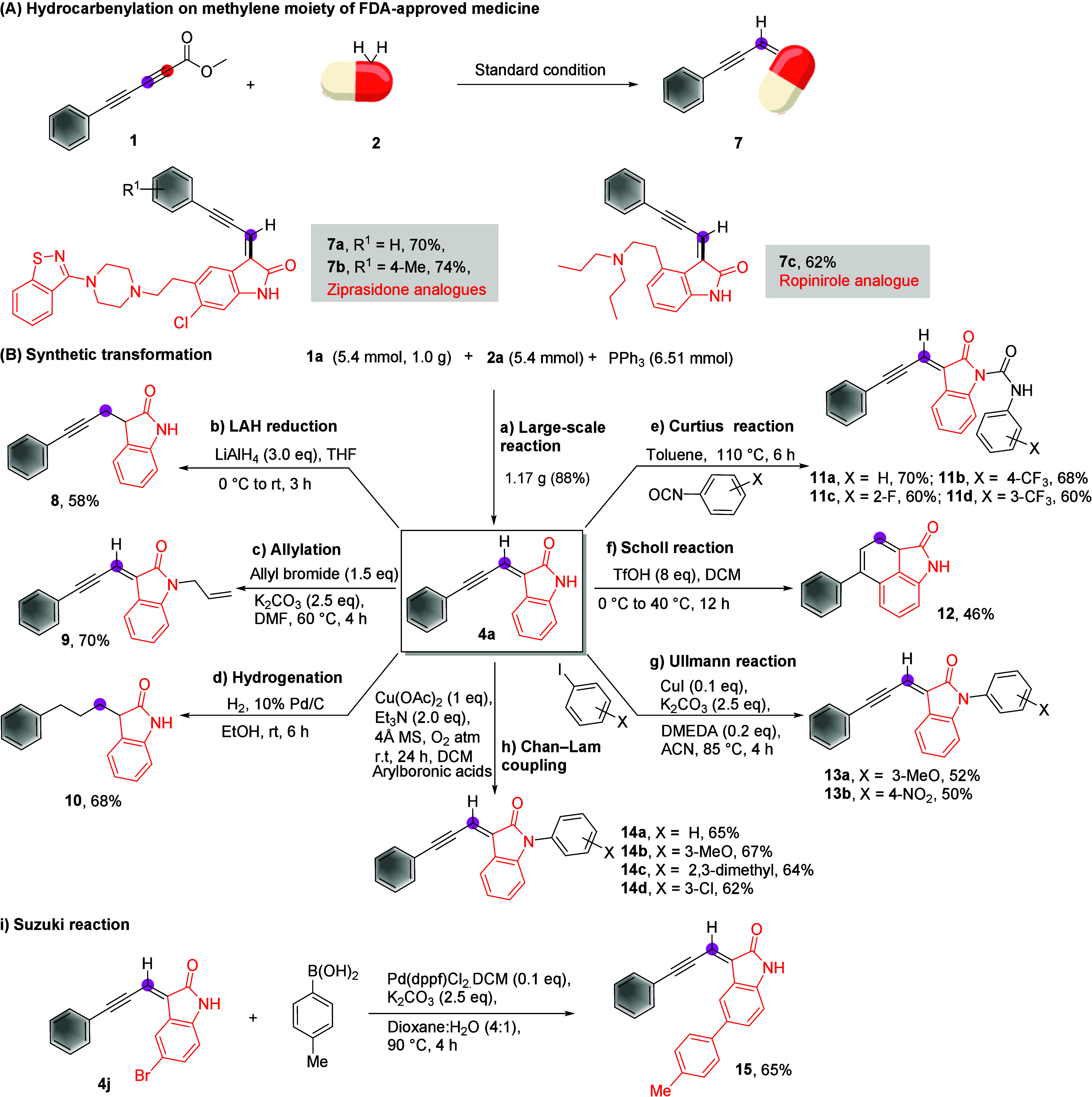
(A) Late-Stage Functionalization: Application Foreground of the Presented Strategy in Drug Discovery; (B) (a) Large-Scale Reaction; (b–i) Follow-Up Transformations

Next, to investigate the mechanistic pathway, control experiments were conducted, and the results are shown in Scheme 4A. When the reaction was performed without triphenylphosphine under standard conditions, no product formation was observed, indicating that triphenylphosphine is crucial for the conversion (Scheme 4A, eq a). Furthermore, identifying byproduct species is key to understanding the reaction mechanism. In this study, a reactive byproduct was clearly visible distinctly on TLC upon completion of the reaction and was further confirmed by ^1^H and ^31^P NMR of the crude reaction mixture, which showed two ^31^P signals at 22.56 and 18.59 ppm, clearly delineating the isomeric forms of the phosphorus ylide byproduct (**4′**). Thus, attempts were made to isolate this byproduct; however, it proved highly unstable, readily transforming into triphenylphosphine oxide during column chromatography. Subsequently, we tried to trap this *in situ* generated byproduct methyl (triphenylphosphoranylidene) acetate (**4′**) by reacting it with 4-nitrobenzaldehyde (**2′**) through Wittig olefination without altering the reaction parameters, which resulted in the formation of the desired product species **4a″**, *viz*., olefin, in 65% yield, which was confirmed by NMR spectroscopy (Scheme 4A, eq b). To further support the existence of phosphorus ylide byproduct **4′**, Wittig olefination of the possible phosphorus ylide byproduct, methyl (triphenylphosphoranylidene)­acetate (**4′**) with 4-nitrobenzaldehyde (**2′**) under standard conditions provided the desired olefin **4a″** in 70% yield, indicating that phosphorus ylide **4′** is a potential byproduct (Scheme 4A, eq c). An isotope labeling experiment with D_3_-**2a** (>99% D) gave 36% deuterium incorporation in the olefinic C–H of **4a** (Scheme 4A, eq d, see the SI for ^1^H and D NMR in Figures S148–S149).

**Scheme 4:**
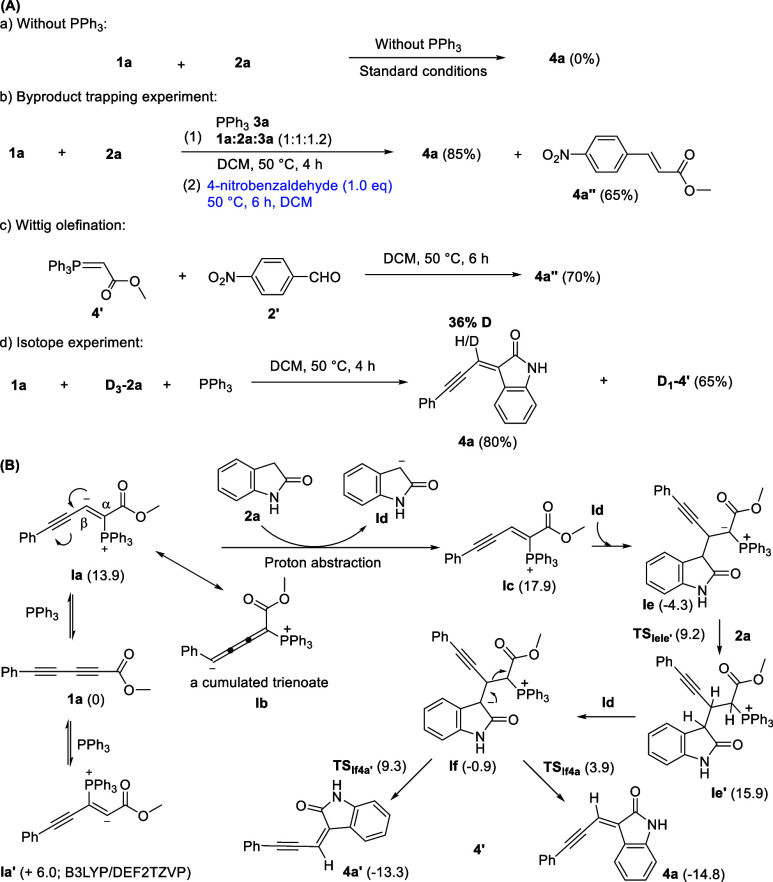
(A) Control Experiments and (B) Proposed Mechanism

Based on the mechanistic studies and literature precedents,^16b^ a plausible mechanistic network for this three-component tandem reaction is depicted in Scheme 4B. The reaction begins with the nucleophilic conjugate addition of phosphine to the α-carbon of diynoate **1a**, furnishing the expected reactive zwitterionic intermediate, **Ia**, which possesses a carbanion at the β-carbon. The alternative 1,3-dipolar species, **Ia′**, resulting from β-attack, possess higher energy by 6.0 kcal/mol, as determined using the B3LYP/DEF2TZVP theory.^20^ Then, the adduct intermediate **Ia**, upon proton abstraction from 2-oxindole, converted to phosphonium cation intermediate **Ic**, leaving behind 2-oxindole carbanion **Id**. The intermediate **Ic**, underwent the nucleophilic attack by anion **Id** at β-position, followed by a proton abstraction from **2a** again to furnish **Ie′**. The intermediate **Ie′** was deprotonated by **Id** to give **If**. Finally, intermediate **If** upon C–C bond cleavage through electron shifting delivered the desired product **4a**, along with the release of phosphorus ylide byproduct **4′**. The exclusive *E* stereochemistry of **4a** is attributed to the bulky triarylphosphine moiety, which sits on the same side as the amide in **If**, with a Coulombic interaction between the oxygen and phosphonium groups. Thereby, the overall decarbynylative hydrocarbenylation of **1a** with **2a** is performed. Two key points were summarized from DFT calculation with B3LYP-D3 6–31G­(d,p) theory using solvation model SMD (DCM): (1) a direct 1,3-proton shift from **Ie** to **If** is excluded due to its high transition state energy of 46.9 kcal/mol (2) the stereoselective formation of **4a** over **4a′** is corroborated with the Gibbs free energy of activation **TS**_**If4a**_ and **TS**_**If4a′**_: 4.8 versus 10.2 kcal/mol (See Figure S150 for energy profile). Lastly, we did not observe any products from *N*-addition, even though the *N*-H proton is more acidic than the C3–H proton of 2-oxindole.

We have uncovered an organophosphine-promoted decarbynylative hydrocarbenylation of diynoates with 2-oxindoles, affording (*E*)-3-alkyl/aryl propynylidene/allylidene oxindoles in an excellent regio- and stereoselective fashion through unique C≡C bond scission of diynoates. The tandem reaction proceeded via nucleophilic α addition pattern. The advantages of this decarbynylative hydrocarbenylation strategy included a wide substrate scope (>50 entries), the scalability, mild reaction conditions, metal-free C≡C bond scission, operational simplicity, easily accessible feedstocks, novel reaction mechanism, good to excellent yields (up to 92%) with exclusive regio- and stereoselectivity, and excellent functional group compatibility. Moreover, the late-stage functionalization of some FDA-approved marketed drugs and a series of intriguing downstream transformations (>14 examples) were also successfully executed to further showcase the potential of this approach. Further application of this nucleophilic addition reaction pattern for synthesizing valuable scaffolds, alongside ongoing studies on (*E*)-3-alkyl/aryl propynylidene/allylidene oxindoles in anticancer screening and OLED production, will be disclosed in due course. We anticipate that the robustness of this protocol will encourage medicinal chemists to adopt it in the near future, as it may be applicable to bioconjugation studies.

## Supplementary Material



## Data Availability

The data underlying this study are available in the published article and its Supporting Information.

## Notes

Experimental procedures and compound characterization data, including the ^1^H/^13^C NMR spectra (PDF)
