# Supplementary material for: Organophosphine-Promoted Decarbynylative Hydrocarbenylation of the Carbon–Carbon Triple Bond
Source: Org Lett. 2026 Jul 3;28(28):8793–9. doi: 10.1021/acs.orglett.6c02063 (PMC13386537; doi:10.1021/acs.orglett.6c02063)
Supplement: Supplementary file 1 [file ol6c02063_si_001.pdf]

Supporting Information

Organophosphine-Promoted Decarbynylative Hydrocarbenylation of the Carbon-Carbon Triple Bond

Mohd Yeshab Ansari, Bang-Zhen Chen, Ting-Jyun Wang, Li-Ching Shen, and Shih-Ching Chuang*

Department of Applied Chemistry, National Yang Ming Chiao Tung University, Hsinchu 300093, Taiwan

*Corresponding author: Email: jscchuang@nycu.edu.tw

Contents

General remarks 2

General experimental procedure for the synthesis of 4, 5, 6, and 7 3

Unsuccessful substrates 4

Mechanistic investigations 5

^{31}P NMR monitoring to observe byproduct evolution 7

Large-scale experiment 7

Synthetic transformations 8

Characterization data of all the synthesized compounds 11

^{1}H and ^{13}C NMR Spectra of Compounds 30

Optimized coordinates of computed species 107

Figure S150. Energy profile of the reaction of 1a, 2a, and PPh_3 (B3LYP-D3/6-31g(d,p)) 150

References 150

General remarks

All reagents and solvents were purchased from Sigma-Aldrich or Merck Chemical Co. and used directly without further purification. TLC (Thin Layer Chromatography) was performed on Merck-precoated silica gel, and 100-200 mesh silica gel was used for column chromatography. The chromatographic solvents are listed as volume-to-volume (v/v) ratios. All the synthesized compounds were fully characterized by ^{1}H and ^{13}C NMR, IR, and further confirmed through ESI-MS and HRMS analyses. NMR spectra were recorded using 400 MHz spectrometers for ^{1}H NMR, 100 MHz for ^{13}C NMR, and 162 MHz for ^{31}P NMR, respectively. Chemical shifts are reported in δ (ppm) relative to TMS (^{1}H), $CDCl_3$, and DMSO-d_6 (^{13}C) as internal standards. The chemical shifts of ^{31}P NMR were taken with reference to 85% of H_3PO_4 in D_2O, and those of ^{1}H and ^{13}C with reference to TMS or $CHCl_3$. Multiplicities are reported as follows: singlet (s), doublet (d), doublet of doublets (dd), doublet of triplets (dt), triplet (t), triplet of doublets (td), quartet (q), multiplet (m), broad singlet (bs). Integrals are in accordance with assignments, and coupling constants are given in Hz. High-resolution mass spectra (HRMS) were recorded on a JEOL JMS-T200GC AccuTOF GCx time-of-flight (TOF) mass spectrometer using field desorption (FD) ionization. Melting points were determined using a melting point apparatus; IR spectra were recorded on a Bruker spectrometer. Solvents were distilled prior to use. All reactions were performed under argon. Anhydrous benzene and THF were distilled from sodium/benzophenone under argon. Anhydrous 1,2-dichlorobenzene (*o*-DCB) and dichloromethane (DCM) were distilled from CaH_2 under argon. The diynoates **1** and oxindoles **2** were prepared according to reported procedures.[1-17] Computational studies were performed using Gaussian 16. All optimized structures and frequency calculations were performed using B3LYP-D3/6-31 G(d,p) with the SMD (DCM) solvation model, unless otherwise specified.

Table S1. Screening of phosphine catalysts for the reaction. [a-b]

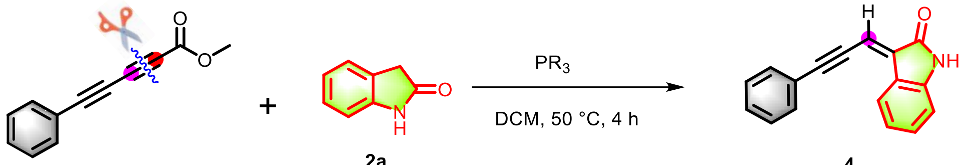

Entry	Molar ratio **1a:2a:3**	Solvent	Catalyst (PR_3)	Yield[b] (%)
**1**	**1:1:1.2**	**DCM**	**PPh_3**	**90**
2	1:1:1.2	DCM	PPh_2(*p*-tolyl)	89
3	1:1:1.2	DCM	P(*p*-tolyl)$_3$	88
4	1:1:1.2	DCM	P(*m*-tolyl)$_3$	86
5	1:1:1.2	DCM	P(4-OMe-Ph)$_3$	78
6	1:1:1.2	DCM	P(4-Cl-Ph)$_3$	71
7	1:1:1.2	DCM	P(4-F-Ph)$_3$	76
8	1:1:1.2	DCM	Tri(2-thienyl)phosphine (PTh3)	55
9	1:1:1.2	DCM	Tri(1-naphthyl)phosphine P(1-nap)$_3$	23
10	1:1:1.2	DCM	Tricyclohexylphosphine P(Cy)$_3$	75

[a]Reaction condition: **1a** (0.10 mmol), **2a** (0.10 mmol), and PR_3 (0.12 mmol) in anhydrous DCM at 50 °C for 4 h.
[b]Yields were determined using ^{1}H NMR with mesitylene as an internal standard.

General experimental procedure for the synthesis of 4, 5, 6, and 7

To a 3.0 mL of anhydrous DCM solution containing phosphine **3a** (31.6 mg, 0.12 mmol, 1.2 equiv) and oxindole **2** (13.3 mg, 0.1 mmol, 1.0 equiv) at room temperature (rt), **1** (18.5 mg, 0.1 mmol in 1.0 mL of DCM, 1.0 equiv) was added *via* a syringe in 10 min. Upon completion of the injection, the mixture was transferred to 50 ºC using an oil bath and stirred for another 4 h. After completion of the reaction, monitored by TLC, the mixture was concentrated under reduced pressure and purified by flash column chromatography with hexanes/EA as eluents to give the target products **4**, **5**, and **7**. For compounds **6** (Pcy)$_3$ was used in place of PPh_3. The isolated compounds were well characterized by IR, ^{1}H NMR, ^{13}C NMR, HRMS, and X-ray crystallographic study.

Unsuccessful substrates

Scheme S1: Screening of various class of nucleophiles with diynoates

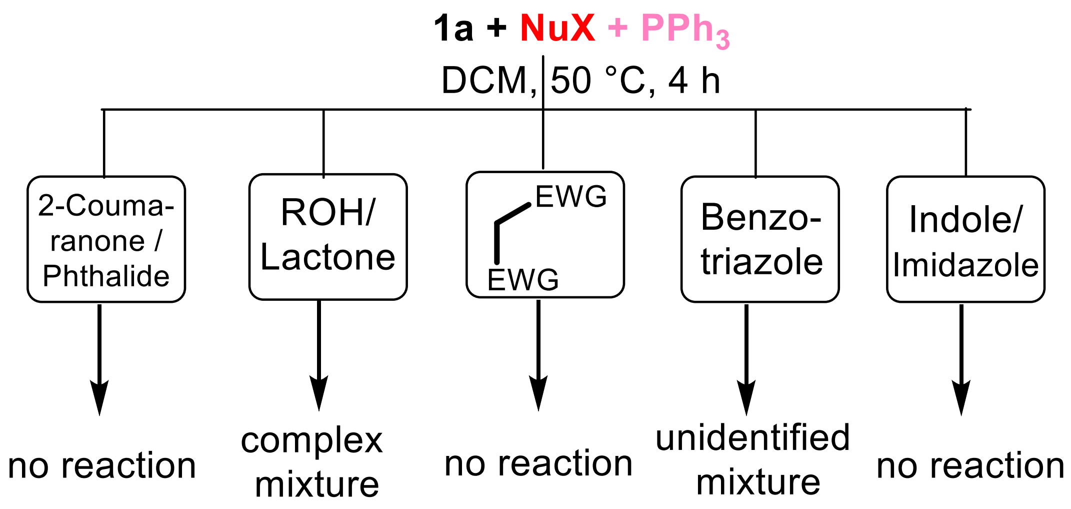

Screening of Various Nucleophiles: We carried out a series of reactions using diynoate **1a** (0.1 mmol, 1.0 eq) with different nucleophiles **2** (1.0 eq) such as 2-coumaranone, phthalide, ROH (ethanol, phenol, 2-naphthol and triflouroethanol), lactones (δ-valerolactone, ε-caprolactone), active methylene compounds ((phenylsulfonyl)acetonitrile, ethyl acetoacetate (EAA), malonic ester, ethyl cyanoacetate, malononitrile, dimedone), benzotriazole, indole, imidazole, benzimidazole, *N*-Methyl-2-pyrrolidone (NMP), quinoxalin-2(1*H*)-one and PPh_3 **3a** (1.2 eq) under optimized reaction conditions (Table 1, entry 14) in dry DCM at 50 °C, unlike oxindole target products were not formed in any cases.

Mechanistic investigations

a) Reaction without PPh_3

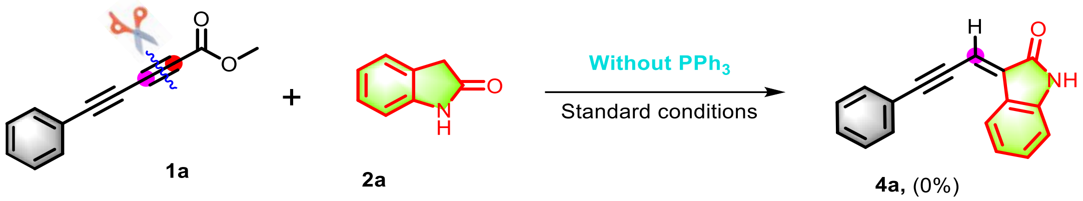

To investigate the mechanistic pathway, some control experiments were conducted. First, the reaction was performed without triphenylphosphine under standard conditions as follows. To a 3.0 mL solution of anhydrous DCM containing oxindole **2a** (0.1 mmol) was added **1a** (0.1 mmol in 1.0 mL of DCM) *via* a syringe over 10 min. Upon completion of the injection, the mixture was transferred to 50 °C and stirred for an additional 4 hours. No progress/product formation in the reaction was observed, indicating that triphenylphosphine is crucial for this conversion.

b) Byproduct trapping experiment

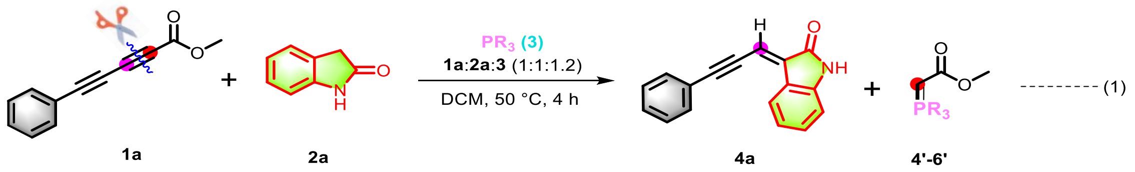

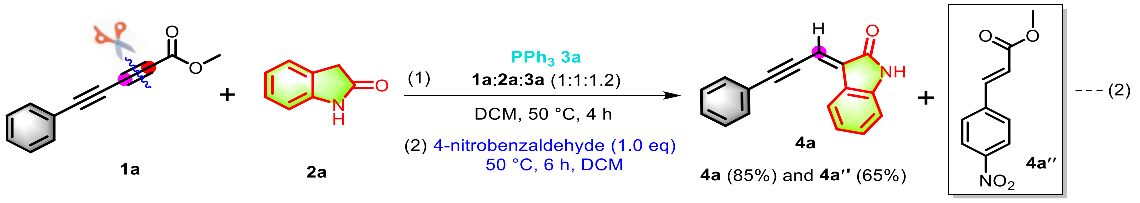

The identification of byproduct species plays a key role in understanding the reaction mechanism. In this study, a reactive byproduct was clearly visible distinctly on TLC upon completion of the reaction and further was confirmed by NMR of the crude reaction mixture. Thus, attempts were made to isolate this byproduct; however, it was found to be highly unstable, readily transforming into triphenylphosphine oxide upon column chromatography. Subsequently, we carried out the model reaction using different phosphine catalysts (3) and the resulting corresponding byproduct methyl (triphenylphosphoranylidene)acetates (**4'-6'**) were confirmed by ^{1}H and ^{31}P NMR of crude reaction mixture (Scheme b, 1). Furthermore,

we conducted an byproduct trapping experiment using Wittig olefination, maintaining the reaction parameters as follows. To a 3.0 mL of anhydrous DCM solution containing phosphine **3a** (0.12 mmol) and 2-oxindole **2a** (0.1 mmol) at rt was added **1a** (0.1 mmol in 1.0 mL of DCM) *via* a syringe in 10 min. Upon completion of the injection, the mixture was heated to 50 ºC and stirred for 4 hours. After that, 4-nitrobenzaldehyde (1.0 equiv) in DCM (1 mL) was added, and the mixture was stirred at the same temperature for an additional 6 hours. After completion of the reaction monitored by TLC, the mixture was concentrated under reduced pressure and purified by flash column chromatography with hexanes/EA as eluents to give the target olefin, methyl (*E*)-3-(4-nitrophenyl)acrylate **4a''** in 65% yield and **4a** in 85% yield, confirmed by NMR spectroscopy (Scheme b, 2).

c) Wittig olefination

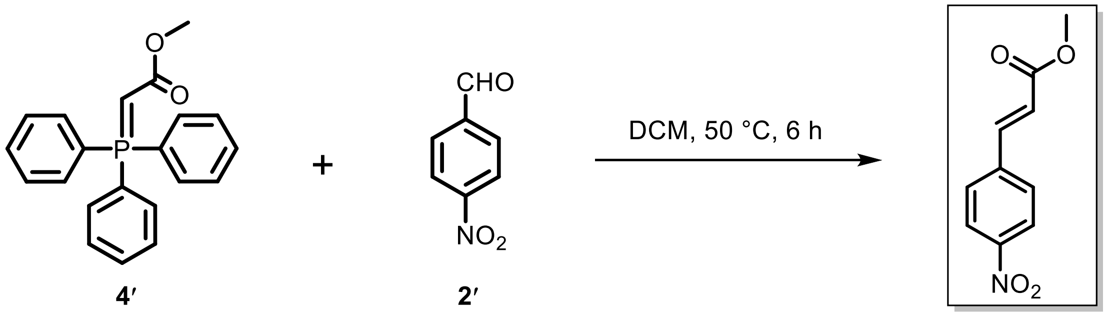

4a″ (70%)

To further support the existence of phosphorous ylide byproduct **4'**, Wittig olefination of the possible phosphorus ylide byproduct methyl (triphenylphosphoranylidene)acetate (**4'**) was carried out under standard conditions as follows. To a 3.0 mL of anhydrous DCM solution containing methyl (triphenylphosphoranylidene)acetate **4'** (0.1 mmol) was added 4-nitrobenzaldehyde **2'** (0.12 mmol). The reaction mixture was stirred at 50°C for 6 hours. After completion of the reaction monitored by TLC, the mixture was concentrated under reduced pressure and purified by flash column chromatography with hexanes/EA as eluents to give the target olefin, methyl (*E*)-3-(4-nitrophenyl)acrylate **4a''** in 70% yield as a white solid, indicating that phosphorus ylide **4'** is a potential byproduct.[18]

d) Crossover experiments

Furthermore, to probe the possibility of intermolecular scrambling, a crossover experiment was performed using diynoate **1a** (0.1 mmol), phosphine **3a** (0.12 mmol) in the presence of 5-methyl oxindole (0.033 mmol), 5-fluoro oxindole (0.033 mmol), and oxindole (0.033

mmol), under the standard reaction conditions. Crude ^{1}H NMR analysis of the reaction mixture displayed three distinct olefinic resonances corresponding to the expected products derived from the three oxindole substrates. No additional olefinic signals attributable to crossover or scrambled products were observed. The corresponding products were obtained in 32%, 31%, and 15% yields, respectively. These results suggest that intermolecular scrambling is unlikely and support the proposed reaction pathway.

^{31}P NMR monitoring to observe byproduct evolution

To gain further insight into the reaction mechanism and monitor the evolution of phosphorus-containing species, the model reaction was monitored by time-dependent ^{31}P NMR spectroscopy. Aliquots were collected at 10 min intervals and analyzed by ^{31}P NMR. At the initial stage of the reaction, signals corresponding to triphenylphosphine ($\delta_P \approx 4.7$ ppm) and triphenylphosphine oxide ($\delta_P \approx 29.5$ ppm) were observed, together with two additional resonances at $\delta_P \approx 22.5$ and 18.5 ppm, which were assigned to isomeric forms of the phosphorus ylide byproduct (**4'**). As the reaction progressed, the intensities of the resonances at $\delta_P \approx 22.5$ and 18.5 ppm increased steadily, indicating the gradual formation and accumulation of these byproducts during the course of the reaction.

The observation of these time-dependent changes in the ^{31}P NMR spectra provides direct evidence for the generation of phosphorus-containing byproducts and is consistent with the proposed mechanistic pathway involving phosphorus ylide byproduct (**4'**).

Large-scale experiment

To highlight the synthetic utility, a large-scale, metal-free, regioselective cross-coupling alkenynlation/olefination of oxindole was carried out under optimal reaction conditions. To a 20 mL solution of anhydrous DCM containing phosphine **3a** (6.51 mmol) and 2-oxindole **2a** (5.4 mmol) at room temperature (rt) was added **1a** (5.4 mmol in 10 mL of DCM) *via* a syringe in 10 minutes. Upon completion of the injection, the mixture was heated at 50 ºC and stirred for another 4 hours. After completion of the reaction monitored by TLC, the mixture was concentrated under reduced pressure and purified by flash column chromatography with hexanes/EA as eluents to give the target product **4a** in 88% yield (1.17 g), pleasingly without the significant decrease in the product yield. This has proven to have great potential for future applications from an industrial point of view.

Synthetic transformations

Example 1: Synthesis of 3-(3-phenylprop-2-yn-1-yl)indolin-2-one (8)

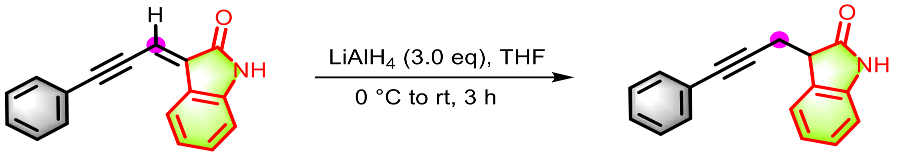

4a **8**

To a solution of **4a** (24.5 mg, 0.1 mmol, 1 eq) in 3.0 mL anhydrous THF was added dropwise 0.3 mL of 1M $LiAlH_4$ (3.0 eq) at 0 °C under nitrogen. The reaction mixture was allowed to warm to room temperature (rt) and stir for 3 hours. Upon completion of the reaction, monitored by TLC, the mixture was cooled to 0 °C and treated sequentially with 0.1 mL of H_2O, 0.1 mL of 10% NaOH, and 0.3 mL of H_2O in a dropwise manner. The mixture was stirred for 10 min, filtered, and the filter cake was washed with THF. The filtrate was dried (Na_2SO_4) and concentrated *in vacuo* to give a yellow solid material. The material was purified by flash column chromatography with hexanes/EA as eluents to give the target products **8** (58%, 14.3 mg) as a yellow solid.

Example 2: Synthesis of (*E*)-1-allyl-3-(3-phenylprop-2-yn-1-ylidene)indolin-2-one (9)

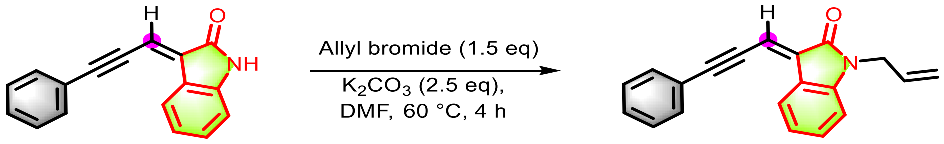

4a **9**

To a solution of the **4a** (24.5 mg, 0.1 mmol, 1 eq) in dry DMF (3 mL) was added K_2CO_3 (2.5 eq), and the mixture was stirred under N_2 at room temperature (rt) for 20 min. To this mixture, allyl bromide (1.5 equiv) was added, and the reaction was stirred at 60 °C for 4 hours. After completion, the reaction mixture was cooled to room temperature and diluted with EtOAc (20 mL). The mixture was washed with brine (20 mL) and then with cold H_2O (20 mL). The organic layer was dried (Na_2SO_4) and concentrated *in vacuo*. The resulting material was purified by flash column chromatography with hexanes/EA as eluents to give the target products **9** (70%, 20.0 mg) as yellow solid.

Example 3: Synthesis of 3-(3-phenylpropyl)indolin-2-one (10)

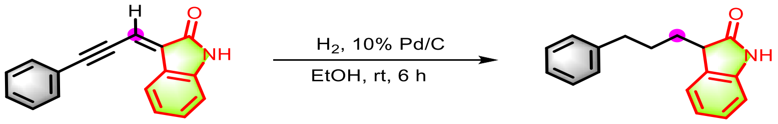

4a **10**

To a round-bottom flask containing **4a** (24.5 mg, 0.1 mmol, 1.0 equiv) in EtOH (3 mL) was added 10% Pd/C (10.6 mg, 0.01 mmol) under a hydrogen atmosphere (balloon), and the reaction mixture was stirred at room temperature (rt) for 6 hours. After completion of the reaction as monitored by TLC, the reaction mixture was filtered on Celite and the filtrate was concentrated and purified by flash column chromatography with hexanes/EA as eluents to give the target products **10** (68%, 17.1 mg) as white solids.

Example 4: General experimental procedure for the synthesis of (*E*)-2-oxo-*N*-phenyl-3-(3-phenylprop-2-yn-1-ylidene)indoline-1-carboxamides (11a-11d) or *N*-(phenylaminocarbonyl)-2-oxyindoles (11a-11d)

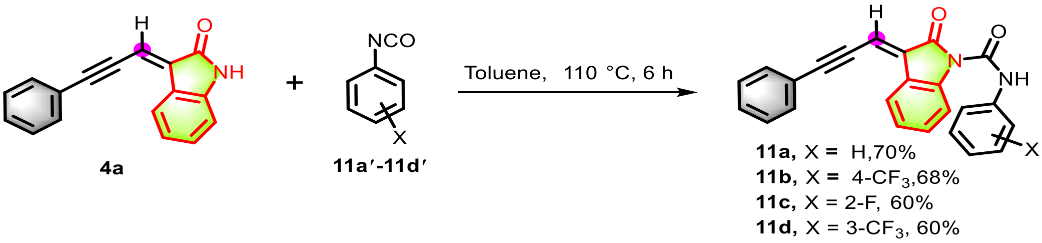

To a solution of **4a** (24.5 mg, 0.1 mmol) in dry toluene (3 mL) was added phenyl isocyanate **11a'-11d'** (1.2 eq) at room temperature. After the resultant mixture had been stirred at 110 °C for 6 hours, the solvent of the mixture was evaporated under reduced pressure. The resultant product was purified by flash column chromatography with hexanes/EA as eluents to give the target products **11a-11d**.

Example 5: Synthesis of 5-phenylbenzo[*cd*]indol-2(1*H*)-one (12)

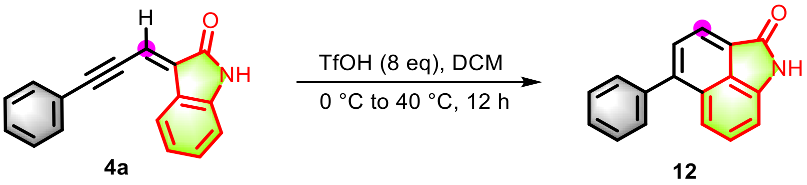

In a flame-dried round-bottom flask, compound **4a** (24.5 mg, 0.1 mmol) was dissolved in dry DCM (3 mL) under a nitrogen atmosphere and cooled to 0 °C. A DCM solution of trifluoromethanesulfonic acid (8 eq, 0.01 M in DCM) was added over 10 min using a syringe pump at 0 °C. The resultant reaction mixture was stirred at 40 °C for 12 hours. After completion of the reaction as indicated by TLC, the reaction mixture was cooled to room temperature (rt) and diluted with DCM (15 mL) and then quenched with a saturated aqueous solution of $NaHCO_3$. The DCM layer is washed with water, dried over Na_2SO_4, and concentrated under reduced pressure. The residue obtained was purified by silica column

chromatography using 80% hexanes/EA as eluents to afford compound 12 (52%, 12.7 mg) as a yellow solid.

Example 6: Synthesis of (*E*)-1-phenyl-3-(3-phenylprop-2-yn-1-ylidene)indolin-2-ones (13a-13b)

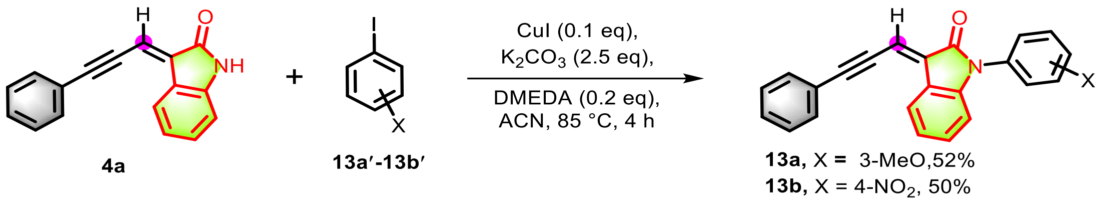

An oven-dried vial was charged with compound **4a** (24.5 mg, 0.1 mmol, 1 eq), iodoarenes **13a'-13b'** (1.2 equiv), and acetonitrile (3 mL) under a nitrogen atmosphere. The mixture was purged with N_2 for 15 min at 40 °C. Thereafter, potassium carbonate (2.5 equiv), copper (I) iodide (10 mol %), and *N,N'*-dimethylethylenediamine (20 mol %) were added and sealed under nitrogen blanket. The reaction mixture was then stirred for 4 hours at 85 °C. The reaction mixture was then cooled to room temperature, and 1M HCl (15 mL/mmol) was added. The reaction mixture was extracted with ethyl acetate (EtOAc) (3 × 15 mL). The combined organic layer was washed with brine (2 × 10 mL), dried over sodium sulfate (Na_2SO_4), and concentrated under reduced pressure. The residue obtained was purified by flash column chromatography using 20% ethyl acetate in hexane as eluents to afford compounds **13a-13b**.

Example 7: Synthesis of (*E*)-1-phenyl-3-(3-phenylprop-2-yn-1-ylidene)indolin-2-ones (14a-14d)

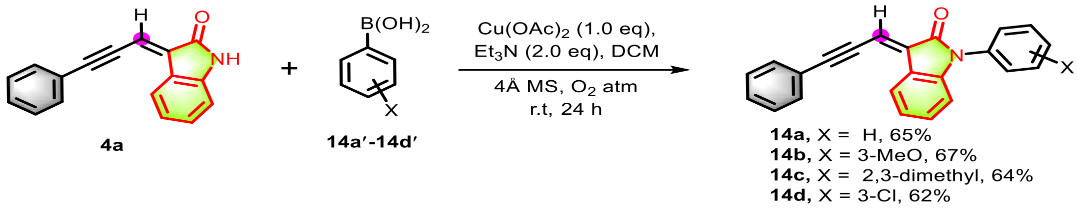

To a solution of **4a** (24.5 mg, 0.1 mmol, 1 eq) in dry dichloromethane (3.0 mL) were added $PhB(OH)_2$ **14a'-14d'** (0.15 mmol, 1.5 eq), 4Å MS (50 mg), Et_3N (28.1 µL, 0.2 mmol, 2 eq), and $Cu(OAc)_2$ (18.1 mg, 0.1 mmol, 1 eq) under an oxygen atmosphere at room temperature. After stirring at the same temperature for 24 hours, to the reaction mixture was added silica gel (4 g). The resultant mixture was filtered through a pad of celite and the filter cake was washed with EtOAc. The filtrate was concentrated under reduced pressure. The residue was purified by flash column chromatography using 20% ethyl acetate in hexane as eluents to afford compounds **14a-14d** as yellow solid.

Example 8: Synthesis of (*E*)-3-(3-phenylprop-2-yn-1-ylidene)-5-(*p*-tolyl)indolin-2-one (15)

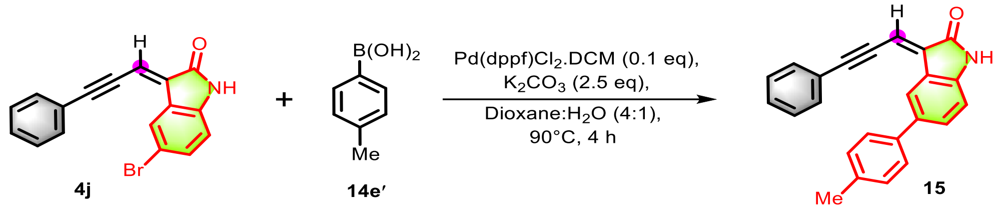

To a solution of compound **4j** (32.3 mg, 0.1 mmol) and *p*-tolylboronic acid (16.2 mg, 1.2 eq) in dioxane (4 mL), a solution of K_2CO_3 (2.5 eq) in H_2O (1 mL) was added. The mixture was purged with N_2 for 10 minutes, after which Pd(dppf)Cl_2 was added (0.1 eq), and the mixture was stirred at 90 °C for 4 hours. After completion of the reaction, as indicated by TLC, the mixture was then poured into ice-cold H_2O (20 mL) and extracted with EtOAc. The combined organics were washed with H_2O, dried over Na_2SO_4, concentrated in vacuum, and the resulting residue was purified by flash column chromatography using 20% ethyl acetate in hexane as eluents to afford compound **15** (65%, 21. 8 mg) as a yellow solids.

Characterization data of all the synthesized compounds

(*E*)-3-(3-phenylprop-2-yn-1-ylidene)indolin-2-one (4a)

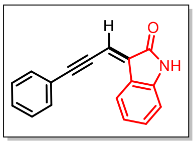

Yellow solid; yield 90% (22.1 mg); m.p. 194-196 °C; R_f = 0.38 (hexanes/EA = 7:3); **^{1}H NMR** (400 MHz, $CDCl_3$) δ 8.14 (d, J = 7.5 Hz, 1H), 7.90 (brs, 1H), 7.63-7.59 (m, 2H), 7.43 (dd, J = 5.1, 2.0 Hz, 3H), 7.29 (dd, J = 7.7, 1.3 Hz, 1H), 7.08 (td, J = 7.6, 1.0 Hz, 1H), 6.97 (s, 1H), 6.88 (d, J = 7.8 Hz, 1H); **^{13}C NMR** (100 MHz, $CDCl_3$) δ 168.8, 141.2, 134.8, 132.1, 130.6, 129.8, 128.8, 124.1, 122.6, 122.5, 122.4, 113.9, 110.0, 105.6, 87.7; **HRMS** (FD^+) Calcd for $C_{17}H_{11}NO$ $[M]^+$ 245.0835, Found 245.0831; **FT-IR** (KBr) $\tilde{\nu}$ (cm^{-1}) 2189, 1707, 1607, 1592.

(*E*)-3-(3-(3-fluorophenyl)prop-2-yn-1-ylidene)indolin-2-one(4b)

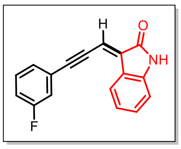

Brown solid; yield 86% (22.6 mg); m.p. 208-210 °C; R_f = 0.42 (hexanes/EA = 7:3); **^{1}H NMR** (400 MHz, $CDCl_3$) δ 8.08 (d, J = 7.6 Hz, 1H), 7.68 (br s, 1H), 7.42-7.38 (m, 2H), 7.30-7.27 (m, 2H), 7.17-7.11 (m, 1H), 7.09 (td, J = 7.6, 1.0 Hz, 1H), 6.93 (s, 1H), 6.88-6.86 (m, 1H); **^{13}C NMR** (100 MHz, $CDCl_3$) δ 168.5, 162.5 (d, $^1J_{FC}$ = 246.2 Hz), 141.3, 135.4, 130.9, 130.4 (d, $^3J_{FC}$ = 8.7 Hz), 128.0 (d, $^4J_{FC}$ = 2.9 Hz), 124.2 (d, $^3J_{FC}$ = 7.9 Hz), 122.5 (d, $^3J_{FC}$ = 12.9 Hz), 118.8 (d, $^2J_{FC}$ = 23.0 Hz), 117.2 (d, $^2J_{FC}$ = 20.9 Hz), 113.2, 110.0, 88.2; **HRMS** (FD^+) Calcd for $C_{17}H_{10}FNO$ $[M]^+$ 263.0741, Found 263.0742; **FT-IR** (KBr) $\tilde{\nu}$ (cm^{-1}) 2191, 1706, 1607, 1579.

(*E*)-3-(3-(*p*-tolyl)prop-2-yn-1-ylidene)indolin-2-one (4c)

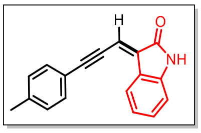

Yellow solid; yield 92% (23.8 mg); m.p. 198-200 °C; R_f = 0.38 (hexanes/EA = 7:3); **^{1}H NMR** (400 MHz, DMSO-d_6) δ 10.61 (br s, 1H), 8.01 (d, J = 7.5 Hz, 1H), 7.56 (d, J = 7.8 Hz, 2H), 7.32-7.28 (m, 3H), 7.07 (t, J = 7.6 Hz, 1H), 6.88 (d, J = 7.7 Hz, 1H), 6.81 (s, 1H), 2.37 (s, 3H); **^{13}C NMR** (100 MHz, DMSO-d_6) δ 167.6, 142.8, 140.4, 135.2, 131.9, 131.0, 129.8, 123.2, 121.8, 121.6, 118.4, 111.8, 110.1, 104.7, 87.2, 21.2; **HRMS** (FD$^+$) Calcd for $C_{18}H_{13}NO$ [M]$^+$ 259.0991, Found 259.0985; **FT-IR** (KBr) $\tilde{\nu}$ (cm^{-1}) 2177, 1708, 1614, 1596.

(*E*)-3-(3-(4-methoxyphenyl)prop-2-yn-1-ylidene)indolin-2-one (4d)

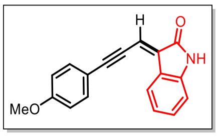

Light yellow solid; yield 87% (23.9 mg); m.p. 205-207 °C; R_f = 0.36 (hexanes/EA = 7:3); **^{1}H NMR** (400 MHz, DMSO-d_6) δ 10.59 (br s, 1H), 8.02 (d, J = 7.5 Hz, 1H), 7.62 (d, J = 8.7 Hz, 2H), 7.30 (t, J = 7.7 Hz, 1H), 7.09-7.05 (m, 3H), 6.88 (d, J = 7.7 Hz, 1H), 6.80 (s, 1H), 3.83 (s, 3H); **^{13}C NMR** (100 MHz, DMSO-d_6) δ 167.7, 160.8, 142.7, 134.5, 133.8, 130.8, 123.2, 121.8, 121.7, 114.9, 113.3, 112.2, 110.1, 105.2, 87.1, 55.5; **HRMS** (FD$^+$) Calcd for $C_{18}H_{13}NO_2$ [M]$^+$ 275.0941, Found 275.0933; **FT-IR** (KBr) $\tilde{\nu}$ (cm^{-1}) 2183, 1711, 1614, 1594.

(*E*)-3-(3-(4-(*tert*-butyl)phenyl)prop-2-yn-1-ylidene)indolin-2-one (4e)

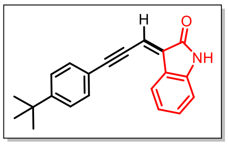

Yellow solid; yield 92% (27.7 mg); m.p. 204-206 °C; R_f = 0.42 (hexanes/EA = 7:3); **^{1}H NMR** (400 MHz, $CDCl_3$) δ 8.59 (br s, 1H), 8.11 (d, J = 7.5 Hz, 1H), 7.52 (d, J = 8.4 Hz, 2H), 7.42 (d, J = 8.4 Hz, 2H), 7.25-7.22 (m, 1H), 7.06-7.01 (m, 1H), 6.94 (s, 1H), 6.88 (d, J = 7.8 Hz, 1H), 1.32 (s, 9H); **^{13}C NMR** (100 MHz, $CDCl_3$) δ 169.3, 153.4, 141.4, 134.6, 132.0, 130.5, 125.8, 124.0, 122.7, 122.4, 119.4, 114.2, 110.1, 106.2, 87.4, 35.1, 31.2; **HRMS** (FD$^+$) Calcd for $C_{21}H_{19}NO$ [M]$^+$ 301.1461, Found 301.1454; **FT-IR** (KBr) $\tilde{\nu}$ (cm^{-1}) 2185, 1740, 1613, 1599.

(*E*)-3-(3-(4-fluorophenyl)prop-2-yn-1-ylidene)indolin-2-one (4f)

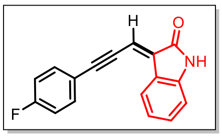

Brown solid; yield 85% (22.3 mg); m.p. 220-222 °C; R_f = 0.36 (hexanes/EA = 7:3); **^{1}H NMR** (400 MHz, DMSO-d_6) δ 10.62 (br s, 1H), 8.00 (d, J = 7.6 Hz, 1H), 7.79-7.70 (m, 2H), 7.38-7.29 (m, 3H), 7.07 (t, J = 7.6 Hz, 1H), 6.89 (d, J = 7.8 Hz, 1H), 6.80 (s, 1H); **^{13}C NMR** (100 MHz, DMSO-d_6) δ 167.6, 162.9 (d, $^1J_{FC}$ = 248.8 Hz), 142.9, 135.6, 134.5 (d, $^3J_{FC}$ = 8.8 Hz), 131.1, 123.4, 121.9, 121.5, 117.9, 116.5 (d, $^2J_{FC}$ = 22.2 Hz), 111.4, 110.1, 103.1, 87.3; **HRMS (FD$^+$):** Calcd for $C_{17}H_{10}FNO$ [M]$^+$ 263.0740, Found 263.0732; **FT-IR** (KBr) $\tilde{\nu}$ (cm^{-1}) 2186, 1708, 1615, 1594.

(*E*)-5-bromo-3-(3-(4-methoxyphenyl)prop-2-yn-1-ylidene)indolin-2-one (4g)

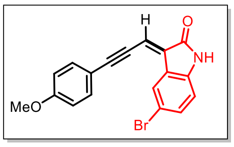

Yellow solid; yield 80% (28.2 mg); m.p. 227-229 °C; R_f = 0.40 (hexanes/EA = 7:3); **^{1}H NMR** (400 MHz, $CDCl_3$) δ 8.35 (d, *J* = 2.0 Hz, 1H), 7.59 (d, *J* = 8.8 Hz, 2H), 7.49 (br s, 1H), 7.38 (dd, *J* = 8.3, 2.0 Hz, 1H), 6.99-6.95 (m, 3H), 6.75 (d, *J* = 8.3 Hz, 1H), 3.87 (s, 3H); **^{13}C NMR** (100 MHz, $CDCl_3$) δ 167.5, 141.8, 133.9, 133.5, 133.1, 124.0, 115.3, 114.4, 113.3, 112.3, 106.6, 79.0, 55.7; **HRMS** (FD$^+$) Calcd for $C_{18}H_{12}BrNO_2$ $[M]^+$ 353.0046, Found 353.0048; **FT-IR** (KBr) $\tilde{\nu}$ (cm^{-1}) 2177, 1710, 1622, 1593.

(*E*)-5-bromo-3-(3-(4-(*tert*-butyl)phenyl)prop-2-yn-1-ylidene)indolin-2-one (4h)

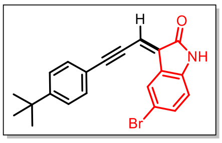

Light yellow solid; yield 84% (31.8 mg); m.p. 212-214 °C; R_f = 0.36 (hexanes/EA = 7:3); **^{1}H NMR** (400 MHz, $CDCl_3$) δ 8.36 (s, 1H), 7.90 (br s, 1H), 7.58 (d, *J* = 8.4 Hz, 2H), 7.49-7.45 (m, 2H), 7.39 (dd, *J* = 8.2, 2.1 Hz, 1H), 7.00 (s, 1H), 6.77 (d, *J* = 8.2 Hz, 1H), 1.35 (s, 9H); **^{13}C NMR** (100 MHz, $CDCl_3$) δ 168.4, 154.0, 139.9, 133.2, 132.7, 132.2, 127.0, 126.1, 124.6, 119.1, 116.0, 115.0, 111.3, 107.8, 87.4, 35.2, 31.2; **HRMS** (FD$^+$) Calcd for $C_{21}H_{18}BrNO$ $[M]^+$ 379.0566, Found 379.0574; **FT-IR** (KBr) $\tilde{\nu}$ (cm^{-1}) 2180, 1713, 1619, 1595.

(*E*)-5-bromo-3-(3-(*p*-tolyl)prop-2-yn-1-ylidene)indolin-2-one (4i)

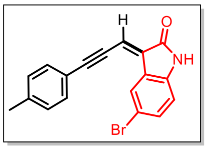

Yellow solid; yield 81% (27.3 mg); m.p. 228-230 °C; R_f = 0.34 (hexanes/EA = 7:3); **^{1}H NMR** (400 MHz, $CDCl_3$) δ 10.75 (br s, 1H), 8.14 (d, *J* = 2.0 Hz, 1H), 7.53 (d, *J* = 7.9 Hz, 2H), 7.48 (dd, *J* = 8.3, 2.0 Hz, 1H), 7.35 (d, *J* = 7.9 Hz, 2H), 6.91-6.83 (m, 2H), 2.38 (s, 3H); **^{13}C NMR** (100 MHz, $CDCl_3$) δ 167.0, 141.8, 140.8, 134.1, 133.1, 131.6, 129.9, 125.5, 123.7, 118.2, 113.7, 113.0, 112.0, 105.6, 87.2, 21.2; **HRMS** (FD$^+$) Calcd for $C_{18}H_{12}BrNO$ $[M]^+$ 337.0097, Found 337.0099; **FT-IR** (KBr) $\tilde{\nu}$ (cm^{-1}) 2176, 1719, 1608, 1593.

(*E*)-5-bromo-3-(3-phenylprop-2-yn-1-ylidene)indolin-2-one (4j)

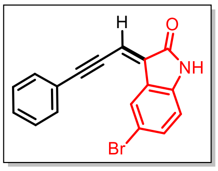

Yellow solid; yield 80% (26.6 mg); m.p. 220-222 °C; R_f = 0.40 (hexanes/EA = 7:3); **^{1}H NMR** (400 MHz, $CDCl_3$) δ 10.77 (br s, 1H), 8.15 (s, 1H), 7.65 (dd, *J* = 5.8, 2.1 Hz, 2H), 7.55-7.53 (m, 3H), 7.49 (dd, *J* = 8.3, 2.0 Hz, 1H), 6.91 (s, 1H), 6.86 (d, *J* = 8.2 Hz, 1H); **^{13}C NMR** (100 MHz, $CDCl_3$) δ 167.0, 141.9, 134.6, 133.2, 131.7, 130.5, 129.3, 125.6, 123.6, 121.1, 113.5, 113.1, 112.1, 105.1, 87.5; **HRMS** (FD$^+$) Calcd for $C_{17}H_{10}BrNO$ $[M]^+$ 322.9940, Found 322.9941; **FT-IR** (KBr) $\tilde{\nu}$ (cm^{-1}) 2127, 1733, 1656, 1447.

(*E*)-5-chloro-3-(3-phenylprop-2-yn-1-ylidene)indolin-2-one (4k)

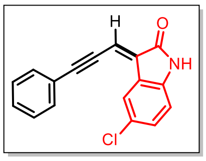

Yellow solid; yield 83% (23.1 mg); m.p. 232-234 °C; R_f = 0.38 (hexanes/EA = 7:3); **^{1}H NMR** (400 MHz, $CDCl_3$) δ 10.77 (br s, 1H), 7.98 (d, *J* = 2.1 Hz, 1H), 7.64 (dd, *J* = 5.9, 2.1 Hz, 2H), 7.57-7.51 (m, 3H), 7.36 (dd, *J* = 8.3, 2.2 Hz, 1H), 6.90 (d, *J* = 8.7 Hz, 2H); **^{13}C NMR** (100 MHz, $CDCl_3$) δ 167.1, 141.6, 134.6, 131.7, 130.4, 129.3, 125.5, 123.1, 122.8, 121.1, 113.5, 111.6, 105.1, 87.4; **HRMS** (FD^+) Calcd for $C_{17}H_{10}ClNO$ $[M]^+$ 279.0445, Found 279.0456; **FT-IR** (KBr) $\tilde{\nu}$ (cm^{-1}) 2182, 1717, 1609, 1589.

(*E*)-5-chloro-3-(3-(*p*-tolyl)prop-2-yn-1-ylidene)indolin-2-one (4l)

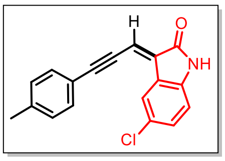

Yellow solid; yield 85% (24.9 mg); m.p. 230-232 °C; R_f = 0.40 (hexanes/EA = 7:3); **^{1}H NMR** (400 MHz, DMSO-d_6) δ 10.76 (br s, 1H), 7.98 (d, *J* = 2.1 Hz, 1H), 7.53 (d, *J* = 7.8 Hz, 2H), 7.37-7.34 (m, 3H), 6.91-6.89 (m, 2H), 2.37 (s, 3H); **^{13}C NMR** (100 MHz, $CDCl_3$) δ 167.2, 141.5, 140.8, 134.2, 131.7, 130.3, 129.9, 125.4, 123.2, 122.7, 118.2, 113.8, 111.6, 105.7, 87.2, 21.2; **HRMS** (FD^+) Calcd for $C_{18}H_{12}ClNO$ $[M]^+$ 293.0602, Found 293.0604; **FT-IR** (KBr) $\tilde{\nu}$ (cm^{-1}) 2178, 1721, 1611, 1595.

(*E*)-6-chloro-3-(3-phenylprop-2-yn-1-ylidene)indolin-2-one(4m)

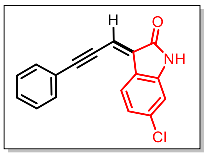

Light yellow solid; yield 80% (22.3 mg); m.p. 235-237 °C; R_f = 0.34 (hexanes/EA = 7:3); **^{1}H NMR** (400 MHz, DMSO-d_6) δ 10.78 (br s, 1H), 7.99 (d, *J* = 8.1 Hz, 1H), 7.69 (d, *J* = 6.7 Hz, 2H), 7.52 (br s, 3H), 7.15 (d, *J* = 8.3 Hz, 1H), 6.89 (d, *J* = 13.5 Hz, 2H); **^{13}C NMR** (100 MHz, DMSO-d_6) δ 167.4, 144.1, 135.0, 134.4, 132.0, 130.3, 129.1, 124.5, 121.7, 121.2, 120.4, 112.5, 110.1, 104.9, 87.4; **HRMS** (FD^+) Calcd for $C_{17}H_{10}ClNO$ $[M]^+$ 279.0445, Found 279.0455; **FT-IR** (KBr) $\tilde{\nu}$ (cm^{-1}) 2127, 1733, 1656, 1447.

(*E*)-6-chloro-3-(3-(*p*-tolyl)prop-2-yn-1-ylidene)indolin-2-one (4n)

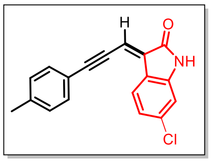

Light yellow solid; yield 83% (24.3 mg); m.p. 228-230 °C; R_f = 0.36 (hexanes/EA = 7:3); **^{1}H NMR** (400 MHz, DMSO-d_6) δ 10.77 (br s, 1H), 7.99 (d, *J* = 8.0 Hz, 1H), 7.58 (d, *J* = 7.7 Hz, 2H), 7.32 (d, *J* = 7.8 Hz, 2H), 7.15 (dd, *J* = 8.0, 1.9 Hz, 1H), 6.90-6.86 (m, 2H), 2.37 (s, 3H); **^{13}C NMR** (100 MHz, DMSO-d_6) δ 167.5, 144.0, 140.6, 134.9, 133.9, 132.0, 129.7, 124.4, 121.7, 120.5, 118.2, 112.7, 110.1, 105.5, 87.1, 21.2; **HRMS** (FD^+) Calcd for $C_{18}H_{12}ClNO$ $[M]^+$ 293.0602, Found 293.0613; **FT-IR** (KBr) $\tilde{\nu}$ (cm^{-1}) 2186, 1708, 1615, 1594.

(*E*)-5-phenyl-3-(3-phenylprop-2-yn-1-ylidene)indolin-2-one (4o)

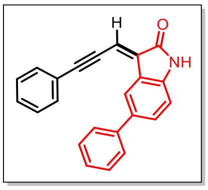

Light yellow solid; yield 87% (27.9 mg); m.p. 216-218 °C; R_f = 0.40 (hexanes/EA = 7:3); **^{1}H NMR** (400 MHz, $CDCl_3$) δ 8.49 (br s, 1H), 7.98 (br s, 1H), 7.63-7.59 (m, 4H), 7.53 (d, *J* = 8.0 Hz, 1H), 7.45-7.33 (m, 6H), 7.04-6.95 (m, 2H); **^{13}C NMR** (100 MHz, $CDCl_3$) δ 168.9, 141.0, 140.5, 135.8, 134.9, 132.2, 129.9, 129.3, 128.9, 128.8, 127.3, 126.8, 123.1, 122.4, 114.2, 110.2, 106.0, 88.1; **HRMS** (FD^+) Calcd for $C_{23}H_{15}NO$ $[M]^+$ 321.1148, Found 321.1155; **FT-IR** (KBr) $\tilde{\nu}$ (cm^{-1}) 2185, 1703, 1608, 1592.

(*E*)-5-(2-chlorophenyl)-3-(3-phenylprop-2-yn-1-ylidene)indolin-2-one (4p)

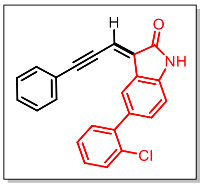

Light yellow solid; yield 84% (29.8 mg); m.p. 236-238 °C; R_f = 0.36 (hexanes/EA = 7:3); **^{1}H NMR** (400 MHz, $CDCl_3$) δ 8.36 (br s, 1H), 7.69 (br s, 1H), 7.51 (t, *J* = 7.0 Hz, 3H), 7.41-7.36 (m, 3H), 7.34-7.31 (m, 4H), 6.99 (br s, 1H), 6.94 (d, *J* = 8.0 Hz, 1H); **^{13}C NMR** (100 MHz, $CDCl_3$) δ 168.8, 140.5, 140.1, 134.6, 133.7, 132.5, 132.2, 131.4, 130.2, 129.9, 128.7, 127.1, 125.5, 122.5, 122.3, 114.4, 109.5, 106.1, 87.9; **HRMS** (FD^+) Calcd for $C_{23}H_{14}ClNO$ $[M]^+$ 355.0758, Found 355.0754; **FT-IR** (KBr) $\tilde{\nu}$ (cm^{-1}) 2177, 1715, 1607, 1588.

(*E*)-5-(2-chlorophenyl)-3-(3-(*p*-tolyl)prop-2-yn-1-ylidene)indolin-2-one (4q)

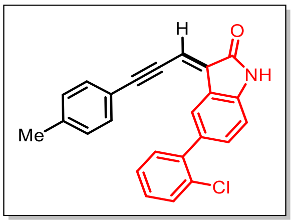

Light yellow solid; yield 85% (31.3 mg); m.p. 248-250 °C; R_f = 0.34 (hexanes/EA = 7:3); **^{1}H NMR** (400 MHz, DMSO-d_6) δ 10.76 (s, 1H), 8.19 (d, *J* = 1.7 Hz, 1H), 7.59 (dd, *J* = 7.6, 1.5 Hz, 1H), 7.50-7.45 (m, 2H), 7.43-7.41 (m, 2H), 7.41-7.38 (m, 2H), 7.22 (d, *J* = 8.0 Hz, 2H), 6.99 (d, *J* = 8.0 Hz, 1H), 6.85 (s, 1H), 2.33 (s, 3H); **^{13}C NMR** (100 MHz, DMSO-d_6) δ 167.6, 142.3, 140.5, 139.4, 135.1, 131.9, 131.6, 131.4, 131.2, 130.0, 129.6, 129.1, 127.6, 124.3, 121.6, 118.3, 112.4, 109.9, 104.8, 87.5, 21.1; **HRMS** (FD^+) Calcd for $C_{24}H_{16}ClNO$ $[M]^+$ 369.0914, Found 369.0916; **FT-IR** (KBr) $\tilde{\nu}$ (cm^{-1}) 2172, 1698, 1606, 1590.

(*E*)-5-(*p*-tolyl)-3-(3-(p-tolyl)prop-2-yn-1-ylidene)indolin-2-one (4r)

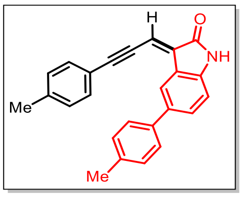

Light yellow solid; yield 88% (30.7 mg); m.p. 236-238 °C; R_f = 0.38 (hexanes/EA = 7:3); **^{1}H NMR** (400 MHz, $CDCl_3$) δ 8.48 (d, *J* = 1.8 Hz, 1H), 7.51 (d, *J* = 2.8 Hz, 3H), 7.49 (d, *J* = 2.8 Hz, 4H), 7.24 (s, 1H), 7.20 (d, *J* = 7.8 Hz, 2H), 6.98 (s, 1H), 6.91 (d, *J* = 8.0 Hz, 1H), 2.42 (s, 3H), 2.41 (s, 3H); **^{13}C NMR** (100 MHz, DMSO-d_6) δ 167.7, 142.0, 140.6, 137.2, 136.4,

135.3, 133.9, 131.7, 129.8, 129.6, 129.2, 126.0, 122.3, 121.3, 118.3, 112.2, 110.4, 104.9, 87.6, 21.2, 20.7; **HRMS** (FD$^+$) Calcd for $C_{25}H_{19}NO$ [M]$^+$ 349.1461, Found 349.1464; **FT-IR** (KBr) $\tilde{\nu}$ (cm^{-1}) 2187, 1710, 1606, 1486.

(*E*)-5-(naphthalen-2-yl)-3-(3-phenylprop-2-yn-1-ylidene)indolin-2-one (4s)

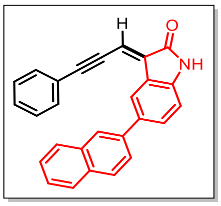

Light yellow solid; yield 87% (32.2 mg); m.p. 246-248 °C; R_f = 0.40 (hexanes/EA = 7:3); **^{1}H NMR** (400 MHz, DMSO-d_6) δ 10.76 (s, 1H), 8.48 (s, 1H), 8.14 (s, 1H), 7.99-7.89 (m, 3H), 7.79-7.74 (m, 2H), 7.69(d, *J* = 7.4 Hz, 2H), 7.57-7.49 (m, 3H), 7.44 (t, *J* = 7.4 Hz, 2H), 7.02 (d, *J* = 8.0 Hz, 1H), 6.89 (s, 1H); **^{13}C NMR** (100 MHz, DMSO-d_6) δ 167.6, 142.4, 137.5, 135.8, 133.7, 133.4, 132.0, 131.8, 130.2, 129.8, 129.1, 128.5, 127.8, 127.5, 126.5, 126.0, 124.8, 124.4, 122.5, 122.0, 121.4, 112.1, 110.6, 104.4, 87.9; **HRMS** (FD$^+$) Calcd for $C_{27}H_{17}NO$ [M]$^+$ 371.1305, Found 371.1312; **FT-IR** (KBr) $\tilde{\nu}$ (cm^{-1}) 2187, 1713, 1610, 1592.

(*E*)-5-(naphthalen-2-yl)-3-(3-(*p*-tolyl)prop-2-yn-1-ylidene)indolin-2-one (4t)

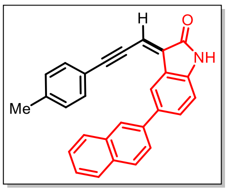

Light yellow solid; yield 88% (33.8 mg); m.p. 236-238 °C; R_f = 0.42 (hexanes/EA = 7:3); **^{1}H NMR** (400 MHz, DMSO-d_6) δ 10.75 (s, 1H), 8.49 (d, *J* = 1.8 Hz, 1H), 8.14 (d, *J* = 2.0 Hz, 1H), 8.01 (d, *J* = 8.5 Hz, 1H), 7.95 (d, *J* = 7.9 Hz, 1H), 7.91 (d, *J* = 8.0 Hz, 1H), 7.81-7.75 (m, 2H), 7.59-7.50 (m, 4H), 7.26 (d, *J* = 7.8 Hz, 2H), 7.03 (d, *J* = 8.0 Hz, 1H), 6.89 (s, 1H), 2.36 (s, 3H); **^{13}C NMR** (100 MHz, DMSO-d_6) δ 167.6, 142.3, 140.5, 137.5, 135.4, 133.7, 133.4, 132.0, 131.7, 129.8, 129.7, 128.5, 127.9, 127.6, 126.5, 126.0, 124.8, 124.4, 122.5, 121.9, 118.4, 112.4, 110.5, 105.0, 87.6, 21.2; **HRMS** (FD$^+$) Calcd for $C_{28}H_{19}NO$ [M]$^+$ 385.1461, Found 385.1467; **FT-IR** (KBr) $\tilde{\nu}$ (cm^{-1}) 2183, 1715, 1596, 1573.

(*E*)-5-cyclopropyl-3-(3-phenylprop-2-yn-1-ylidene)indolin-2-one (4u)

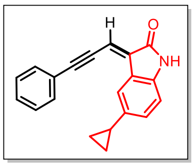

Light yellow solid; yield 82% (23.3 mg); m.p. 198-200 °C; R_f = 0.44 (hexanes/EA = 7:3); **^{1}H NMR** (400 MHz, $CDCl_3$) δ 7.92 (s, 1H), 7.90 (d, *J* = 1.7 Hz, 1H), 7.63-7.60 (m, 2H), 7.44-7.43 (m, 3H), 7.05 (dd, *J* = 8.0, 1.8 Hz, 1H), 6.92 (s, 1H), 6.76 (d, *J* = 8.0 Hz, 1H), 1.94-1.88 (m, 1H), 0.98-0.93 (m, 2H), 0.70-0.66 (m, 2H); **^{13}C NMR** (100 MHz, $CDCl_3$) δ 169.0, 139.1, 138.0, 135.1, 132.1, 132.1, 129.8, 128.8, 128.6, 122.7, 122.5, 121.5, 113.5, 109.7, 105.4, 87.9, 15.4, 8.7; **HRMS** (FD$^+$) Calcd for $C_{20}H_{15}NO$ [M]$^+$ 285.1148, Found 285.1152; **FT-IR** (KBr) $\tilde{\nu}$ (cm^{-1}) 2182, 1705, 1609, 1482.

(*E*)-5-methyl-3-(3-phenylprop-2-yn-1-ylidene)indolin-2-one (4v)

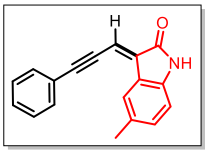

Light yellow solid; yield 91% (23.5 mg); m.p. 210-212 °C; R_f = 0.36 (hexanes/EA = 7:3); **^{1}H NMR** (400 MHz, DMSO-d_6) δ 10.50 (s, 1H), 7.84 (d, J = 1.7 Hz, 1H), 7.66-7.63 (m, 2H), 7.52- 7.50 (m, 3H), 7.11 (dd, J = 8.0, 1.7 Hz, 1H), 6.77 (t, J = 3.1 Hz, 2H), 2.30 (s, 3H); **^{13}C NMR (**100 MHz, DMSO-d_6) δ 167.5, 140.6, 135.9, 131.7, 131.4, 130.4, 130.1, 129.1, 123.9, 121.6, 121.5, 111.1, 109.8, 103.8, 87.7, 20.8; **HRMS** (FD$^+$**)** Calcd for $C_{18}H_{13}NO$ [M]$^+$ 259.0991, Found 259.0992; **FT-IR** (KBr) $\tilde{\nu}$ (cm^{-1}) 2185, 1714, 1610, 1591.

(*E*)-5-methyl-3-(3-(*p*-tolyl)prop-2-yn-1-ylidene)indolin-2-one (4w)

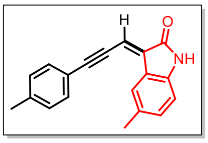

Light yellow solid; yield 92% (25.1 mg); m.p. 208-210 °C; R_f = 0.38 (hexanes/EA = 7:3); **^{1}H NMR** (400 MHz, DMSO-d_6) δ 10.48 (s, 1H), 7.83 (s, 1H), 7.52 (d, J = 7.7 Hz, 2H), 7.31 (d, J = 7.8 Hz, 2H), 7.11-7.08 (m, 1H), 6.76 (d, J = 7.4 Hz, 2H), 2.35 (s, 3H), 2.29 (s, 3H); **^{13}C NMR** (100 MHz, DMSO-d_6) δ 167.6, 140.5, 140.3, 135.5, 131.7, 131.3, 130.3, 129.8, 123.8, 121.8, 118.5, 111.4, 109.8, 104.3, 87.5, 21.2, 20.9; **HRMS** (FD$^+$) Calcd for $C_{19}H_{15}NO$ [M]$^+$ 273.1148, Found 273.1145; **FT-IR** (KBr) $\tilde{\nu}$ (cm^{-1}) 2182, 1707, 1613, 1598.

(*E*)-3-(3-(4-(*tert*-butyl)phenyl)prop-2-yn-1-ylidene)-5-methylindolin-2-one (4x)

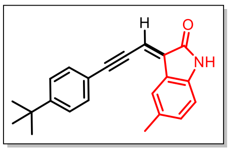

Light yellow solid; yield 92% (28.9 mg); m.p. 214-216 °C; R_f = 0.44 (hexanes/EA = 7:3); **^{1}H NMR** (400 MHz, DMSO-d_6) δ 10.50 (s, 1H), 7.86-7.85(m, 1H), 7.59-7.57(m, 2H), 7.54-7.52 (m, 2H), 7.12-7.10(m, 1H), 6.78-6.76 (m, 2H), 2.31 (s, 3H), 1.29 (s, 9H); **^{13}C NMR** (100 MHz, DMSO-d_6) δ 167.6, 153.1, 140.5, 135.5, 131.6, 131.3, 130.4, 126.1, 123.8, 121.7, 118.6, 111.4, 109.8, 104.2, 87.4, 34.8, 30.8, 20.9; **HRMS** (FD$^+$) Calcd for $C_{22}H_{21}NO$ [M]$^+$ 315.1618, Found 315.1619; **FT-IR** (KBr) $\tilde{\nu}$ (cm^{-1}) 2181, 1696, 1611, 1595.

(*E*)-5-fluoro-3-(3-phenylprop-2-yn-1-ylidene)indolin-2-one (4y)

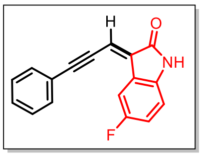

Yellow solid; yield 81% (21.3 mg); m.p. 236-238 °C; R_f = 0.38 (hexanes/EA = 7:3); **^{1}H NMR** (400 MHz, DMSO-d_6) δ 10.65 (s, 1H), 7.71 (dd, J = 8.5, 2.6 Hz, 1H), 7.66-7.64 (m, 2H), 7.54-7.51 (m, 3H), 7.16 (td, J = 8.9, 2.6 Hz, 1H), 6.90-6.86 (m, 2H); **^{13}C NMR** (100 MHz, DMSO-d_6) δ 167.4, 157.5 (d, $^1J_{FC}$ = 235.2 Hz), 139.2, 139.2, 135.2 (d, $^4J_{FC}$ = 3.2 Hz), 131.8, 130.4, 129.2, 122.4 (d, $^3J_{FC}$ = 9.0 Hz), 121.1, 117.3 (d, $^2J_{FC}$ = 23.5 Hz), 113.3, 111.0 (d, $^3J_{FC}$ = 8.0 Hz), 110.0 (d, $^2J_{FC}$ = 25.4 Hz),105.0, 87.2; **HRMS** (FD$^+$) Calcd for $C_{17}H_{10}FNO$ [M]$^+$ 263.0741, Found 263.0746; **FT-IR** (KBr) $\tilde{\nu}$ (cm^{-1}) 2182, 1718, 1611, 1590.

(*E*)-5-fluoro-3-(3-(*p*-tolyl)prop-2-yn-1-ylidene)indolin-2-one (4z)

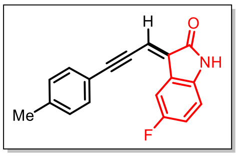

Yellow solid; yield 83% (22.9 mg); m.p. 208-210 °C; R_f = 0.40 (hexanes/EA = 7:3); **^{1}H NMR** (400 MHz, DMSO-d_6 δ 10.62 (s, 1H), 7.71-7.68 (m, 1H), 7.53 (d, J = 7.7 Hz, 2H), 7.32 (d, J = 7.6 Hz, 2H), 7.15 (td, J = 9.1, 2.6 Hz, 1H), 6.88-6.85 (m, 2H), 2.36 (s, 3H); **^{13}C NMR** (100 MHz, DMSO-d_6) δ 167.4, 157.5 (d, $^{1}J_{FC}$ = 235.1 Hz), 140.6, 139.1, 139.1, 134.7 (d, $^{4}J_{FC}$ = 3.2 Hz), 131.7, 129.8, 122.5 (d, $^{3}J_{FC}$ = 9.0 Hz), 118.2, 117.2 (d, $^{2}J_{FC}$ = 23.6 Hz), 113.6, 111.9 (d, $^{3}J_{FC}$ = 8.0 Hz), 109.9 (d, $^{2}J_{FC}$ = 25.4 Hz), 105.6, 86.9, 21.2; **HRMS** (FD^{+}) Calcd for $C_{18}H_{12}FNO$ $[M]^{+}$ 277.0897, Found 277.0894; **FT-IR** (KBr) $\tilde{\nu}$ (cm^{-1}) 2179, 1717, 1675, 1596.

(*E*)-3-(3-(4-(*tert*-butyl)phenyl)prop-2-yn-1-ylidene)-5-fluoroindolin-2-one (4aa)

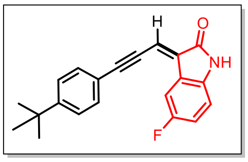

Yellow solid; yield 84% (26.8 mg); m.p. 200-202 °C; R_f = 0.38 (hexanes/EA = 7:3); **^{1}H NMR** (400 MHz, DMSO-d_6) δ 11.13 (s, 1H), 7.87 (d, J = 7.5 Hz, 1H), 7.62-7.51 (m, 4H), 7.24 (t, J = 9.3 Hz, 1H), 7.12-7.07 (m, 1H), 6.91 (s, 1H), 1.30 (s, 9H); **^{13}C NMR** (100 MHz, DMSO-d_6) δ 167.3, 153.3, 146.4 (d, $^{1}J_{FC}$ = 24.9 Hz), 134.5, 131.8, 129.5 (d, $^{3}J_{FC}$ = 12.5 Hz), 126.0, 124.5, 122.7 (d, $^{3}J_{FC}$ = 5.8 Hz), 119.3, 118.3, 117.7 (d, $^{2}J_{FC}$ = 17.3 Hz), 113.7, 105.6, 87.0, 34.8, 30.8; **HRMS** (FD^{+}) Calcd for $C_{21}H_{18}FNO$ $[M]^{+}$ 319.1366, Found 319.1364; **FT-IR** (KBr) $\tilde{\nu}$ (cm^{-1}) 2179, 1717, 1675, 1596.

(*E*)-7-fluoro-3-(3-phenylprop-2-yn-1-ylidene)indolin-2-one (4ab)

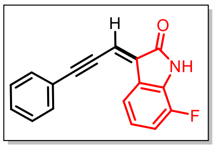

Light yellow solid; yield 80% (21.0 mg); m.p. 202-204 °C; R_f = 0.40 (hexanes/EA = 7:3); **^{1}H NMR** (400 MHz, DMSO-d_6) δ 11.15 (s, 1H), 7.87 (d, J = 7.5 Hz, 1H), 7.69 (d, J = 6.8Hz, 2H), 7.54-7.49 (m, 3H), 7.25 (t, J = 9.2 Hz, 1H), 7.13-7.09 (m, 1H), 6.92 (s, 1H); **^{13}C NMR** (100 MHz, DMSO-d_6) δ 167.3, 146.4 (d, $^{1}J_{FC}$ = 240.9 Hz), 134.8 (d, $^{4}J_{FC}$ = 4.1 Hz), 132.0, 130.3, 129.6 (d, $^{3}J_{FC}$ = 12.6 Hz), 129.1, 124.4, 124.4, 122.77 (d, $^{3}J_{FC}$ = 5.9 Hz), 121.2, 119.4 (d, $^{4}J_{FC}$ = 3.2 Hz), 117.8 (d, $^{2}J_{FC}$ = 17.4 Hz), 113.4, 105.1, 87.3; **HRMS** (FD^{+}) Calcd for $C_{17}H_{10}FNO$ $[M]^{+}$ 263.0740, Found 263.0741; **FT-IR** (KBr) $\tilde{\nu}$ (cm^{-1}) 2181, 1711, 1617, 1589.

(*E*)-7-fluoro-3-(3-(*p*-tolyl)prop-2-yn-1-ylidene)indolin-2-one (4ac)

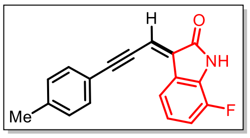

Yellow solid; yield 82% (22.7 mg); m.p. 210-212 °C; R_f = 0.42 (hexanes/EA = 7:3); **^{1}H NMR** (400 MHz, DMSO-d_6) δ 11.14 (s, 1H), 7.87 (dd, J = 7.5, 0.9 Hz, 1H), 7.58 (d, J = 8.1 Hz, 2H), 7.32 (d, J = 7.8 Hz, 2H), 7.26-7.21 (m, 1H), 7.13-7.08 (m, 1H), 6.91 (s, 1H), 2.37 (s, 3H); **^{13}C NMR** (100

MHz, DMSO-d_6) δ 167.3, 146.4 (d, $^1J_{FC}$ = 240.9 Hz), 140.6, 134.3 (d, $^4J_{FC}$ = 3.8 Hz), 131.9, 129.7, 129.5 (d, $^3J_{FC}$ = 12.8 Hz), 124.4 (d, $^4J_{FC}$ = 4.7 Hz), 122.7 (d, $^3J_{FC}$ = 5.9 Hz), 119.3 (d, $^4J_{FC}$ = 3.2 Hz), 118.2, 117.7 (d, $^2J_{FC}$ = 17.3 Hz), 113.7, 105.7, 87.0, 21.23; **HRMS** (FD$^+$) Calcd for $C_{18}H_{12}FNO$ [M]$^+$ 277.0897, Found 277.0899; **FT-IR** (KBr) $\tilde{\nu}$ (cm^{-1}) 2170, 1705, 1614, 1593.

(*E*)-3-(3-(4-(*tert*-butyl)phenyl)prop-2-yn-1-ylidene)-7-fluoroindolin-2-one (4ad)

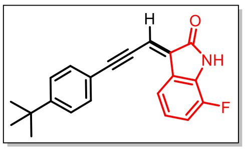

Light Yellow solid; yield 85% (27.1 mg); m.p. 204-206 °C; R_f = 0.40 (hexanes/EA = 7:3); **^{1}H NMR** (400 MHz, DMSO-d_6) δ 7.95-7.92 (m, 1H), 7.90 (br s, 1H), 7.54 (d, *J* = 8.2 Hz, 2H), 7.46-7.43 (m, 2H), 7.08-6.99 (m, 3H), 1.34 (s, 9H); **^{13}C NMR** (100 MHz, DMSO-d_6) δ 167.9, 153.7, 147.0 (d, $^1J_{FC}$ = 242.2 Hz), 133.7, 132.0, 128.2 (d, $^3J_{FC}$ = 12.7 Hz), 125.9, 125.3 (d, $^4J_{FC}$ = 4.2 Hz), 123.0 (d, $^3J_{FC}$ = 5.8 Hz), 119.8 (d, $^4J_{FC}$ = 3.4 Hz), 119.2, 117.2 (d, $^2J_{FC}$ = 17.2 Hz), 116.1, 107.2, 87.2, 35.1, 31.2; **HRMS** (FD$^+$) Calcd for $C_{21}H_{18}FNO$ [M]$^+$ 319.1367, Found 319.1373; **FT-IR** (KBr) $\tilde{\nu}$ (cm^{-1}**)** 2181, 1716, 1613, 1595.

(*E*)-3-(3-(thiophen-3-yl)prop-2-yn-1-ylidene)indolin-2-one (4ae)

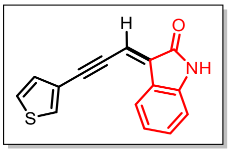

Light yellow solid; yield 88% (22.1 mg); m.p. 198-200 °C; R_f = 0.38 (hexanes/EA = 7:3); **^{1}H NMR** (400 MHz, DMSO-d_6) δ 10.59 (s, 1H), 8.17-8.16 (m, 1H), 8.01 (d, *J* = 7.5 Hz, 1H), 7.75-7.73 (m, 1H), 7.39 (dd, *J* = 5.0, 1.2 Hz, 1H), 7.30 (td, *J* = 7.7, 1.2 Hz, 1H), 7.07 (td, *J* = 7.5, 1.0 Hz, 1H), 6.87 (d, *J* = 7.6 Hz, 1H), 6.79 (s, 1H); **^{13}C NMR** (100 MHz, DMSO-d_6) δ 167.5, 142.7, 135.1, 132.5, 130.9, 129.5, 127.7, 123.2, 121.8, 121.5, 120.4, 111.6, 110.0, 99.9, 87.2; **HRMS** (FD$^+$) Calcd for $C_{15}H_9NOS$ [M]$^+$ 251.0399, Found 251.0394; **FT-IR** (KBr) $\tilde{\nu}$ (cm^{-1}) 2181, 1704, 1604, 1512.

(*E*)-5-methyl-3-(3-(thiophen-3-yl)prop-2-yn-1-ylidene)indolin-2-one (4af)

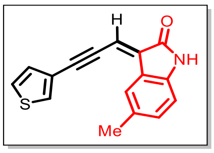

Light yellow solid; yield 90% (23.8 mg); m.p. 208-210 °C; R_f = 0.44 (hexanes/EA = 7:3); **^{1}H NMR** (400 MHz, DMSO-d_6) δ 10.48 (s, 1H), 8.12-8.11 (m, 1H), 7.83 (s, 1H), 7.76-7.74 (m, 1H), 7.36 (d, *J* = 5.0 Hz, 1H), 7.11 (d, *J* = 7.9 Hz, 1H), 6.78-6.75 (m, 2H), 2.31 (s, 3H); **^{13}C NMR** (100 MHz, DMSO-d_6) δ 167.5, 140.4, 135.4, 132.4, 131.3, 130.4, 129.3, 127.8, 123.7, 121.6, 120.4, 111.3, 109.8, 99.7, 87.4, 20.8; **HRMS** (FD$^+$) Calcd for $C_{16}H_{11}NOS$ [M]$^+$ 265.0556, Found 265.0563; **FT-IR** (KBr) $\tilde{\nu}$ (cm^{-1}) 2178, 1691, 1603, 1515.

(*E*)-5-fluoro-3-(3-(thiophen-3-yl)prop-2-yn-1-ylidene)indolin-2-one (4ag)

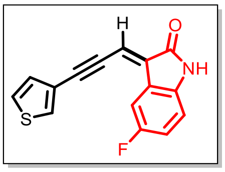

Yellow solid; yield 85% (22.8 mg); m.p. 214-216 °C; R_f = 0.44 (hexanes/EA = 7:3); **^{1}H NMR** (400 MHz, DMSO-d_6) δ 10.64 (s, 1H), 8.16-8.16 (m, 1H), 7.77-7.75 (m, 1H), 7.71-7.68 (m, 1H), 7.36 (d, J = 5.0 Hz, 1H), 7.19-7.14 (m, 1H), 6.89-6.86 (m, 2H); **^{13}C NMR** (100 MHz, DMSO-d_6) δ 167.4, 157.5 (d, $^1J_{FC}$ = 235.20 Hz), 139.0, 134.7 (d, $^4J_{FC}$ = 3.1 Hz), 132.8, 129.3, 127.9, 122.4 (d, $^3J_{FC}$ = 9.0 Hz), 120.1, 117.2 (d, $^2J_{FC}$ = 23.6 Hz), 113.5, 110.9 (d, $^3J_{FC}$ = 8.2 Hz), 109.9 (d, $^2J_{FC}$ = 25.4 Hz), 101.0, 86.9; **HRMS** (FD$^+$) Calcd for $C_{15}H_8FNOS$ $[M]^+$ 269.0305, Found 269.0298; **FT-IR** (KBr) $\tilde{\nu}$ (cm^{-1}) 2184, 1703, 1608, 1514.

(*E*)-7-fluoro-3-(3-(thiophen-3-yl)prop-2-yn-1-ylidene)indolin-2-one (4ah)

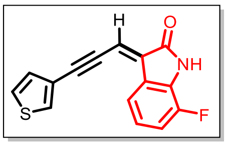

Light yellow solid; yield 81% (21.8 mg); m.p. 232-234 °C; R_f = 0.44 (hexanes/EA = 7:3); **^{1}H NMR** (400 MHz, $CDCl_3$) δ 7.90 (d, J = 7.2 Hz, 1H), 7.70-7.66 (m, 2H), 7.39-7.37 (m, 1H), 7.27 (br s, 1H), 7.08-7.00 (m, 3H); **^{13}C NMR** (100 MHz, $CDCl_3$) δ 167.9, 147.0 (d, $^1J_{FC}$ = 242.1 Hz), 133.8 (d, $^4J_{FC}$ = 4.0 Hz), 131.4, 129.8, 128.2 (d, $^3J_{FC}$ = 12.7 Hz), 126.3, 125.2 (d, $^4J_{FC}$ = 3.8 Hz), 123.0 (d, $^3J_{FC}$ = 5.8 Hz), 121.5, 119.7 (d, $^4J_{FC}$ = 3.4 Hz), 117.4 (d, $^2J_{FC}$ = 17.2 Hz), 115.7, 101.9, 87.4; **HRMS** (FD$^+$) Calcd for $C_{15}H_8FNOS$ $[M]^+$ 269.0305, Found 269.0313; **FT-IR** (KBr) $\tilde{\nu}$ (cm^{-1}) 2181, 1706, 1605, 1466.

(*E*)-5-chloro-3-(3-(thiophen-3-yl)prop-2-yn-1-ylidene)indolin-2-one (4ai)

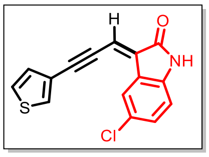

Light yellow solid; yield 78% (22.2 mg); m.p. 240-242 °C; R_f = 0.40 (hexanes/EA = 7:3); **^{1}H NMR** (400 MHz, DMSO-d_6) δ 10.74 (s, 1H), 8.11 (t, J = 1.9 Hz, 1H), 7.96 (d, J = 2.0 Hz, 1H), 7.79-7.77 (m, 1H), 7.36-7.33 (m, 2H), 6.90 (t, J = 4.4 Hz, 2H); **^{13}C NMR** (100 MHz, DMSO-d_6) δ 167.1, 141.4, 134.1, 132.8, 130.3, 129.1, 128.1, 125.4, 123.1, 122.5, 120.1, 113.7, 111.5, 101.0, 87.1; **HRMS** (FD$^+$) Calcd for $C_{15}H_8ClNOS$ $[M]^+$ 285.0010, Found 285.0020; **FT-IR** (KBr) $\tilde{\nu}$ (cm^{-1}) 2179, 1709, 1603, 1514.

(*E*)-3-(non-2-yn-1-ylidene)indolin-2-one (4aj)

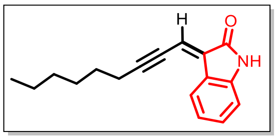

Light yellow solid; yield 56% (14.1 mg); m.p. 110-112 °C; R_f = 0.62 (hexanes/EA = 7:3); **^{1}H NMR** (400 MHz, $CDCl_3$) δ 8.29 (s, 1H), 8.03 (dd, J = 7.6, 1.2 Hz, 1H), 7.23 (dd, J = 7.7, 1.2 Hz, 1H), 7.02 (td, J = 7.6, 1.0 Hz, 1H), 6.86 (dt, J = 7.7, 0.8 Hz, 1H), 6.76 (t, J = 2.6 Hz, 1H), 2.59 (td, J = 7.1, 2.6 Hz, 2H), 1.72-1.68 (m, 1H), 1.66-1.64 (m, 1H), 1.53-1.45 (m, 2H), 1.36-1.32 (m, 4H), 0.93-0.89 (m, 3H); **^{13}C NMR** (100 MHz, $CDCl_3$) δ 169.3, 141.0, 134.4, 130.2, 123.6, 122.7, 122.2,

115.4, 109.9, 109.1, 79.1, 31.4, 28.8, 28.5, 22.6, 20.6, 14.1; **HRMS** (FD$^+$) Calcd for $C_{17}H_{19}NO$ [M]$^+$ 253.1461, Found 253.1461; **FT-IR** (KBr) $\tilde{\nu}$ (cm^{-1}) 2927, 2856, 2200, 1713, 1610, 1462.

(*E*)-3-(3-(trimethylsilyl)prop-2-yn-1-ylidene)indolin-2-one (4ak)

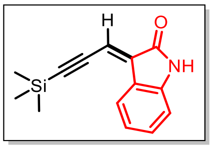

Yellow solid; yield 82% (19.7 mg); m.p. 154-156 °C; R_f = 0.56 (hexanes/EA = 7:3); **^{1}H NMR** (400 MHz, $CDCl_3$) δ 8.21 (br s, 1H), 8.06 (d, *J* = 7.6 Hz, 1H), 7.28-7.24 (m, 1H), 7.03 (td, *J* = 7.6, 0.9 Hz, 1H), 6.86 (d, *J* = 7.8 Hz, 1H), 6.70 (s, 1H), 0.32 (s, 9H); **^{13}C NMR** (100 MHz, $CDCl_3$) δ 168.9, 141.4, 136.1, 130.8, 124.2, 122.4, 113.5, 113.2, 110.0, 102.2, -0.2; **HRMS** (FD$^+$) Calcd for $C_{14}H_{15}NOSi$ [M]$^+$ 241.0917, Found 241.0921; **FT-IR** (KBr) $\tilde{\nu}$ (cm^{-1}) 2150, 1709, 1611, 1484, 846.

(*E*)-5-fluoro-3-(3-(trimethylsilyl)prop-2-yn-1-ylidene)indolin-2-one (4al)

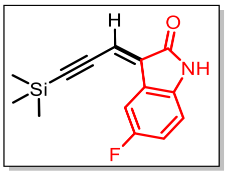

Light yellow solid; yield 78% (20.2 mg); m.p. 158-160 °C; R_f = 0.58 (hexanes/EA = 7:3); **^{1}H NMR** (400 MHz, $CDCl_3$) δ 7.96 (br s, 1H), 7.81 (dd, *J* = 8.6, 2.6 Hz, 1H), 6.98 (td, *J* = 8.7, 2.6 Hz, 1H), 6.78 (dd, *J* = 8.5, 4.2 Hz, 1H), 6.74 (s, 1H), 0.33 (s, 9H); **^{13}C NMR** (100 MHz, $CDCl_3$) δ 167.7, 158.8 (d, $^1J_{FC}$ = 238.2 Hz), 137.3, 135.8, 123.4 (d, $^3J_{FC}$ = 9.1 Hz), 117.1 (d, $^2J_{FC}$ = 24.0 Hz), 115.0, 114.7, 111.7 (d, $^2J_{FC}$ = 25.9 Hz), 110.4 (d, $^3J_{FC}$ = 7.9 Hz), 101.8, -0.2; **HRMS** (FD$^+$) Calcd for $C_{14}H_{14}FNOSi$ [M]$^+$ 259.0823, Found 259.0816; **FT-IR** (KBr) $\tilde{\nu}$ (cm^{-1}) 2143, 1709, 1617, 1481, 808.

(*E*)-1-benzyl-3-(3-phenylprop-2-yn-1-ylidene)indolin-2-one (5a)

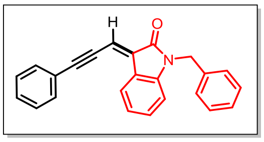

Light yellow solid; yield 70% (23.4 mg); m.p. 132-134 °C; R_f = 0.42 (hexanes/EA = 7:3); **^{1}H NMR** (400 MHz, $CDCl_3$) δ 8.16-8.14 (m, 1H), 7.62-7.59 (m, 2H), 7.44-7.41 (m, 3H), 7.32-7.31 (m, 4H), 7.26 (s, 1H), 7.21 (td, *J* = 7.7, 1.2 Hz, 1H), 7.07-7.03 (m, 2H), 6.71 (dt, *J* = 7.8, 0.8 Hz, 1H), 4.96 (s, 2H); **^{13}C NMR** (100 MHz, $CDCl_3$) δ 167.6, 143.2, 136.0, 134.5, 132.1, 130.5, 129.8, 128.9, 128.8, 127.7, 127.4, 123.8, 122.5, 122.1, 113.9, 109.2, 105.4, 87.7, 43.8; **HRMS** (FD$^+$) Calcd for $C_{24}H_{17}NO$ [M]$^+$ 335.1305, Found 335.1306; **FT-IR** (KBr) $\tilde{\nu}$ (cm^{-1}) 2184, 1706, 1607, 1491.

(*E*)-1-benzyl-3-(3-(*p*-tolyl)prop-2-yn-1-ylidene)indolin-2-one (5b)

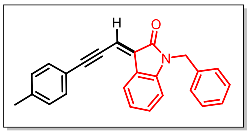

Light yellow solid; yield 73% (25.4 mg); m.p. 198-200 °C; **^{1}H NMR** (400 MHz, $CDCl_3$) δ 8.16-8.14 (m, 1H), 7.51-7.49 (m, 2H), 7.31-7.30 (m, 4H), 7.25 (s, 1H), 7.24-7.18 (m, 3H), 7.05 (td, *J* = 7.6 Hz, 1.0 Hz,

2H), 6.70 (dt, J = 7.7, 0.8 Hz, 1H), 4.96 (s, 2H), 2.41 (s, 3H); **^{13}C NMR** (100 MHz, $CDCl_3$) δ 167.7, 143.1, 140.4, 136.0, 134.1, 132.1, 130.3, 129.6, 128.9, 127.7, 127.4, 123.8, 122.4, 122.2, 119.5, 114.3, 109.1, 106.0, 87.5, 43.8, 21.8; **HRMS** (FD^+) Calcd for $C_{25}H_{19}NO$ $[M]^+$ 349.1461, Found 349.1461; **FT-IR** (KBr) $\tilde{\nu}$ (cm^{-1}) 2182, 1708, 1607, 1491.

(*E*)-1-methyl-3-(3-phenylprop-2-yn-1-ylidene)indolin-2-one (5c)

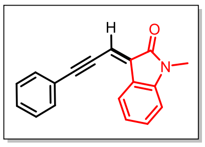

Yellow solid; yield 68% (17.6 mg); m.p. 95-97 °C; R_f = 0.48 (hexanes/EA = 7:3); **^{1}H NMR** (400 MHz, $CDCl_3$) δ 8.13 (dd, J = 7.5, 1.2 Hz, 1H), 7.61-7.59 (m, 2H), 7.43-7.40 (m, 3H), 7.32 (td, J = 7.6, 1.2 Hz, 1H), 7.09 (td, J = 7.6, 1.0 Hz, 1H), 6.97 (s, 1H), 6.82 (d, J = 7.7 Hz, 1H), 3.25 (s, 3H); **^{13}C NMR** (100 MHz, $CDCl_3$) δ 167.5, 144.1, 134.7, 132.1, 130.5, 129.7, 128.8, 123.7, 122.5, 122.4, 121.9, 113.5, 108.1, 105.0, 87.7, 26.2; **HRMS (FD^+):** Calcd for $C_{18}H_{13}NO$ $[M]^+$ 259.0992, Found 259.0996; **FT-IR** (KBr) $\tilde{\nu}$ (cm^{-1}) 2183, 1709, 1607, 1490.

(*E*)-1-ethyl-3-(3-phenylprop-2-yn-1-ylidene)indolin-2-one (5d)

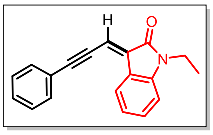

Light yellow solid; yield 70% (19.1 mg); m.p. 104-106 °C; R_f = 0.46 (hexanes/EA = 7:3); **^{1}H NMR** (400 MHz, $CDCl_3$) δ 8.15 (dd, J = 7.5, 1.2 Hz, 1H), 7.61-7.59 (m, 2H), 7.43-7.41 (m, 3H), 7.31 (td, J = 7.7, 1.2 Hz, 1H), 7.07 (td, J = 7.6, 1.0 Hz, 1H), 6.97 (s, 1H), 6.84 (d, J = 7.8 Hz, 1H), 3.80 (q, J = 7.2 Hz, 2H), 1.28 (t, J = 7.1 Hz, 3H); **^{13}C NMR** (100 MHz, $CDCl_3$) δ 167.1, 143.2, 134.8, 132.1, 130.5, 129.7, 128.8, 123.9, 122.5, 122.2, 122.1, 113.4, 108.3, 105.0, 87.7, 34.7, 13.0; **HRMS** (FD^+) Calcd for $C_{19}H_{15}NO$ $[M]^+$ 273.1148, Found 273.1154; **FT-IR** (KBr) $\tilde{\nu}$ (cm^{-1}) 2185, 1711, 1608, 1491.

(*E*)-3-(3-phenylprop-2-yn-1-ylidene)-1-(prop-2-yn-1-yl)indolin-2-one (5e)

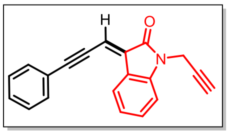

Yellow solid; yield 72% (20.3 mg); m.p. 132-134 °C; R_f = 0.48 (hexanes/EA = 7:3); **^{1}H NMR** (400 MHz, $CDCl_3$) δ 8.16 (d, J = 7.5 Hz, 1H), 7.61-7.59 (m, 2H), 7.44-7.41 (m, 3H), 7.36 (td, J = 7.6, 1.1 Hz, 1H), 7.13 (td, J = 7.6, 1.0 Hz, 1H), 7.05 (d, J = 7.8 Hz, 1H), 7.01 (s, 1H), 4.55 (d, J = 2.5 Hz, 2H), 2.24 (t, J = 2.5 Hz, 1H); **^{13}C NMR** (100 MHz, $CDCl_3$) δ 166.6, 142.1, 134.2, 132.2, 130.6, 129.9, 128.8, 123.9, 122.9, 122.4, 122.0, 114.3, 109.2, 105.7, 87.7, 77.0, 72.4, 29.2; **HRMS** (FD^+) Calcd for $C_{20}H_{13}NO$ $[M]^+$ 283.0991, Found 283.0990; **FT-IR** (KBr) $\tilde{\nu}$ (cm^{-1}) 2084, 1743, 1567, 1475.

(*E*)-1-acetyl-3-(3-phenylprop-2-yn-1-ylidene)indolin-2-one (5f)

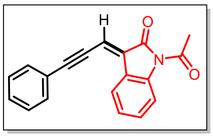

Light yellow solid; yield 66% (18.9 mg); m.p. 148-150 °C; R_f = 0.42 (hexanes/EA = 7:3); **^{1}H NMR** (400 MHz, $CDCl_3$) δ 8.31-8.27 (m, 2H), 7.62-7.60 (m, 2H), 7.46-7.42 (m, 3H), 7.41-7.38 (m, 1H), 7.28-7.24 (m, 1H), 7.03 (s, 1H), 2.73 (s, 3H); **^{13}C NMR** (100 MHz, $CDCl_3$) δ 170.8, 167.7, 140.2, 133.0, 132.2, 131.0, 130.1, 128.9, 125.1, 123.2, 122.7, 122.2, 116.7, 115.0, 107.5, 87.8, 26.8; **HRMS** (FD^+) Calcd for $C_{19}H_{13}NO_2$ $[M]^+$ 287.0941, Found 287.0945; **FT-IR** (KBr) $\tilde{\nu}$ (cm^{-1}) 2185, 1737, 1713, 1615, 1589.

***Tert*-butyl (*E*)-2-oxo-3-(3-phenylprop-2-yn-1-ylidene)indoline-1-carboxylate (5g)**

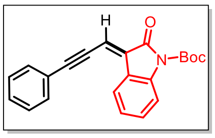

Light yellow solid; yield 68% (23.4 mg); m.p. 136-138 °C; R_f = 0.40 (hexanes/EA = 7:3); **^{1}H NMR** (400 MHz, $CDCl_3$) δ 8.27-8.25 (m, 1H), 7.92-7.89 (m, 1H), 7.61-7.59 (m, 2H), 7.45-7.39 (m, 3H), 7.37 (dd, *J* = 8.0, 1.3 Hz, 1H), 7.21 (td, *J* = 7.6, 1.0 Hz, 1H), 7.01 (s, 1H), 1.66 (s, 9H); **^{13}C NMR** (100 MHz, $CDCl_3$) δ 165.8, 149.3, 140.0, 133.2, 132.2, 130.8, 130.0, 128.8, 124.4, 123.4, 122.3, 115.2, 114.5, 106.9, 87.8, 84.5, 28.2; **HRMS** (FD^+) Calcd for $C_{22}H_{19}NO_3$ $[M]^+$ 345.1359, Found 345.1359; **FT-IR** (KBr) $\tilde{\nu}$ (cm^{-1}) 2184, 1732, 1603, 1490.

Methyl (*E*)-4-((*E*)-2-oxoindolin-3-ylidene)but-2-enoate (6a)

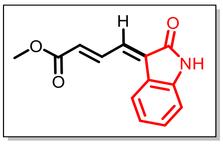

Yellow solid; yield 40% (9.1 mg); m.p. 198-200 °C; R_f = 0.48 (hexanes/EA = 7:3); **^{1}H NMR** (400 MHz, $CDCl_3$) δ 8.83 (dd, *J* = 15.5, 11.8 Hz, 1H), 7.97 (br s, 1H), 7.44 (dd, *J* = 7.6, 1.1 Hz, 1H), 7.27-7.23 (m, 1H), 7.13 (dt, *J* = 11.9, 0.8 Hz, 1H), 7.01 (td, *J* = 7.6, 1.0 Hz, 1H), 6.85 (d, *J* = 7.8 Hz, 1H), 6.26 (dd, *J* = 15.5, 1.0 Hz, 1H), 3.82 (s, 3H); **^{13}C NMR** (100 MHz, $CDCl_3$) δ 168.0, 166.7, 141.0, 138.0, 131.7, 130.8, 129.4, 123.2, 122.3, 120.9, 110.1, 52.1; **HRMS** (FD^+) Calcd for $C_{13}H_{11}NO_3$ $[M]^+$ 229.0733, Found 229.0726; **FT-IR** (KBr) $\tilde{\nu}$ (cm^{-1}) 1705, 1604, 1467.

Ethyl (*E*)-4-((*E*)-2-oxoindolin-3-ylidene)but-2-enoate (6b)

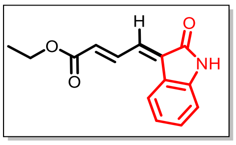

Yellow solid; yield 30% (7.2 mg); m.p. 172-174 °C; R_f = 0.50 (hexanes/EA = 7:3); **^{1}H NMR** (400 MHz, $CDCl_3$) δ 8.11-8.04 (m, 1H), 7.93 (s, 1H), 7.72 (d, *J* = 7.6 Hz, 1H), 7.31-7.29 (m, 1H), 7.28-7.27 (m, 1H), 7.06 (td, *J* = 7.7, 0.9 Hz, 1H), 6.87 (d, *J* = 7.8 Hz, 1H), 6.42-6.38 (m, 1H), 4.29 (q, J = 7.1 Hz, 2H), 1.35 (t, *J* = 7.1 Hz, 3H); **^{13}C NMR** (100 MHz, $CDCl_3$) δ 169.1, 166.1, 141.8, 137.3, 131.8, 131.2, 131.0, 130.8, 125.1, 122.7, 122.0, 110.4, 61.2, 14.4; **HRMS** (FD^+) Calcd for $C_{14}H_{13}NO_3$ $[M]^+$ 243.0889, Found 243.0889; **FT-IR** (KBr) $\tilde{\nu}$ (cm^{-1}) 1702, 1611, 1491.

Methyl (*E*)-4-((*E*)-2-oxoindolin-3-ylidene)but-2-enoate (6c)

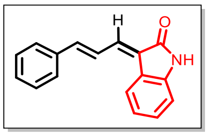

Yellow solid; yield 32% (7.9 mg); m.p. 196-198 °C; R_f = 0.40 (hexanes/EA = 7:3); **^{1}H NMR** (400 MHz, $CDCl_3$) δ 8.55 (dd, *J* = 15.7, 11.6 Hz, 1H), 7.61 (d, *J* = 7.0 Hz, 2H), 7.45 (d, *J* = 7.6 Hz, 2H), 7.40-7.36 (m, 2H), 7.34-7.30 (m, 2H), 7.20 (td, *J* = 7.7, 1.1 Hz, 1H), 7.05-6.99 (m, 2H), 6.83 (d, *J* = 7.8 Hz, 1H); **^{13}C NMR** (100 MHz, $CDCl_3$) δ 168.8, 143.3, 139.7, 136.6, 136.5, 129.5, 128.9, 128.8, 128.0, 124.7, 124.5, 121.9, 119.7, 109.6; **HRMS** (FD^+) Calcd for $C_{17}H_{13}NO$ $[M]^+$ 247.0991, Found 247.0986; **FT-IR** (KBr) $\tilde{\nu}$ (cm^{-1}) 1732, 1604, 1589.

(*E*)-5-(2-(4-(benzo[d]isothiazol-3-yl)piperazin-1-yl)ethyl)-6-chloro-3-(3-phenylprop-2-yn-1-ylidene)indolin-2-one (7a)

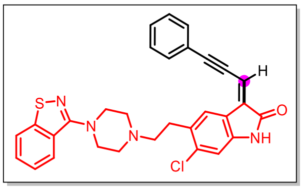

Brown solid; yield 70% (36.6 mg); m.p. 202-204 °C; R_f = 0.38 (hexanes/EA = 7:3);**^{1}H NMR** (400 MHz, $CDCl_3$) δ 8.06 (s, 1H), 7.88 (d, *J* = 8.2 Hz, 1H), 7.81 (d, *J* = 8.1 Hz, 1H), 7.71 (s, 1H), 7.61-7.59 (m, 2H), 7.49-7.45 (m, 1H), 7.43-7.40 (m, 3H), 7.37-7.33 (m, 1H), 6.95 (s, 1H), 6.90 (s, 1H), 3.57 (t, *J* = 4.8 Hz, 4H), 3.02-2.98 (m, 2H), 2.77 (t, *J* = 4.9 Hz, 4H), 2.74-2.70 (m, 2H); **^{13}C NMR** (100 MHz, $CDCl_3$) δ 168.5, 163.9, 152.9, 140.3, 135.7, 134.0, 132.1, 131.5, 130.1, 128.9, 128.1, 127.7, 125.9, 124.0, 122.3, 121.7, 120.7, 114.2, 111.1, 106.1, 87.8, 58.8, 53.1, 50.1, 31.0; **HRMS** (FD^+) Calcd for $C_{30}H_{25}ClN_4OS$ $[M]^+$ 524.1432, Found 524.1441; **FT-IR** (KBr) $\tilde{\nu}$ (cm^{-1}) 2182, 1706, 1668, 1608, 1589.

(*E*)-5-(2-(4-(benzo[*d*]isothiazol-3-yl)piperazin-1-yl)ethyl)-6-chloro-3-(3-(*p*-tolyl)prop-2-yn-1-ylidene)indolin-2-one (7b)

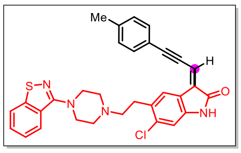

Brown solid; yield 74% (39.8 mg); m.p. 210-212 °C; R_f = 0.38 (hexanes/EA = 7:3); **^{1}H NMR** (400 MHz, $CDCl_3$) δ 8.06 (s, 1H), 7.89 (dt, *J* = 8.2, 1.0 Hz, 1H), 7.83-7.80 (m, 2H), 7.50-7.45 (m, 3H), 7.37-7.33 (m, 1H), 7.23-7.20 (m, 2H), 6.95 (s, 1H), 6.89 (s, 1H), 3.58 (t, *J* = 4.8 Hz, 4H), 3.02-2.98 (m, 2H), 2.78 (t, *J* = 4.9 Hz, 4H), 2.75-2.70 (m, 2H), 2.38(s, 3H); **^{13}C NMR** (100 MHz, $CDCl_3$) δ 168.6, 163.9, 152.9, 140.7, 140.3, 135.5, 133.5, 132.0, 131.4, 129.7, 128.1, 127.7, 125.8, 124.0, 121.8, 120.7, 119.3, 114.5, 111.0, 106.7, 87.6, 58.9, 53.1, 50.1, 31.0, 21.8; **HRMS** (FD^+) Calcd for $C_{31}H_{27}ClN4OS$ $[M]^+$ 538.1588, Found 538.1589; **FT-IR** (KBr) $\tilde{\nu}$ (cm^{-1}) 2180, 1704, 1626, 1611, 1597.

(*E*)-4-(2-(dipropylamino)ethyl)-3-(3-phenylprop-2-yn-1-ylidene)indolin-2-one (7c)

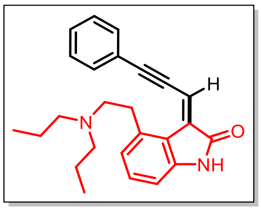

Yellow solid; yield 62% (23.1 mg); m.p. 122-124 °C; R_f = 0.36 (hexanes/EA = 7:3); **^{1}H NMR** (400 MHz, $CDCl_3$) δ 8.05 (s, 1H), 7.64-

7.62 (m, 2H), 7.37-7.36 (m, 3H), 7.13 (t, *J* = 7.7 Hz, 1H), 7.04 (s, 1H), 6.81 (d, *J* = 7.7 Hz, 1H), 6.71 (d, *J* = 7.7 Hz, 1H), 2.97-2.93 (m, 2H), 2.73-2.69 (m, 2H), 2.49 (t, *J* = 7.5 Hz, 4H), 1.55-1.46 (m, 4H), 0.92 (t, *J* = 7.3 Hz, 6H); **^{13}C NMR** (100 MHz, $CDCl_3$) δ 167.2, 140.8, 135.6, 132.5, 130.02, 129.4, 128.5, 124.2, 123.2, 120.6, 116.2, 108.1, 105.4, 88.6, 56.2, 53.1, 29.8, 20.2, 12.1; **HRMS** (FD^+) Calcd for $C_{25}H_{28}N_2O$ $[M]^+$ 372.2196, Found 372.2204; **FT-IR** (KBr) $\tilde{\nu}$ (cm^{-1}) 2183, 1719, 1617, 1588.

3-(3-phenylprop-2-yn-1-yl)indolin-2-one (8)

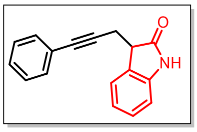

Light yellow solid; yield 58% (14.3 mg); m.p. 120-122 °C; R_f = 0.48 (hexanes/EA = 7:3); **^{1}H NMR** (400 MHz, $CDCl_3$) δ 8.23 (bs, 1H), 7.57 (d, *J* = 7.4 Hz, 1H), 7.35-7.32 (m, 2H), 7.31-7.28 (m, 4H), 7.08 (t, *J* = 7.5 Hz, 1H), 6.93 (d, *J* = 7.7 Hz, 1H), 3.70-3.67 (m, 1H), 3.23 (dd, *J* = 16.8, 4.4 Hz, 1H), 2.88-2.82 (m, 1H); **^{13}C NMR** (100 MHz, $CDCl_3$) δ 178.3, 141.5, 131.7, 128.8, 128.5, 128.3, 128.0, 124.9, 123.4, 122.6, 109.7, 86.1, 82.5, 45.0, 21.5; **HRMS** (FD^+) Calcd for $C_{17}H_{13}NO$ $[M]^+$ 247.0991, Found 247.0982; **FT-IR** (KBr) $\tilde{\nu}$ (cm^{-1}) 1701, 1616, 1486.

(*E*)-1-allyl-3-(3-phenylprop-2-yn-1-ylidene)indolin-2-one (9)

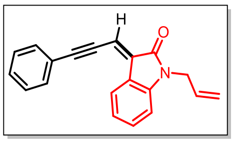

Light yellow solid; yield 70% (19.9 mg); m.p. 156-158 °C; R_f = 0.50 (hexanes/EA = 7:3); **^{1}H NMR** (400 MHz, $CDCl_3$) δ 8.15 (dd, *J* = 7.6, 1.2 Hz, 1H), 7.61-7.59 (m, 2H), 7.43-7.41 (m, 3H), 7.29 (td, *J* = 7.7, 1.2 Hz, 1H), 7.08 (td, *J* = 7.6, 1.0 Hz, 1H), 7.00 (s, 1H), 6.82 (d, J = 7.7 Hz, 1H), 5.90-5.81 (m, 1H), 5.26-5.20 (m, 2H), 4.38 (dt, *J* = 5.2, 1.7 Hz, 2H); **^{13}C NMR** (100 MHz, $CDCl_3$) δ 167.2, 143.3, 134.5, 132.1, 131.6, 130.4, 129.8, 128.8, 123.8, 122.5, 122.4, 122.0, 117.6, 113.7, 109.0, 105.2, 87.7, 42.4; **HRMS** (FD^+) Calcd for $C_{20}H_{15}NO$ $[M]^+$ 285.1148, Found 285.1155; **FT-IR** (KBr) $\tilde{\nu}$ (cm^{-1}) 2182, 1707, 1606, 1555.

3-(3-phenylpropyl)indolin-2-one (10)

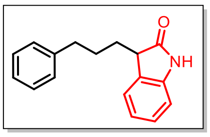

White solid; yield 68% (17.1 mg); m.p. 80-82 °C; R_f = 0.50 (hexanes/EA = 7:3); **^{1}H NMR** (400 MHz, $CDCl_3$) δ 9.14 (s, 1H), 7.28-7.22 (m, 2H), 7.21-7.13 (m, 5H), 7.02 (td, *J* = 7.5, 1.0 Hz, 1H), 6.91 (dd, *J* = 7.6, 1.3 Hz, 1H), 3.50 (t, *J* = 6.0 Hz, 1H), 2.70-2.58 (m, 2H), 2.09-1.96 (m, 2H), 1.82-1.62 (m, 2H); **^{13}C NMR** (100 MHz, $CDCl_3$) δ 180.9, 141.9, 141.8, 129.7, 128.4, 128.4, 127.9, 125.9, 124.1, 122.3, 109.9, 46.1, 35.9, 30.2, 27.6; **HRMS** (FD^+) Calcd for $C_{17}H_{17}NO$ $[M]^+$ 251.1304, Found 251.1308; **FT-IR** (KBr) $\tilde{\nu}$ (cm^{-1}) 2927, 2858, 1706, 1602, 1298, 1267.

(*E*)-2-oxo-*N*-phenyl-3-(3-phenylprop-2-yn-1-ylidene)indoline-1- carboxamide (11a)

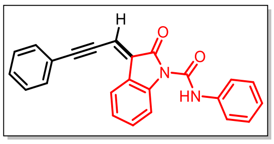

Yellow solid; yield 70% (25.5 mg); m.p. 154-156 °C; R_f = 0.38 (hexanes/EA = 7:3); **^{1}H NMR** (400 MHz, $CDCl_3$) δ 10.75 (s, 1H), 8.38 (dt, J = 8.2, 0.8 Hz, 1H), 8.27 (dd, J = 7.6, 1.3 Hz, 1H), 7.64-7.60 (m, 4H), 7.47-7.40 (m, 4H), 7.39-7.35 (m, 2H), 7.28-7.24 (m, 1H), 7.17-7.13 (m, 1H), 7.11 (s, 1H); **^{13}C NMR** (100 MHz, $CDCl_3$) δ 169.3, 149.5, 140.3, 137.3, 133.1, 132.3, 131.2, 130.3, 129.2, 128.9, 124.8, 124.6, 123.2, 122.1, 121.9, 120.6, 116.6, 116.1, 108.6, 87.8; **HRMS** (FD^+) Calcd for $C_{24}H_{16}N_2O_2$ $[M]^+$ 364.1206, Found 364.1201; **FT-IR** (KBr) $\tilde{\nu}$ (cm^{-1}) 2181, 1724, 1608, 1593.

(*E*)-2-oxo-3-(3-phenylprop-2-yn-1-ylidene)-*N*-(4-(trifluoromethyl)phenyl) indoline-1-carboxamide (11b)

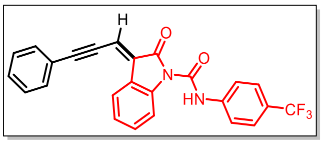

Yellow solid; yield 68% (29.4 mg); m.p. 165-167 °C; R_f = 0.42 (hexanes/EA = 7:3); **^{1}H NMR** (400 MHz, $CDCl_3$) δ 10.96 (s, 1H), 8.31 (d, J = 8.2 Hz, 1H), 8.23 (dd, J = 7.7, 1.2 Hz, 1H), 7.70 (d, J = 8.4 Hz, 2H), 7.60-7.57 (m, 4H), 7.44-7.37 (m, 4H), 7.24-7.22 (m, 1H), 7.08 (s, 1H); **^{13}C NMR** (100 MHz, $CDCl_3$) δ 169.4, 149.3, 140.5, 139.9, 132.8, 132.3, 131.2, 130.4, 128.9, 126.4, 126.4, 125.0, 123.2, 122.0, 121.9, 120.0, 116.6, 109.1, 87.7; **HRMS** (FD^+) Calcd for $C_{25}H_{15}F_3N_2O_2$ $[M]^+$ 432.1080, Found 432.1083; **FT-IR** (KBr) $\tilde{\nu}$ (cm^{-1}) 2183, 1721, 1599, 1555.

(*E*)-*N*-(2-fluorophenyl)-2-oxo-3-(3-phenylprop-2-yn-1-ylidene)indoline-1-carboxamide (11c)

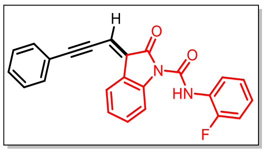

Yellow solid; yield 60% (22.9 mg); m.p. 170-172 °C; R_f = 0.46 (hexanes/EA = 7:3); **^{1}H NMR** (400 MHz, $CDCl_3$) δ 11.03 (s, 1H), 8.36 (d, J = 8.2 Hz, 1H), 8.31-8.26 (m, 2H), 7.63-7.61 (m, 2H), 7.47-7.40 (m, 4H), 7.28-7.24 (m, 1H), 7.19-7.14 (m, 2H), 7.13-7.12 (m, 1H), 7.11-7.06 (m, 1H); **^{13}C NMR** (100 MHz, $CDCl_3$) δ 169.3, 153.3 (d, $^1J_{FC}$ = 243.8 Hz), 149.2, 140.1, 132.9, 132.3, 131.2, 130.3, 128.9, 126.0 (d, $^3J_{FC}$ = 10.2 Hz), 124.9, 124.6 (d, $^4J_{FC}$ = 3.1 Hz), 124.6, 123.2, 122.1, 122.0, 116.4 (d, $^3J_{FC}$ = 16.2 Hz), 115.2 (d, $^2J_{FC}$ = 18.9 Hz), 108.7, 87.8; **HRMS** (FD^+) Calcd for $C_{24}H_{15}FN_2O_2$ $[M]^+$ 382.1112, Found 382.1108; **FT-IR** (KBr) $\tilde{\nu}$ (cm^{-1}) 2179, 1701, 1620, 1594.

(*E*)-2-oxo-3-(3-phenylprop-2-yn-1-ylidene)-*N*-(3-(trifluoromethyl)phenyl) indoline-1-carboxamide (11d)

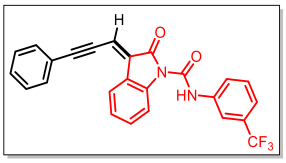

Yellow solid; yield 60% (25.9 mg); m.p. 168-170 °C; R_f = 0.50 (hexanes/EA = 7:3); **^{1}H NMR** (400 MHz, $CDCl_3$) δ 10.96 (s, 1H), 8.36 (dt, J = 8.2, 0.8 Hz, 1H), 8.28 (dd, J = 7.6, 1.2 Hz, 1H), 7.97 (d, J = 1.9 Hz, 1H), 7.76 (dt, J = 8.0, 1.4 Hz, 1H), 7.64-7.62 (m, 2H), 7.50-7.48 (m, 1H), 7.47-7.41 (m, 4H), 7.41-7.38 (m, 1H), 7.28 (dd, J = 7.6, 1.0 Hz, 1H), 7.12 (s, 1H); **^{13}C NMR** (100 MHz, $CDCl_3$) δ 169.5, 149.4, 140.0, 138.0, 132.9, 132.3, 131.8, 131.5, 131.3, 130.4, 129.7, 128.9, 125.0, 124.1 (q, $^1J_{FC}$ = 270.9 Hz), 123.5, 123.3, 122.0, 121.9, 121.0 (q, $^3J_{FC}$ = 3.61 Hz), 117.2 (q, $^3J_{FC}$ = 3.74 Hz), 116.6, 116.5, 109.0, 87.7; **HRMS** (FD^+) Calcd for $C_{25}H_{15}F_3N_2O_2$ $[M]^+$ 432.1080, Found 432.1078; **FT-IR** (KBr) $\tilde{\nu}$ (cm^{-1}) 2180, 1700, 1609, 1573.

5-phenylbenzo[cd]indol-2(1*H*)-one (12)

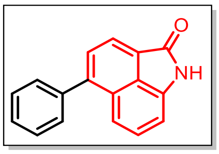

Yellow solid; yield 46% (11.3 mg); m.p. 179-181 °C; R_f = 0.40 (hexanes/EA = 7:3); **^{1}H NMR** (400 MHz, $CDCl_3$) δ 8.31 (s, 1H), 7.51-7.43 (m, 5H), 7.22-7.21 (m, 1H), 7.19-7.17 (m, 1H), 7.13 (d, J = 12.2 Hz, 1H), 7.02-6.98 (m, 1H), 6.81 (d, J = 7.8 Hz, 1H); **^{13}C NMR** (100 MHz, $CDCl_3$) δ 169.2, 155.5, 141.6, 131.7, 130.8, 130.6, 130.4, 129.3, 129.2, 127.6, 124.1, 122.7, 122.0, 120.2, 117.4, 110.5; **HRMS** (FD^+) Calcd for $C_{17}H_{11}NO$ $[M]^+$ 245.0835, Found 245.0840; **FT-IR** (KBr) $\tilde{\nu}$ (cm^{-1}) 1701, 1605, 1490.

(*E*)-1-(4-methoxyphenyl)-3-(3-phenylprop-2-yn-1-ylidene)indolin-2-one (13a)

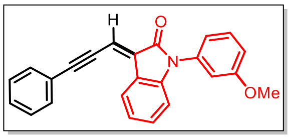

Light yellow solid; yield 52% (18.2 mg); m.p. 160-162 °C; R_f = 0.42 (hexanes/EA = 7:3); **^{1}H NMR** (400 MHz, $CDCl_3$) δ 8.41 (d, J = 7.5 Hz, 1H), 7.82-7.79 (m, 2H), 7.62-7.61 (m, 4H), 7.46-7.42 (m, 2H), 7.31 (t, J = 7.5 Hz, 1H), 7.21 (dd, J = 7.7, 1.9 Hz, 1H), 7.17-7.13 (m, 2H), 7.05 (d, J = 7.8 Hz, 1H), 4.02 (s, 3H); **^{13}C NMR** (100 MHz, $CDCl_3$) δ 166.8, 160.6, 143.9, 135.5, 134.4, 132.1, 130.4, 130.3, 129.8, 128.8, 123.9, 122.9, 122.4, 121.9, 118.7, 114.2, 114.1, 112.2, 109.6, 105.7, 87.8, 55.5; **HRMS** (FD^+) Calcd for $C_{24}H_{17}NO_2$ $[M]^+$ 351.1253, Found 351.1249; **FT-IR** (KBr) $\tilde{\nu}$ (cm^{-1}) 2185, 1713, 1604, 1495.

(*E*)-1-(4-nitrophenyl)-3-(3-phenylprop-2-yn-1-ylidene)indolin-2-one (13b)

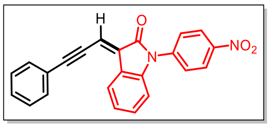

Yellow solid; yield 50% (18.3 mg); m.p. 156-158 °C; R_f = 0.40 (hexanes/EA = 7:3); **^{1}H NMR** (400 MHz, $CDCl_3$) δ 8.42-8.38 (m, 2H), 8.29 (dd, J = 7.6, 1.2 Hz, 1H), 7.71 (d, J = 8.9 Hz, 2H), 7.64-7.62 (m, 2H), 7.47-7.43 (m, 3H), 7.33 (td, J = 7.8, 1.3 Hz, 1H), 7.21 (t, J = 7.5 Hz, 1H), 7.11 (s, 1H), 7.00 (d, J = 7.9 Hz, 1H); **^{13}C NMR** (100 MHz, $CDCl_3$) δ 166.5, 146.4, 142.1, 140.4, 133.4,

132.2, 130.5, 130.1, 128.9, 126.4, 125.1, 124.3, 123.9, 122.3, 122.2, 115.4, 109.5, 107.0, 87.7; **HRMS** (FD$^+$) Calcd for $C_{23}H_{14}N_2O_3$ [M]$^+$ 366.0998, Found 366.0995; **FT-IR** (KBr) $\tilde{\nu}$ (cm^{-1}) 2183, 1716, 1610, 1592.

(*E*)-1-phenyl-3-(3-phenylprop-2-yn-1-ylidene)indolin-2-one (14a)

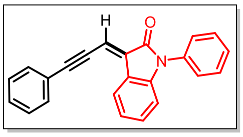

Yellow solid; yield 65% (20.8 mg); m.p. 158-160 °C; R_f = 0.48 (hexanes/EA = 7:3); **^{1}H NMR** (400 MHz, $CDCl_3$) δ 8.24 (dd, *J* = 7.6, 1.2 Hz, 1H), 7.64-7.62 (m, 2H), 7.55-7.51 (m, 2H), 7.46-7.39 (m, 6H), 7.28-7.24 (m, 1H), 7.13 (td, *J* = 7.6, 1.0 Hz, 1H), 7.08 (s, 1H), 6.85 (dt, *J* = 7.8, 0.8 Hz, 1H); **^{13}C NMR** (100 MHz, $CDCl_3$) δ 166.9, 143.9, 134.5, 134.4, 132.2, 130.5, 129.9, 129.7, 128.8, 128.1, 126.7, 123.9, 122.9, 122.5, 122.0, 114.3, 109.5, 105.8, 87.9; **HRMS** (FD$^+$) Calcd for $C_{23}H_{15}NO$ [M]$^+$ 321.1148, Found 321.1144; **FT-IR** (KBr) $\tilde{\nu}$ (cm^{-1}) 2186, 1740, 1607, 1493.

(*E*)-1-(3-methoxyphenyl)-3-(3-phenylprop-2-yn-1-ylidene)indolin-2-one (14b)

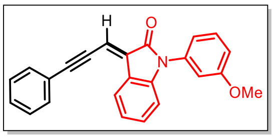

Yellow solid; yield 67% (23.5 mg); m.p. 160-162 °C; R_f = 0.50 (hexanes/EA = 7:3); **^{1}H NMR** (400 MHz, $CDCl_3$) δ 8.08 (dd, *J* = 7.6, 1.2 Hz, 1H), 7.49-7.46 (m, 2H), 7.30-7.27 (m, 4H), 7.13-7.09 (m, 1H), 6.97 (td, *J* = 7.6, 1.0 Hz, 1H), 6.92 (s, 1H), 6.89-6.86 (m, 1H), 6.84-6.79 (m, 2H), 6.72 (d, *J* = 7.9 Hz, 1H), 3.69 (s, 3H); **^{13}C NMR** (100 MHz, $CDCl_3$) δ 166.8, 160.7, 143.9, 135.5, 134.4, 132.2, 130.5, 130.4, 129.9, 128.8, 123.9, 122.9, 122.5, 122.0, 118.8, 114.3, 114.2, 112.3, 109.7, 105.8, 87.9, 55.60; **HRMS** (FD$^+$) Calcd for $C_{24}H_{17}NO_2$ [M]$^+$ 351.1253, Found 351.1243; **FT-IR** (KBr) $\tilde{\nu}$ (cm^{-1}) 2086, 1742, 1605, 1495.

(*E*)-1-(2,3-dimethylphenyl)-3-(3-phenylprop-2-yn-1-ylidene)indolin-2-one (14c)

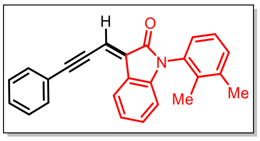

Yellow solid; yield 64% (22.3 mg); m.p. 148-150 °C; R_f = 0.54 (hexanes/EA = 7:3); **^{1}H NMR** (400 MHz, $CDCl_3$) δ 8.23 (d, *J* = 7.5 Hz, 1H), 7.65-7.62 (m, 2H), 7.45-7.44 (m, 3H), 7.28-7.26 (m, 1H), 7.24-7.21 (m, 2H), 7.13-7.09 (m, 2H), 7.08 (s, 1H), 6.47 (d, *J* = 7.8 Hz, 1H), 2.37 (s, 3H), 2.07 (s, 3H); **^{13}C NMR** (100 MHz, $CDCl_3$) δ 166.9, 144.6, 138.9, 135.4, 134.7, 133.2, 132.2, 130.7, 130.6, 129.8, 128.8, 126.79, 125.9, 123.9, 122.7, 122.6, 121.9, 114.3, 109.6, 105.5, 87.9, 20.6, 14.6; **HRMS** (FD$^+$) Calcd for $C_{25}H_{19}NO$ [M]$^+$ 349.1461, Found 349.1455; **FT-IR** (KBr) $\tilde{\nu}$ (cm^{-1}) 2185, 1736, 1607, 1491.

(*E*)-1-(3-chlorophenyl)-3-(3-phenylprop-2-yn-1-ylidene)indolin-2-one (14d)

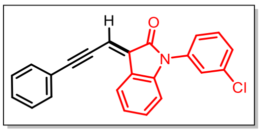

Yellow solid; yield 62% (22.0 mg); m.p. 150-152 °C; R_f = 0.50 (hexanes/EA = 7:3); **^{1}H NMR** (400 MHz, $CDCl_3$) δ 8.24 (d, *J* = 7.5 Hz,

1H), 7.64-7.61 (m, 2H), 7.48-7.42 (m, 5H), 7.40-7.35 (m, 2H), 7.28 (t, J = 7.7 Hz, 1H), 7.15 (t, J = 7.5 Hz, 1H), 7.08 (s, 1H), 6.87 (d, J = 7.9 Hz, 1H); **^{13}C NMR** (100 MHz, $CDCl_3$) δ 166.7, 143.3, 135.7, 135.2, 134.0, 132.2, 130.7, 130.6, 129.9, 128.9, 128.3, 126.9, 124.8, 124.1, 123.3, 122.4, 122.1, 114.8, 109.5, 106.2, 87.8; **HRMS** (FD^+) Calcd for $C_{23}H_{14}ClNO$ $[M]^+$ 355.0758, Found 355.0756; **FT-IR** (KBr) $\tilde{\nu}$ (cm^{-1}) 2184, 1736, 1608, 1590.

(*E*)-3-(3-phenylprop-2-yn-1-ylidene)-5-(*p*-tolyl)indolin-2-one (15)

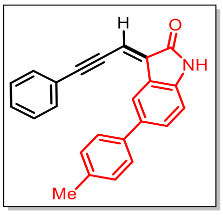

Light yellow solid; yield 65% (21.8 mg); m.p. 198-200 °C; R_f = 0.52 (hexanes/EA = 7:3); **^{1}H NMR** (400 MHz, DMSO-d_6) δ 10.70 (s, 1H), 8.30 (s, 1H), 7.63 (d, J = 6.8 Hz, 2H), 7.57 (d, J = 8.3 Hz, 1H), 7.54-7.48 (m, 5H), 7.24 (d, J = 7.6 Hz, 2H), 6.95 (d, J = 8.1 Hz, 1H), 6.86 (bs, 1H), 2.33 (s, 3H); **^{13}C NMR** (100 MHz, DMSO-d_6) δ 167.5, 142.1, 137.2, 136.4, 135.9, 133.9, 131.7, 130.2, 129.5, 129.2, 129.1, 126.0, 122.3, 121.4, 121.4, 111.9, 110.4, 104.2, 87.8, 20.6; **HRMS** (FD^+) Calcd for $C_{24}H_{17}NO$ $[M]^+$ 335.1304, Found 335.1303; **FT-IR** (KBr) $\tilde{\nu}$ (cm^{-1}) 2184, 1707, 1608, 1591.

Methyl (*E*)-3-(4-nitrophenyl)acrylate (4a'')

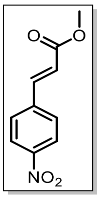

White solid; yield 65% (13.4 mg); m.p. 160-162 °C; R_f = 0.50 (hexanes/EA = 7:3); **^{1}H NMR** (400 MHz, DMSO-d_6) δ 8.24 (d, J = 8.8 Hz, 2H), 7.73 (s, 1H), 7.69-7.65 (m, 2H), 6.55 (d, J = 16.0 Hz, 1H), 3.83 (bs, 3H); **^{13}C NMR** (100 MHz, DMSO-d_6) δ 166.6, 148.6, 142.0, 140.6, 128.7, 124.3, 122.2, 52.2.

Methyl 2-(triphenyl-λ^5-phosphaneylidene)acetate (4')

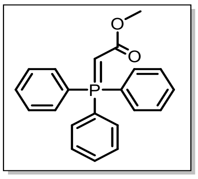

^{1}H NMR (400 MHz, $CDCl_3$) δ 7.68-7.64 (m, 6H), 7.55-7.53 (m, 3H), 7.47-7.44 (m, 7H), 3.59 (s, 3H); **^{31}P NMR** (242.5 Hz, $CDCl_3$) 22.5, 18.59 ppm.

Methyl 2-(tri-*p*-tolyl-λ^5-phosphaneylidene)acetate (5')

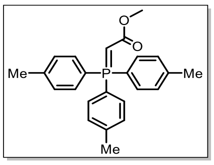

^{1}H NMR (400 MHz, $CDCl_3$) δ 7.53-7.52 (m, 6H), 7.51-7.50 (m, 7H), 3.56 (s, 3H), 2.41 (s, 9H); **^{31}P NMR** (242.5 Hz, $CDCl_3$) 17.40, 15.54 ppm.

Methyl 2-(tris(4-methoxyphenyl)-λ^5-phosphaneylidene)acetate (6')

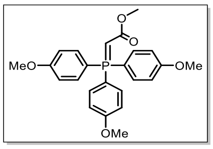

^{1}H NMR (400 MHz, $CDCl_3$) δ 7.59-7.54 (m, 6H), 6.94-6.92 (m, 7H), 3.82 (s, 9H), 3.57 (s, 3H); **^{31}P NMR** (242.5 Hz, $CDCl_3$) 16.10, 14.42 ppm.

^{1}H and ^{13}C NMR Spectra of Compounds

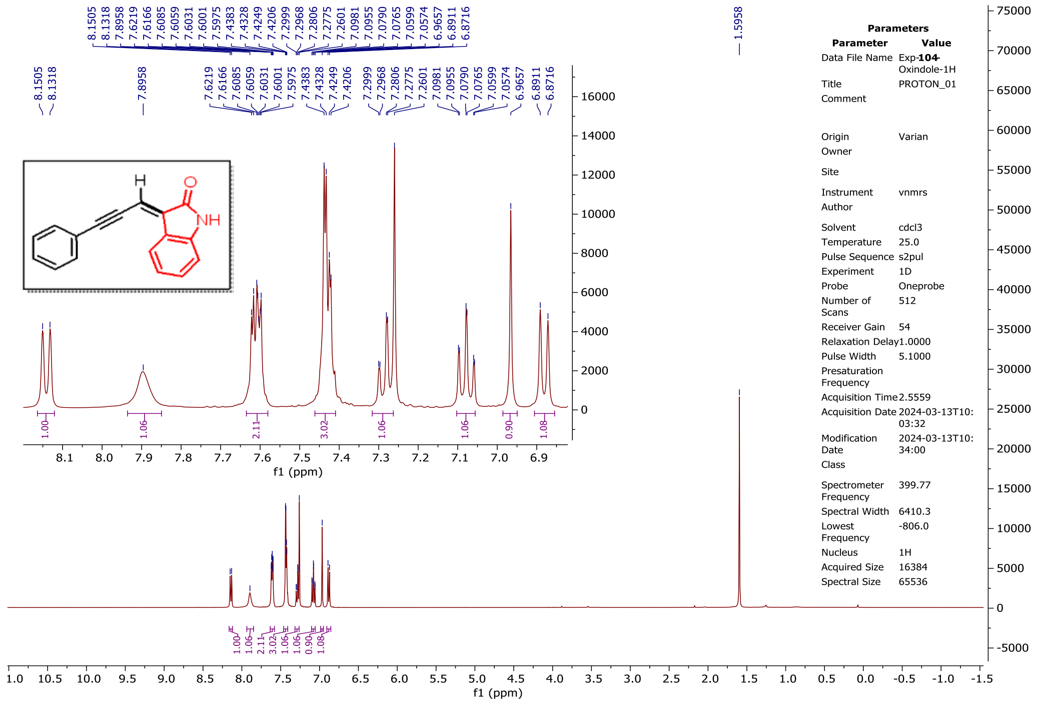

Figure S1. ^{1}H NMR (400 MHz, $CDCl_3$) spectra of compound (4a)

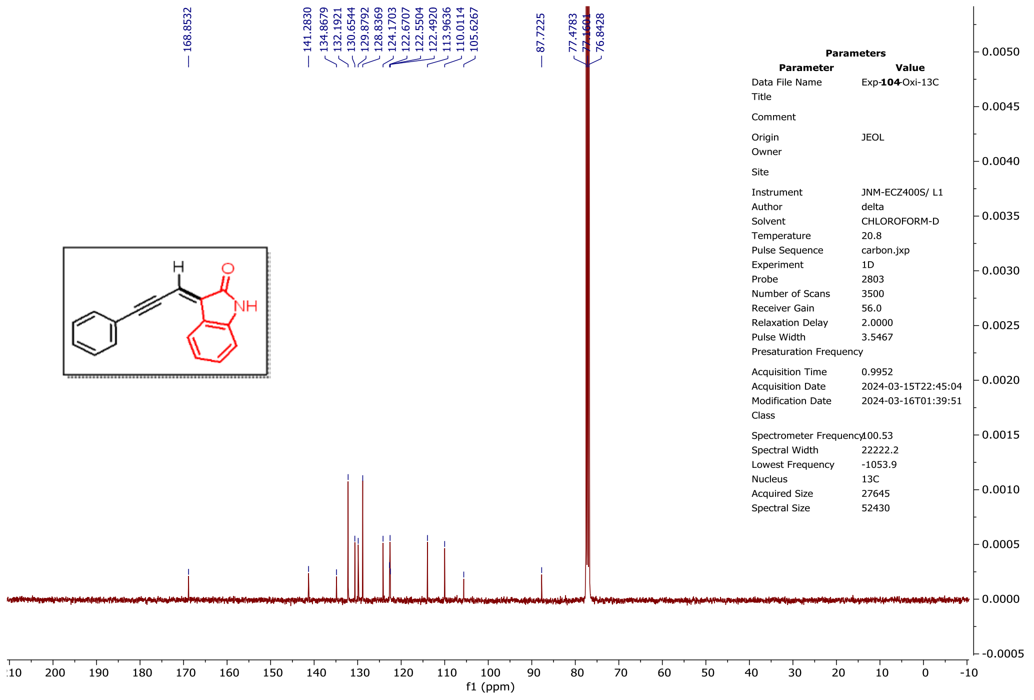

Figure S2. ^{13}C NMR (100 MHz, $CDCl_3$) spectra of compound (4a)

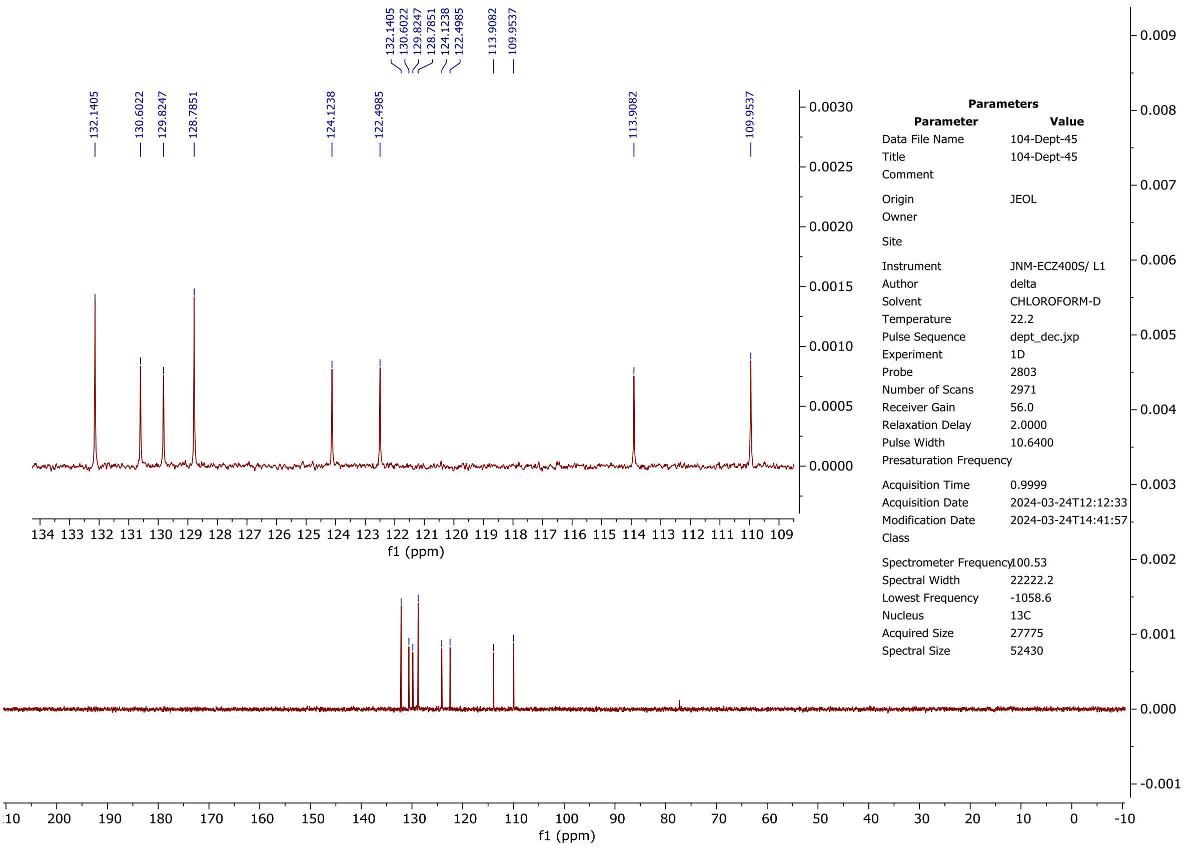

Figure S3. DEPT 45 NMR (100 MHz, $CDCl_3$) spectra of compound (4a)

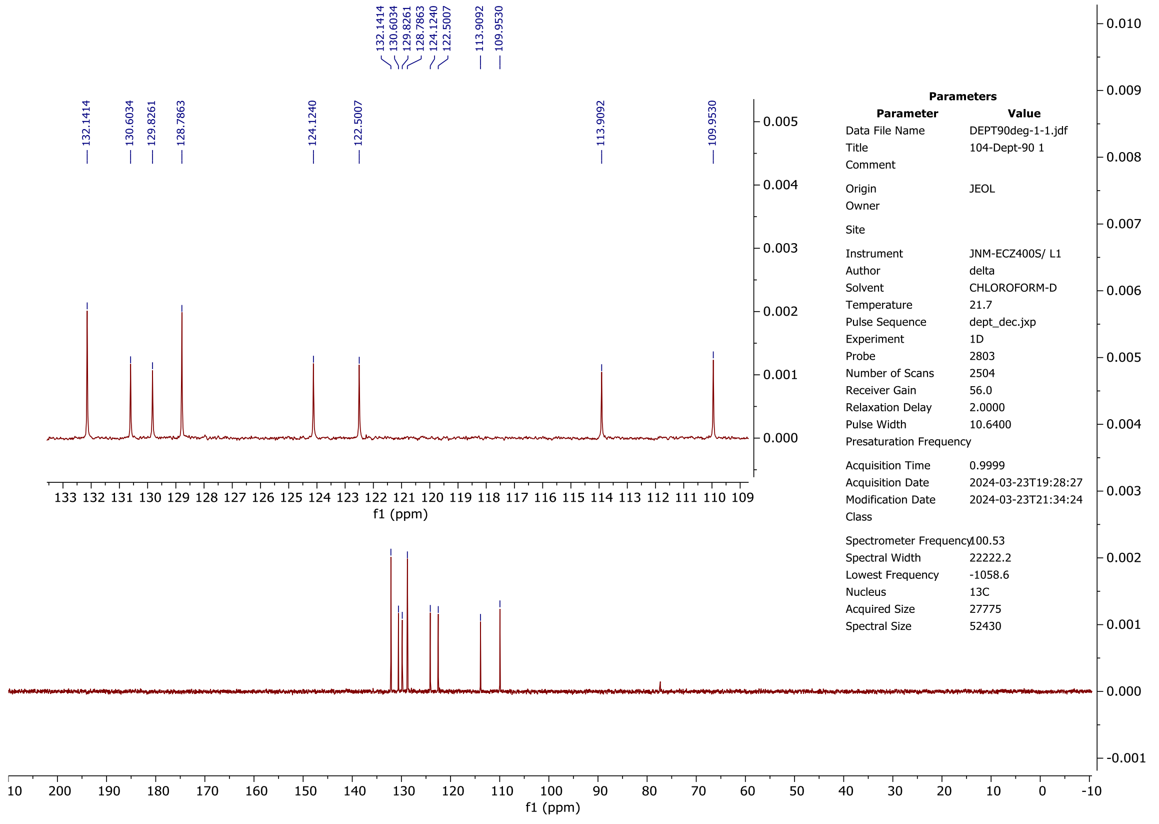

Figure S4. DEPT 90 NMR (100 MHz, $CDCl_3$) spectra of compound (4a)

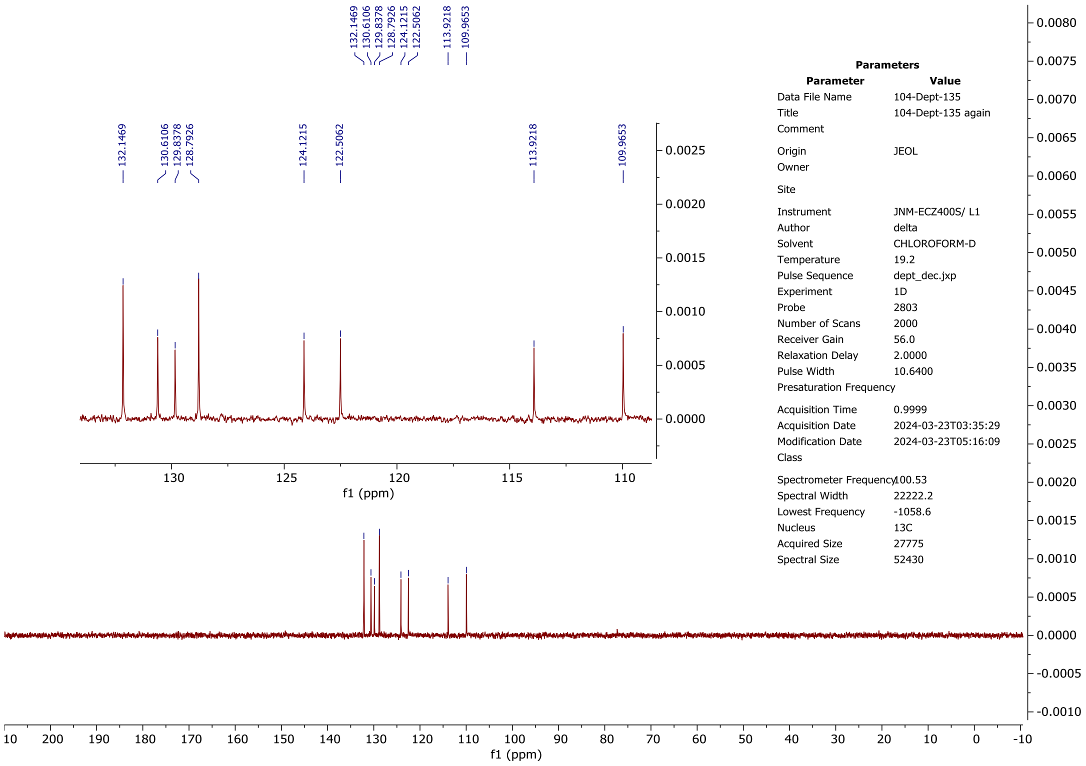

Figure S5. DEPT 135 NMR (100 MHz, $CDCl_3$) spectra of compound (4a)

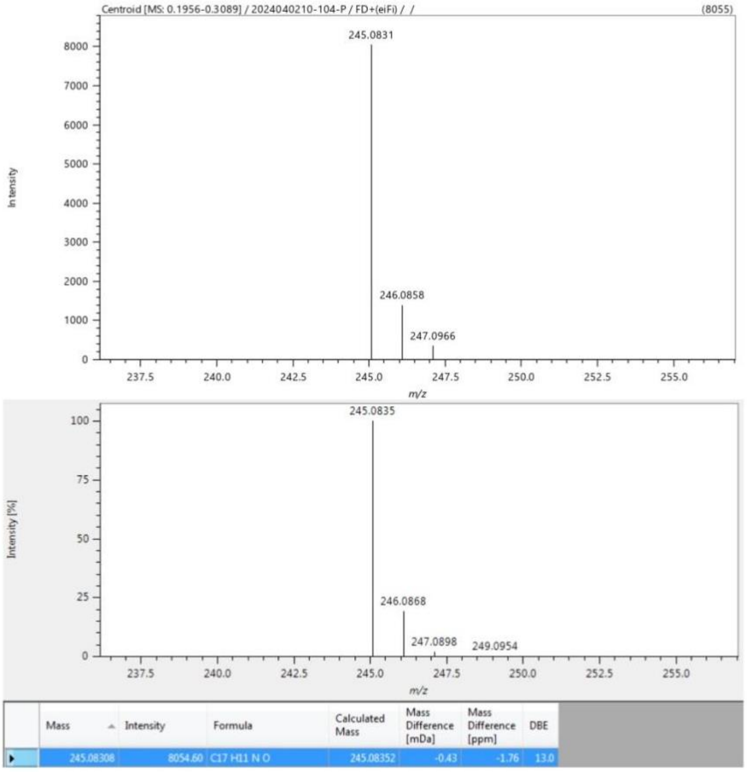

Figure S6: HRMS of compound (4a)

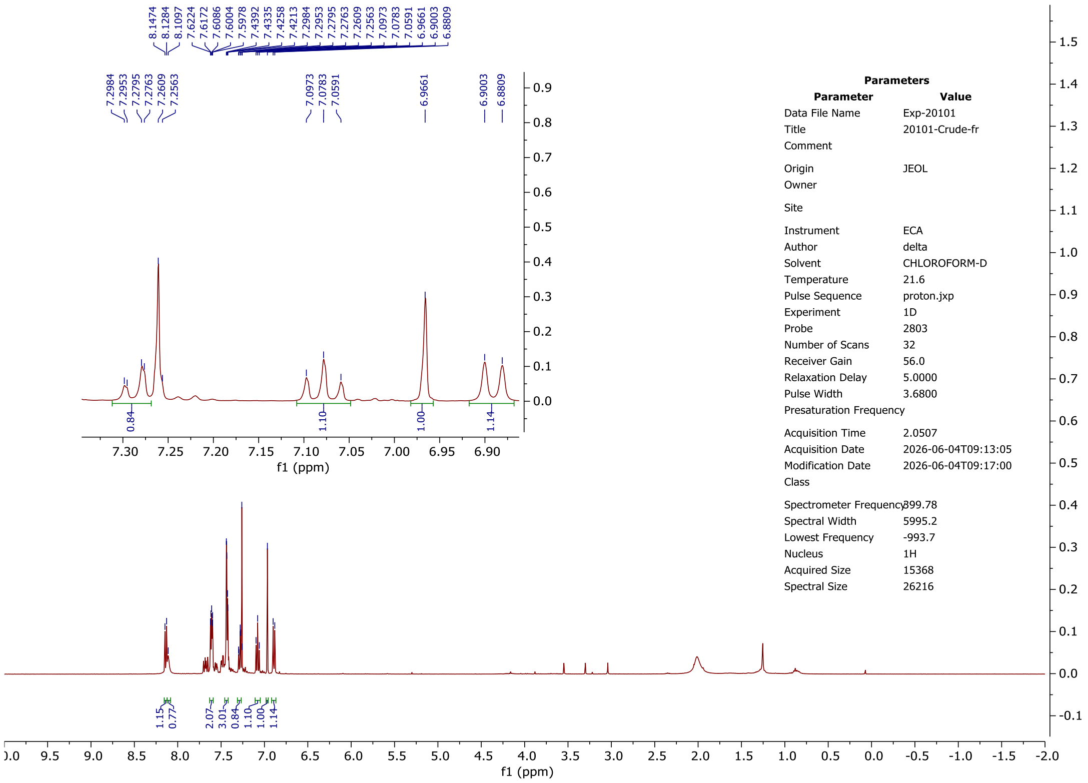

Figure S7. ^{1}H NMR (400 MHz, $CDCl_3$) spectra of crude (4a)

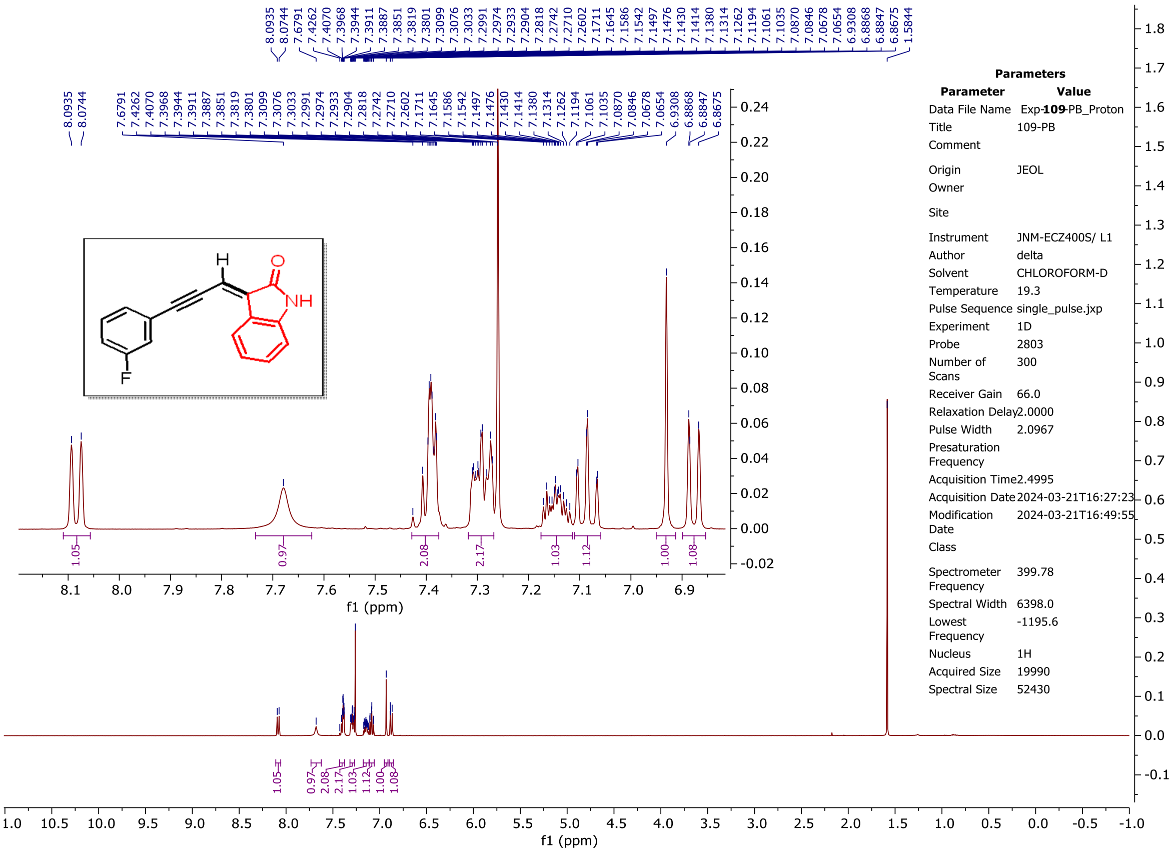

Figure S8. ^{1}H NMR (400 MHz, $CDCl_3$) spectra of compound (4b)

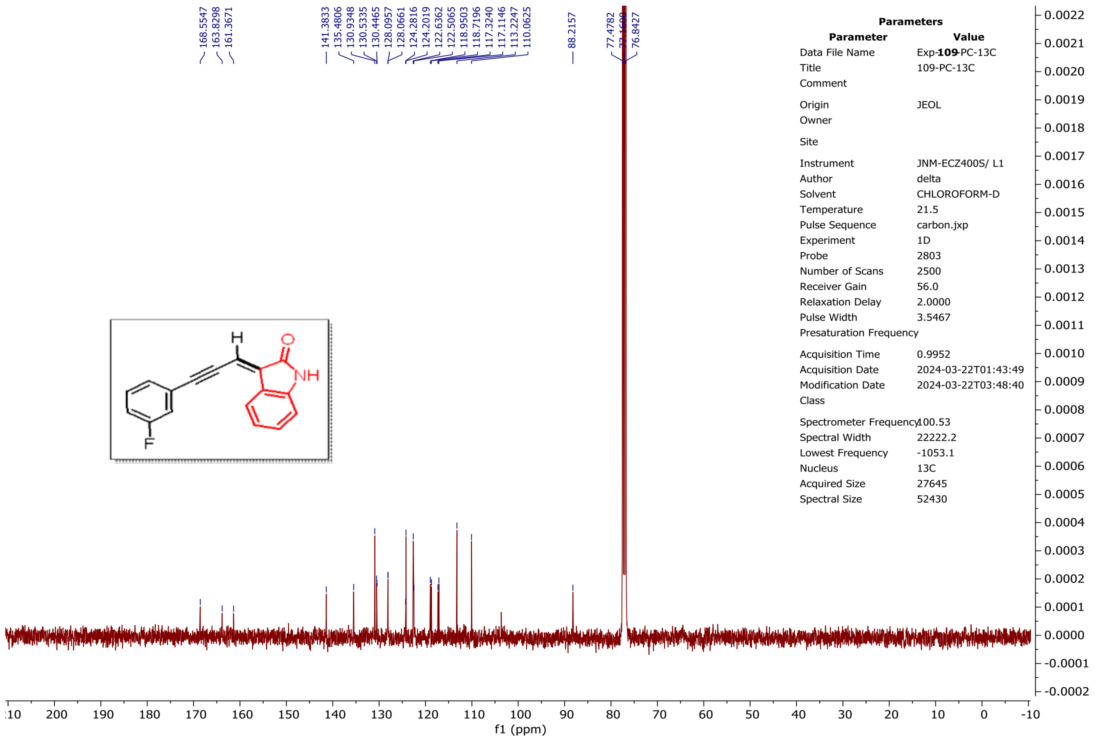

Figure S9. ^{13}C **NMR (100 MHz,** $CDCl_3$**) spectra of compound (4b)**

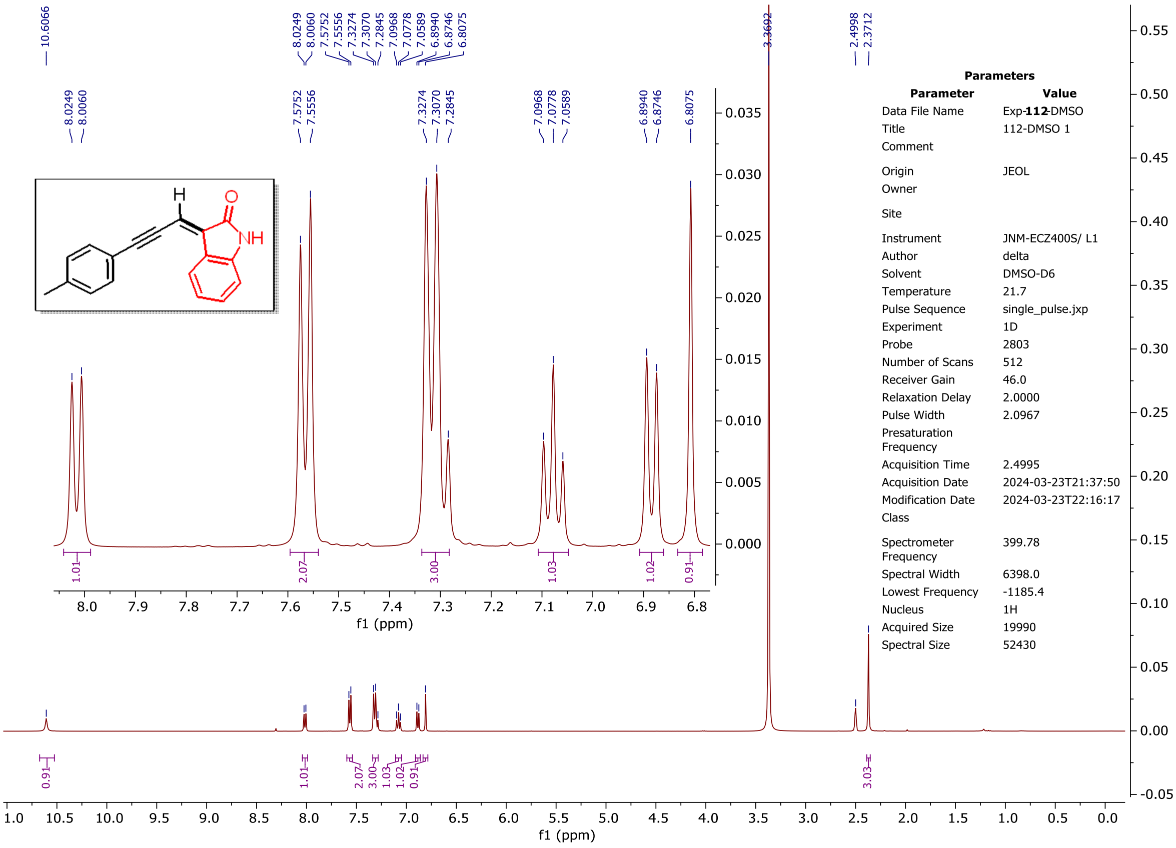

Figure S10. ^{1}H **NMR (100 MHz, DMSO-**d_6**) spectra of compound (4c)**

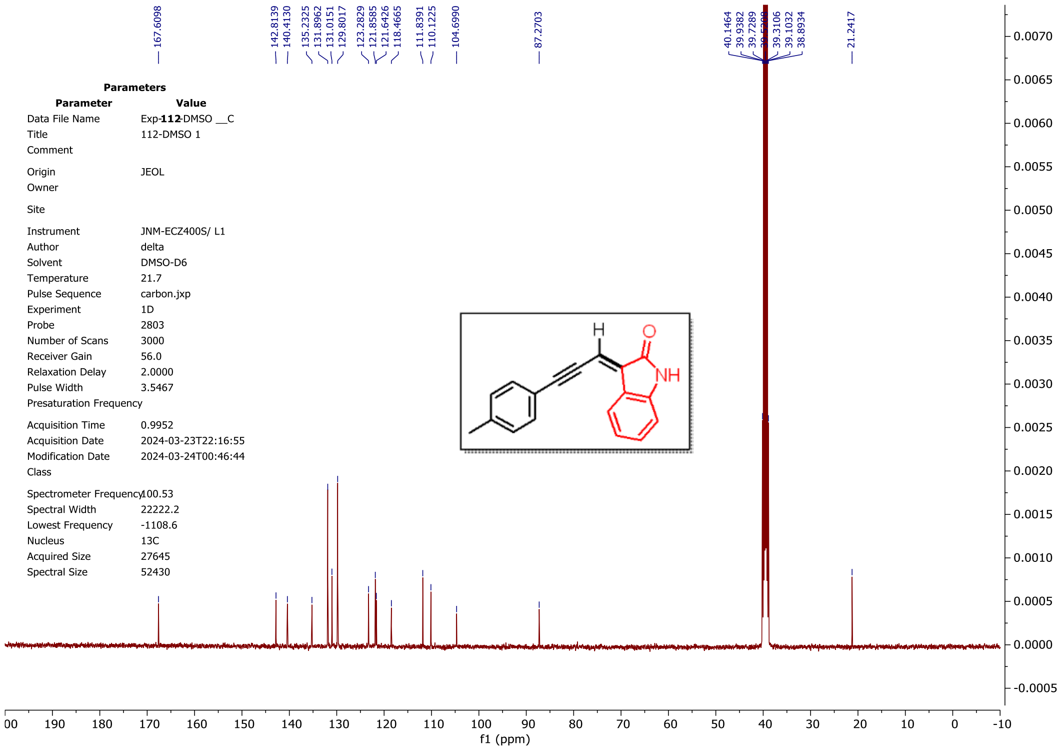

Figure S11. ^{13}C **NMR (100 MHz, DMSO-*d*$_6$) spectra of compound (4c)**

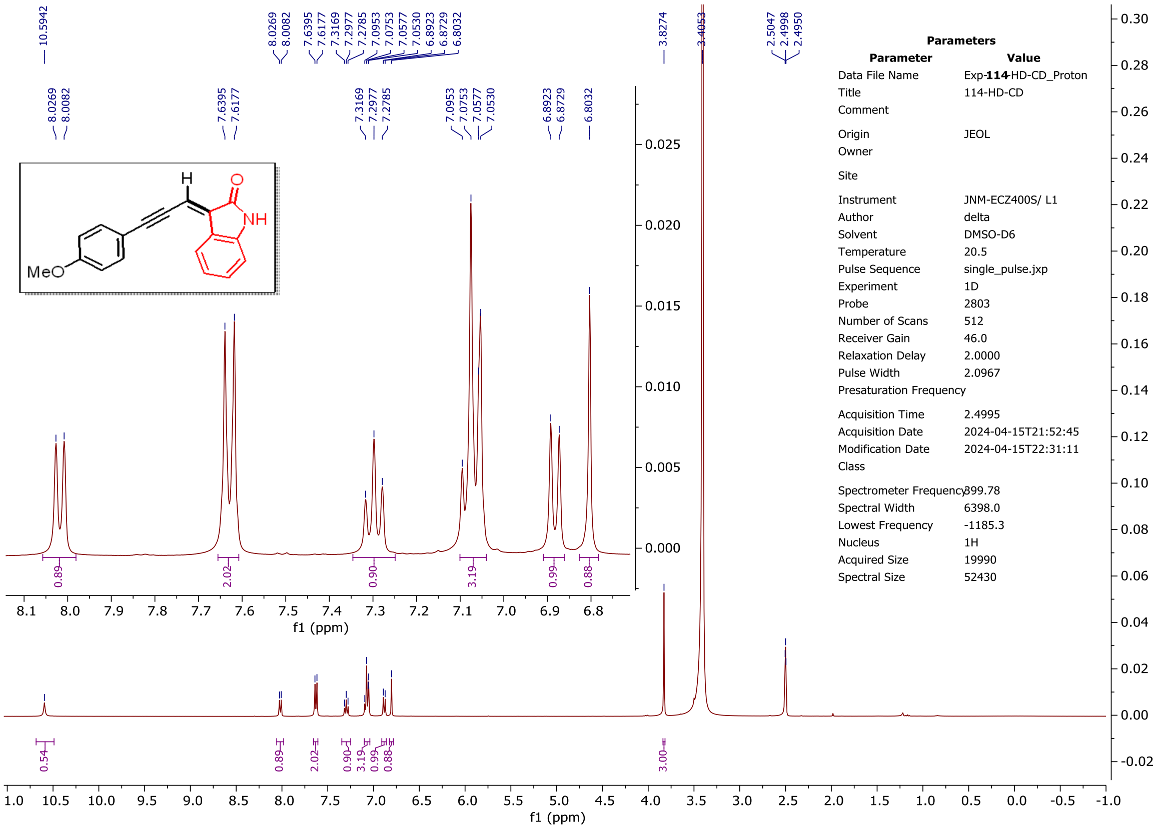

Figure S12. ^{1}H **NMR (400 MHz, DMSO-*d*$_6$) spectra of compound (4d)**

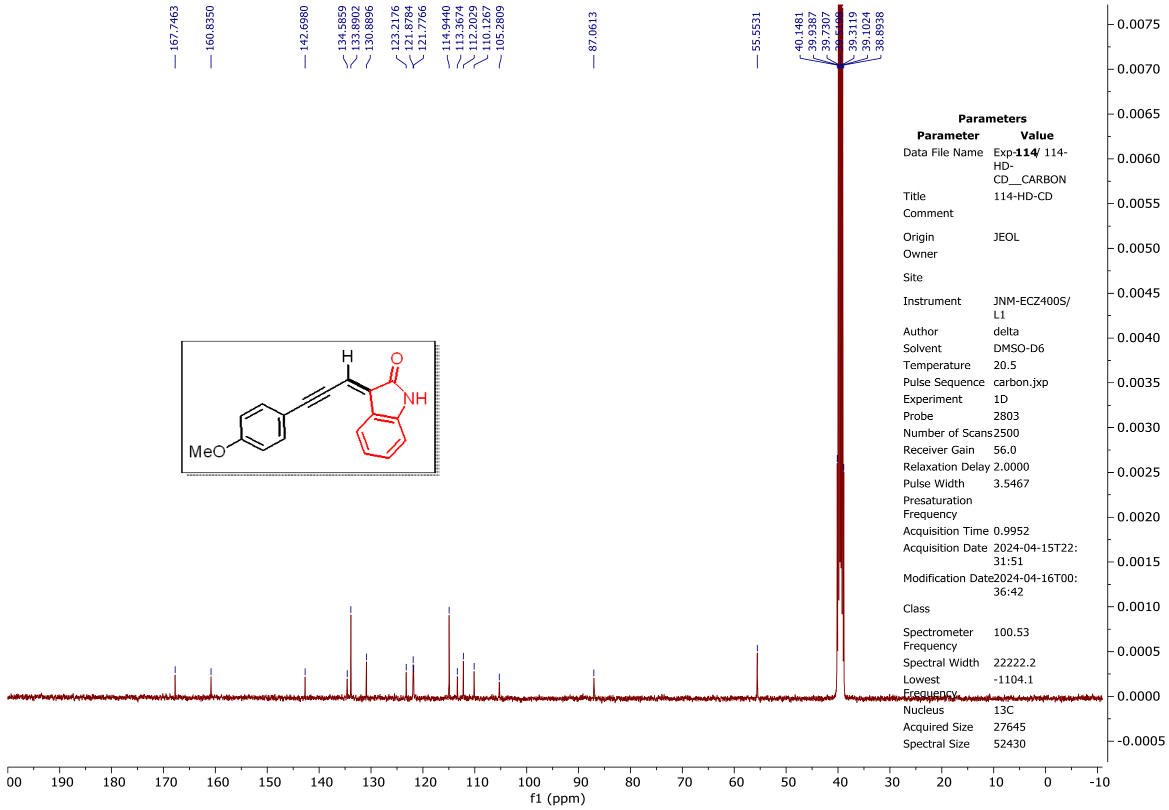

Figure S13. ^{13}C NMR (100 MHz, DMSO-d_6) spectra of compound (4d)

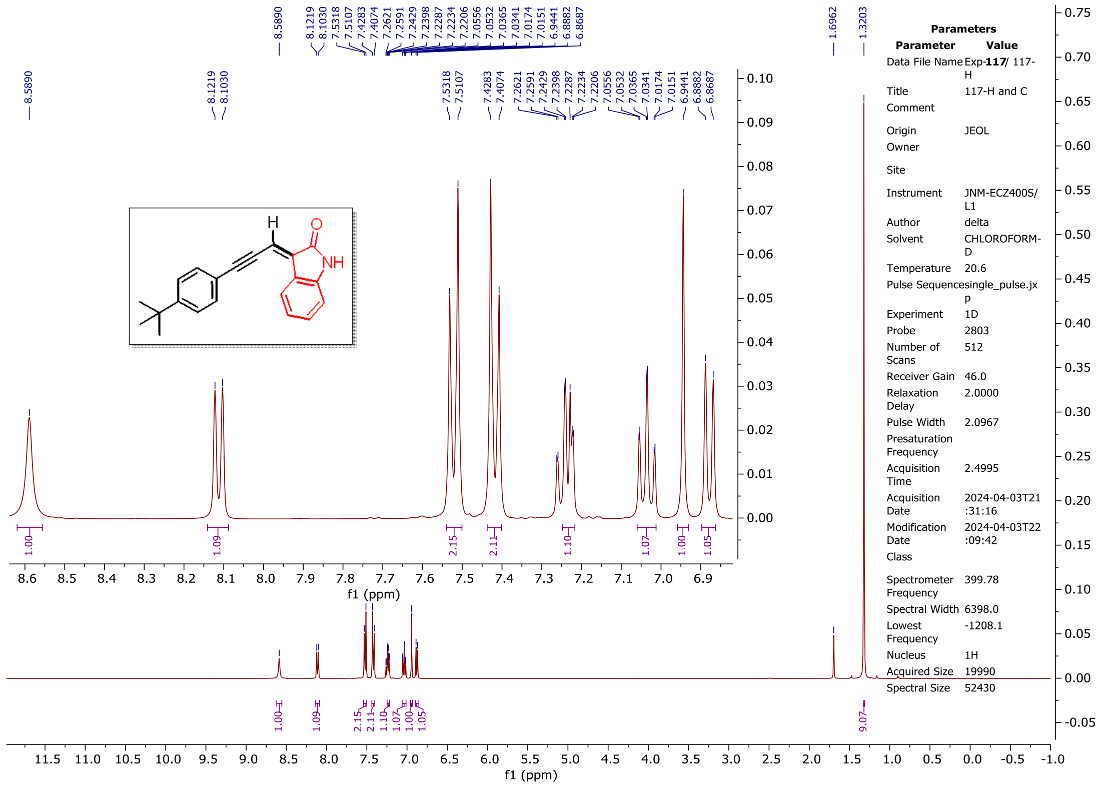

Figure S14. ^{1}H NMR (400 MHz, $CDCl_3$) spectra of compound (4e)

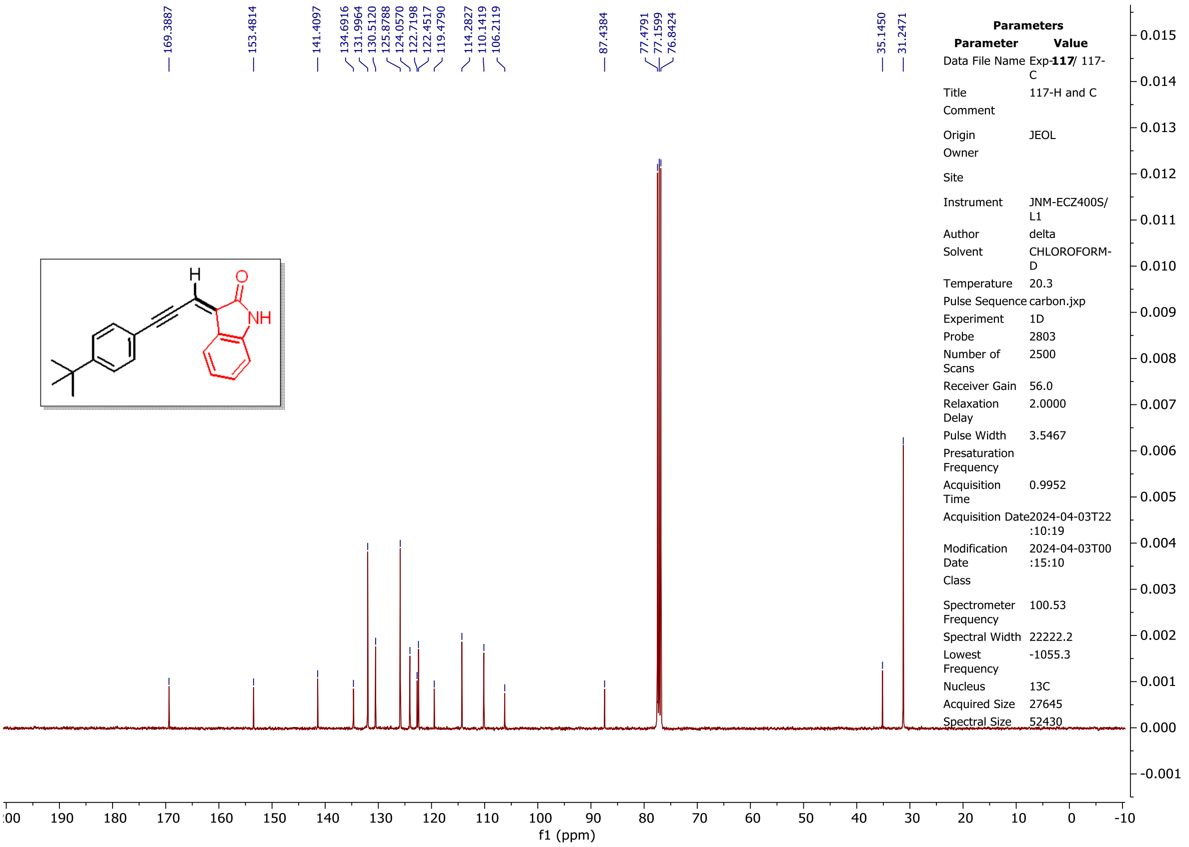

Figure S15. ^{13}**C NMR (100 MHz, $CDCl_3$) spectra of compound (4e)**

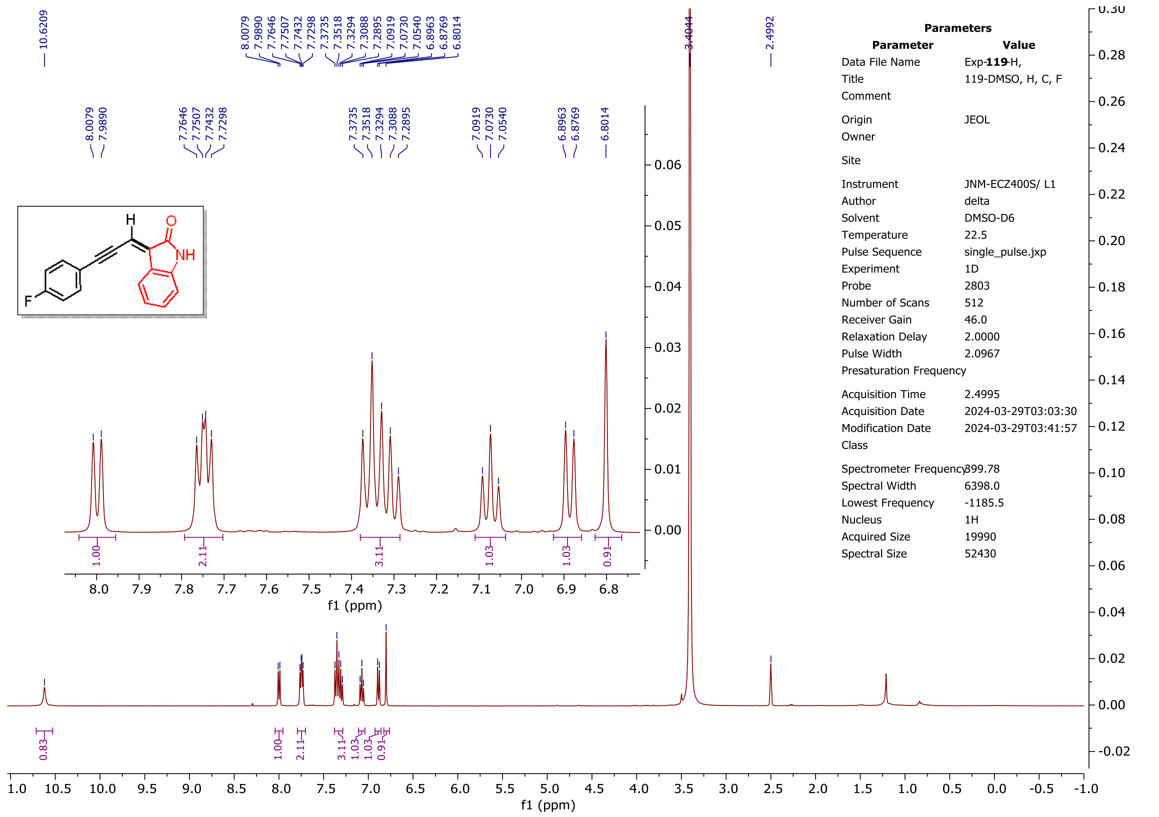

Figure S16. ^{1}**H NMR (400 MHz, DMSO-d_6) spectra of compound (4f)**

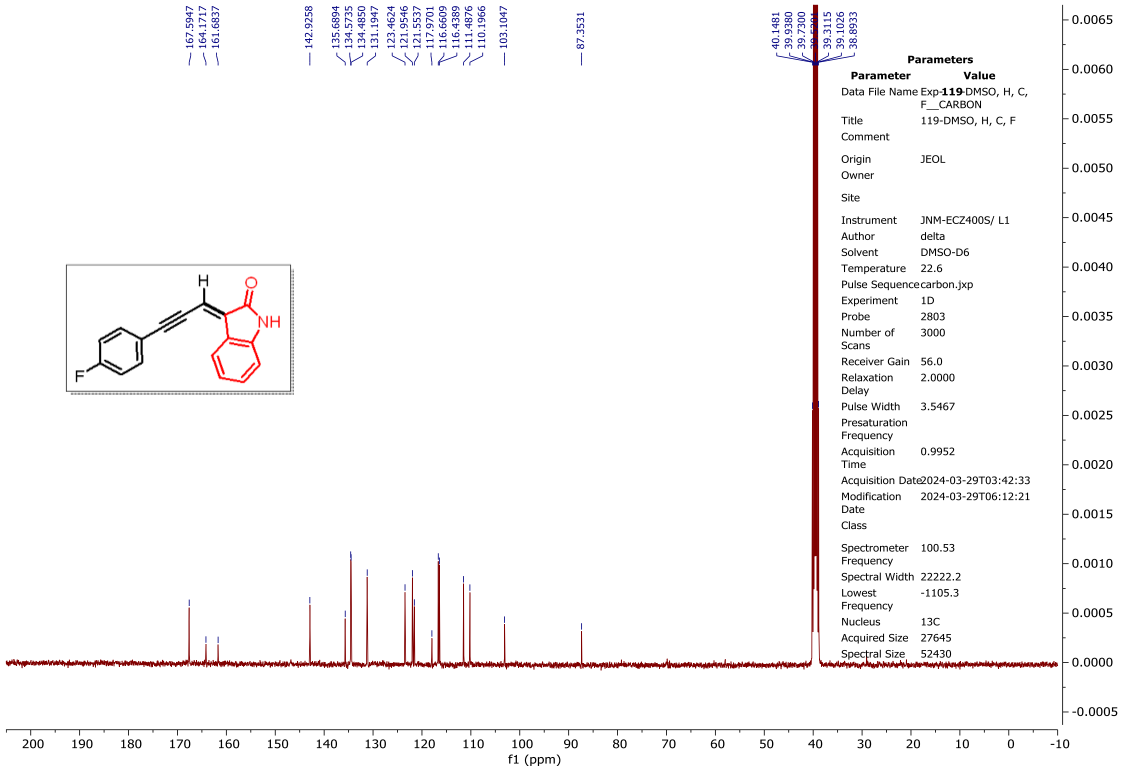

Figure S17. ^{13}**C NMR (100 MHz, DMSO-*d*$_6$) spectra of compound (4f)**

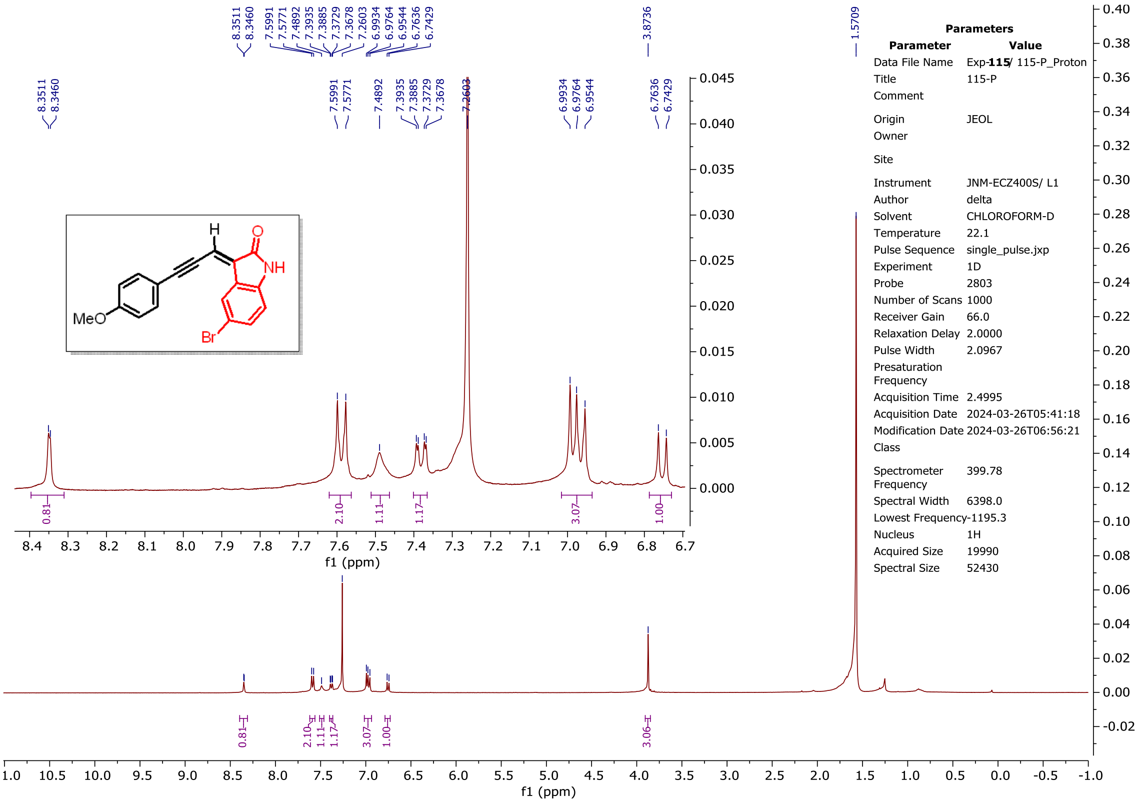

Figure S18. ^{1}**H NMR (400 MHz, CDCl$_3$) spectra of compound (4g)**

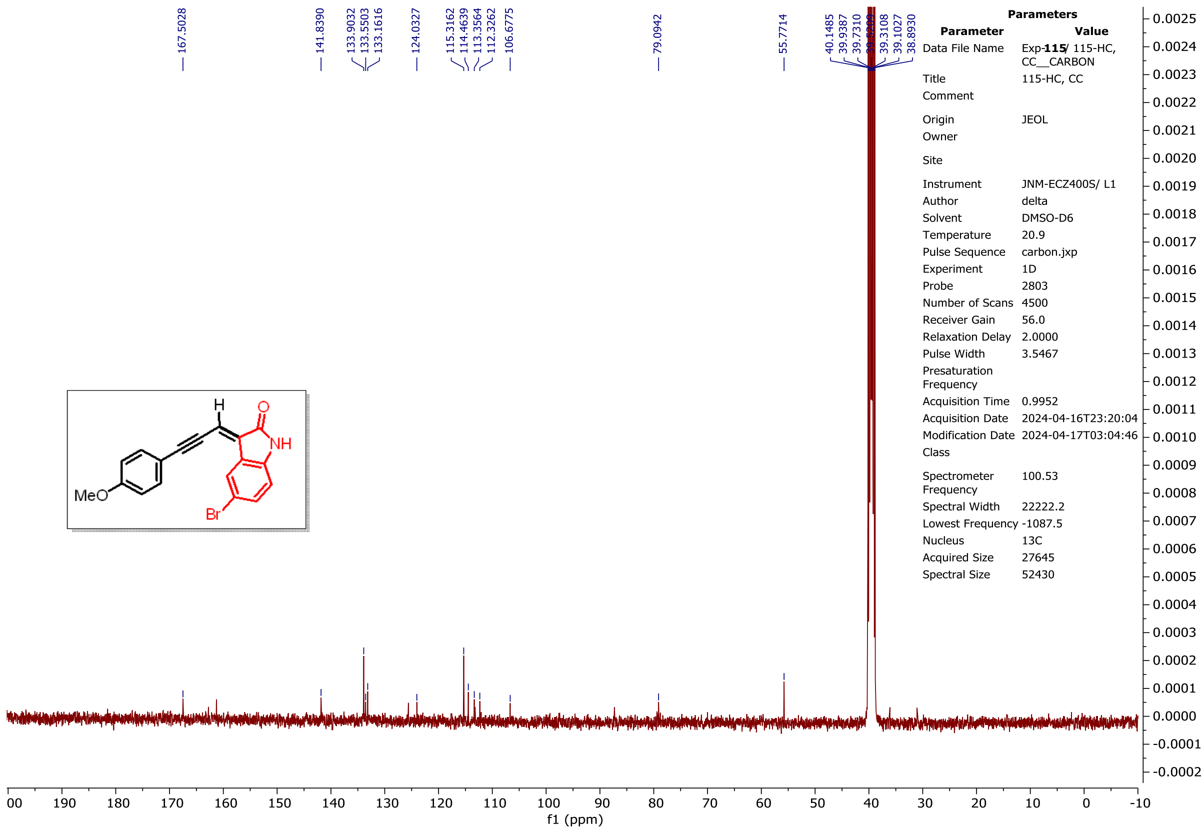

Figure S19. ^{13}C NMR (100 MHz, DMSO-d_6) spectra of compound (4g)

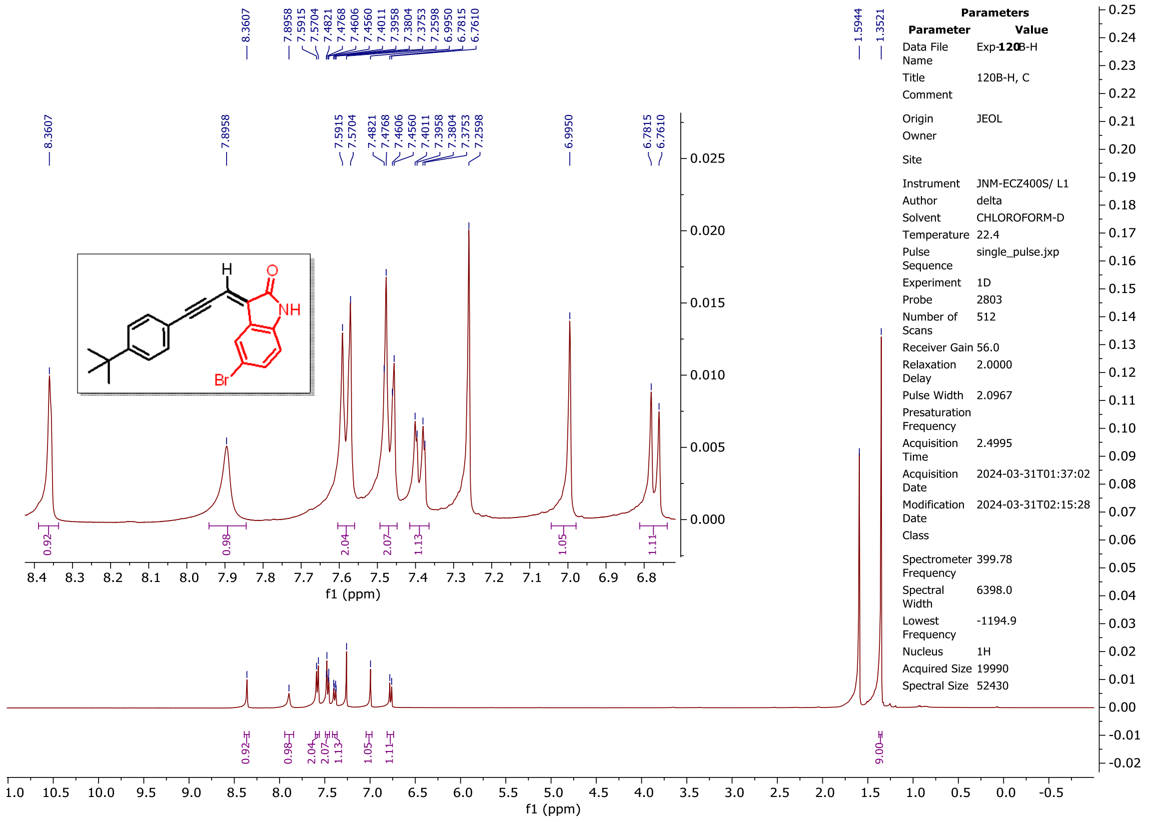

Figure S20. ^{1}H NMR (400 MHz, $CDCl_3$) spectra of compound (4h)

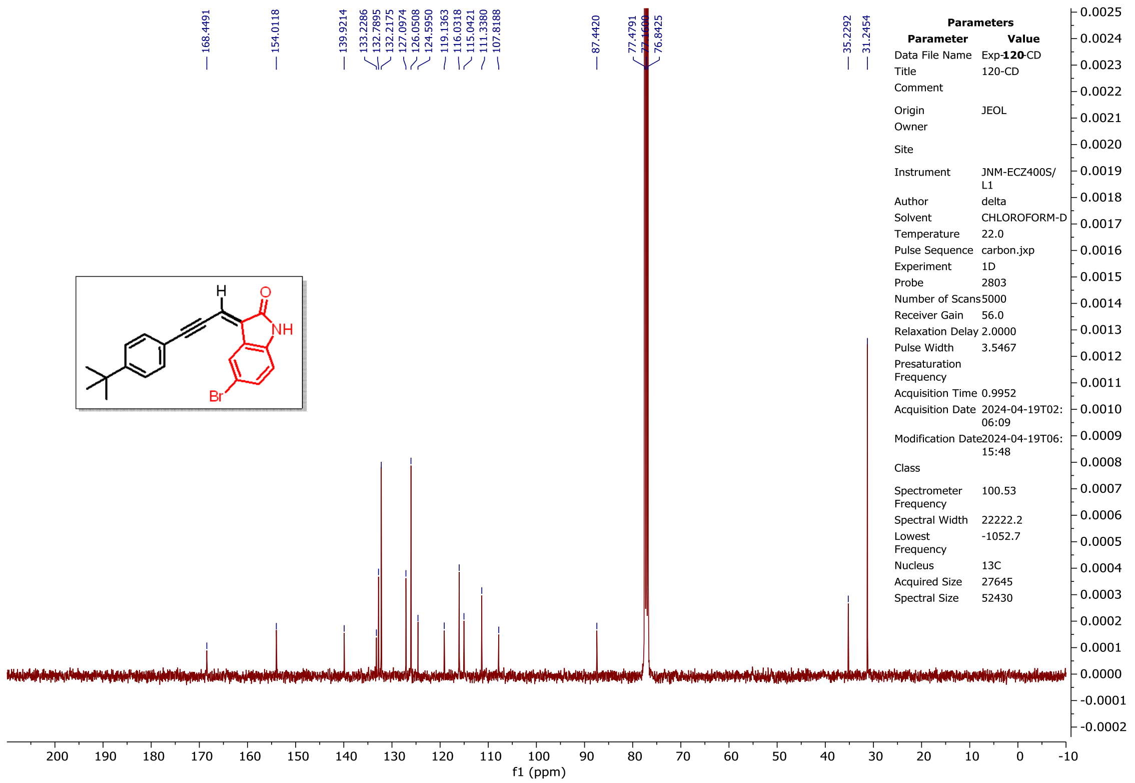

Figure S21. ^{13}**C NMR (100 MHz, CDCl$_3$) spectra of compound (4h)**

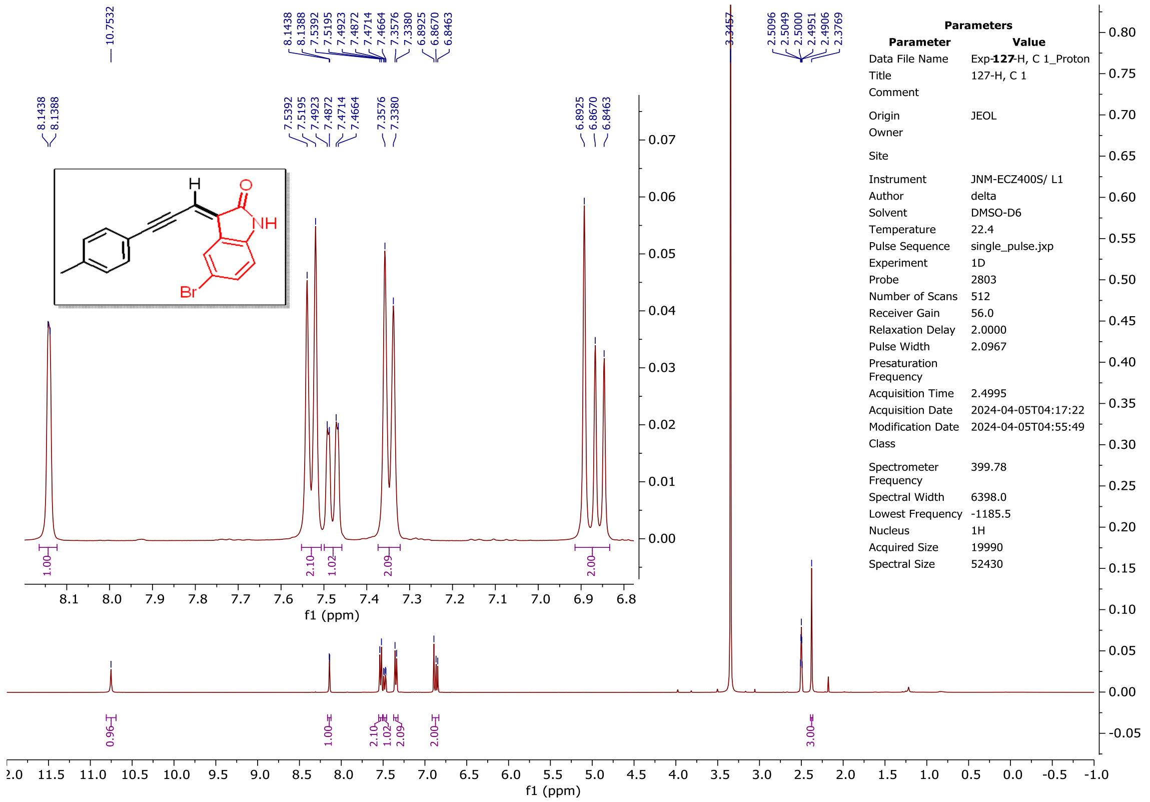

Figure S22. ^{1}**H NMR (400 MHz, DMSO-*d*$_6$) spectra of compound (4i)**

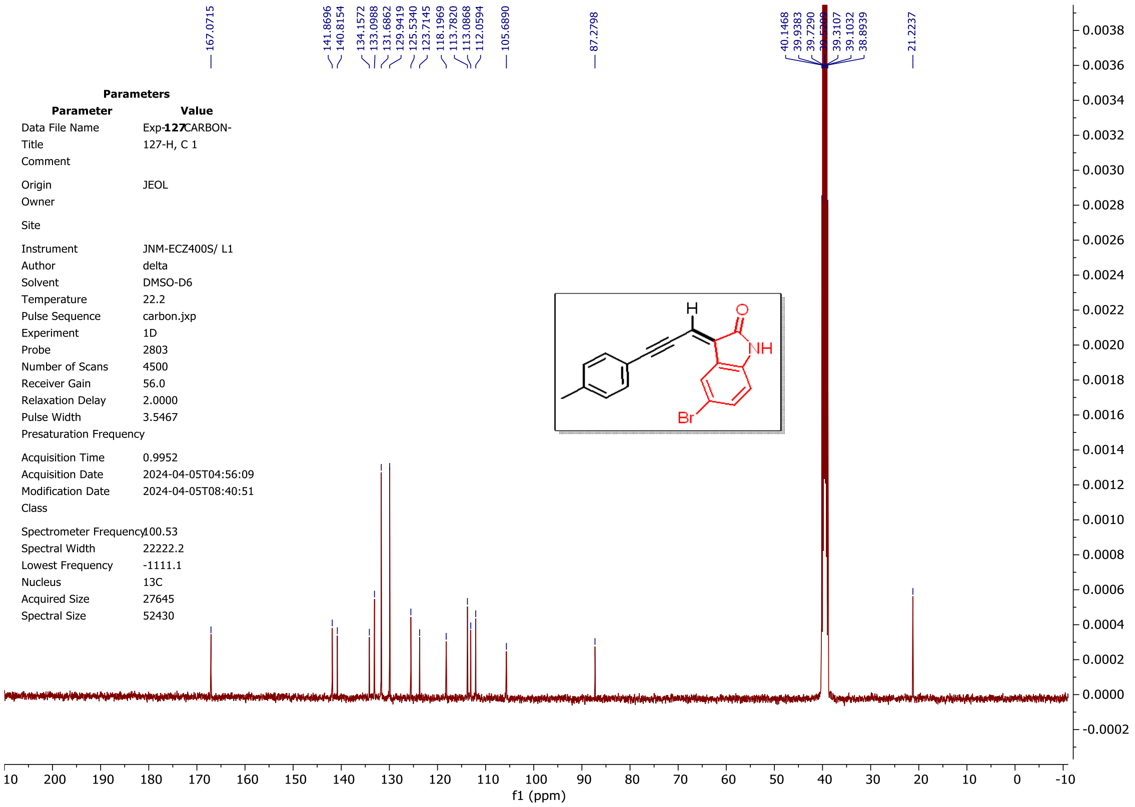

Figure S23. ^{13}**C NMR (100 MHz, DMSO-*d*$_6$) spectra of compound (4i)**

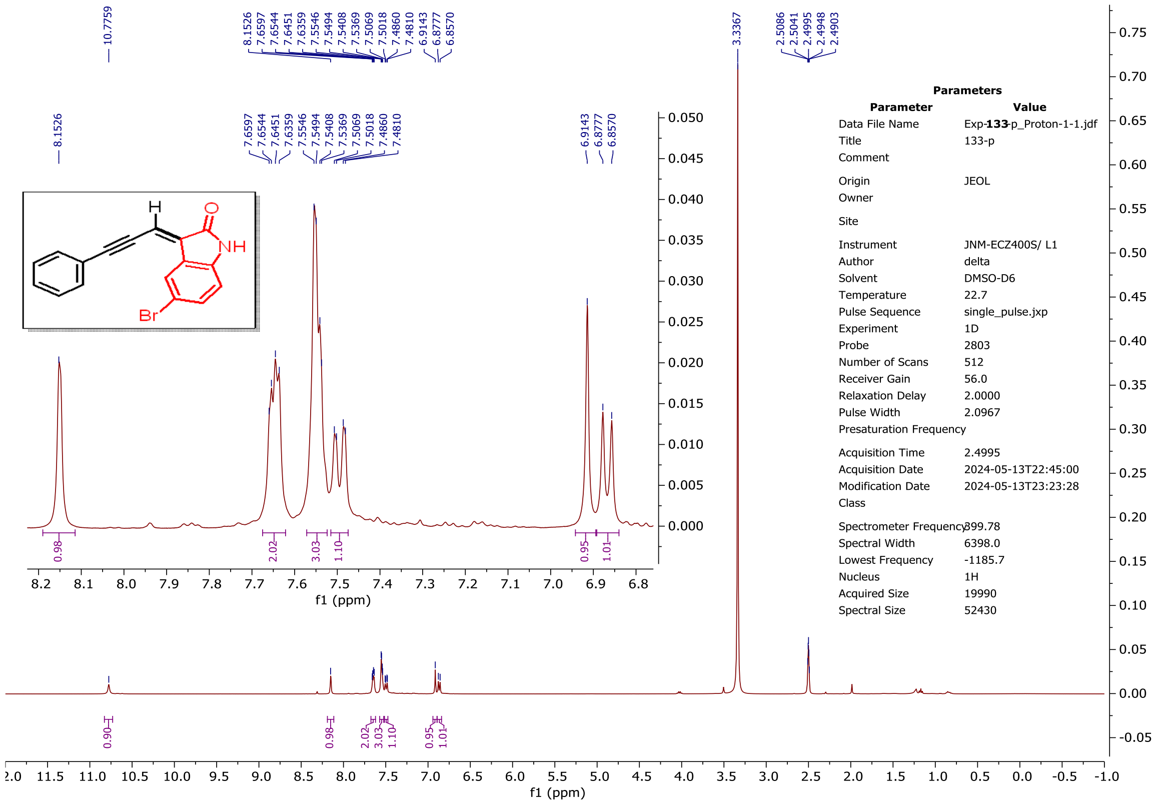

Figure S24. ^{1}**H NMR (400 MHz, DMSO-*d*$_6$) spectra of compound (4j)**

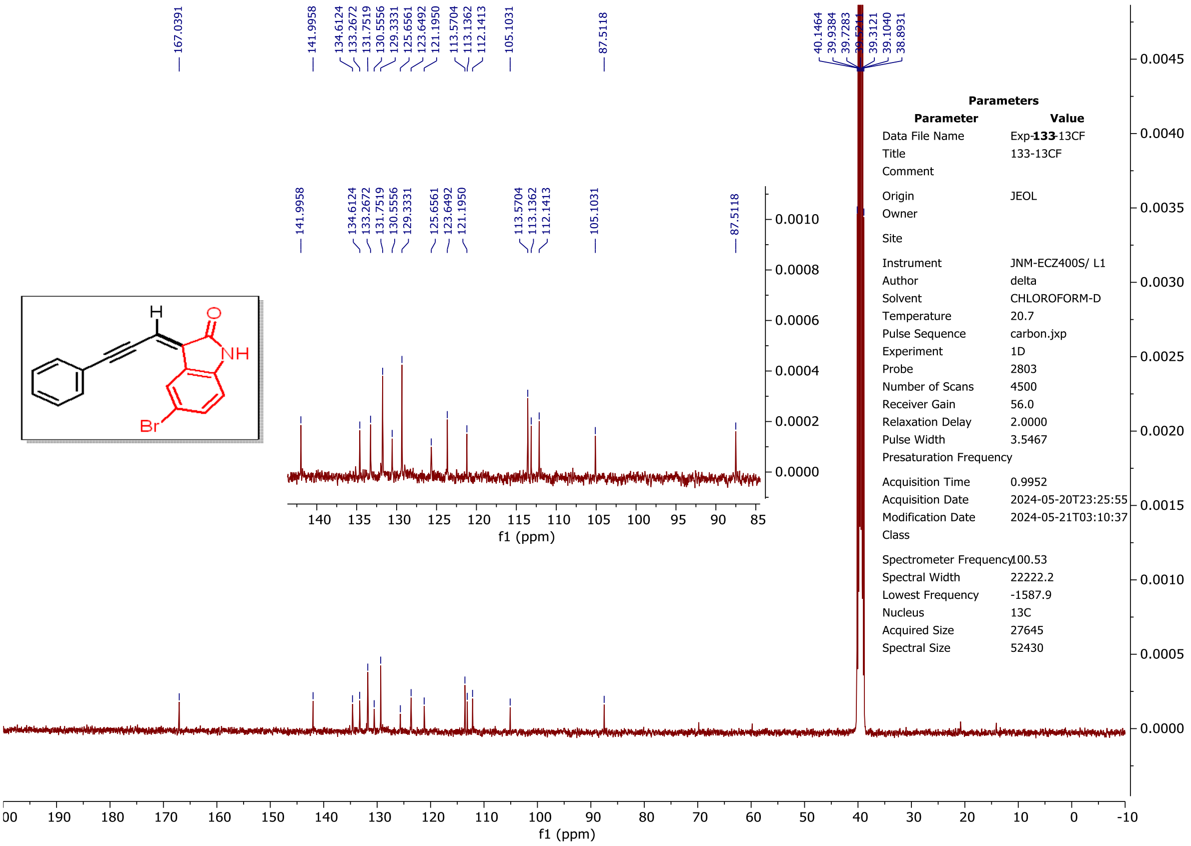

Figure S25. ^{13}C NMR (100 MHz, DMSO-d_6) spectra of compound (4j)

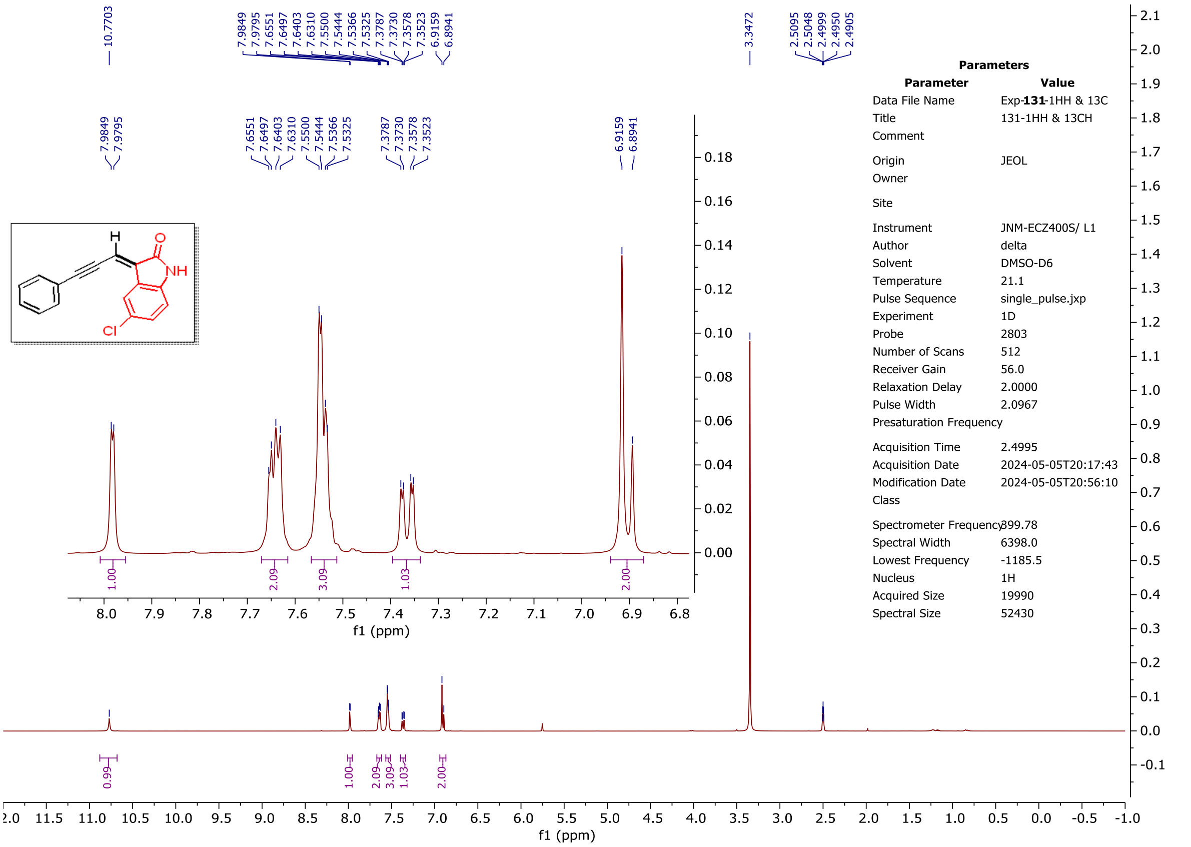

Figure S26. ^{1}H NMR (400 MHz, DMSO-d_6) spectra of compound (4k)

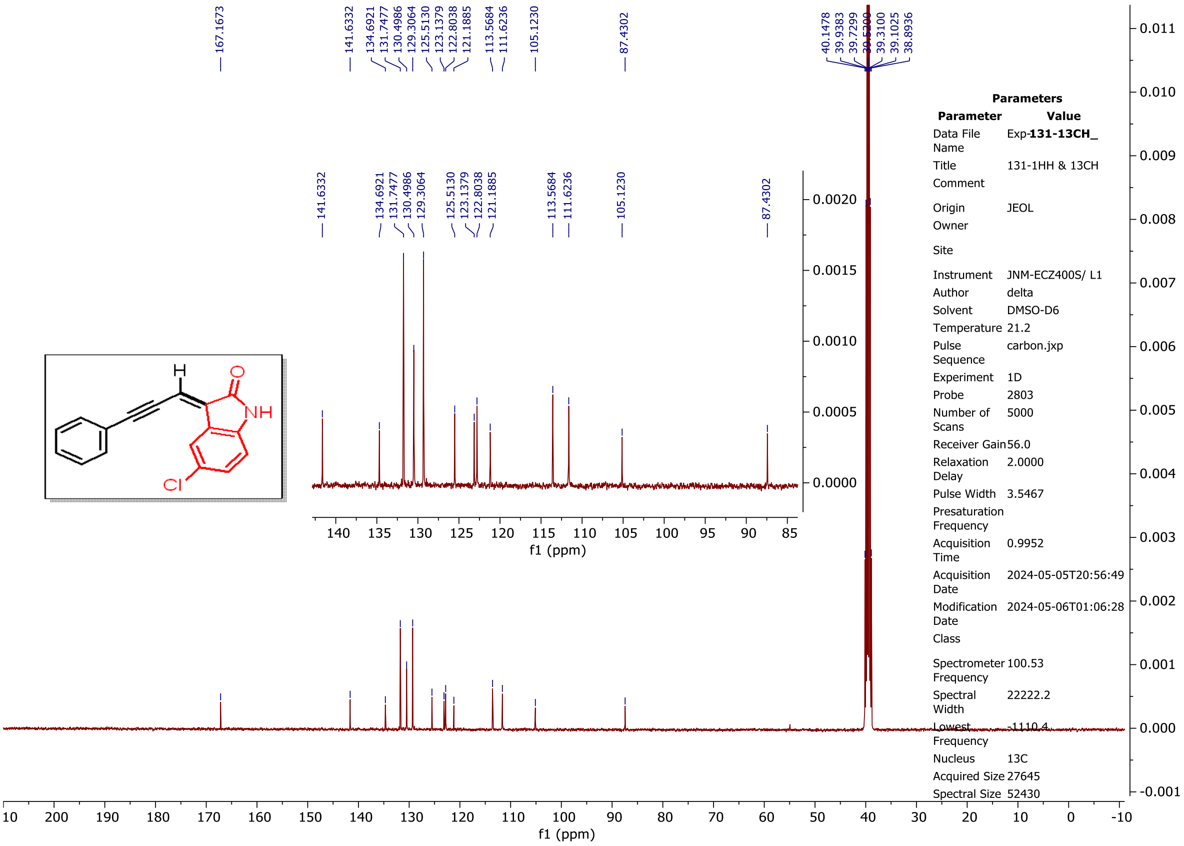

Figure S27. ^{13}C NMR (100 MHz, DMSO-d_6) spectra of compound (4k)

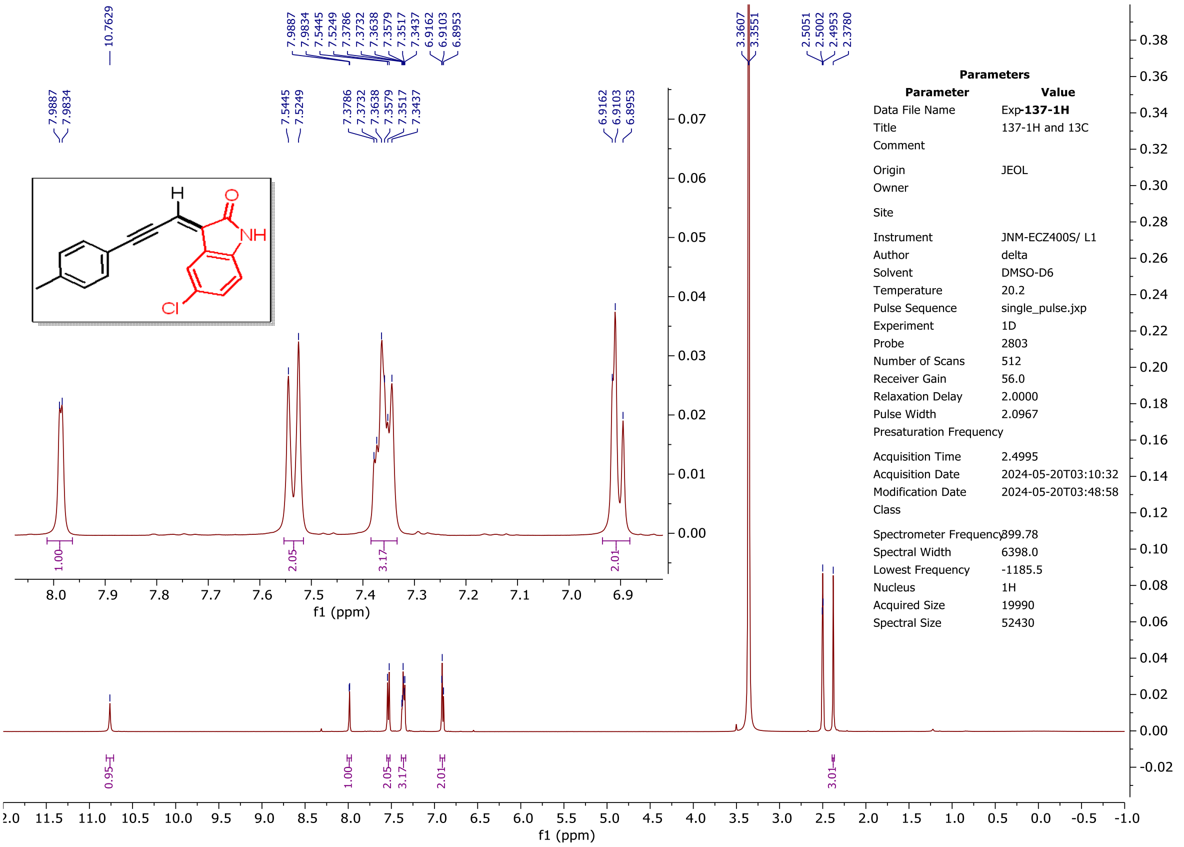

Figure S28. ^{1}H NMR (400 MHz, DMSO-d_6) spectra of compound (4l)

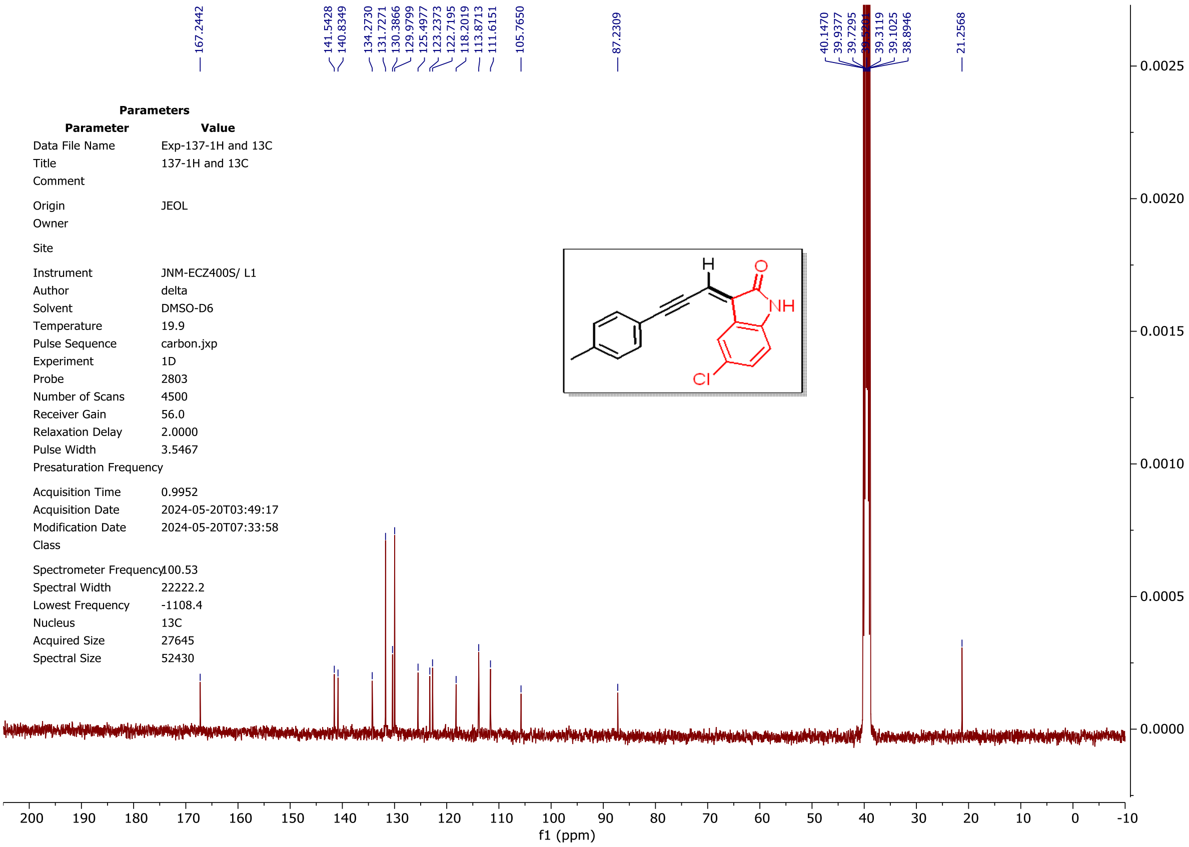

Figure S29. ^{13}C **NMR (100 MHz, DMSO-*d*$_6$) spectra of compound (4l)**

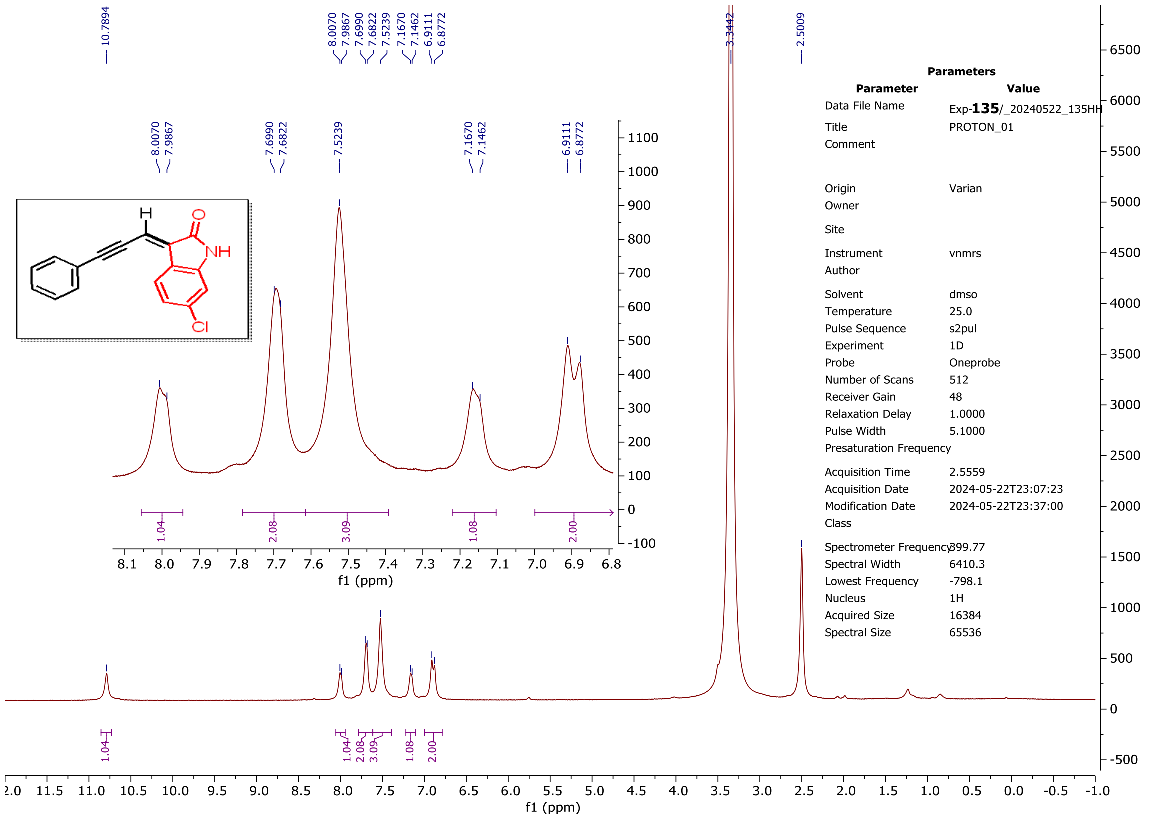

Figure S30. ^{1}H **NMR (400 MHz, DMSO-*d*$_6$) spectra of compound (4m)**

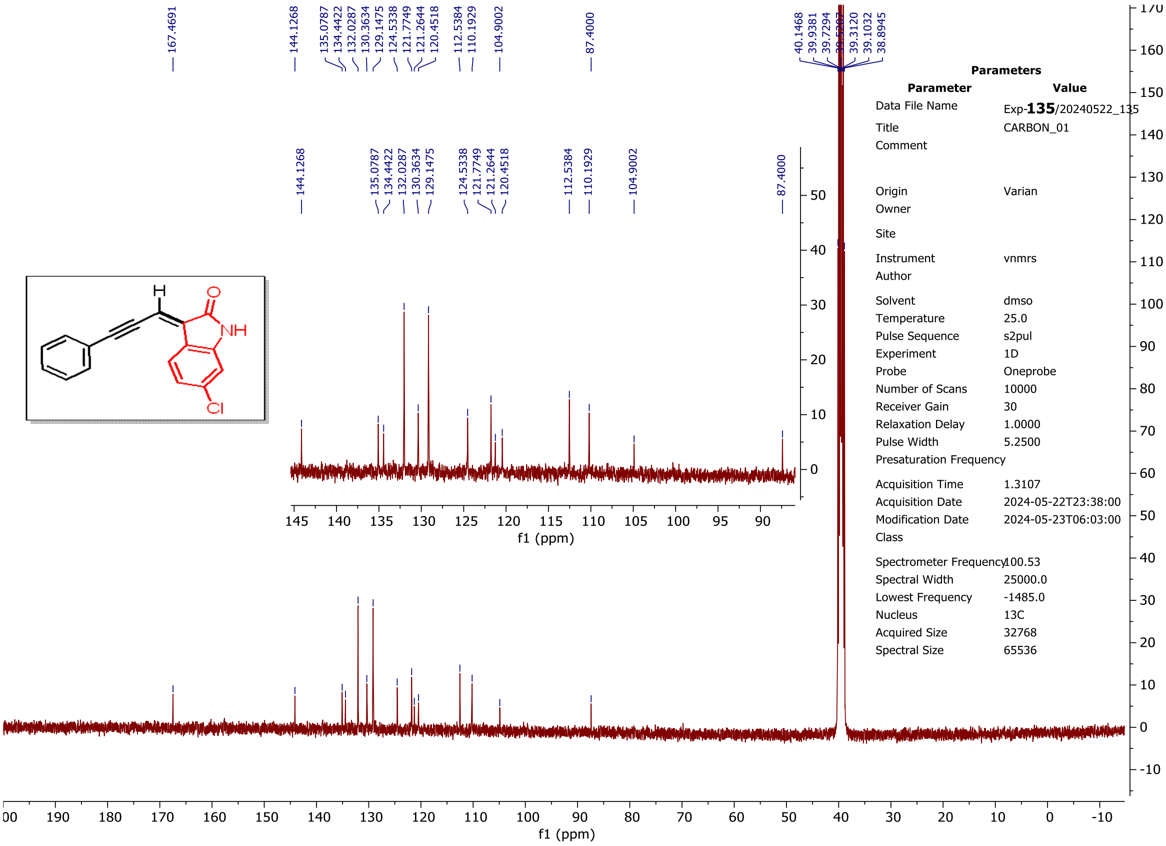

Figure S31. ^{13}C **NMR (100 MHz, DMSO-d_6) spectra of compound (4m)**

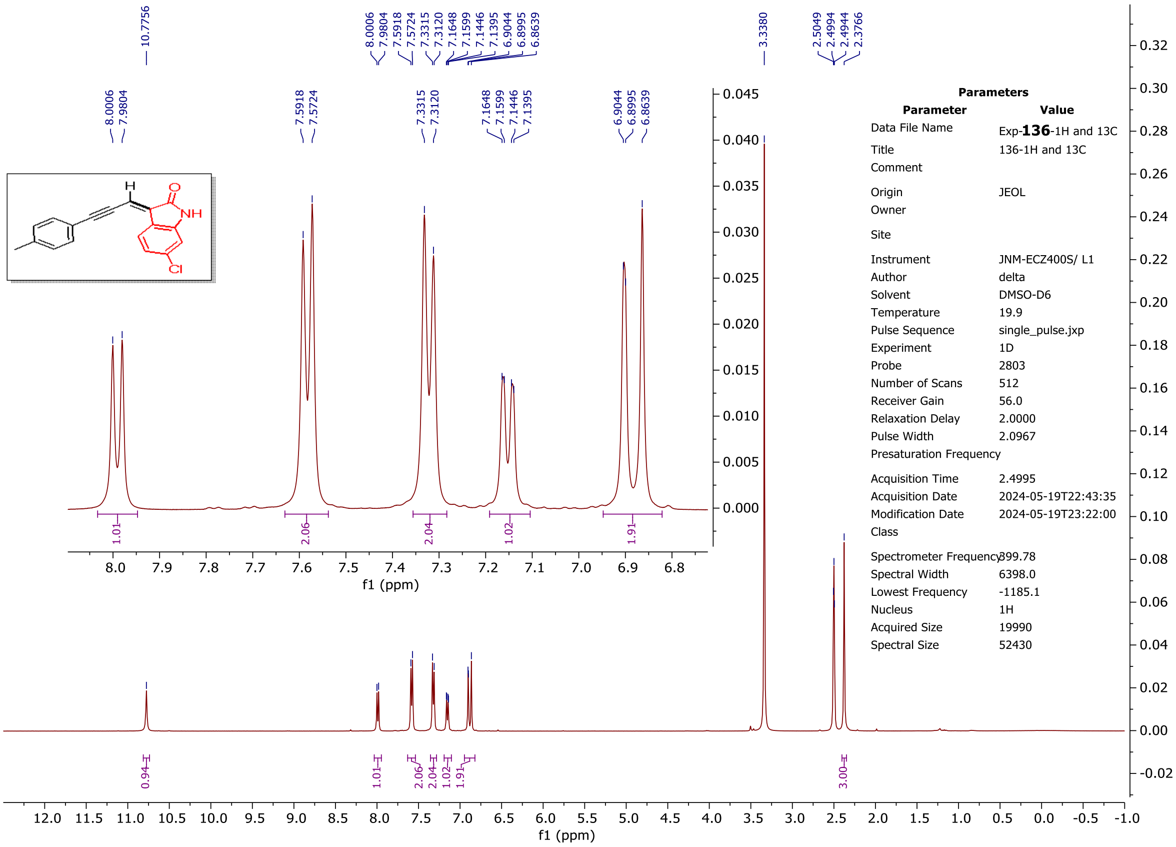

Figure S32. ^{1}H **NMR (400 MHz, DMSO-d_6) spectra of compound (4n)**

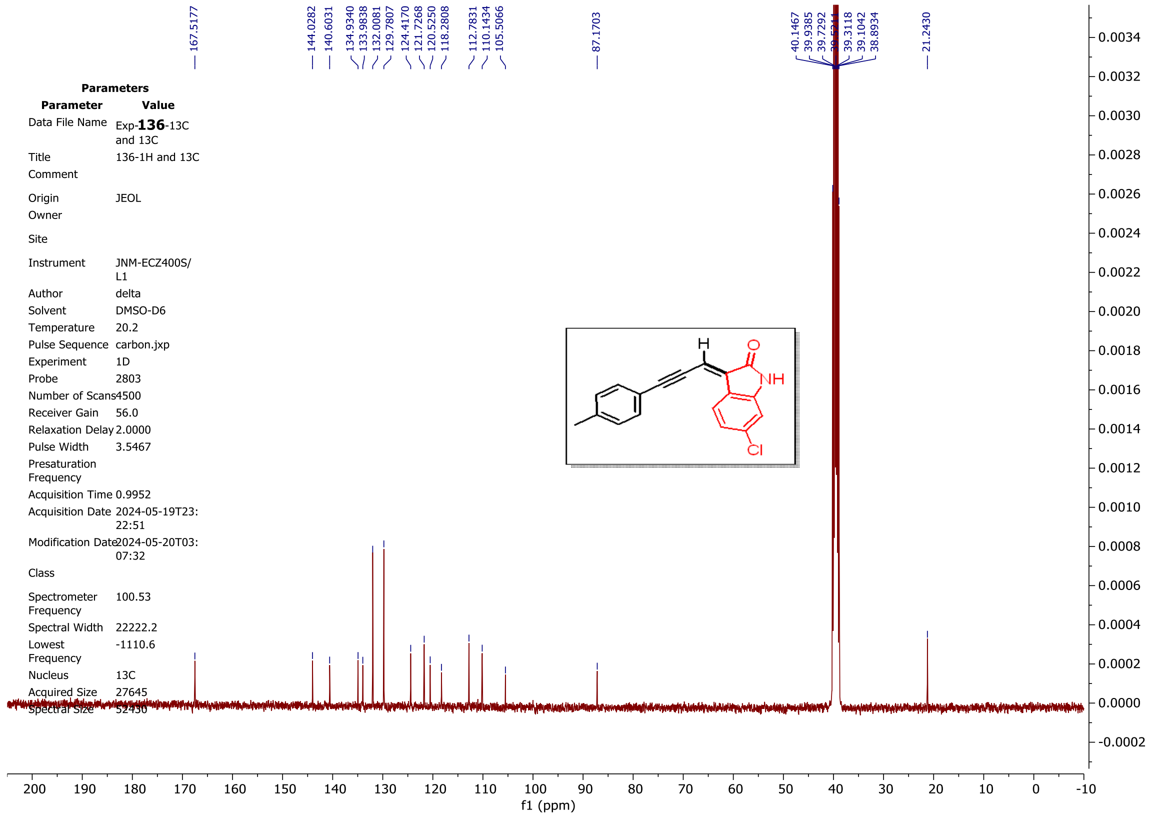

Figure S33. ^{13}C **NMR (100 MHz, DMSO-d_6) spectra of compound (4n)**

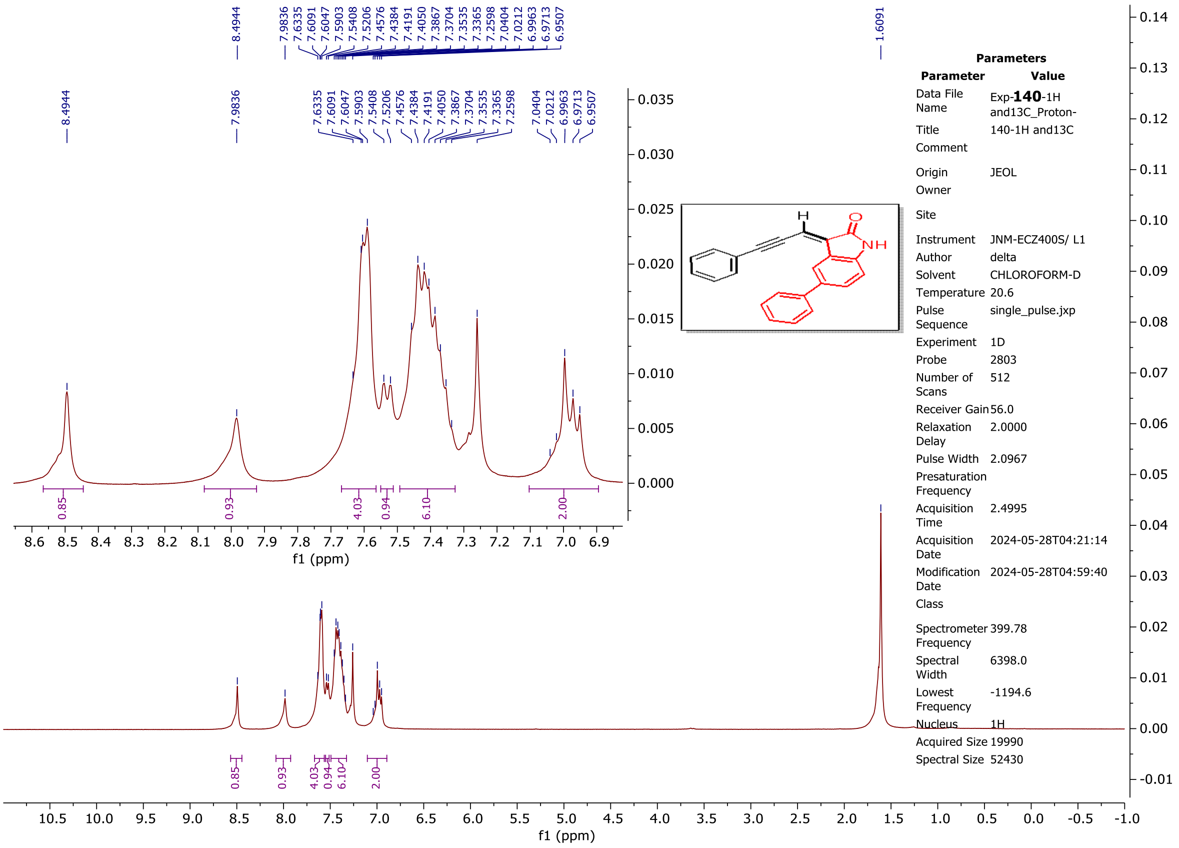

Figure S34. ^{1}H **NMR (400 MHz, $CDCl_3$) spectra of compound (4o)**

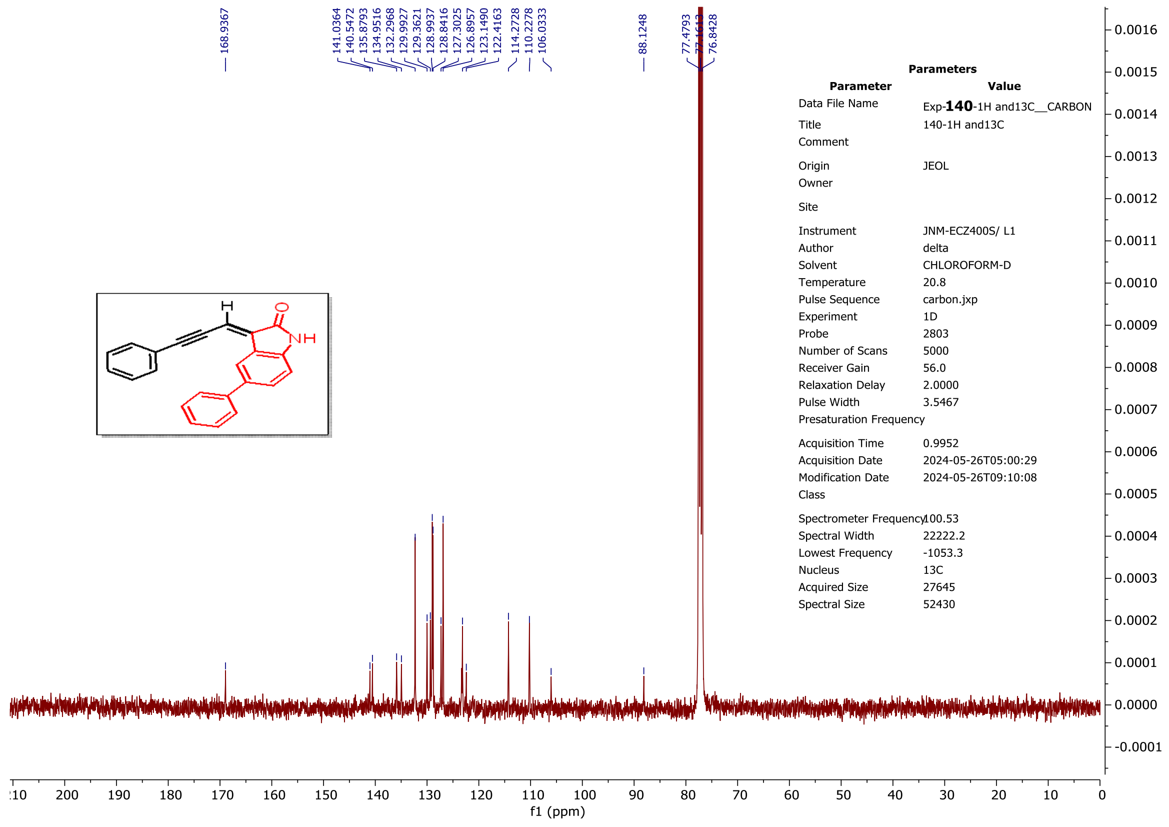

Figure S35. ^{13}C NMR (100 MHz, $CDCl_3$) spectra of compound (4o)

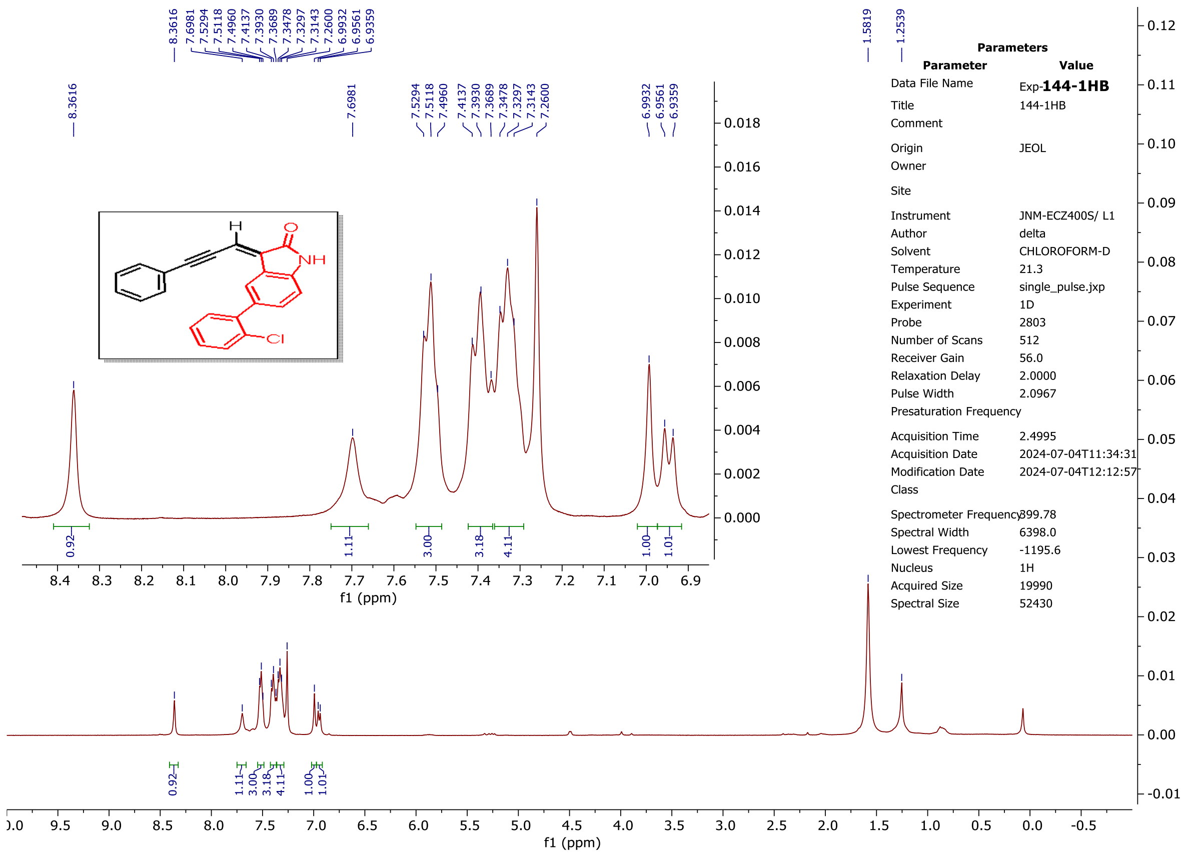

Figure S36. ^{1}H NMR (400 MHz, $CDCl_3$) spectra of compound (4p)

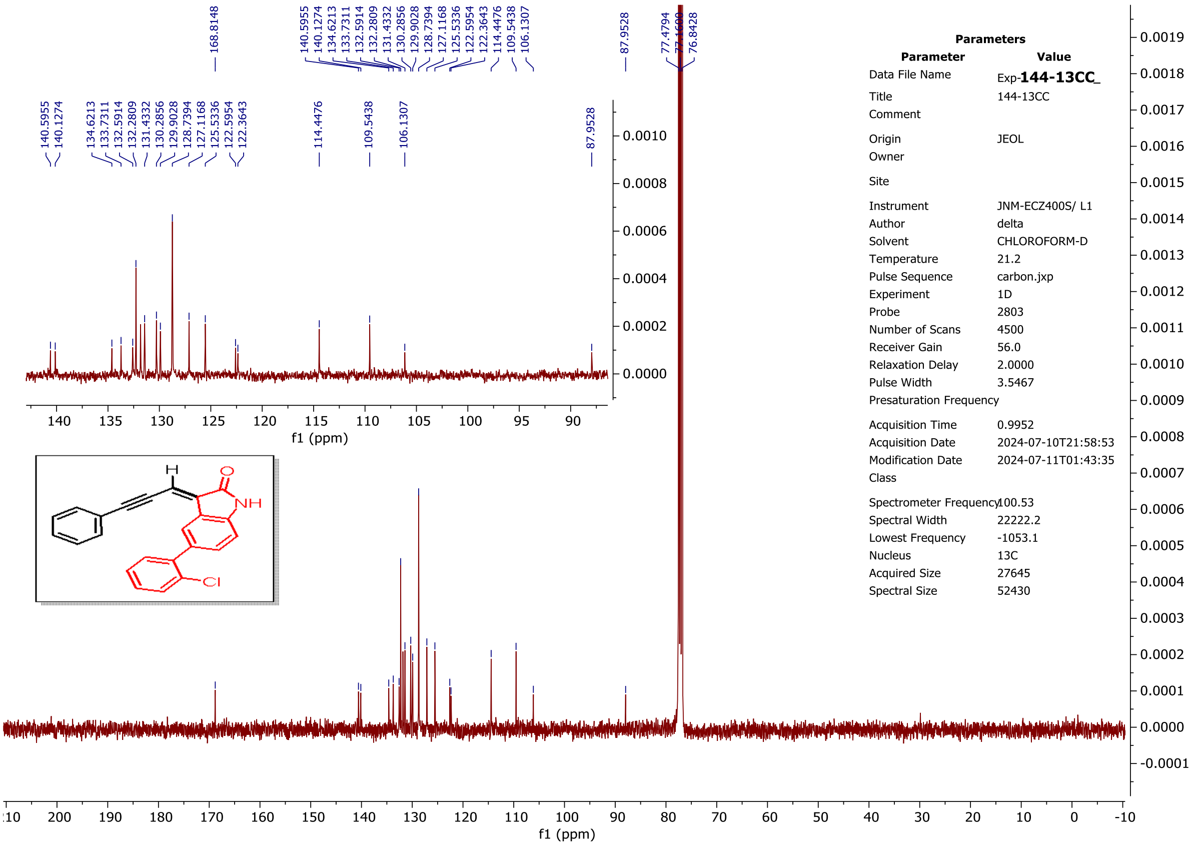

Figure S37. ^{13}**C NMR (100 MHz,** $CDCl_3$**) spectra of compound (4p)**

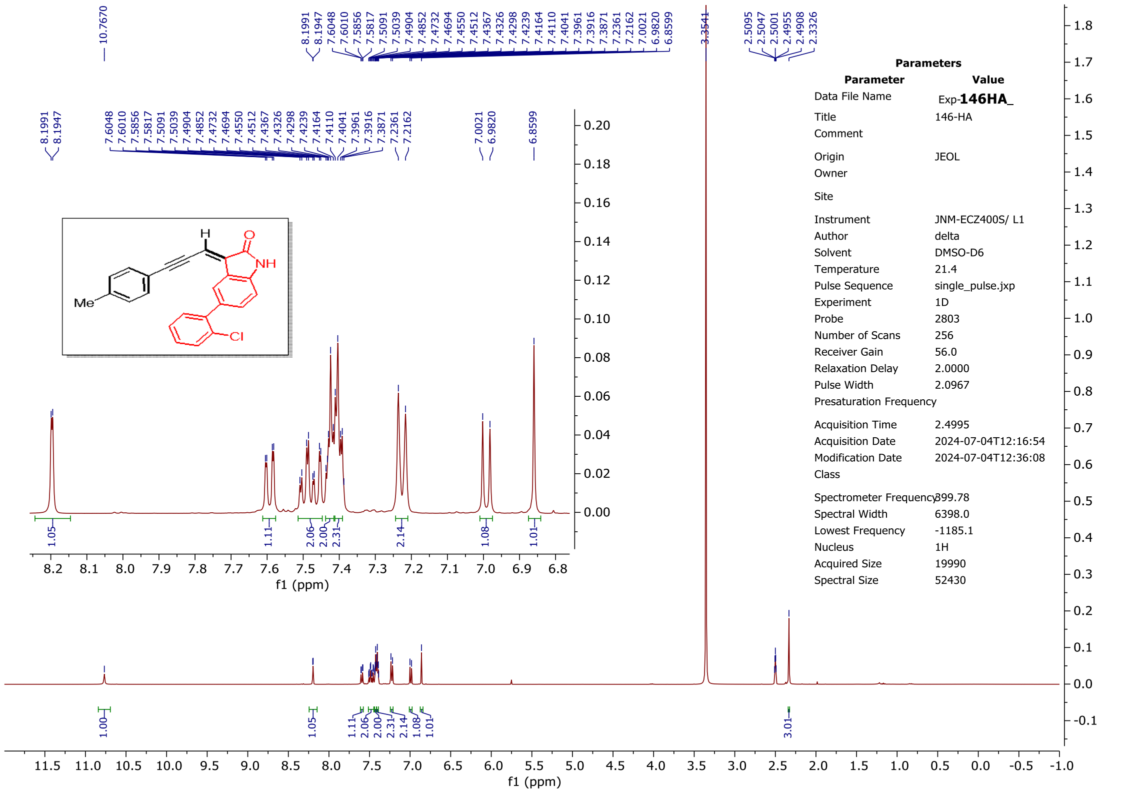

Figure S38. ^{1}**H NMR (400 MHz, DMSO-**d_6**) spectra of compound (4q)**

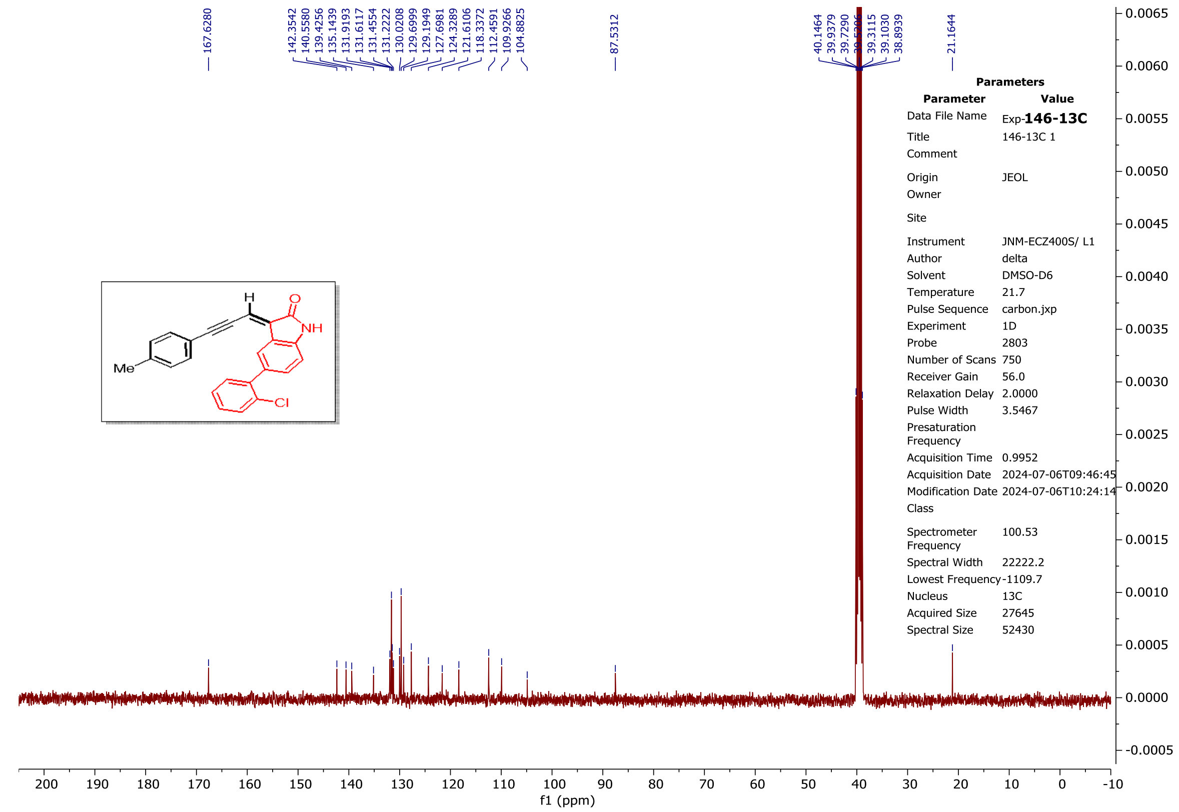

Figure S39. ^{13}**C NMR (100 MHz, DMSO-*d_6*) spectra of compound (4q)**

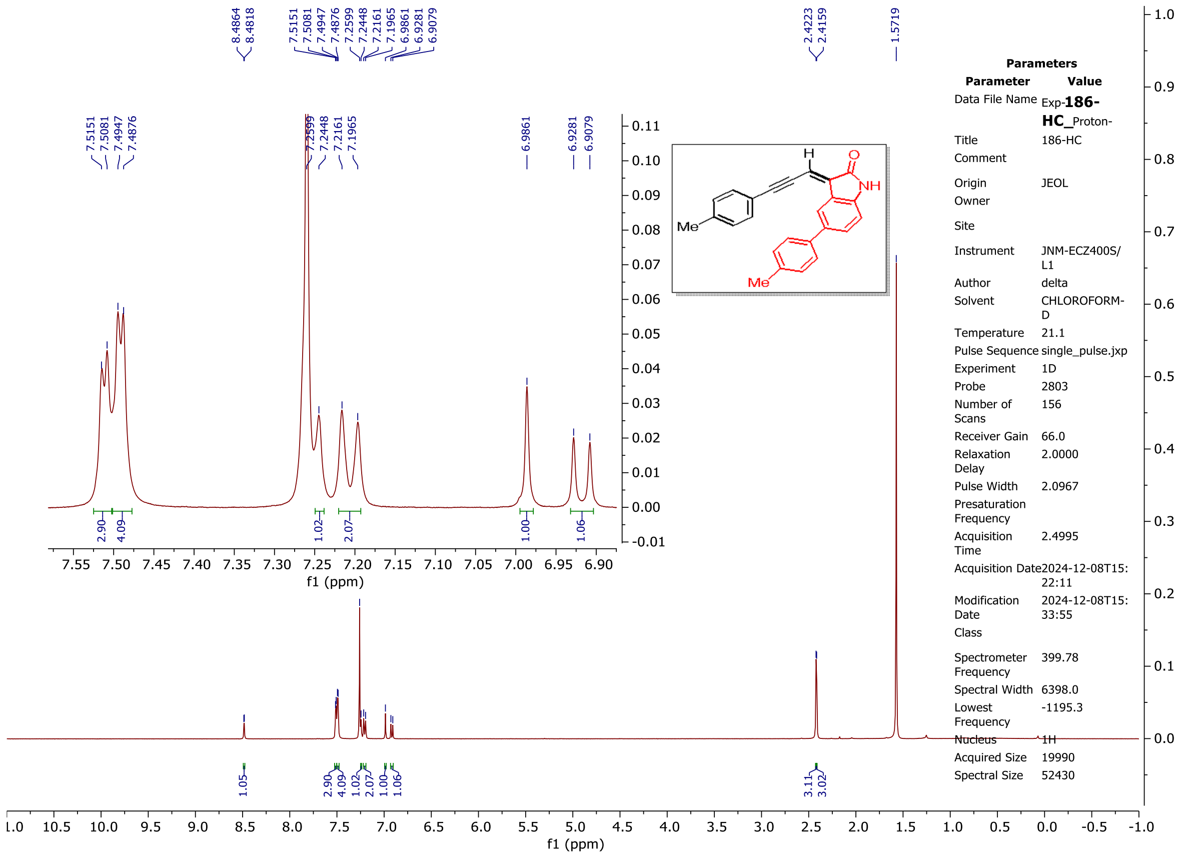

Figure S40. ^{1}**H NMR (400 MHz, $CDCl_3$) spectra of compound (4r)**

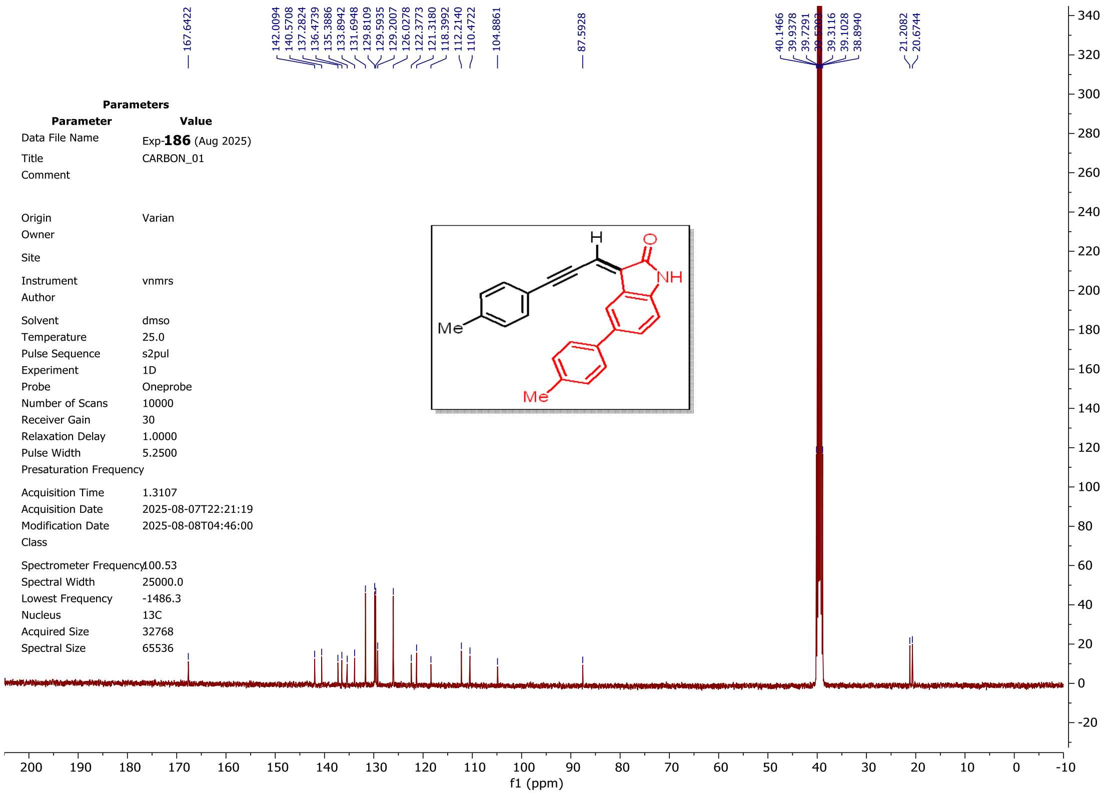

Figure S41. ^{13}C NMR (100 MHz, $CDCl_3$) spectra of compound (4r)

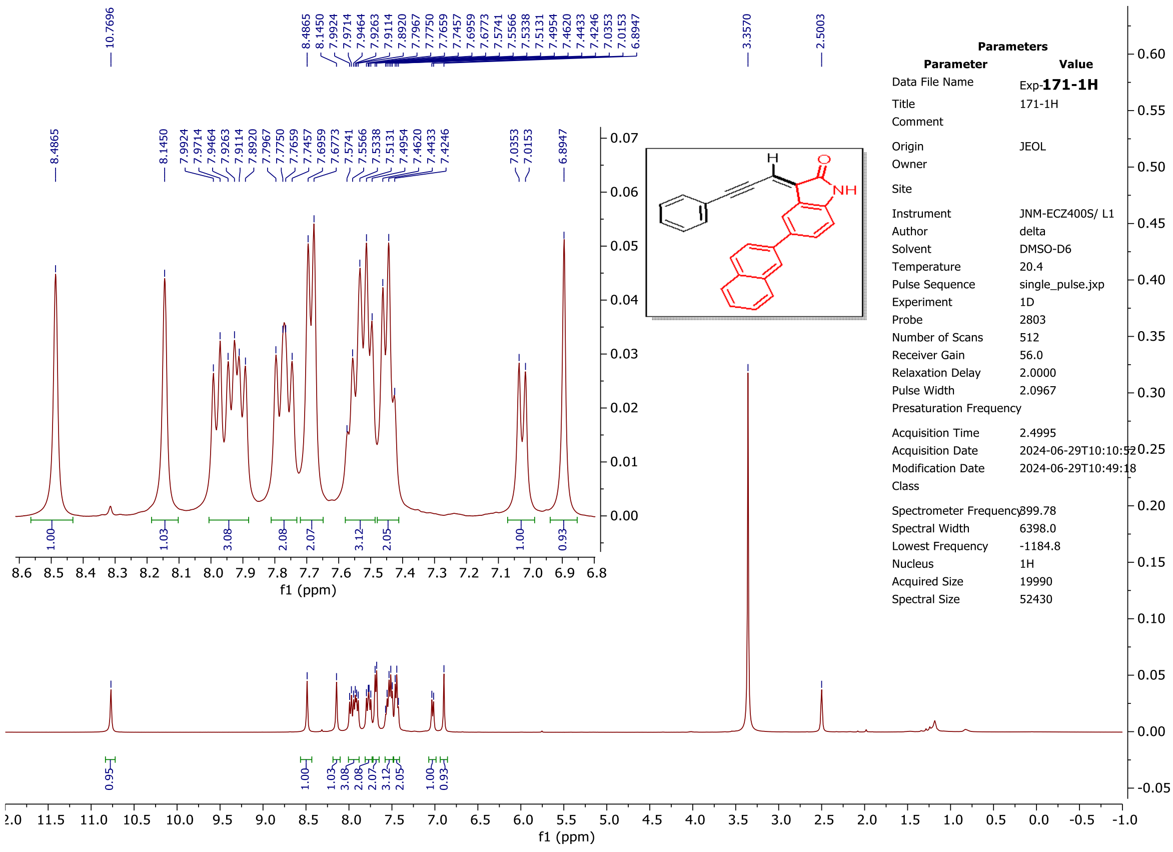

Figure S42. ^{1}H NMR (400 MHz, DMSO-d_6) spectra of compound (4s)

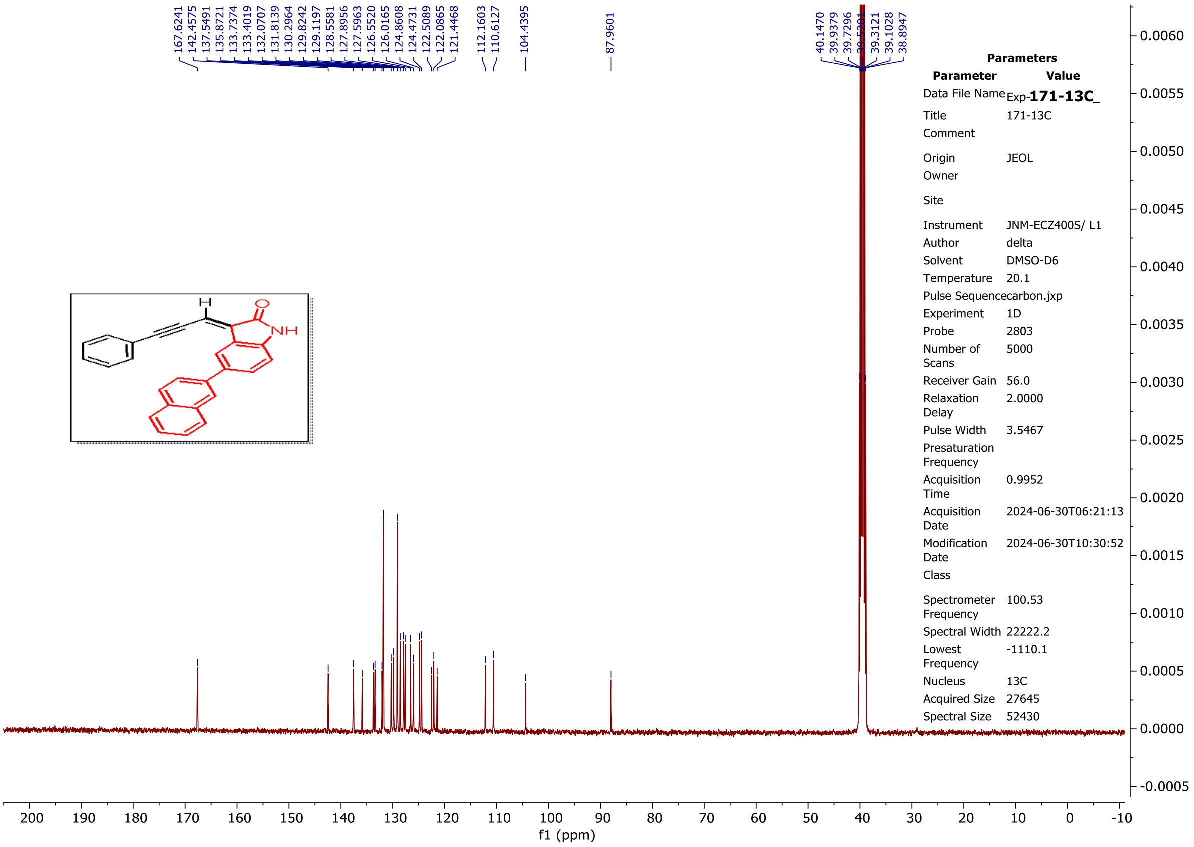

Figure S43. ^{13}C NMR (100 MHz, DMSO-d_6) spectra of compound (4s)

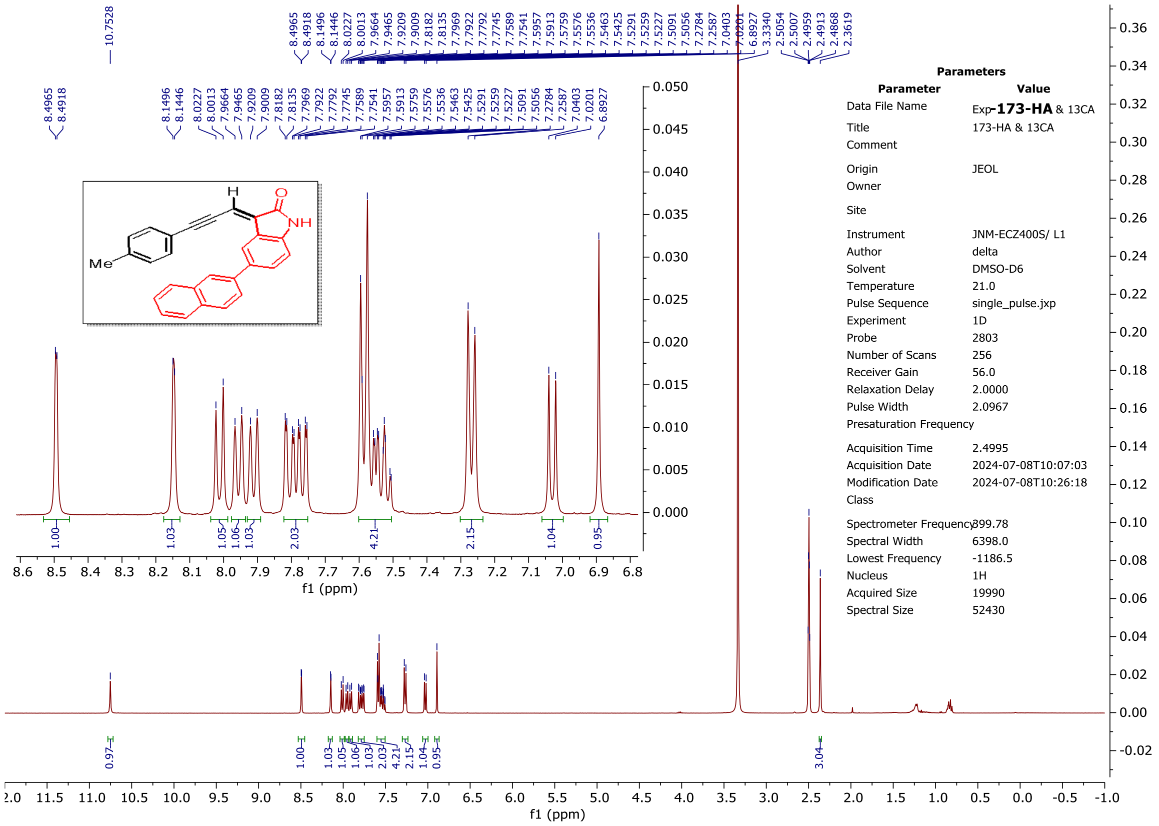

Figure S44. ^{1}H NMR (400 MHz, DMSO-d_6) spectra of compound (4t)

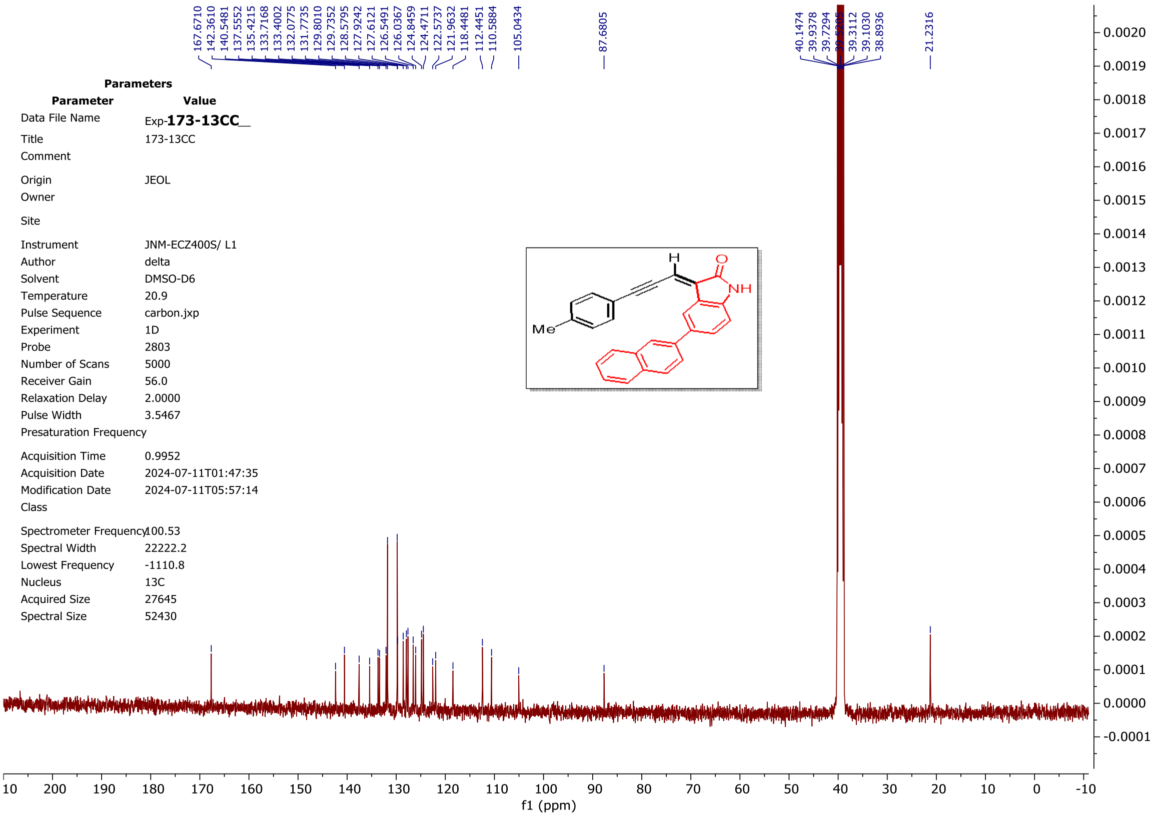

Figure S45. ^{13}C **NMR (100 MHz, DMSO-d_6) spectra of compound (4t)**

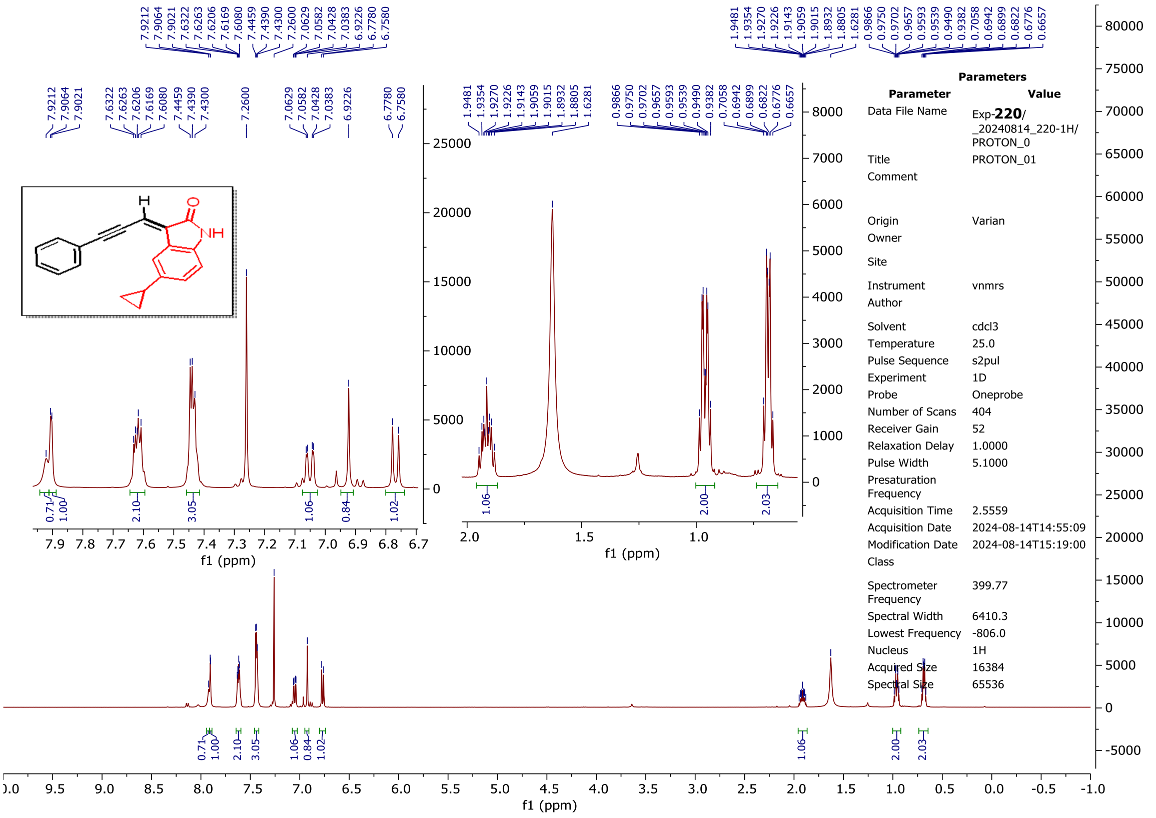

Figure S46. ^{1}H **NMR (400 MHz, $CDCl_3$) spectra of compound (4u)**

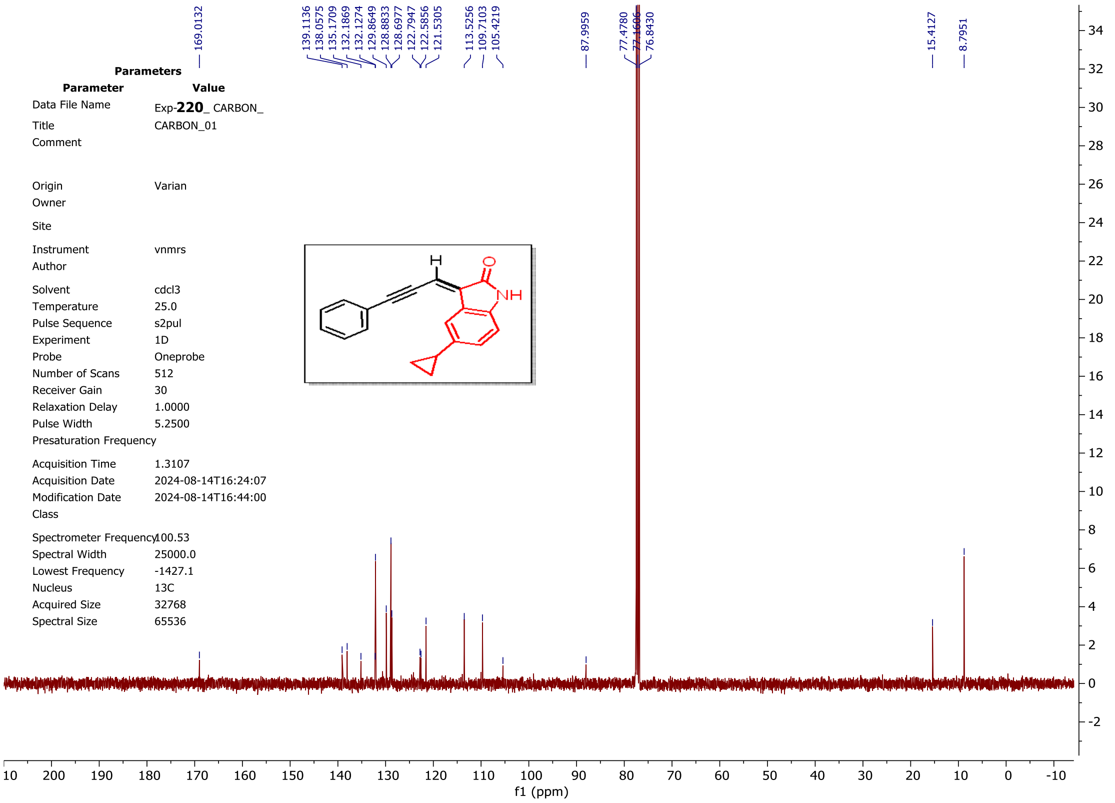

Figure S47. ^{13}C NMR (100 MHz, $CDCl_3$) spectra of compound (4u)

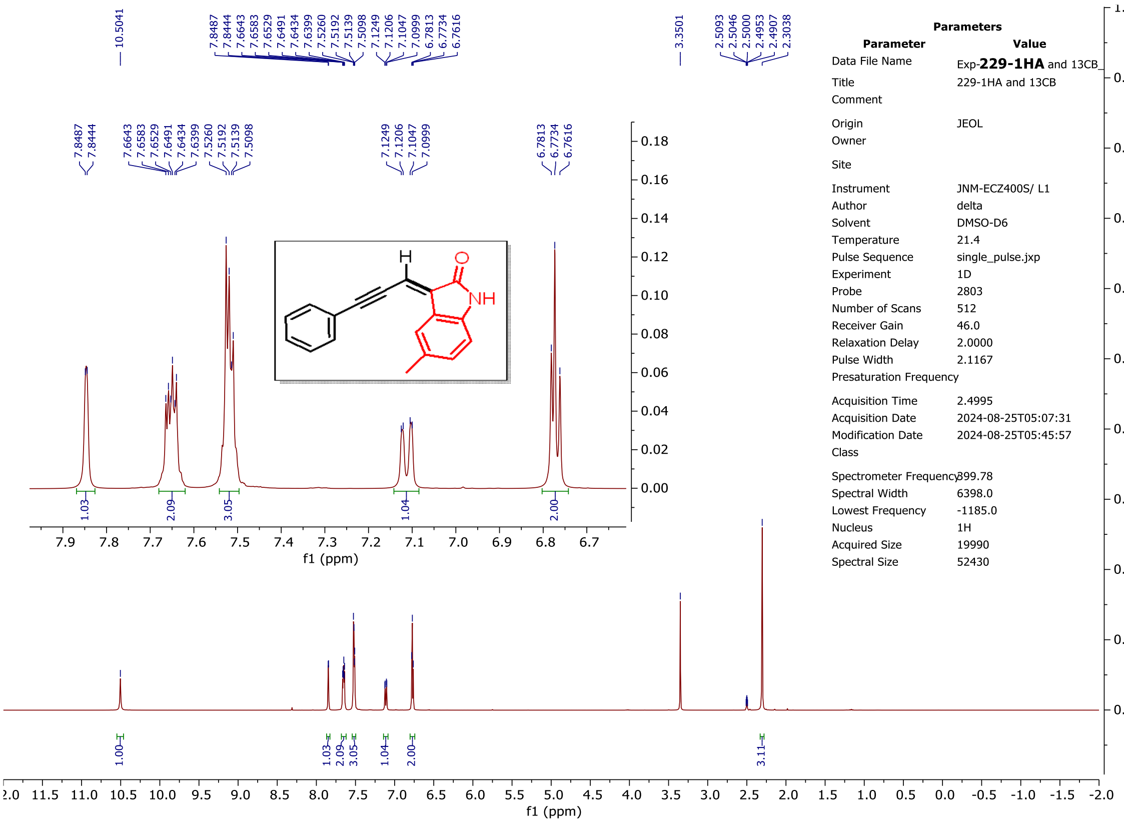

Figure S48. ^{1}H NMR (400 MHz, DMSO-d_6) spectra of compound (4v)

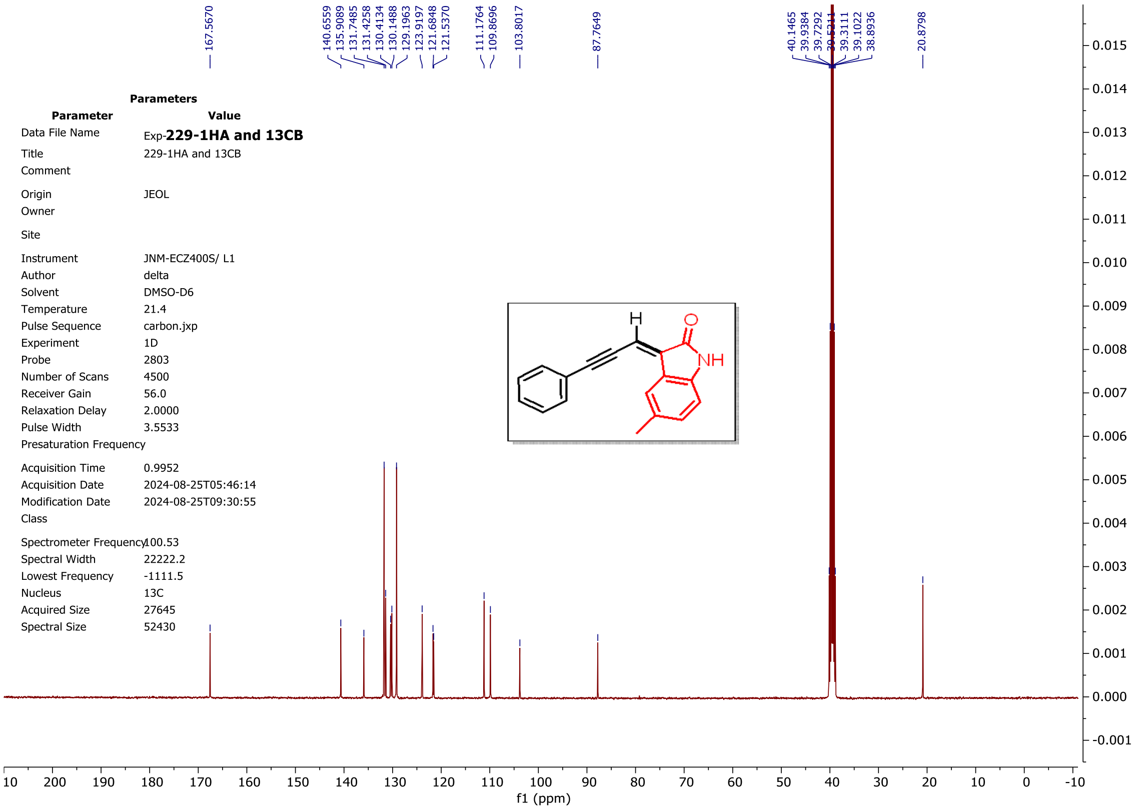

Figure S49. ^{13}**C NMR (100 MHz, DMSO-*d*$_6$) spectra of compound (4v)**

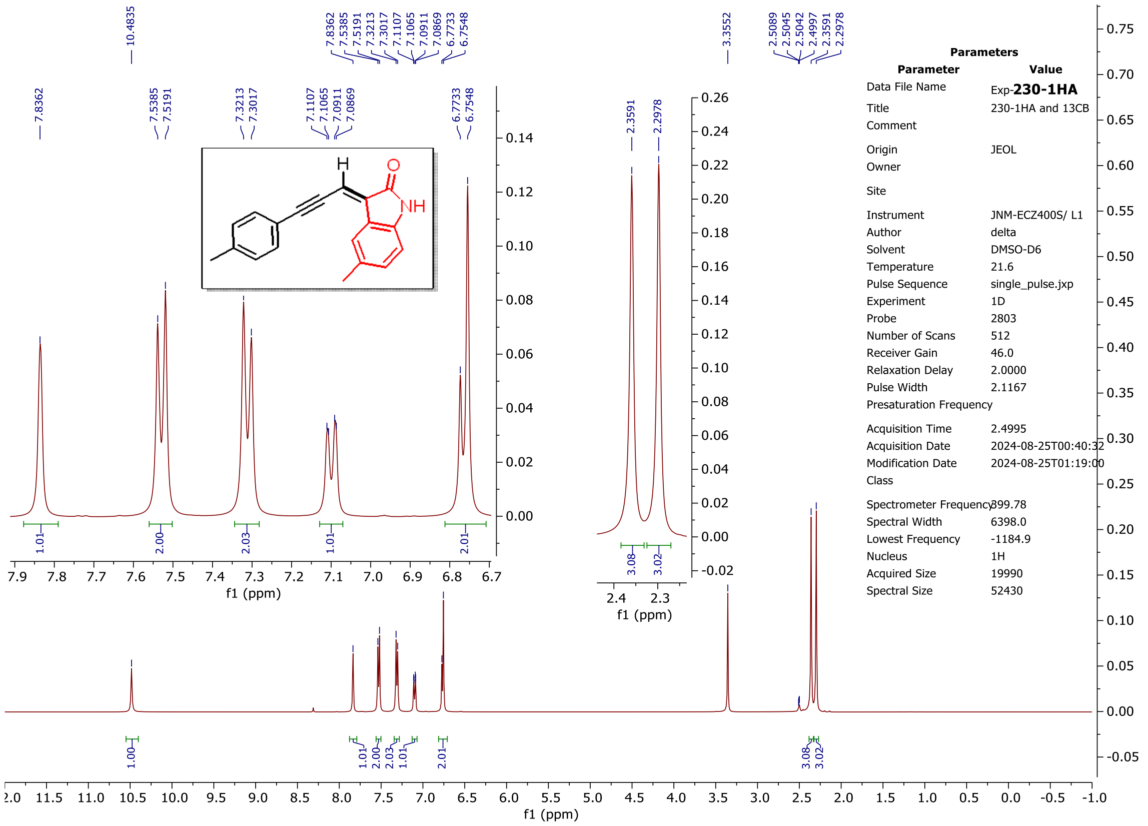

Figure S50. ^{1}**H NMR (400 MHz, DMSO-*d*$_6$) spectra of compound (4w)**

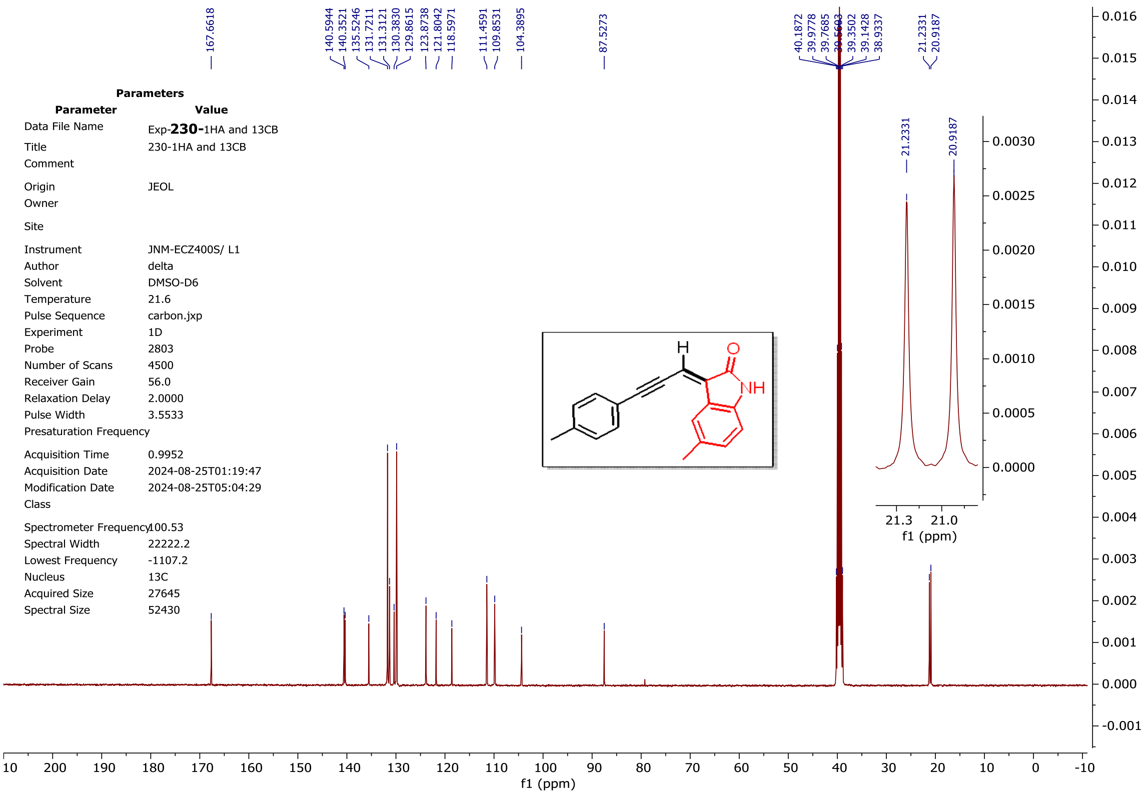

Figure S51. ^{13}C NMR (100 MHz, DMSO-d_6) spectra of compound (4w)

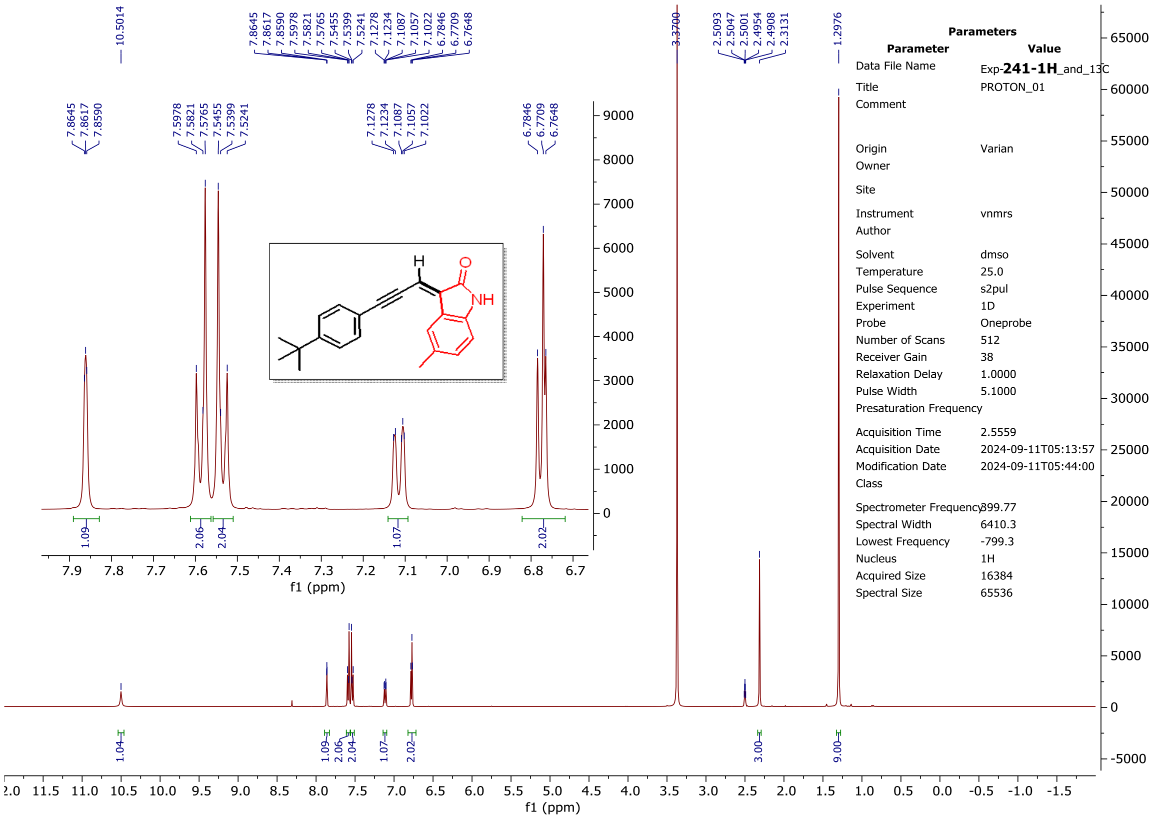

Figure S52. ^{1}H NMR (400 MHz, DMSO-d_6) spectra of compound (4x)

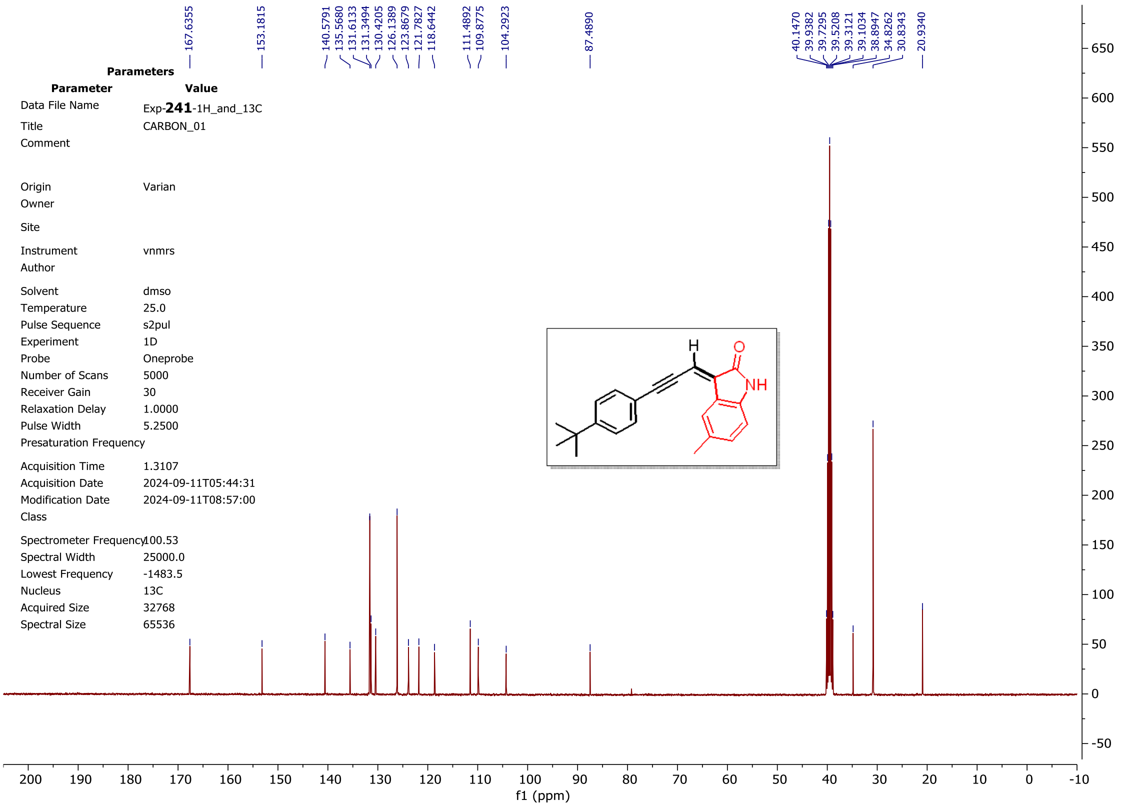

Figure S53. ^{13}C NMR (100 MHz, DMSO-d_6) spectra of compound (4x)

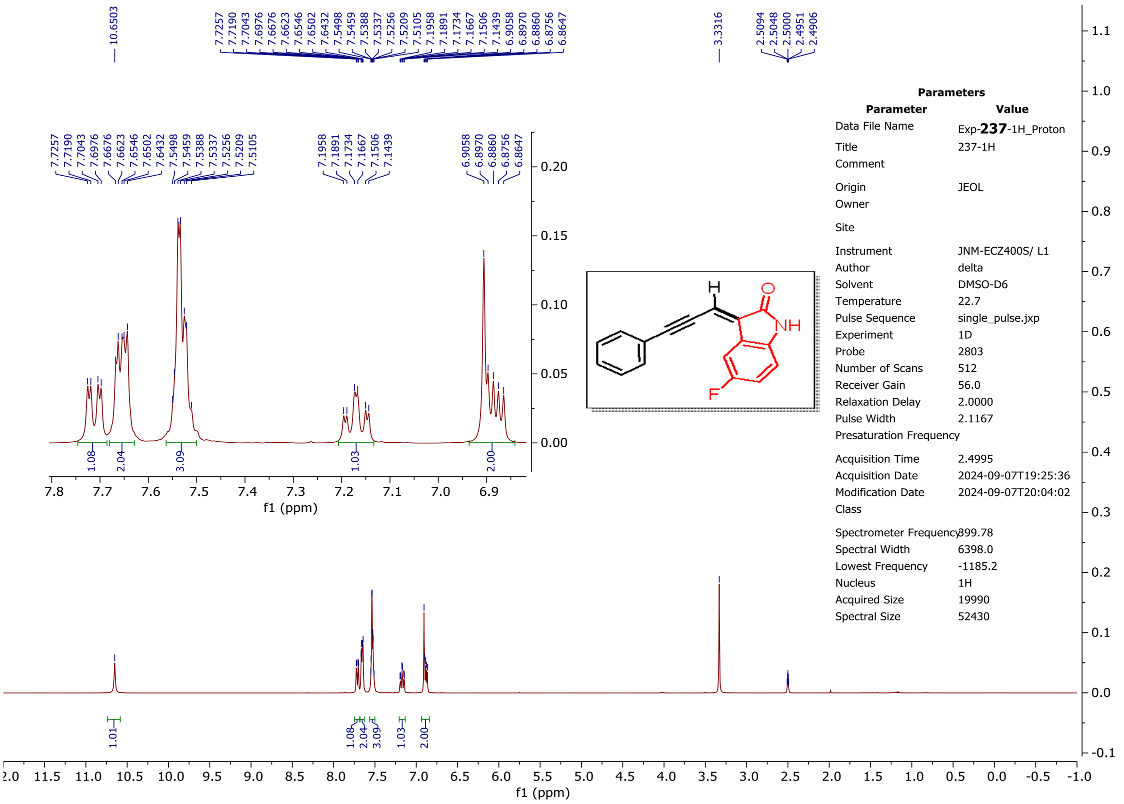

Figure S54. ^{1}H NMR (400 MHz, DMSO-d_6) spectra of compound (4y)

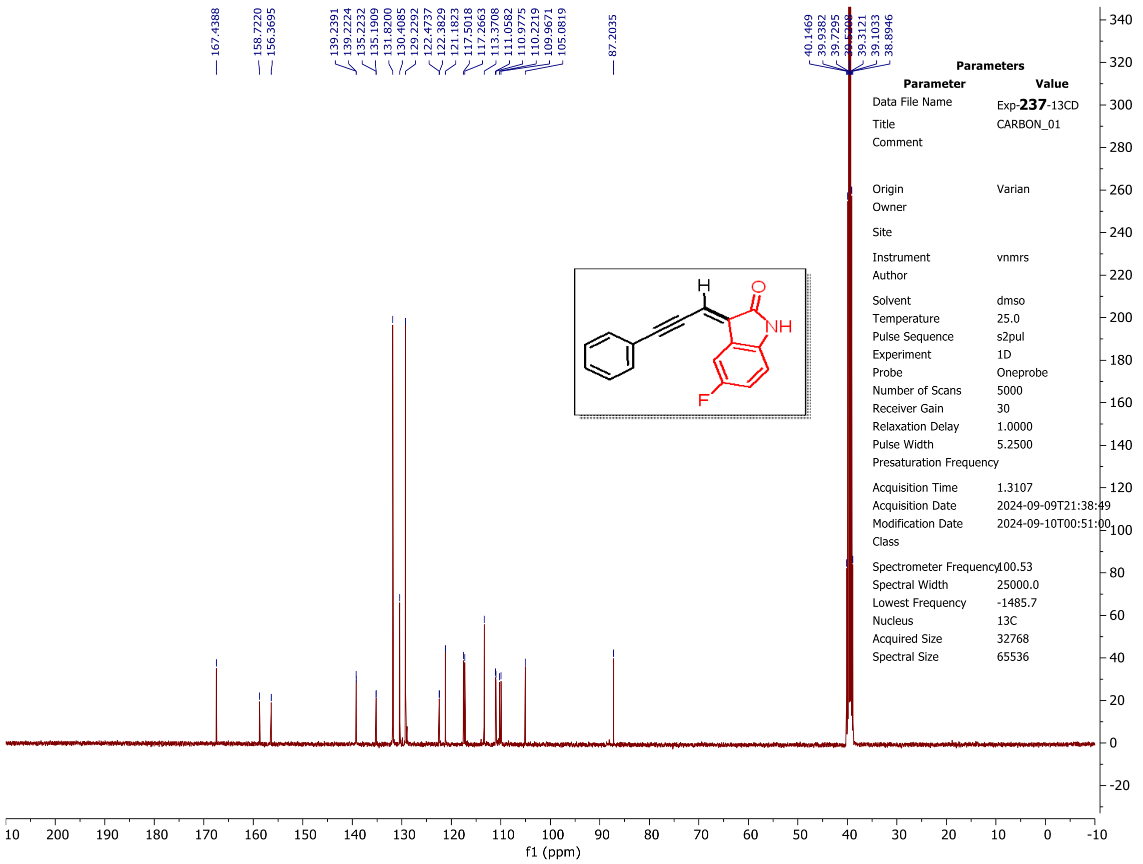

Figure S55. ^{13}**C NMR (100 MHz, DMSO-*d*$_6$) spectra of compound (4y)**

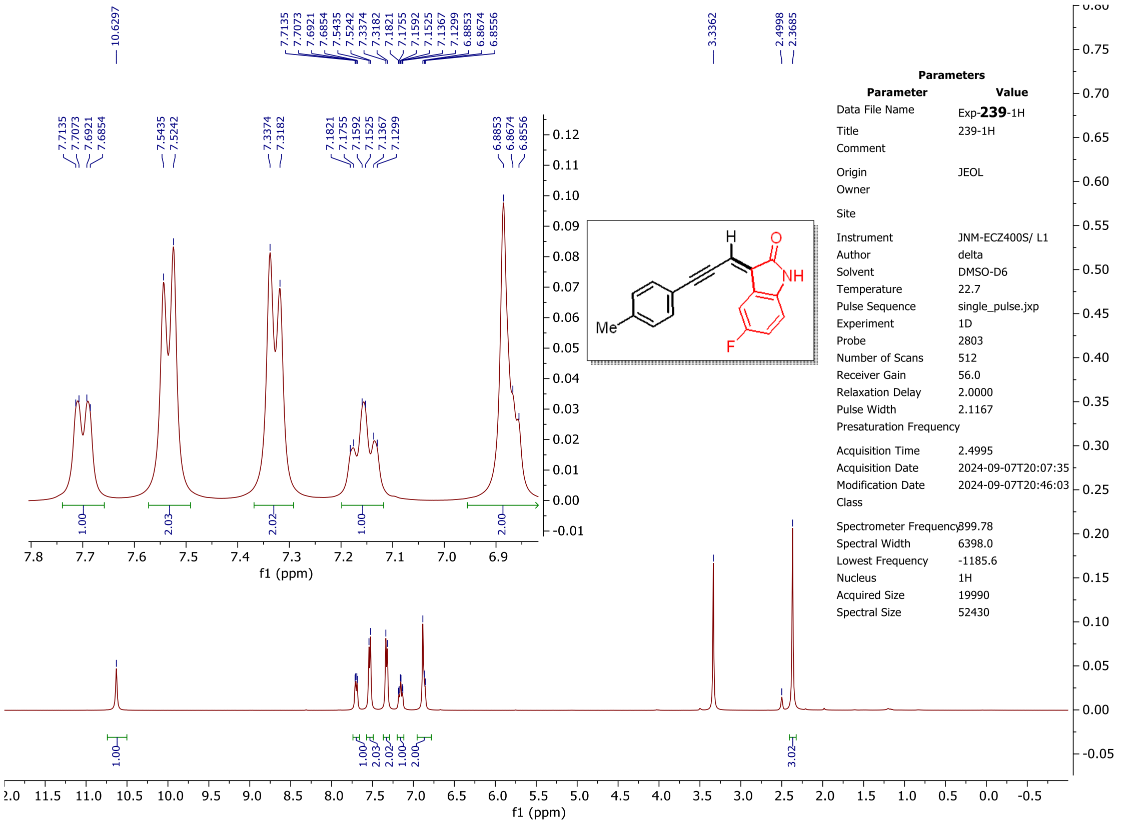

Figure S56. ^{1}**H NMR (400 MHz, DMSO-*d*$_6$) spectra of compound (4z)**

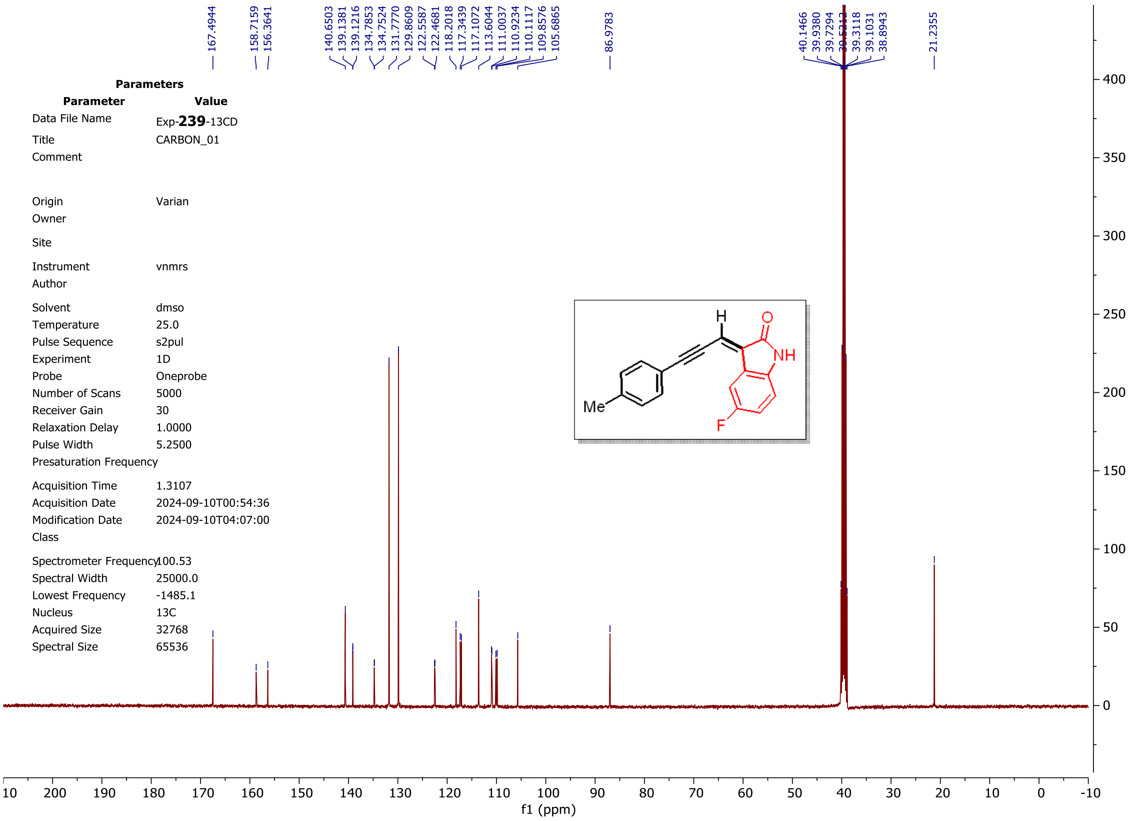

Figure S57. ^{13}**C NMR (100 MHz, DMSO-*d*$_6$) spectra of compound (4z)**

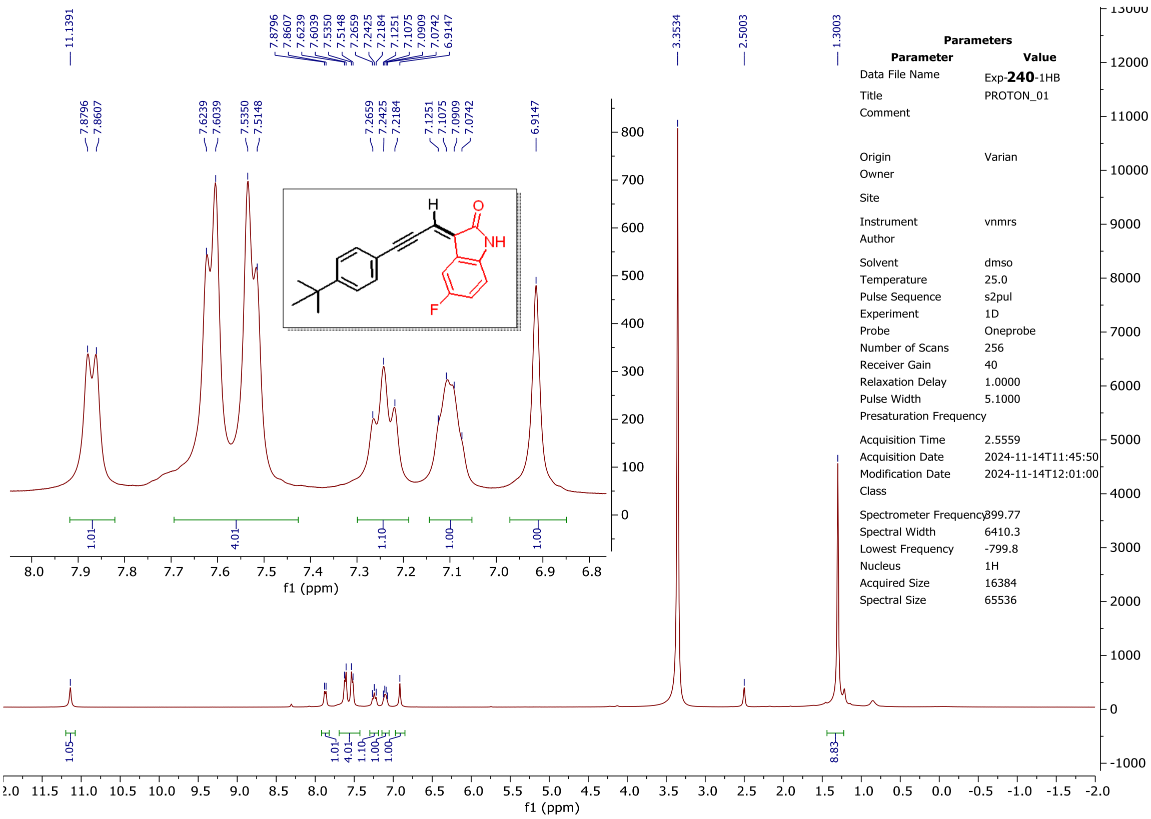

Figure S58. ^{1}**H NMR (400 MHz, DMSO-*d*$_6$) spectra of compound (4aa)**

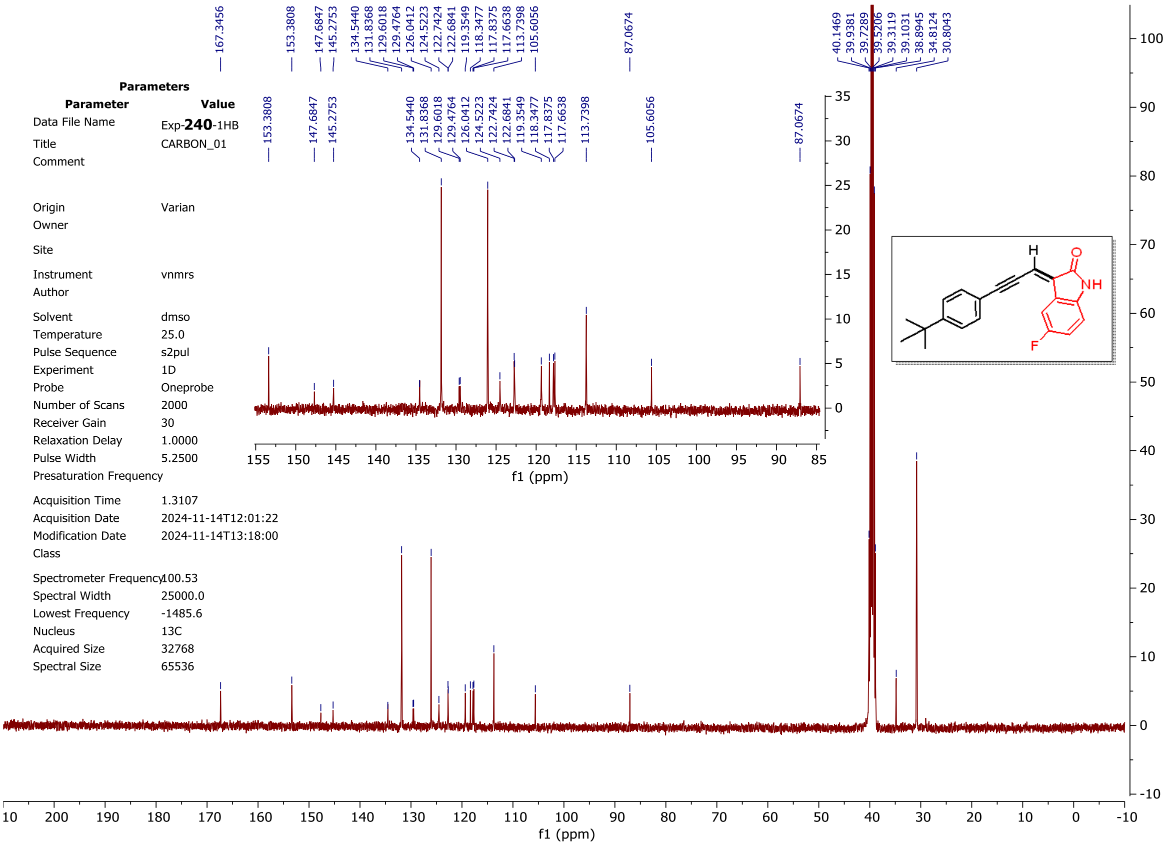

Figure S59. ^{13}C NMR (100 MHz, DMSO-d_6) spectra of compound (4aa)

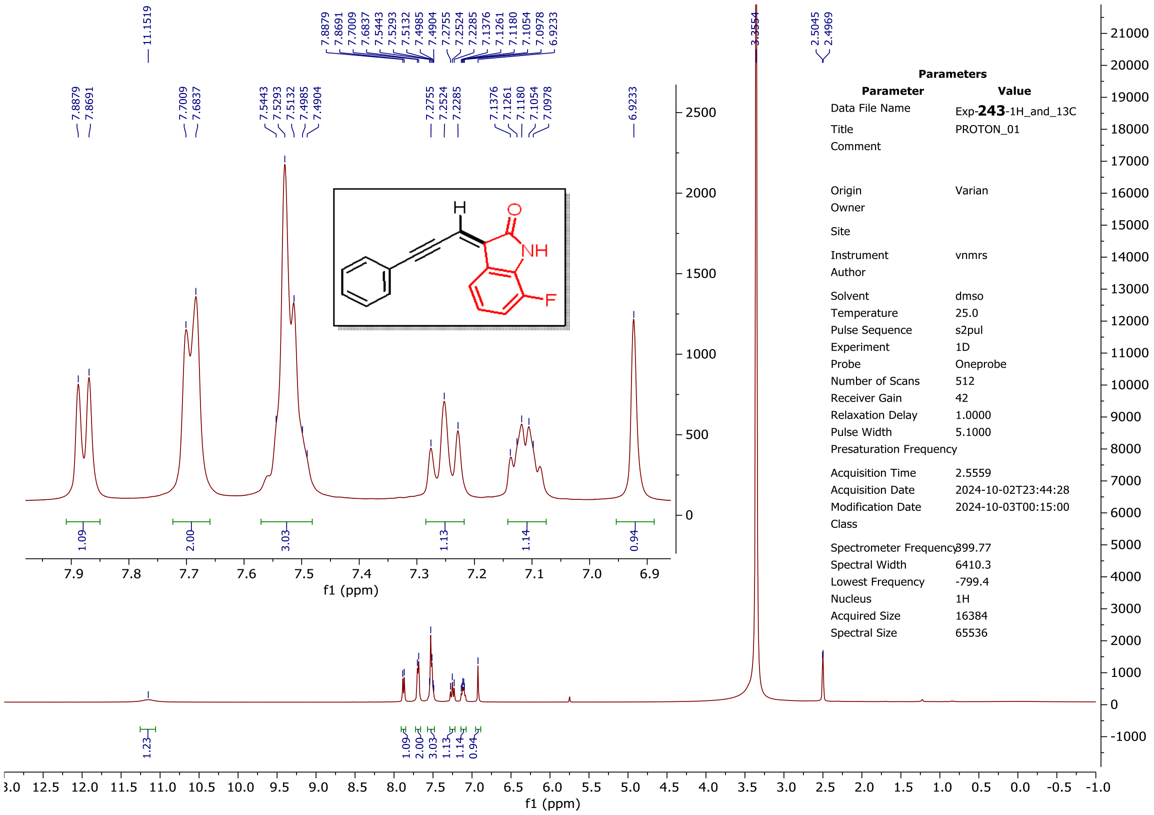

Figure S60. ^{1}H NMR (400 MHz, DMSO-d_6) spectra of compound (4ab)

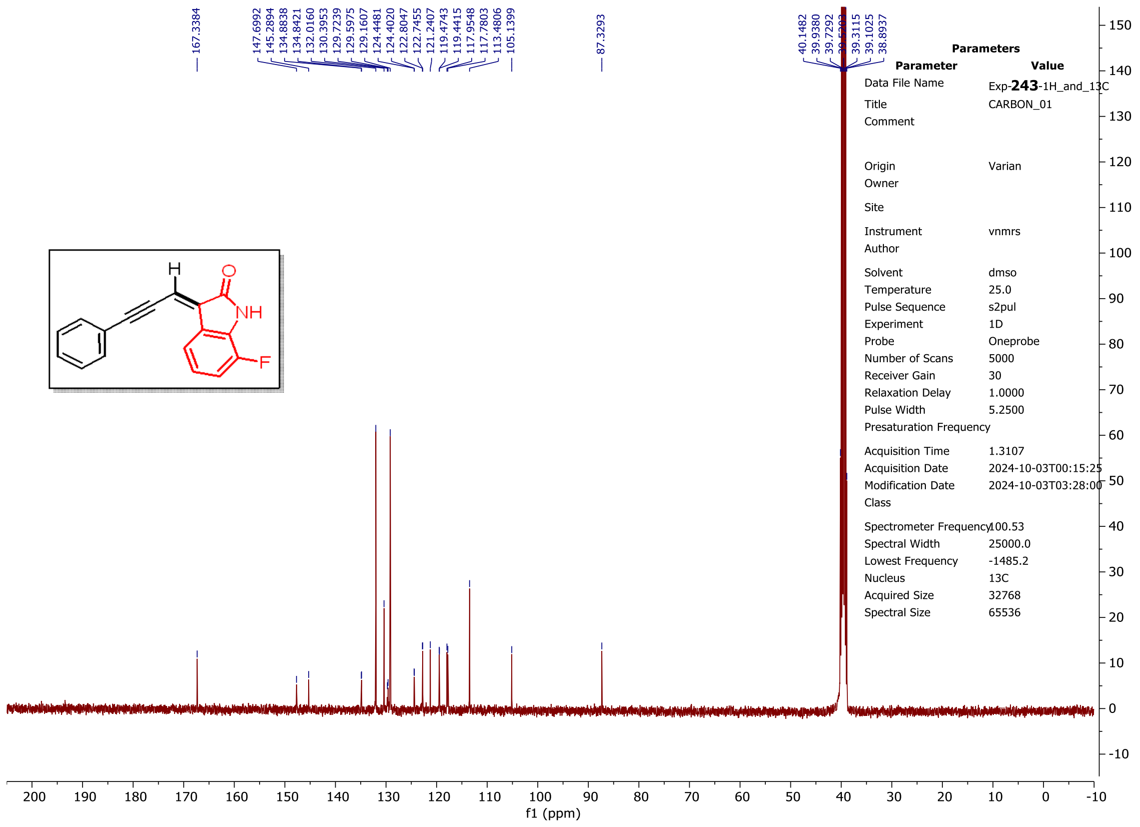

Figure S61. ^{13}C **NMR (100 MHz, DMSO-*d*$_6$) spectra of compound (4ab)**

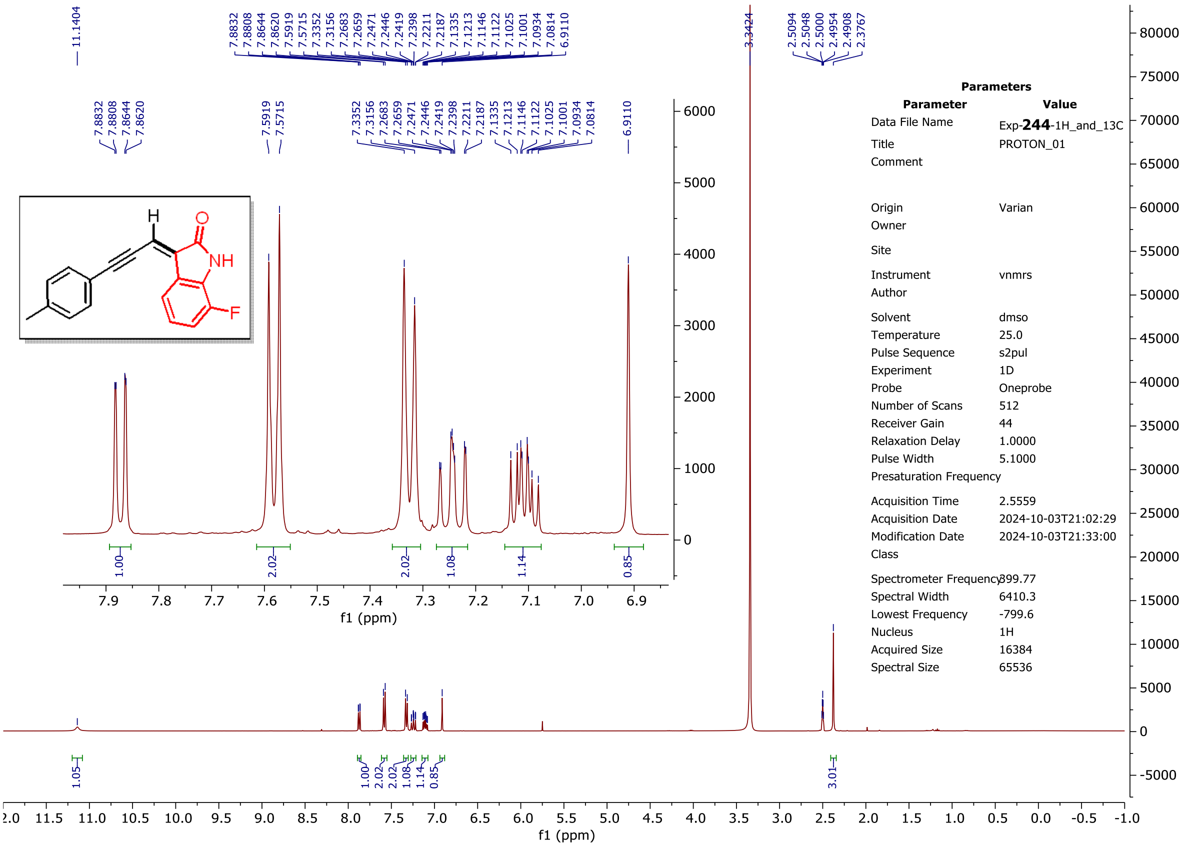

Figure S62. ^{1}H **NMR (400 MHz, DMSO-*d*$_6$) spectra of compound (4ac)**

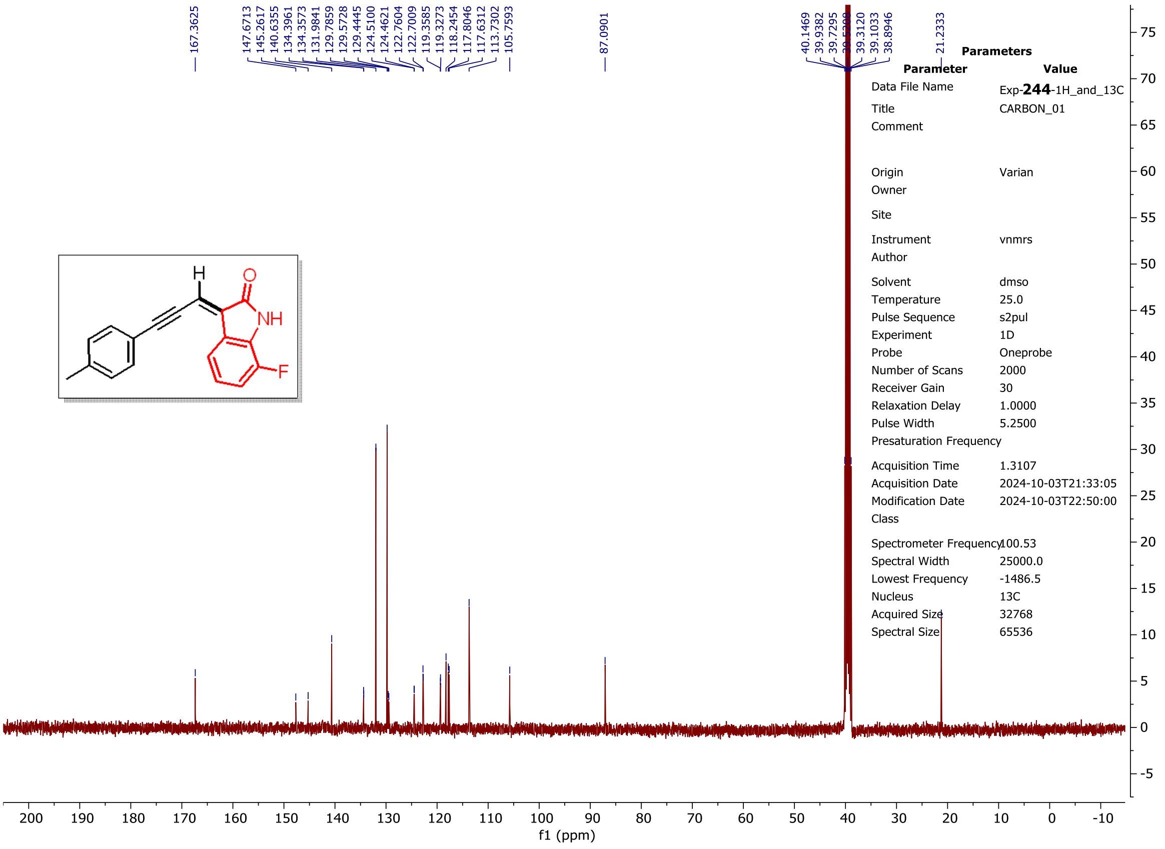

Figure S63. ^{13}C NMR (100 MHz, DMSO-d_6) spectra of compound (4ac)

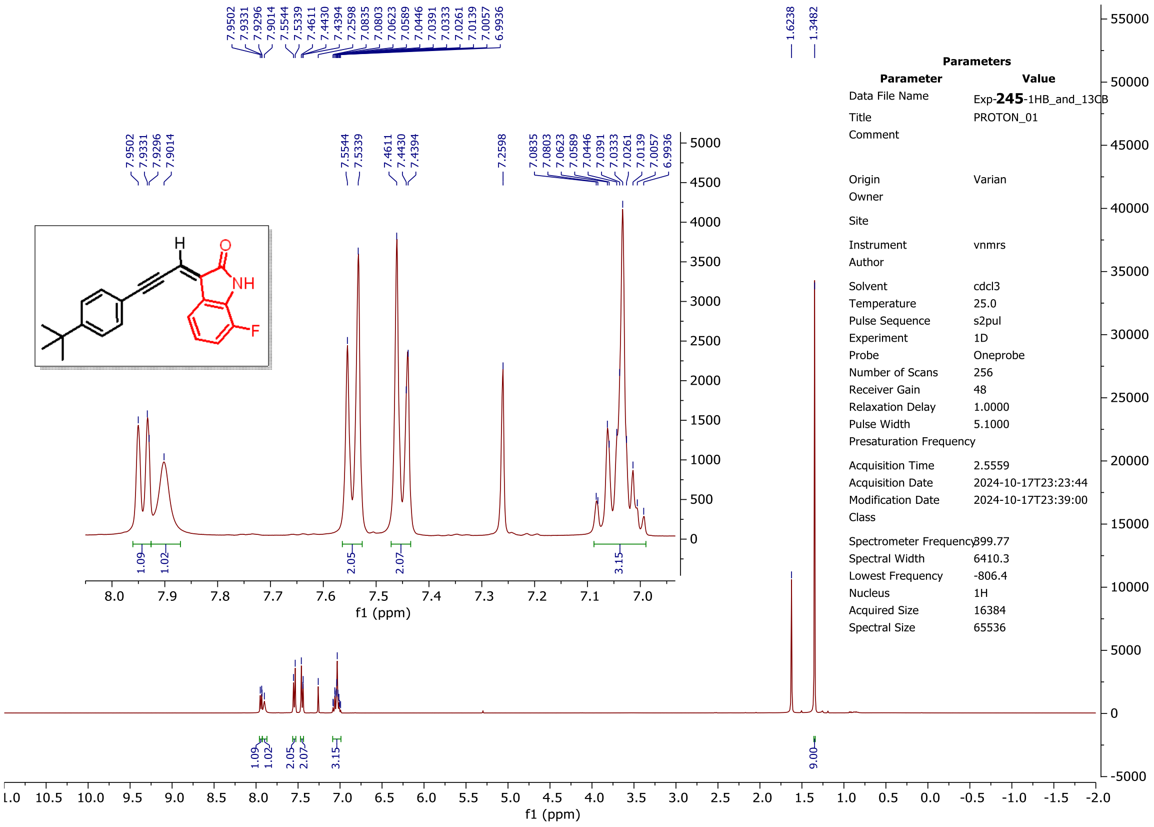

Figure S64. ^{1}H NMR (400 MHz, $CDCl_3$) spectra of compound (4ad)

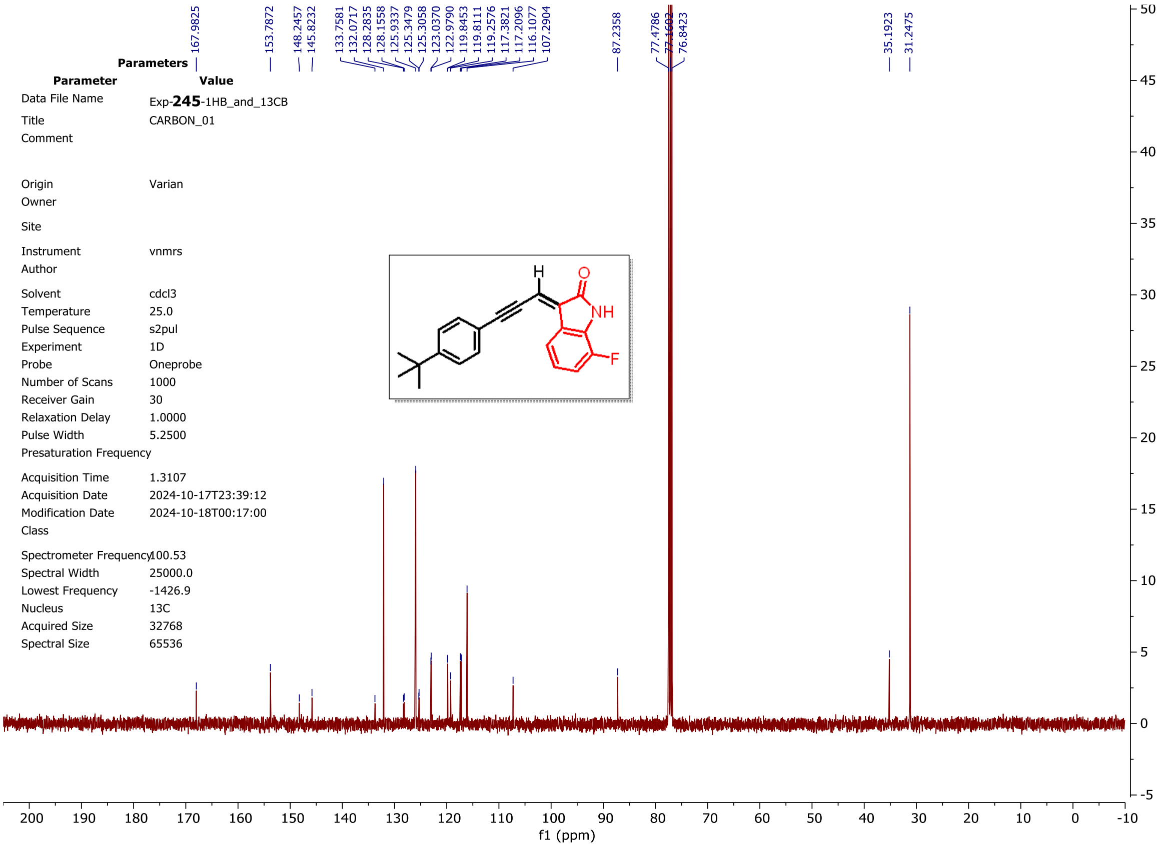

Figure S65. ^{13}C NMR (100 MHz, $CDCl_3$) spectra of compound (4ad)

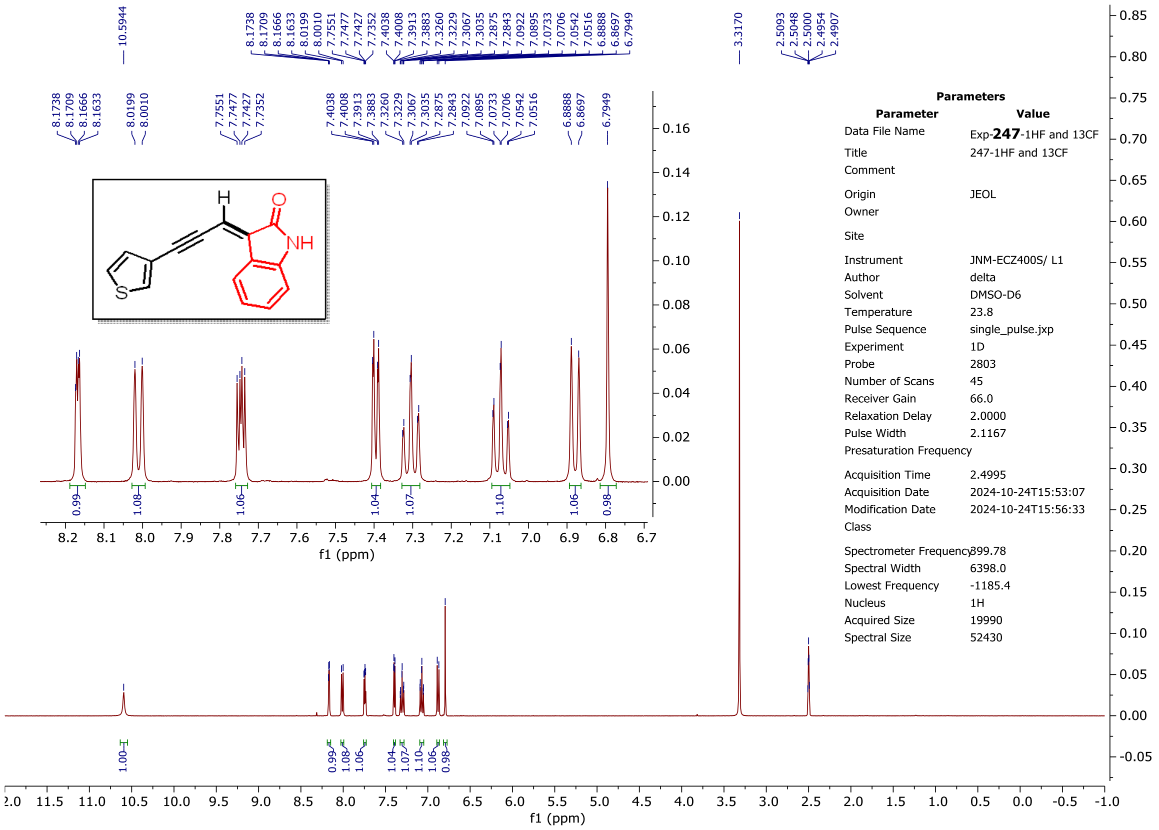

Figure S66. ^{1}H NMR (400 MHz, DMSO-d_6) spectra of compound (4ae)

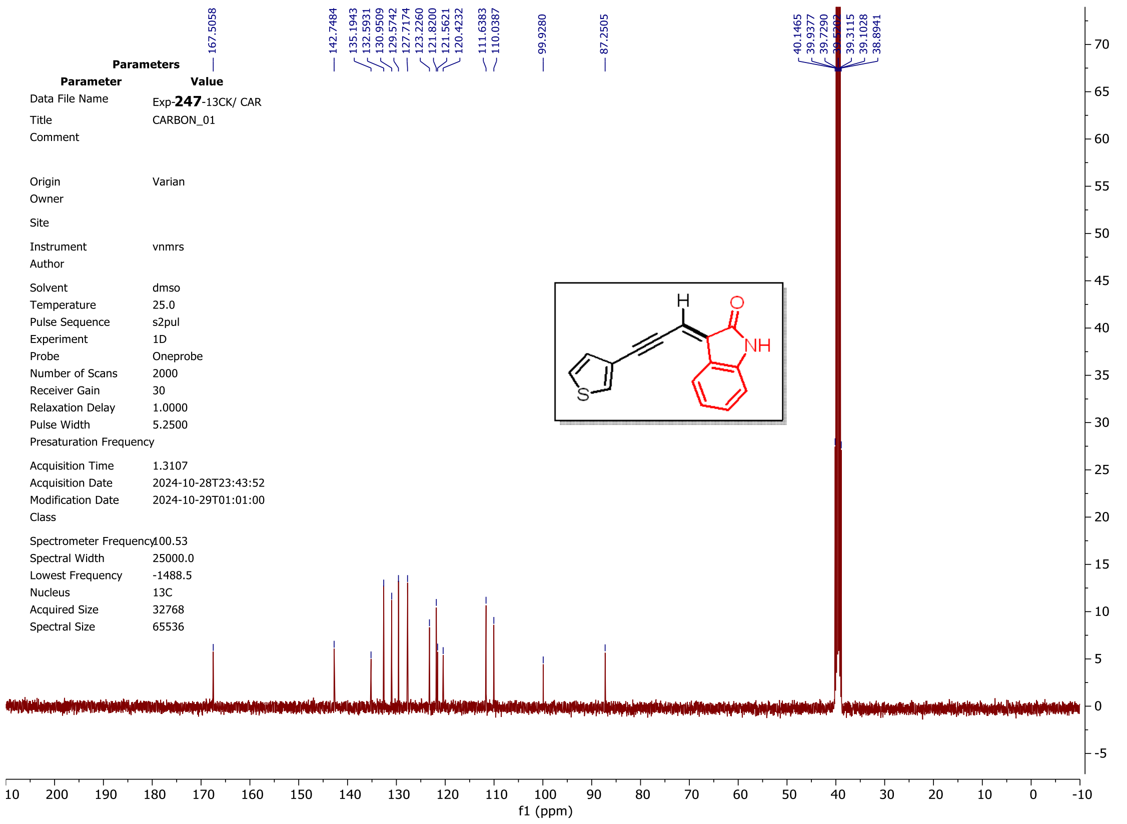

Figure S67. ^{13}C NMR (100 MHz, DMSO-d_6) spectra of compound (4ae)

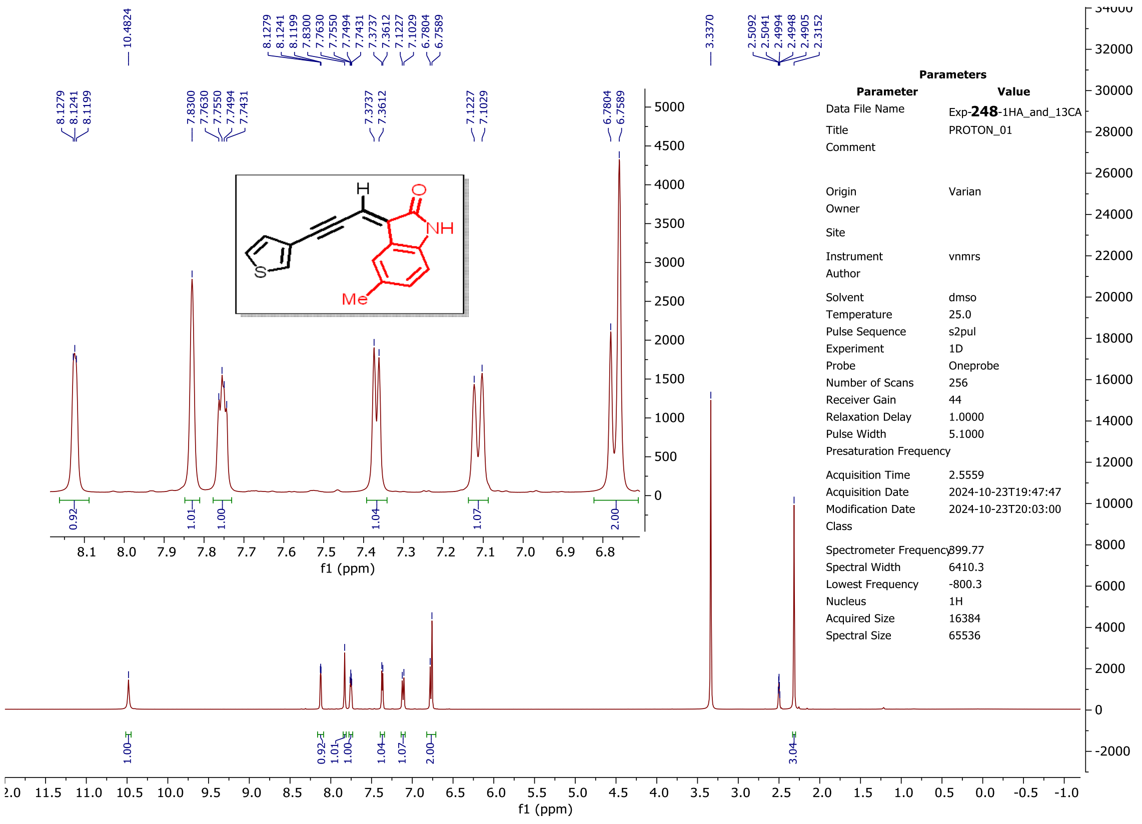

Figure S68. ^{1}H NMR (400 MHz, DMSO-d_6) spectra of compound (4af)

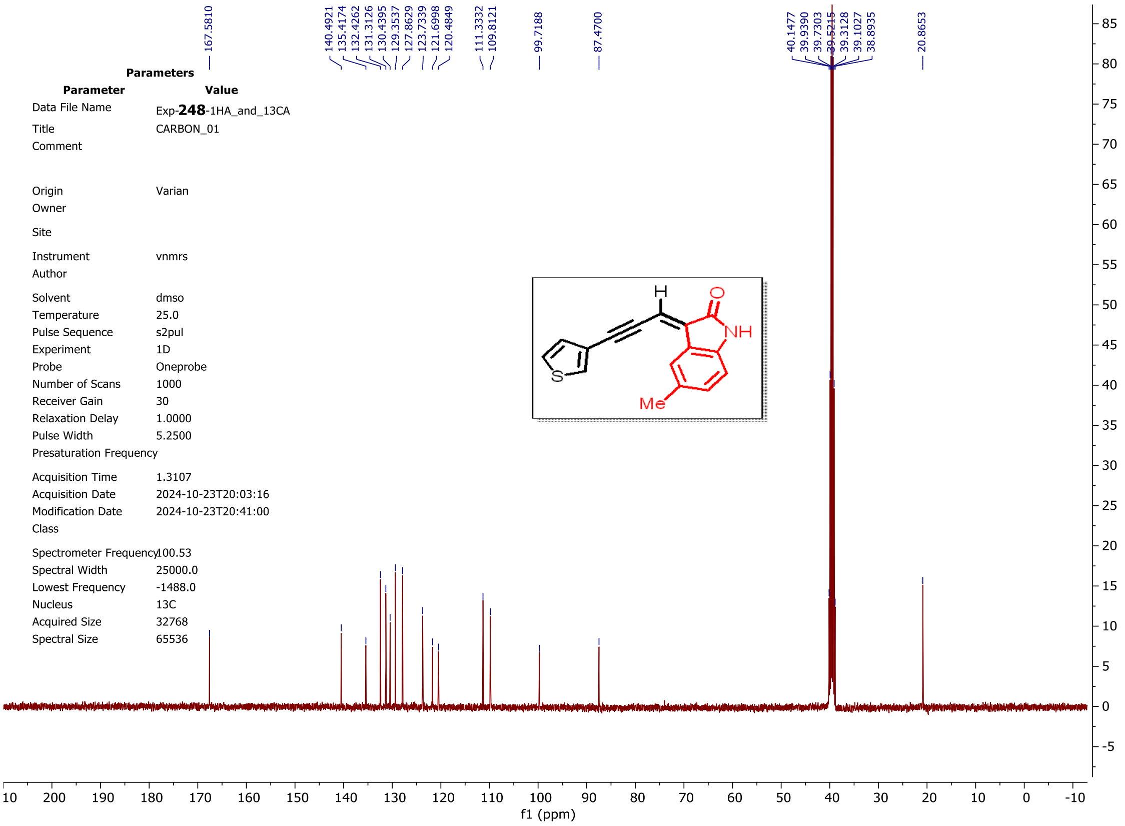

Figure S69. ^{13}C **NMR (100 MHz, DMSO-d_6) spectra of compound (4af)**

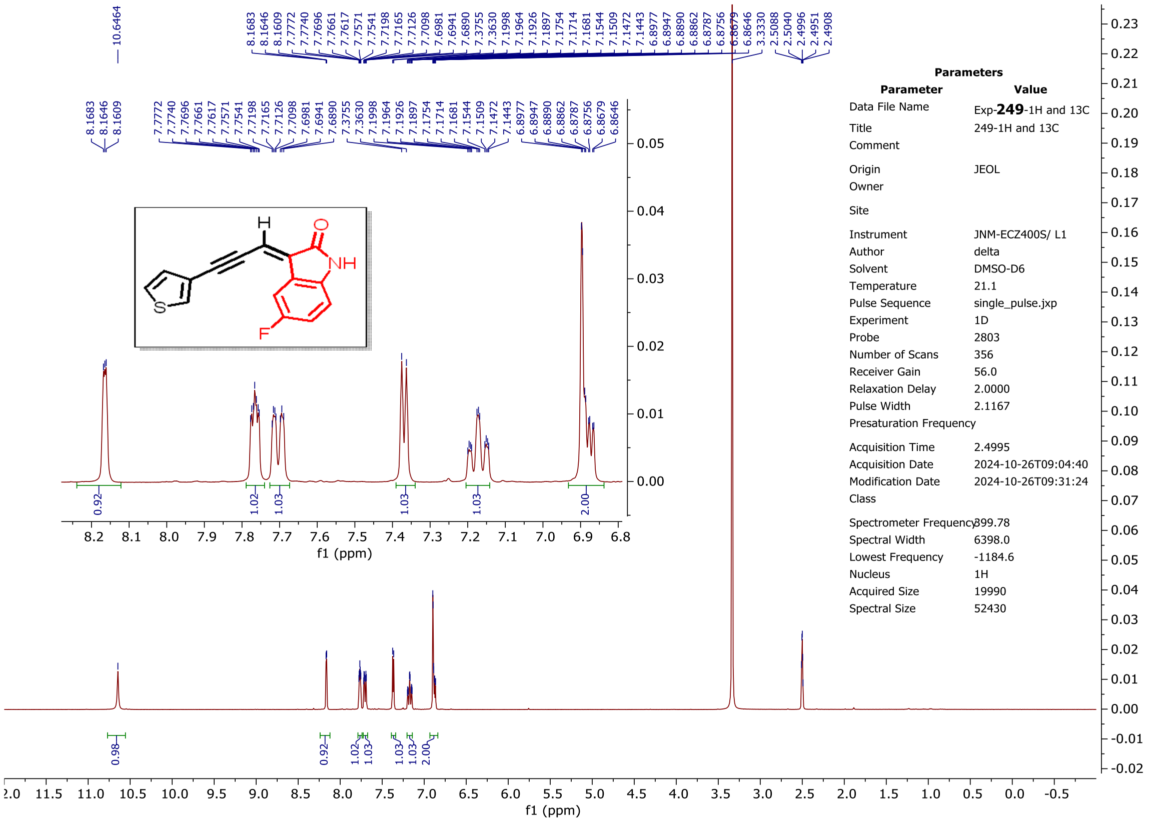

Figure S70. ^{1}H **NMR (400 MHz, DMSO-d_6) spectra of compound (4ag)**

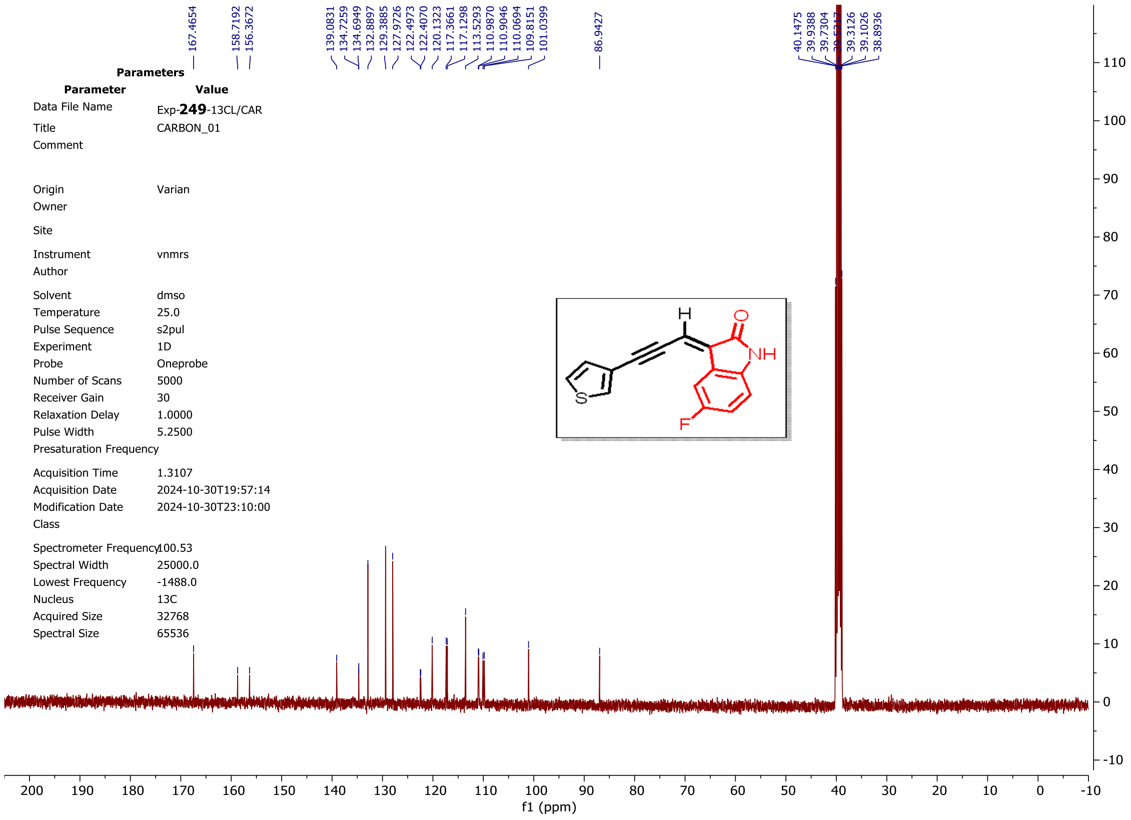

Figure S71. ^{13}C **NMR (100 MHz, DMSO-d_6) spectra of compound (4ag)**

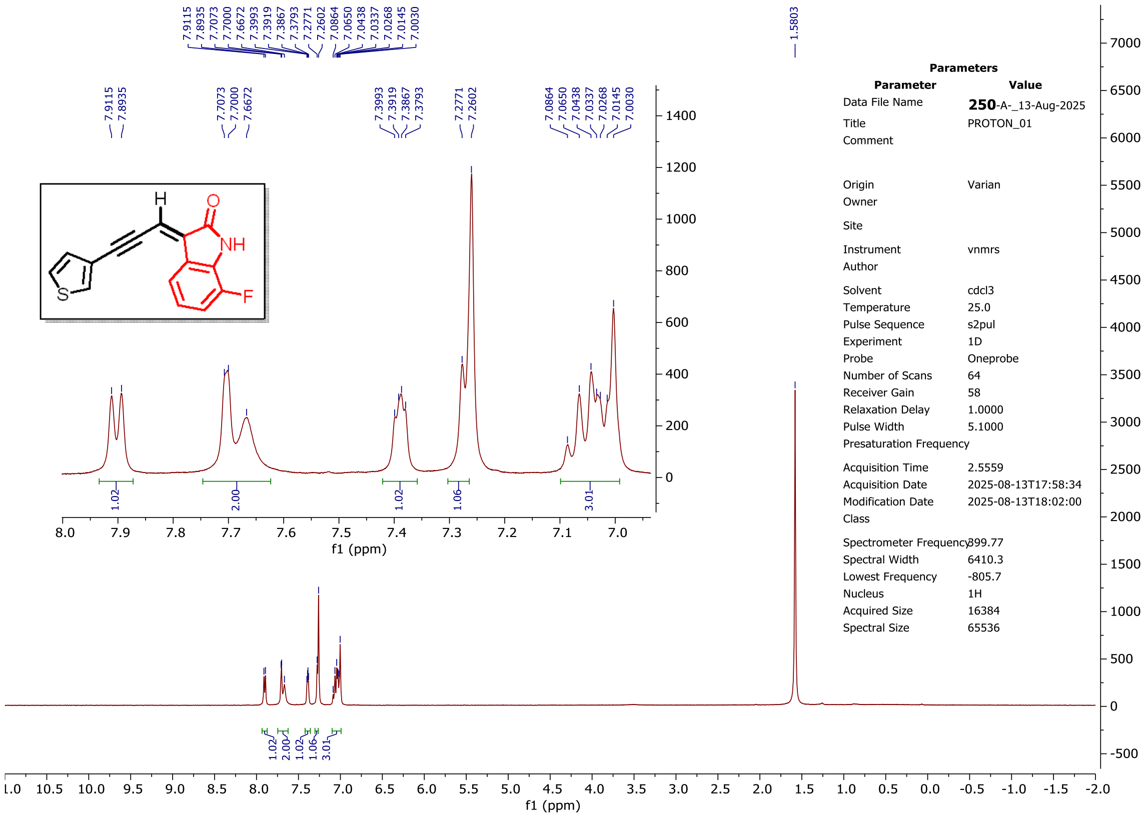

Figure S72. ^{1}H **NMR (400 MHz, $CDCl_3$) spectra of compound (4ah)**

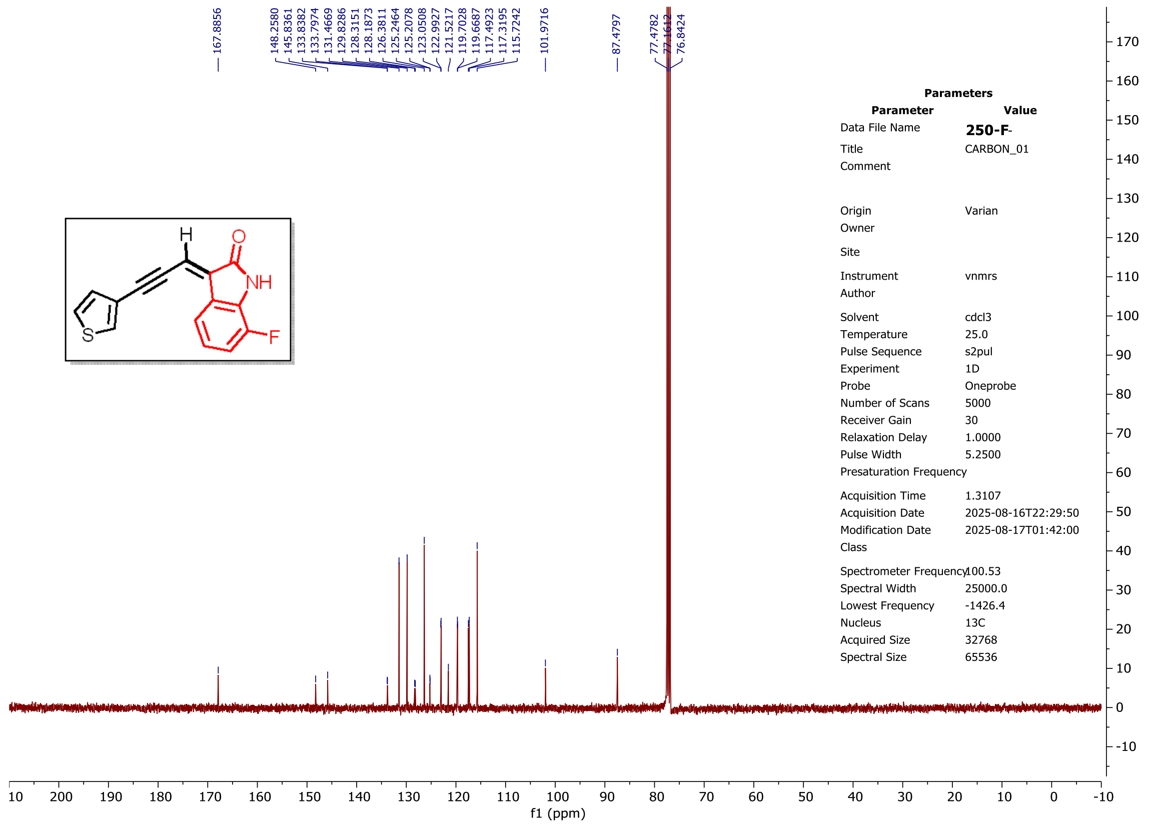

Figure S73. ^{13}C NMR (100 MHz, CDCl$_3$) spectra of compound (4ah)

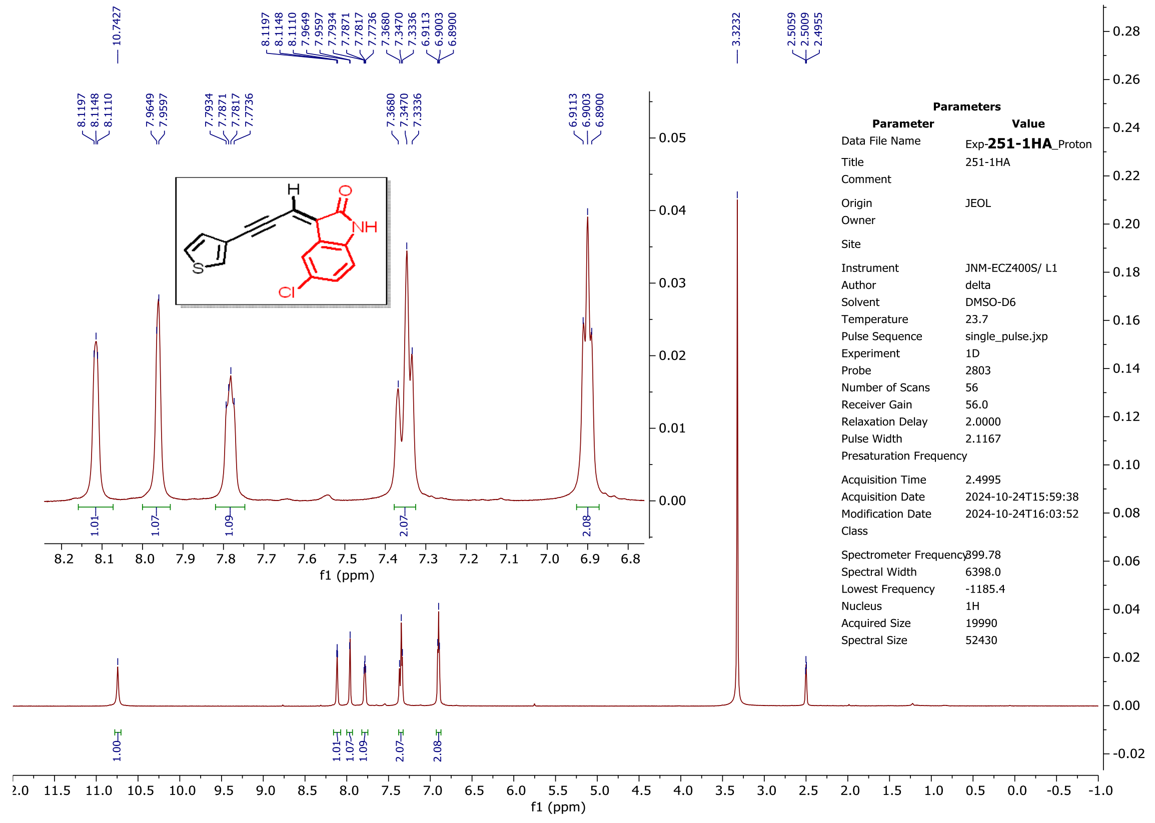

Figure S74. ^{1}H NMR (400 MHz, DMSO-d_6) spectra of compound (4ai)

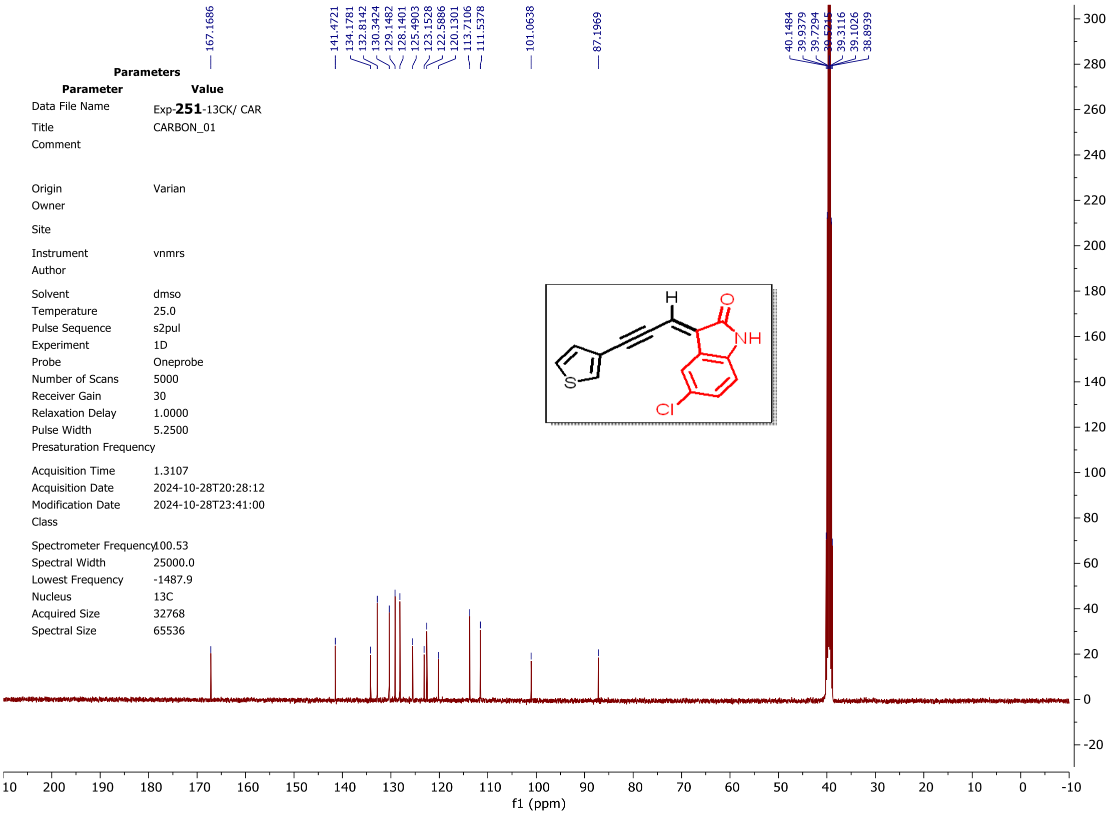

Figure S75. ^{13}C NMR (100 MHz, DMSO-d_6) spectra of compound (4ai)

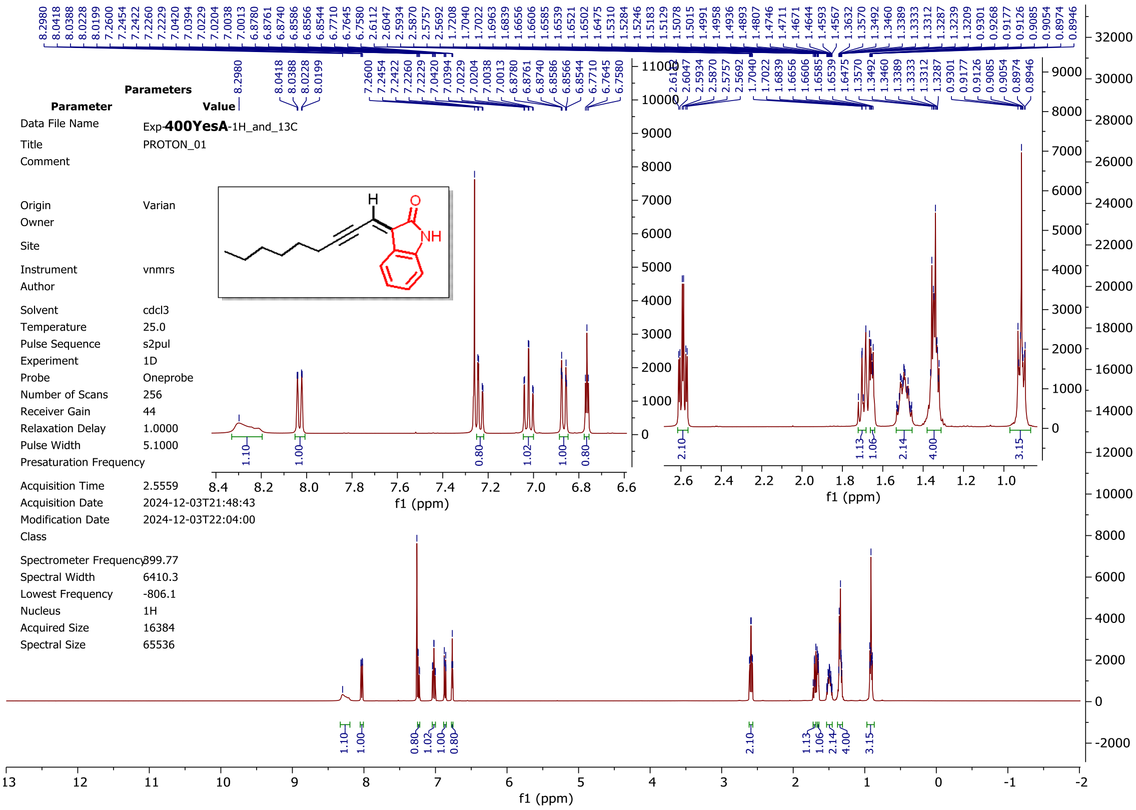

Figure S76. ^{1}H NMR (400 MHz, $CDCl_3$) spectra of compound (4aj)

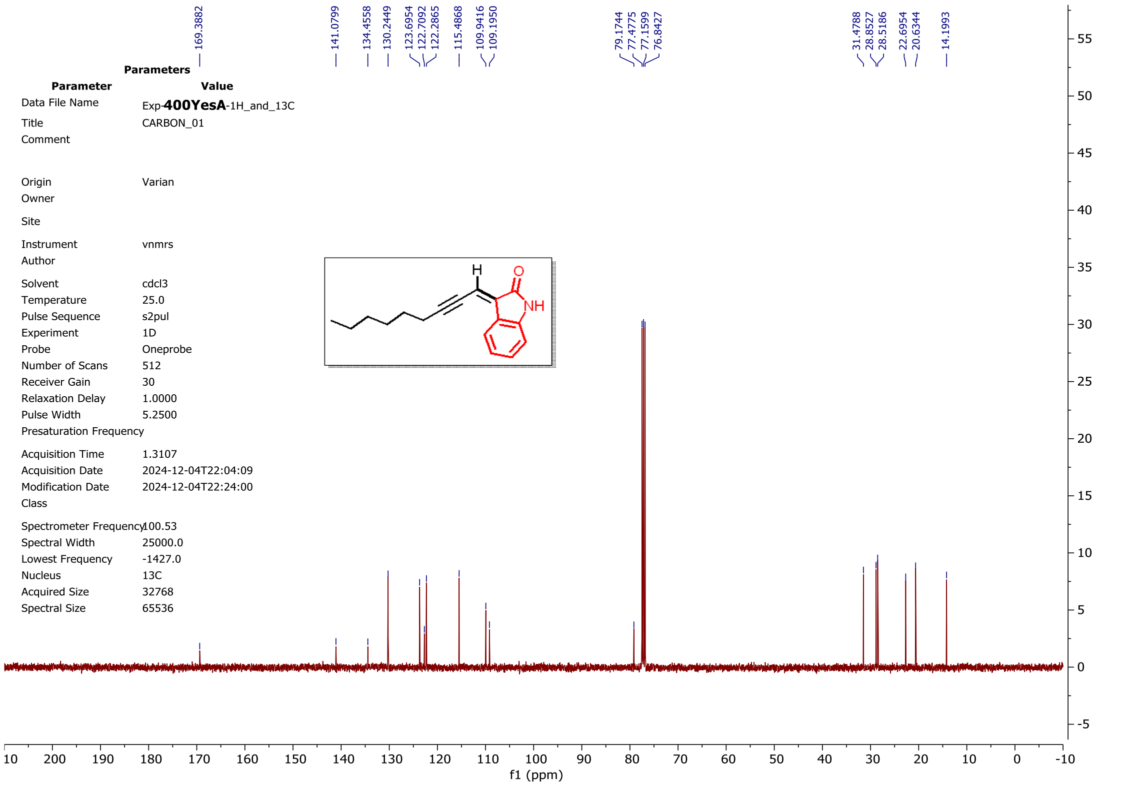

Figure S77. ^{13}**C NMR (100 MHz, CDCl$_3$) spectra of compound (4aj)**

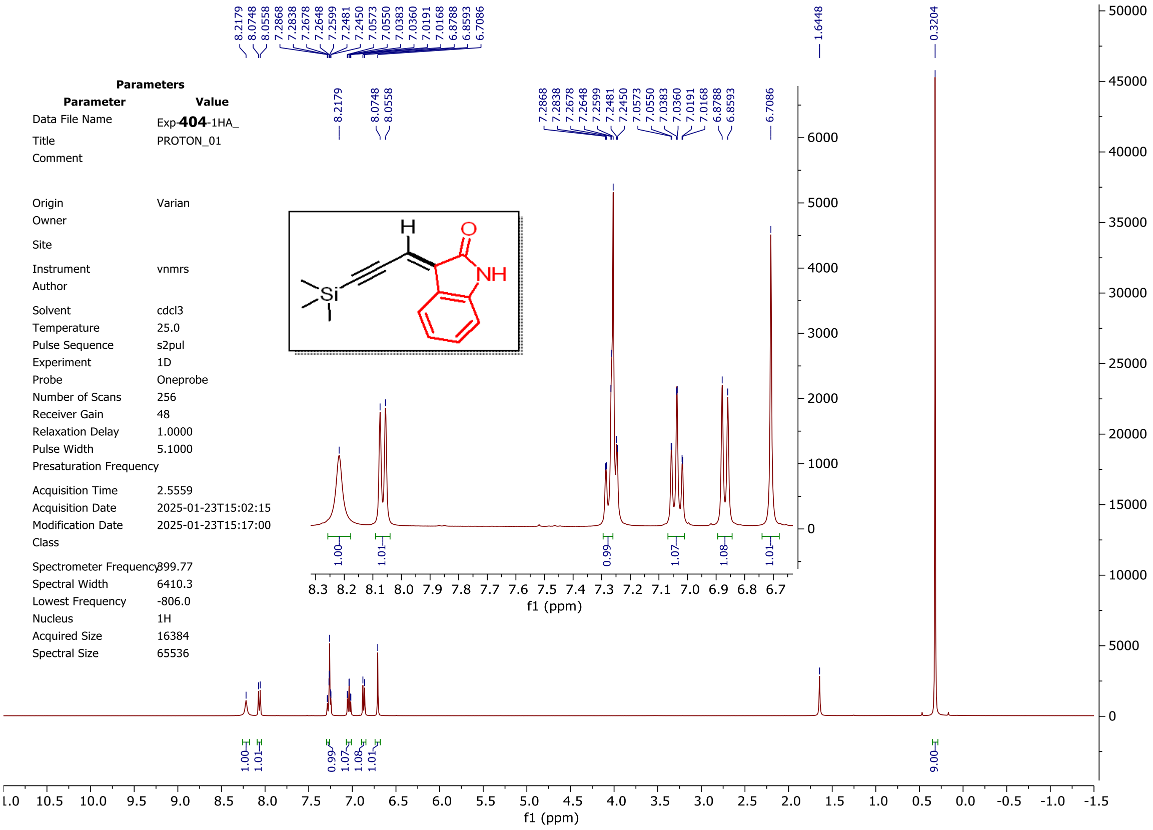

Figure S78. ^{1}**H NMR (400 MHz, CDCl$_3$) spectra of compound (4ak)**

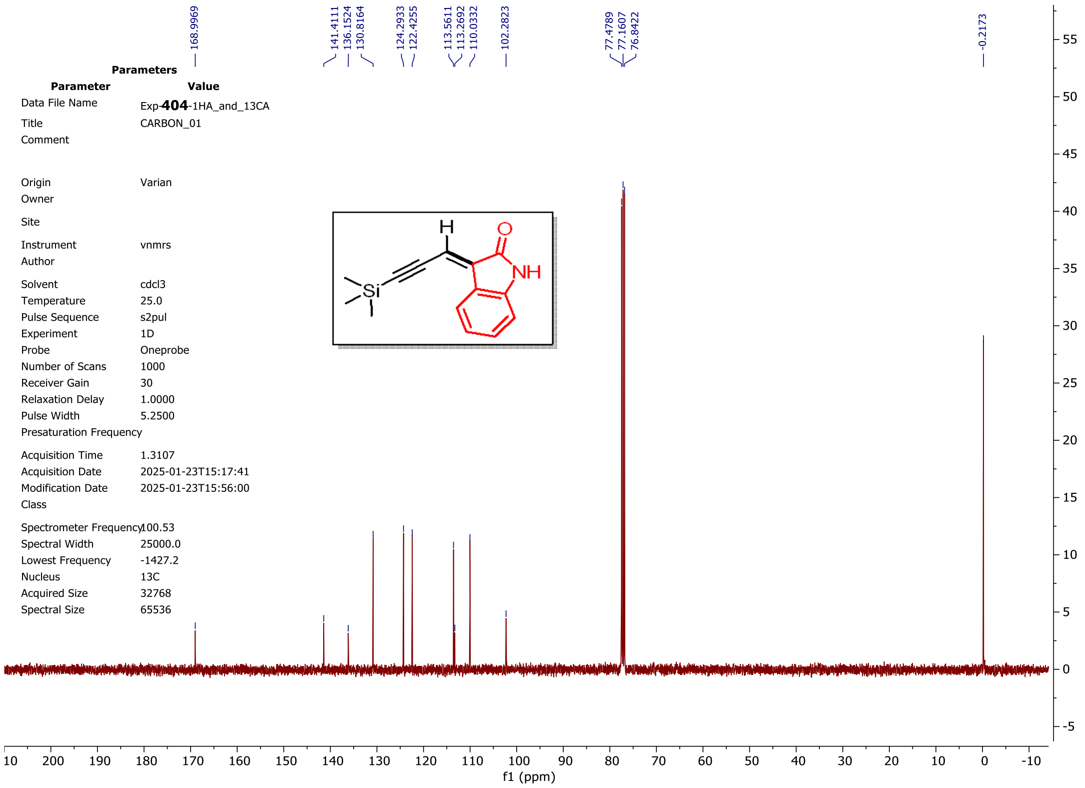

Figure S79. ^{13}C **NMR (100 MHz,** $CDCl_3$**) spectra of compound (4ak)**

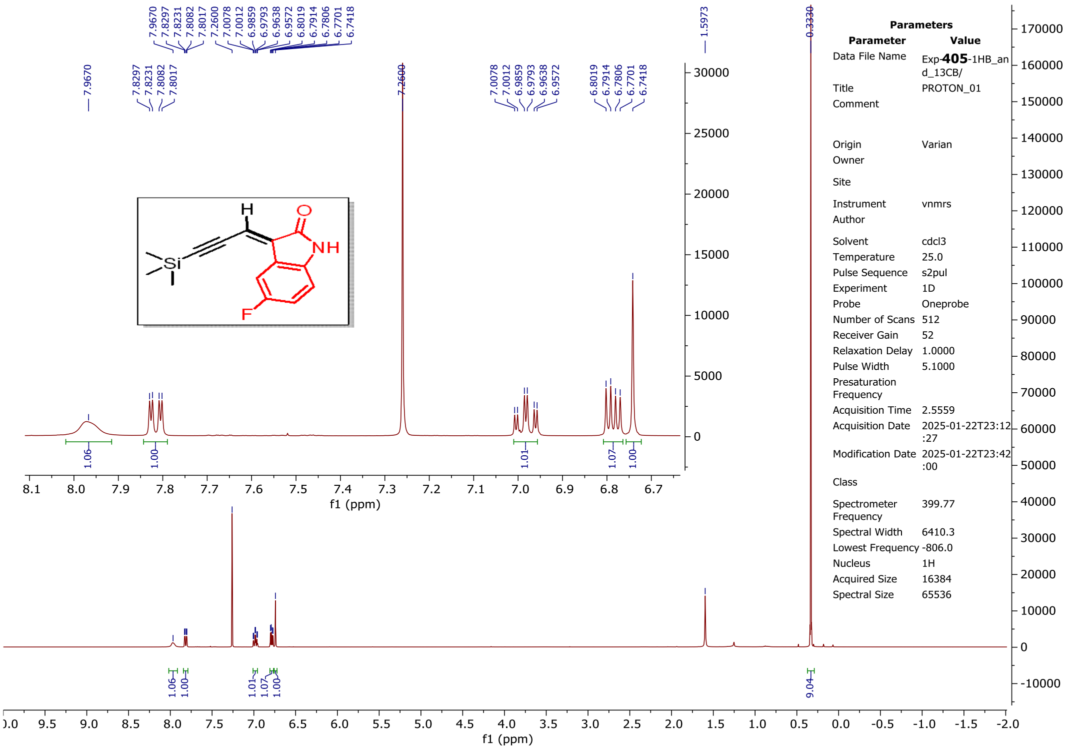

Figure S80. ^{1}H **NMR (400 MHz,** $CDCl_3$**) spectra of compound (4al)**

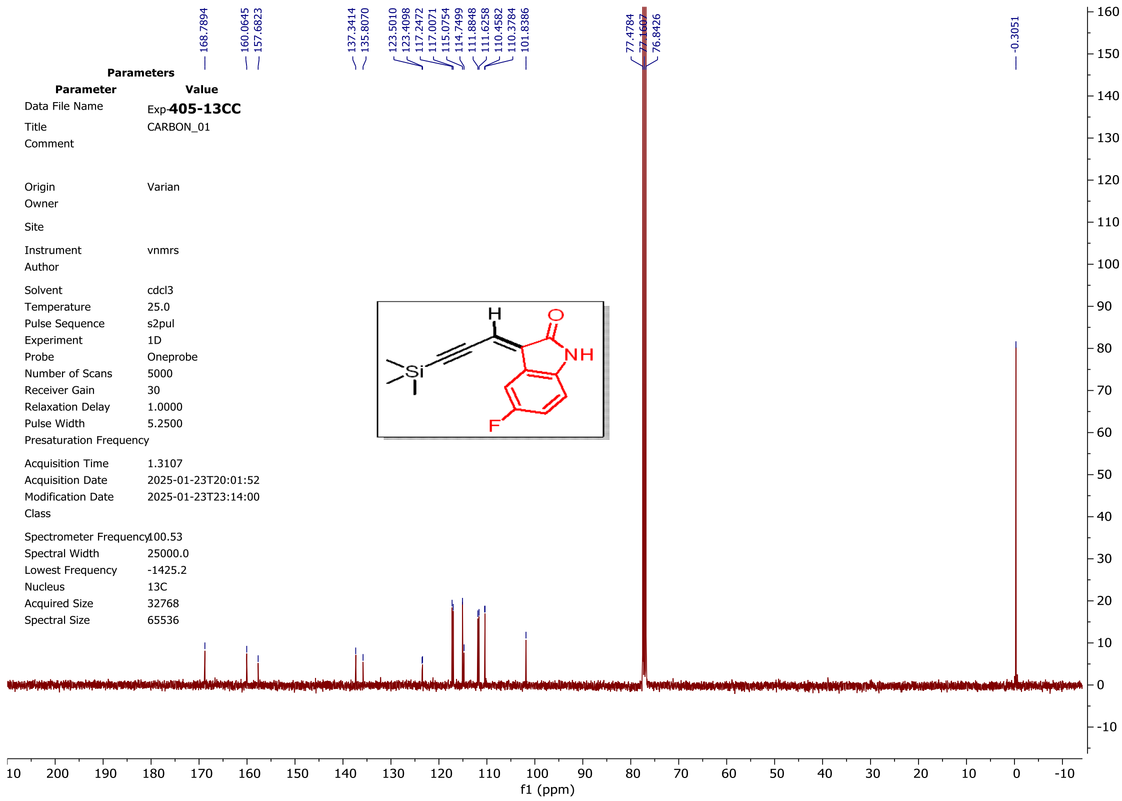

Figure S81. ^{13}C NMR (100 MHz, $CDCl_3$) spectra of compound (4al)

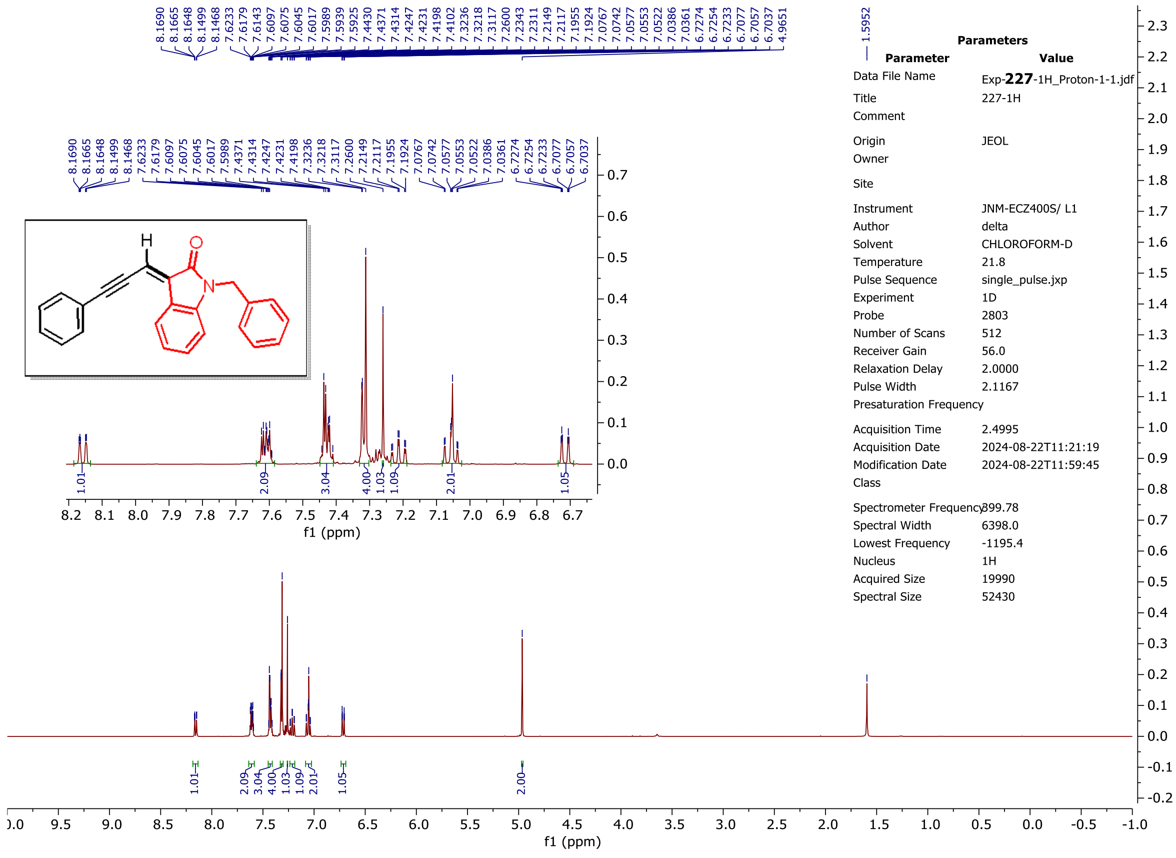

Figure S82. ^{1}H NMR (400 MHz, $CDCl_3$) spectra of compound (5a)

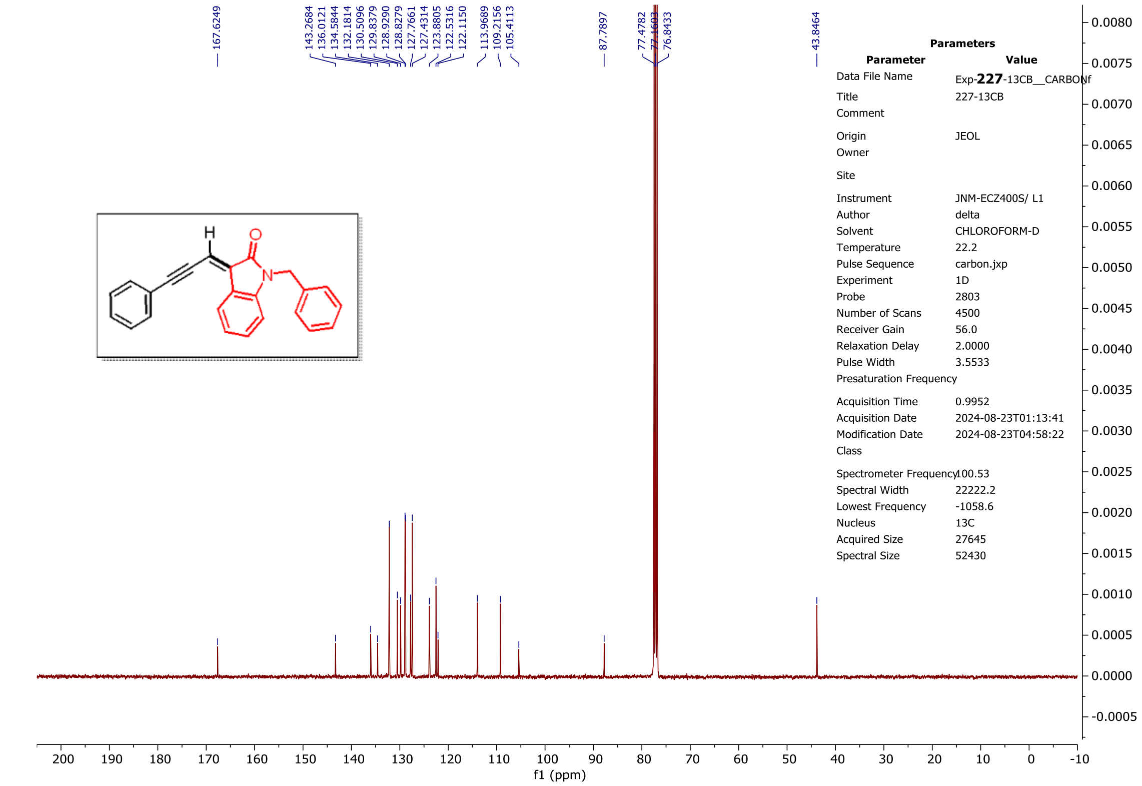

Figure S83. ^{13}C **NMR (100 MHz,** $CDCl_3$**) spectra of compound (5a)**

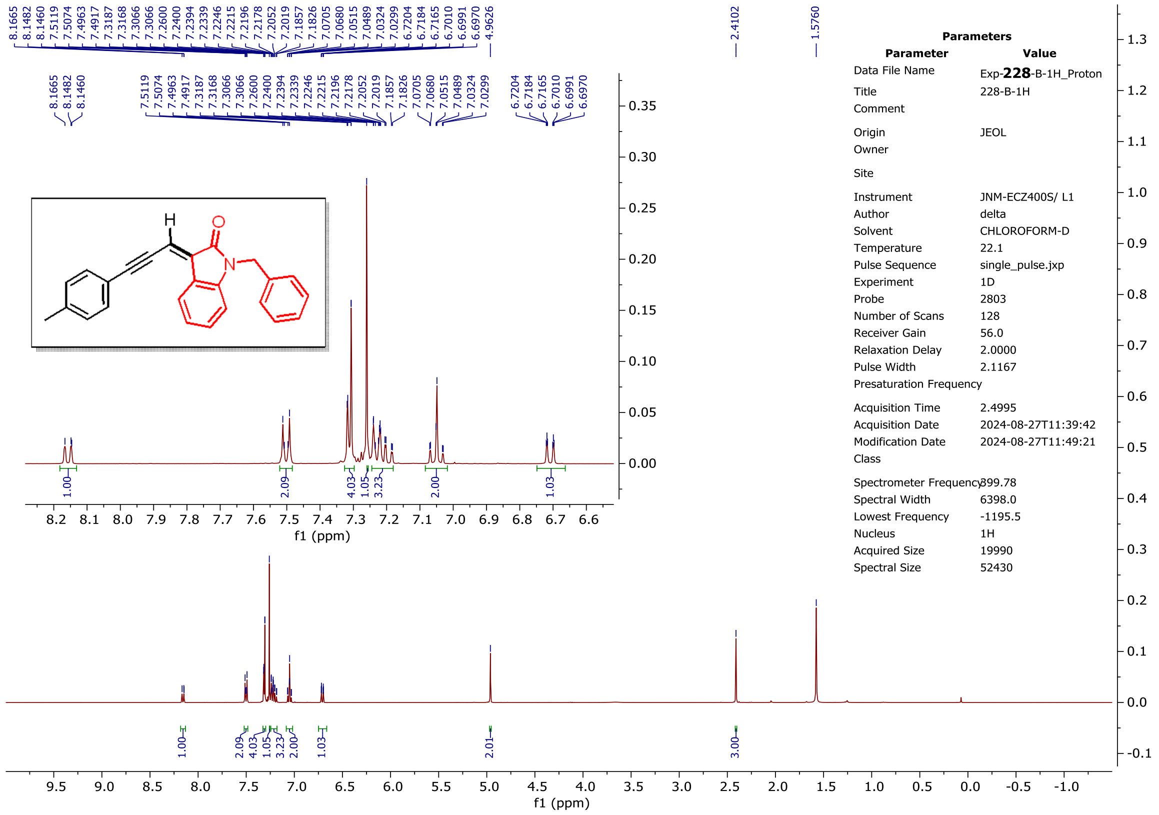

Figure S84. ^{1}H **NMR (400 MHz,** $CDCl_3$**) spectra of compound (5b)**

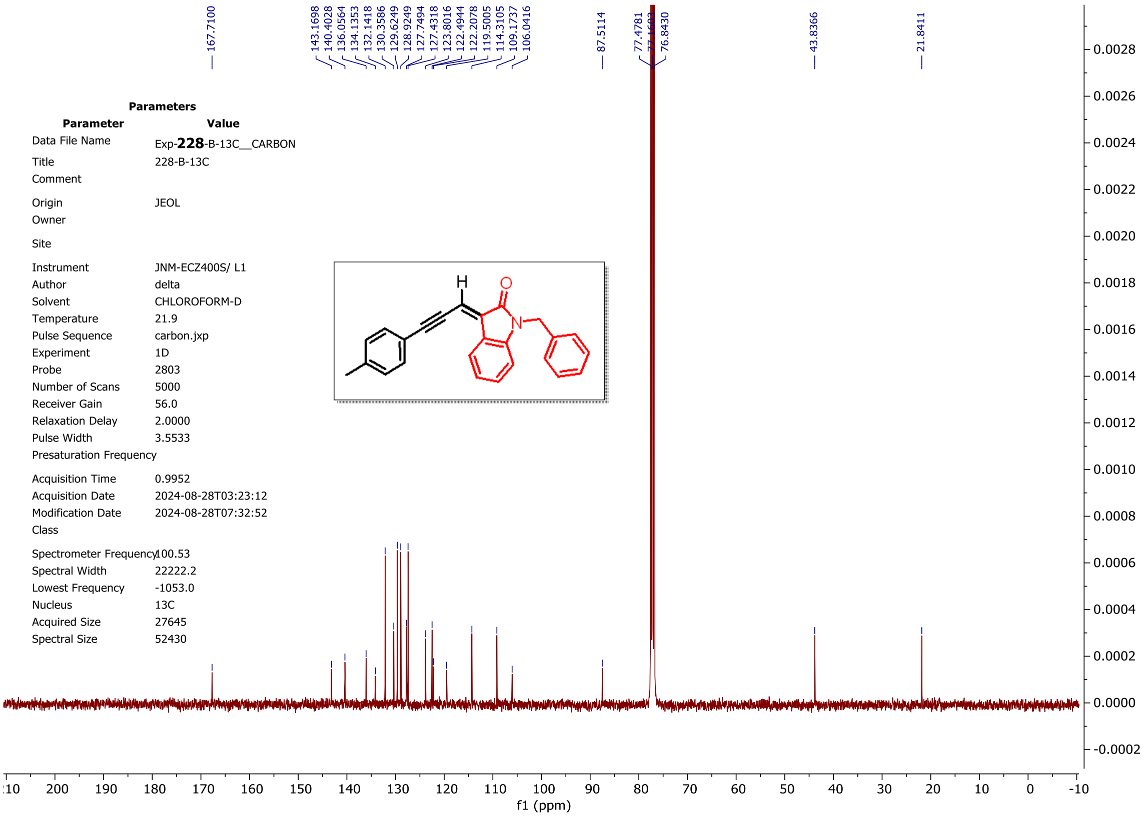

Figure S85. ^{13}C NMR (100 MHz, $CDCl_3$) spectra of compound (5b)

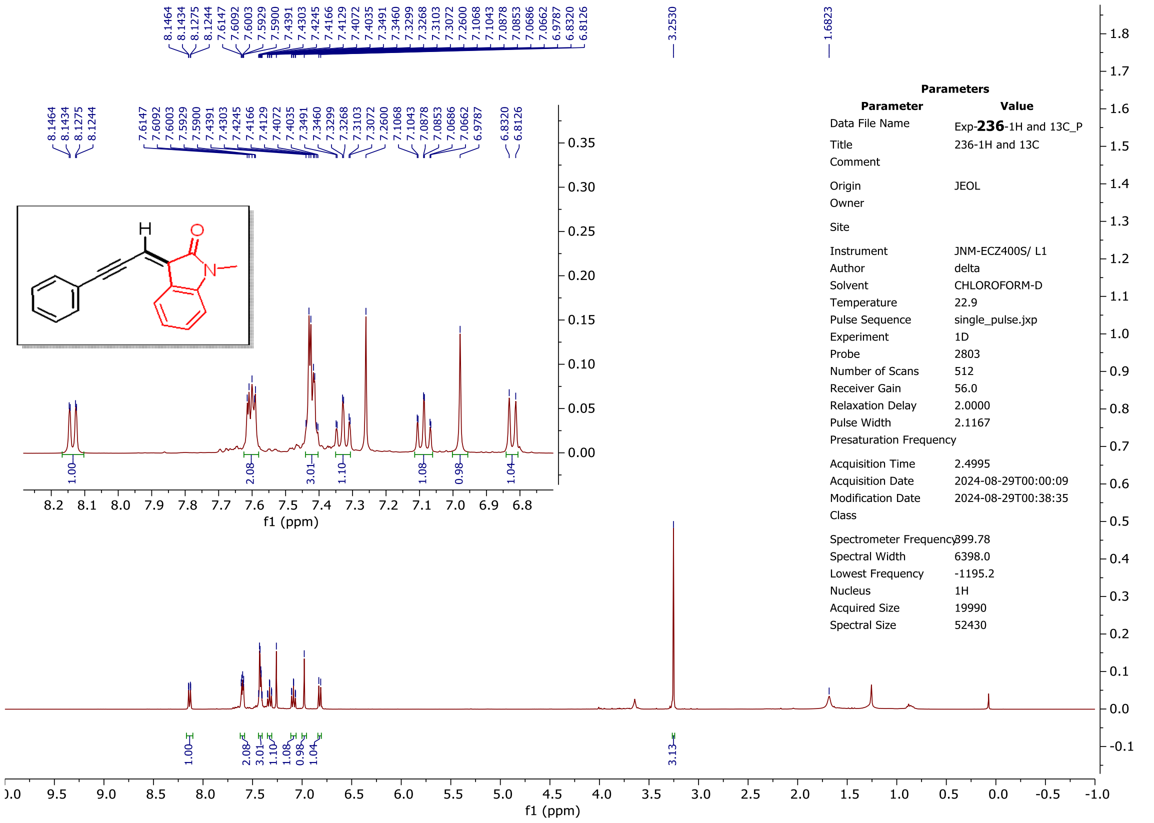

Figure S86. ^{1}H NMR (400 MHz, $CDCl_3$) spectra of compound (5c)

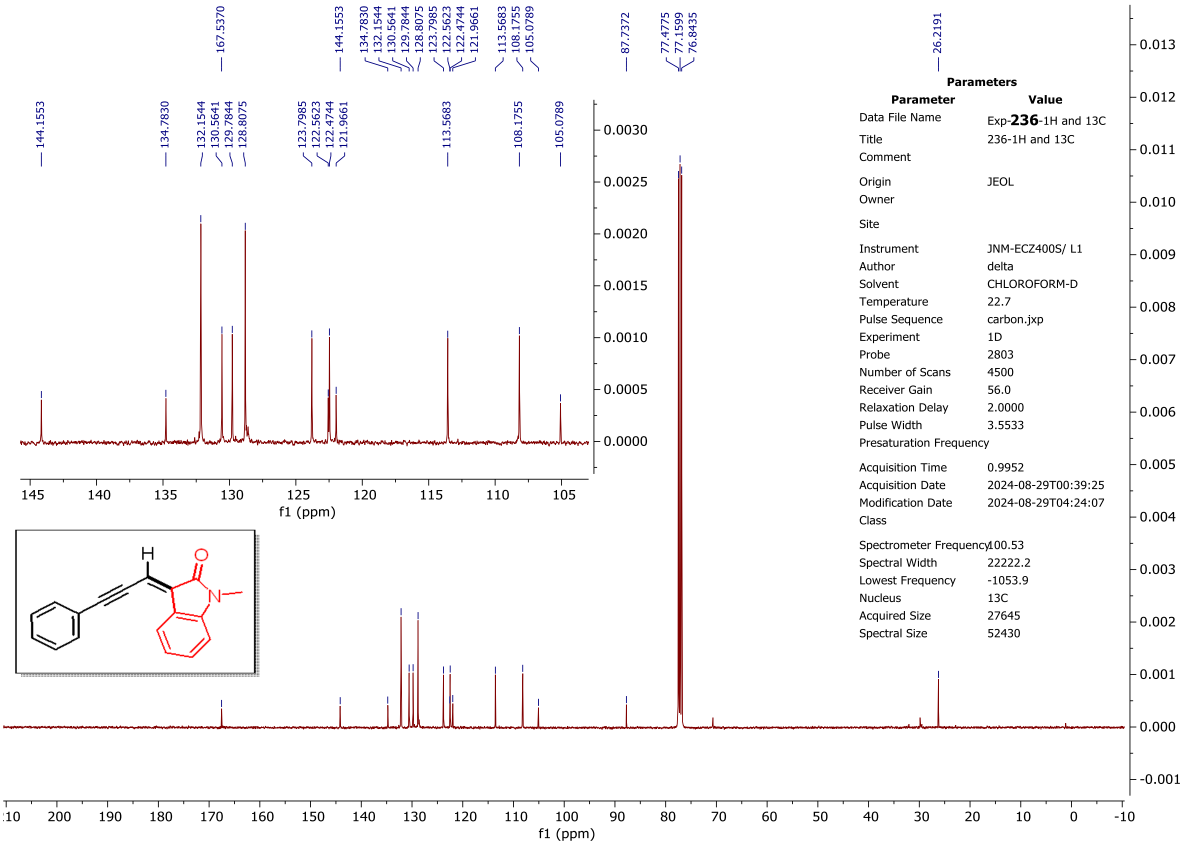

Figure S87. ^{13}C NMR (100 MHz, $CDCl_3$) spectra of compound (5c)

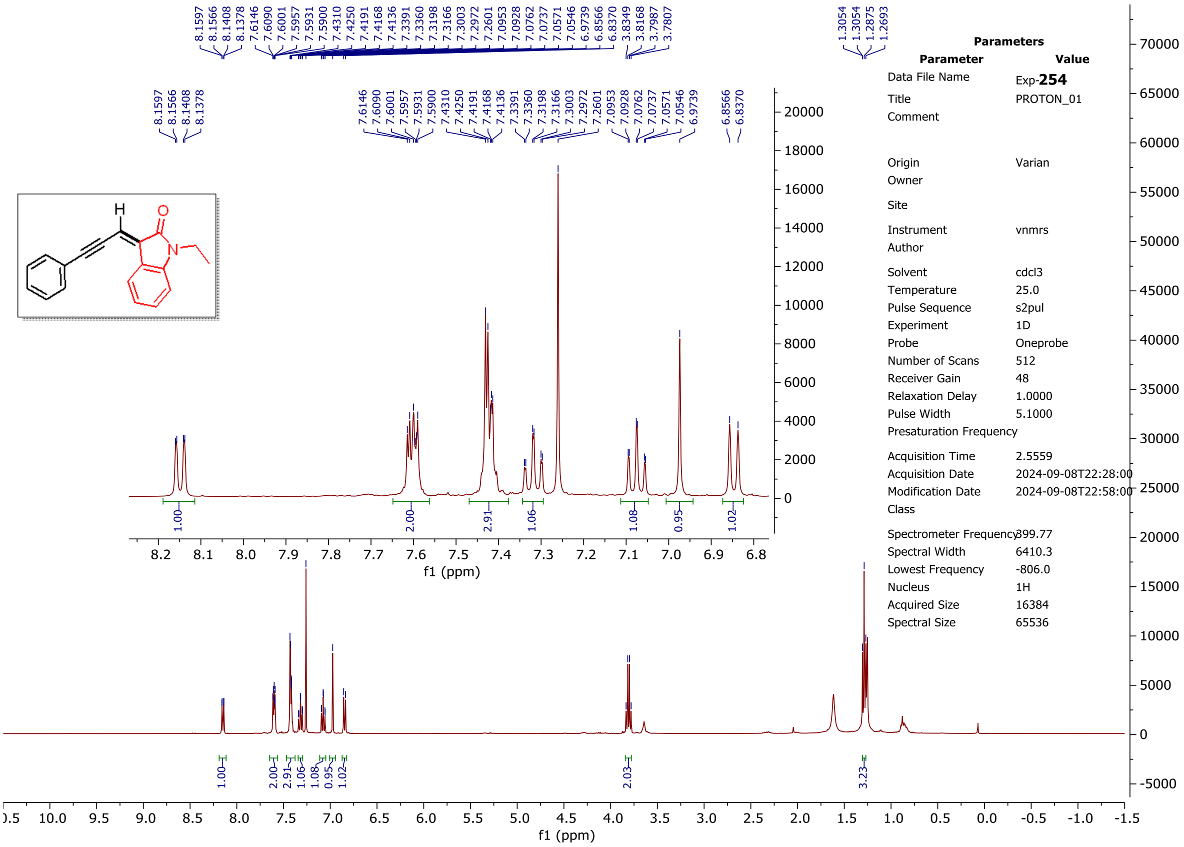

Figure S88. ^{1}H NMR (400 MHz, $CDCl_3$) spectra of compound (5d)

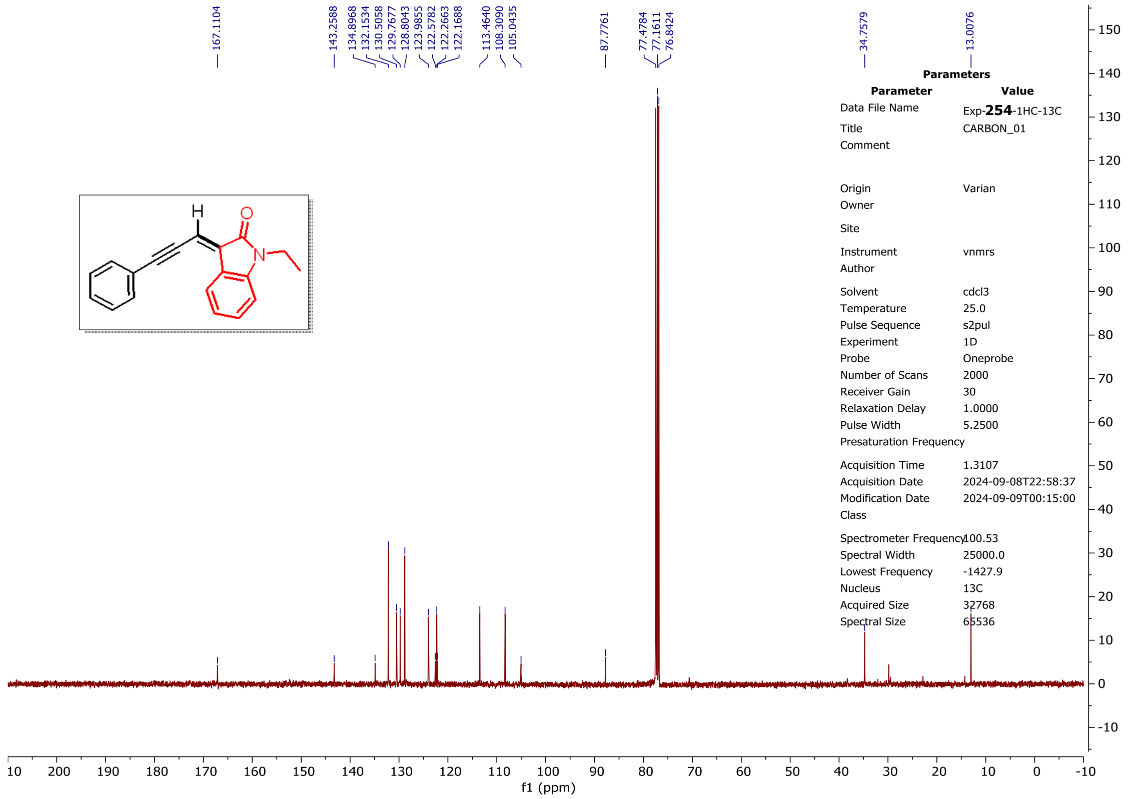

Figure S89. ^{13}C NMR (100 MHz, $CDCl_3$) spectra of compound (5d)

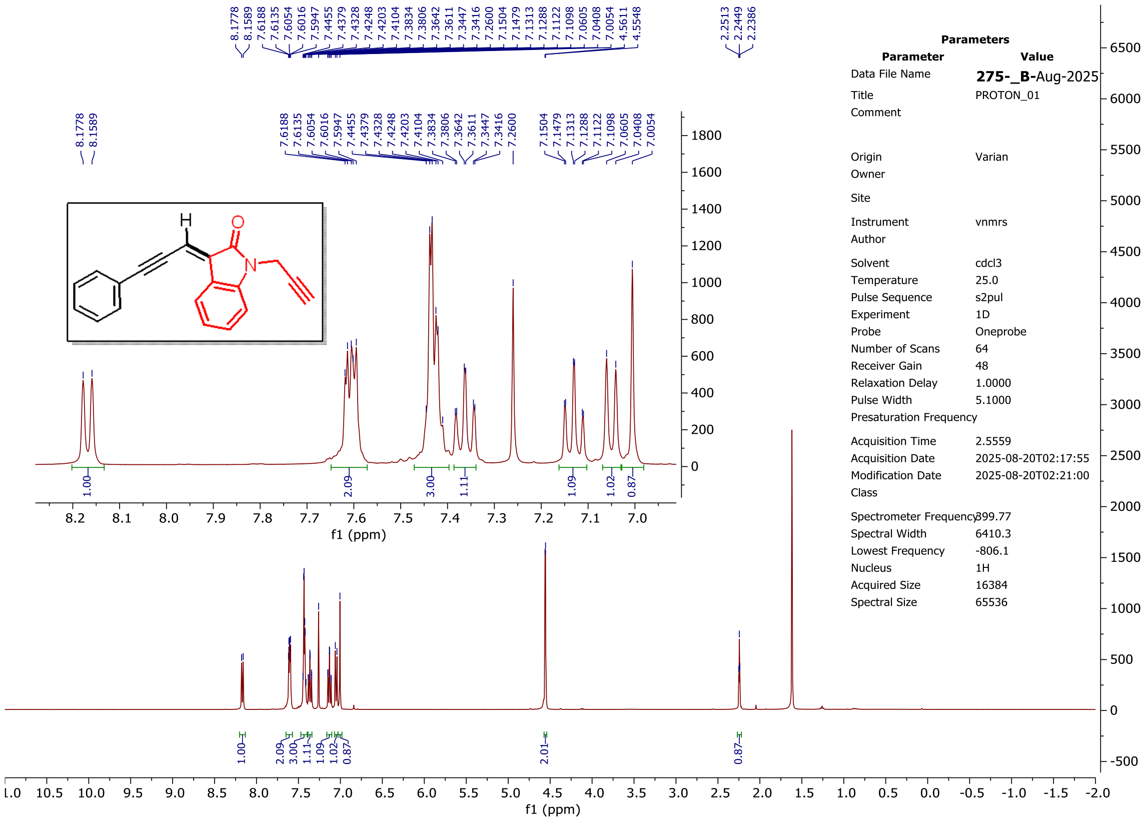

Figure S90. ^{1}H NMR (400 MHz, $CDCl_3$) spectra of compound (5e)

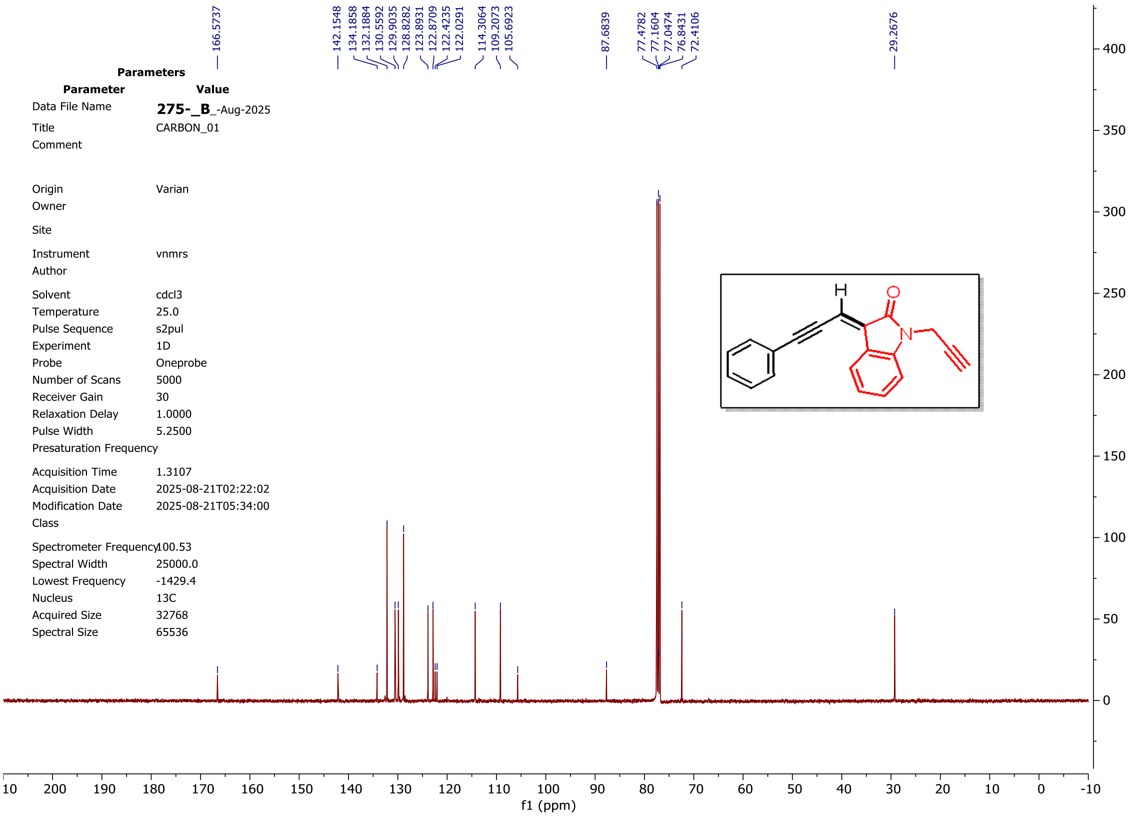

Figure S91. ^{13}**C NMR (100 MHz, CDCl**$_3$**) spectra of compound (5e)**

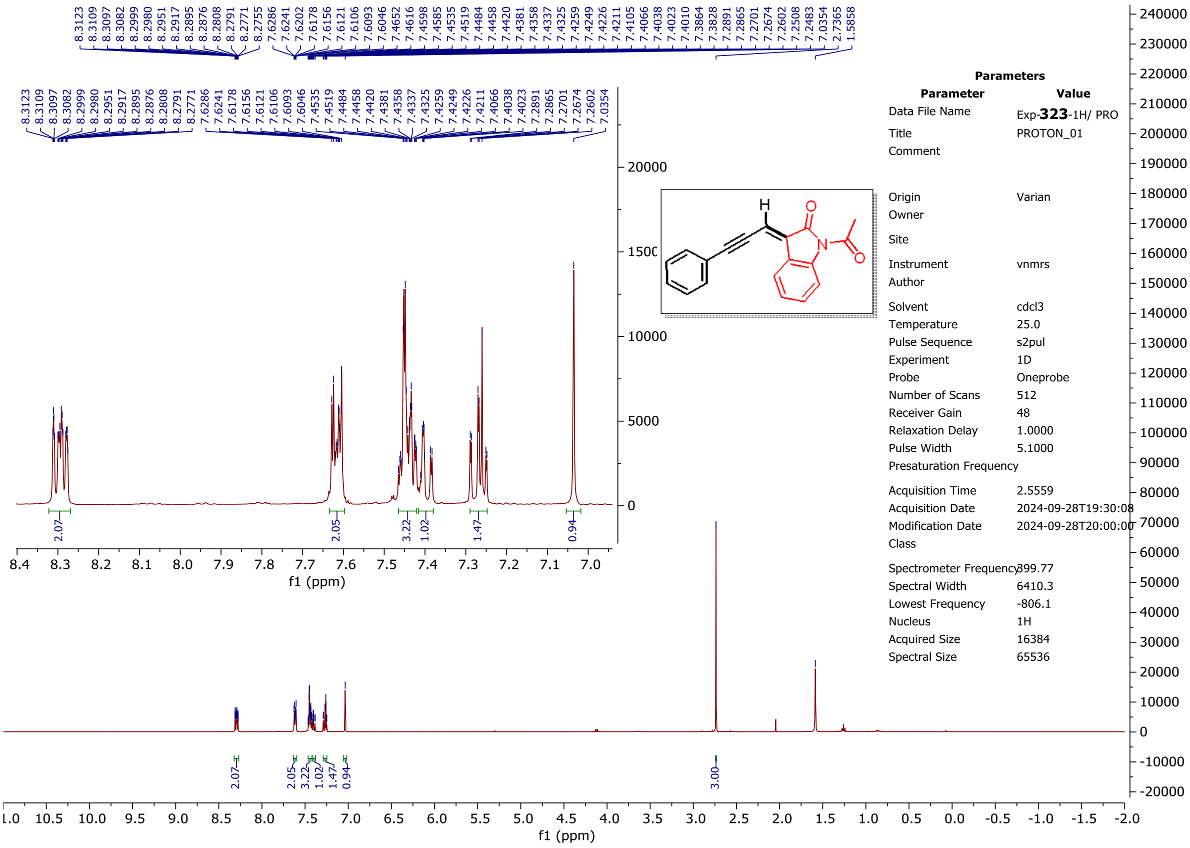

Figure S92. ^{1}**H NMR (400 MHz, CDCl**$_3$**) spectra of compound (5f)**

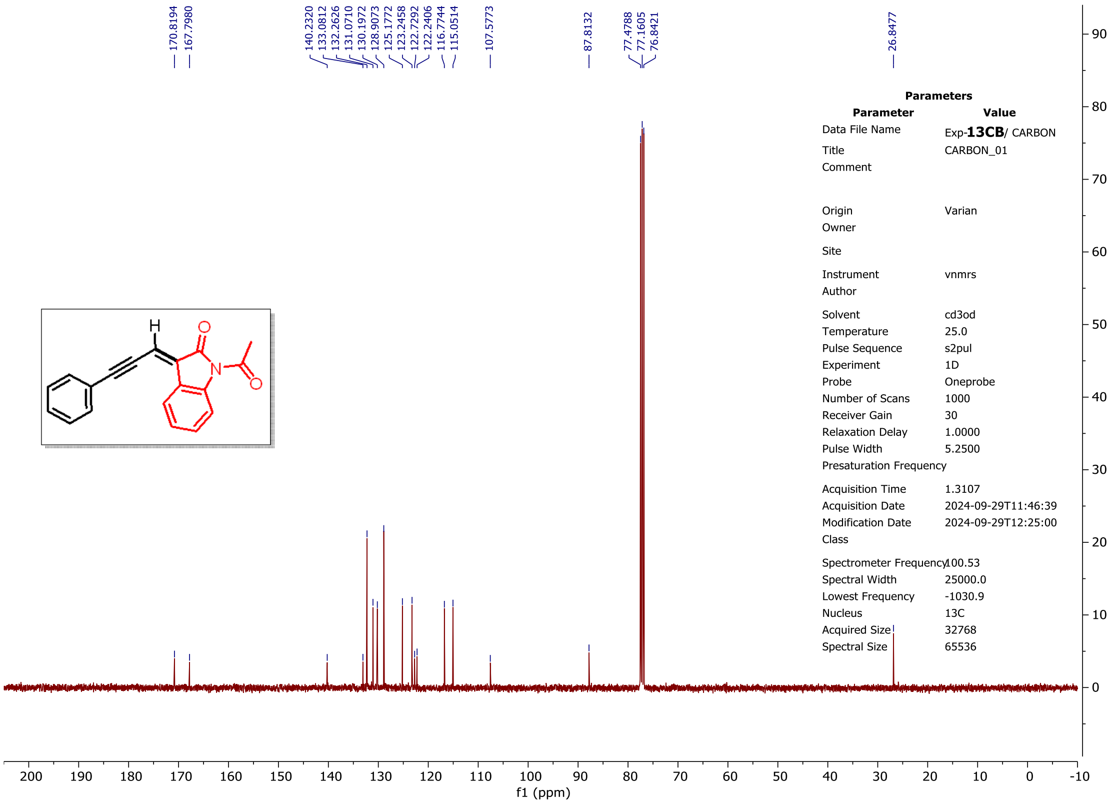

Figure S93. ^{13}C NMR (100 MHz, $CDCl_3$) spectra of compound (5f)

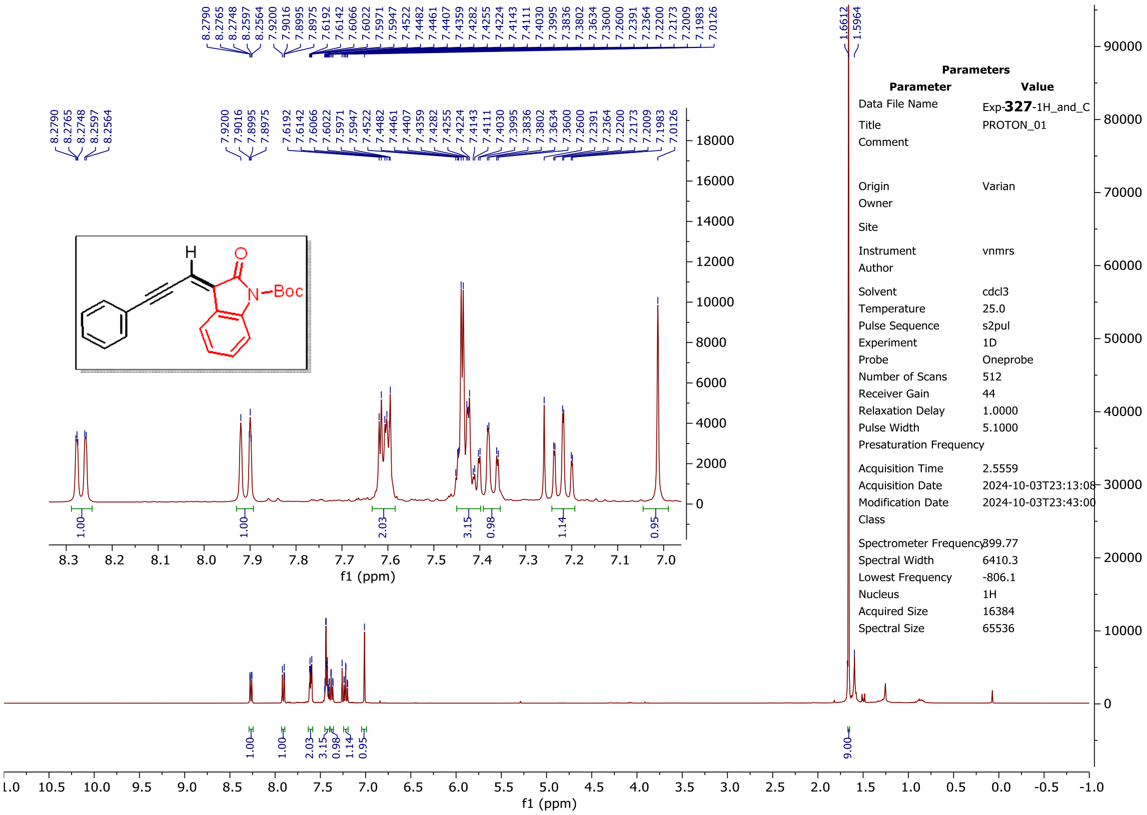

Figure S94. ^{1}H NMR (400 MHz, $CDCl_3$) spectra of compound (5g)

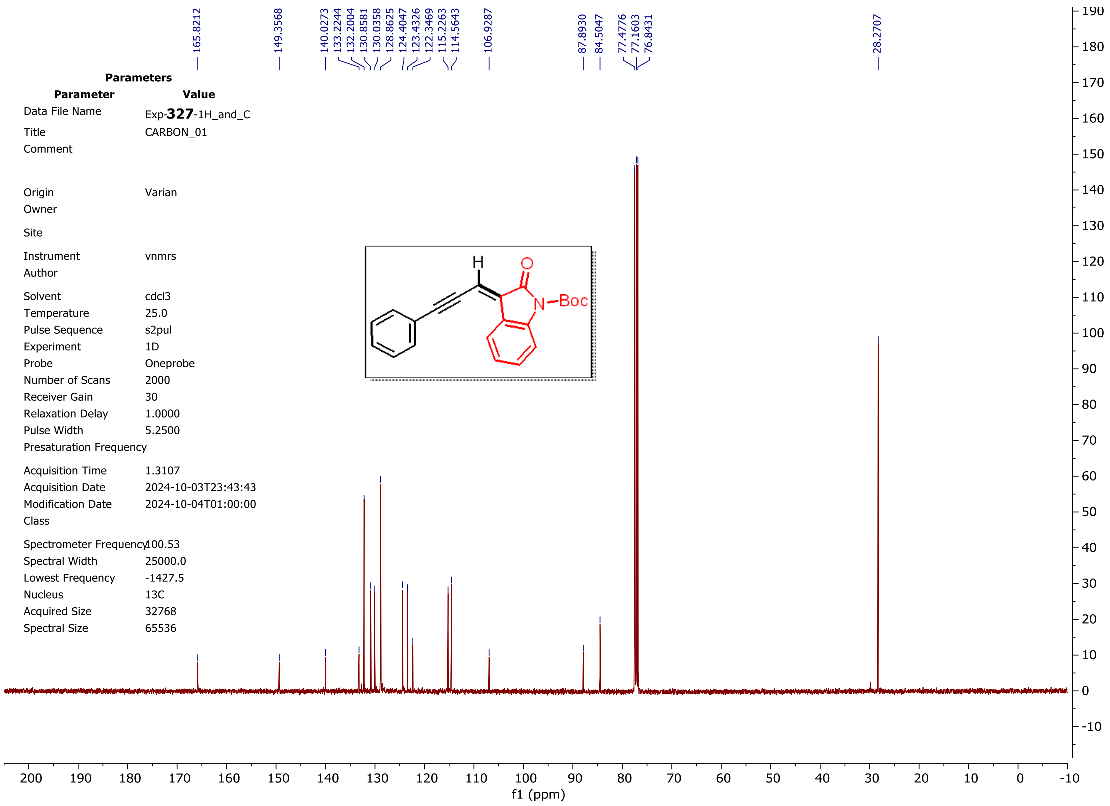

Figure S95. ^{13}C NMR (100 MHz, $CDCl_3$) spectra of compound (5g)

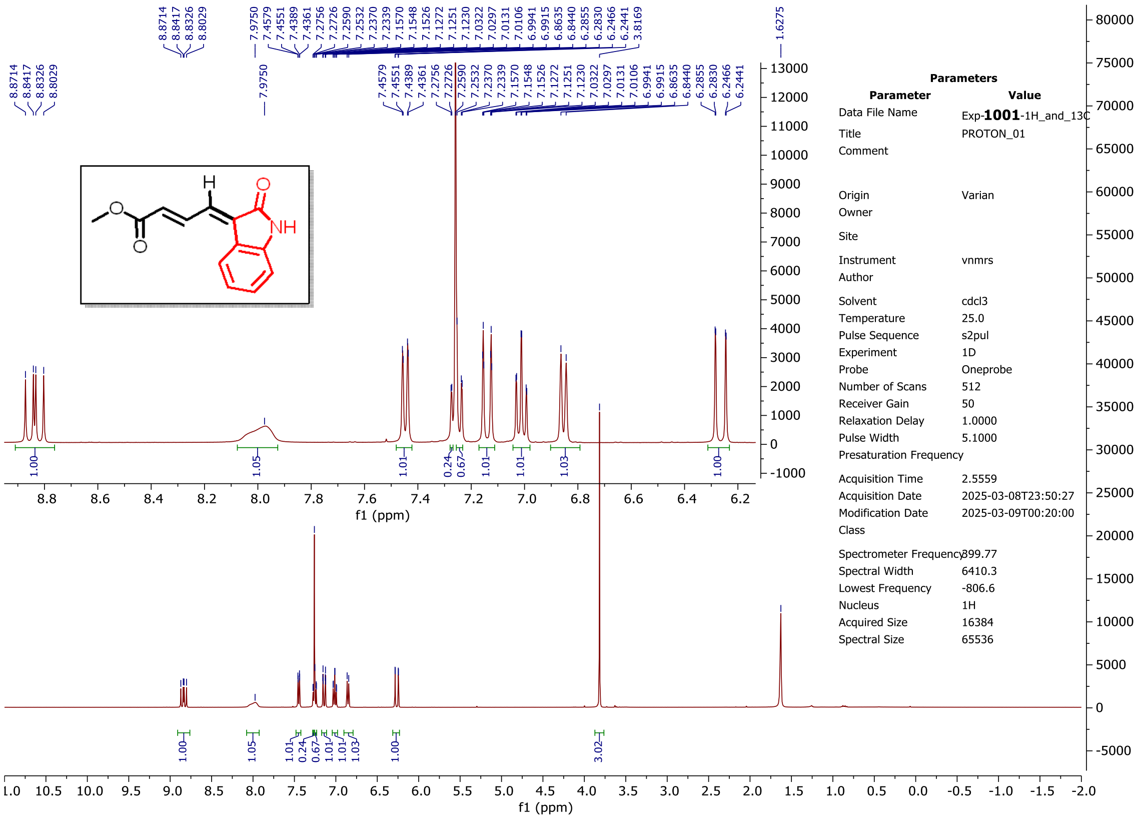

Figure S96. ^{1}H NMR (400 MHz, $CDCl_3$) spectra of compound (6a)

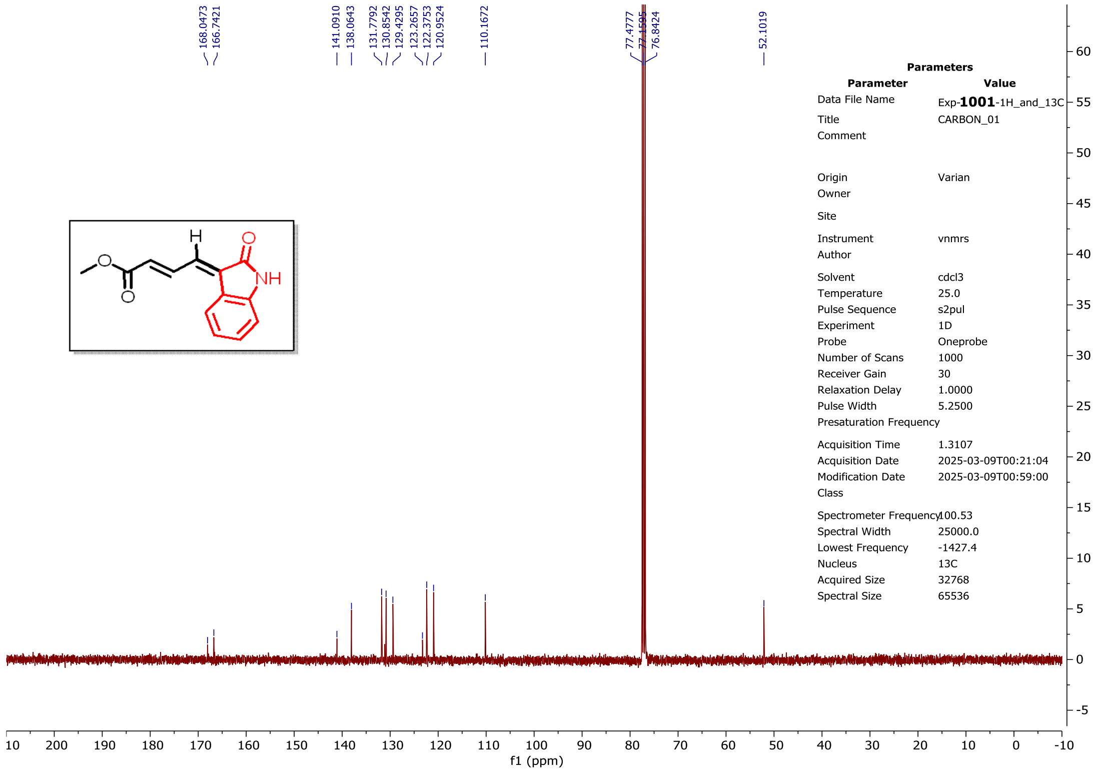

Figure S97. ^{13}C NMR (100 MHz, $CDCl_3$) spectra of compound (6a)

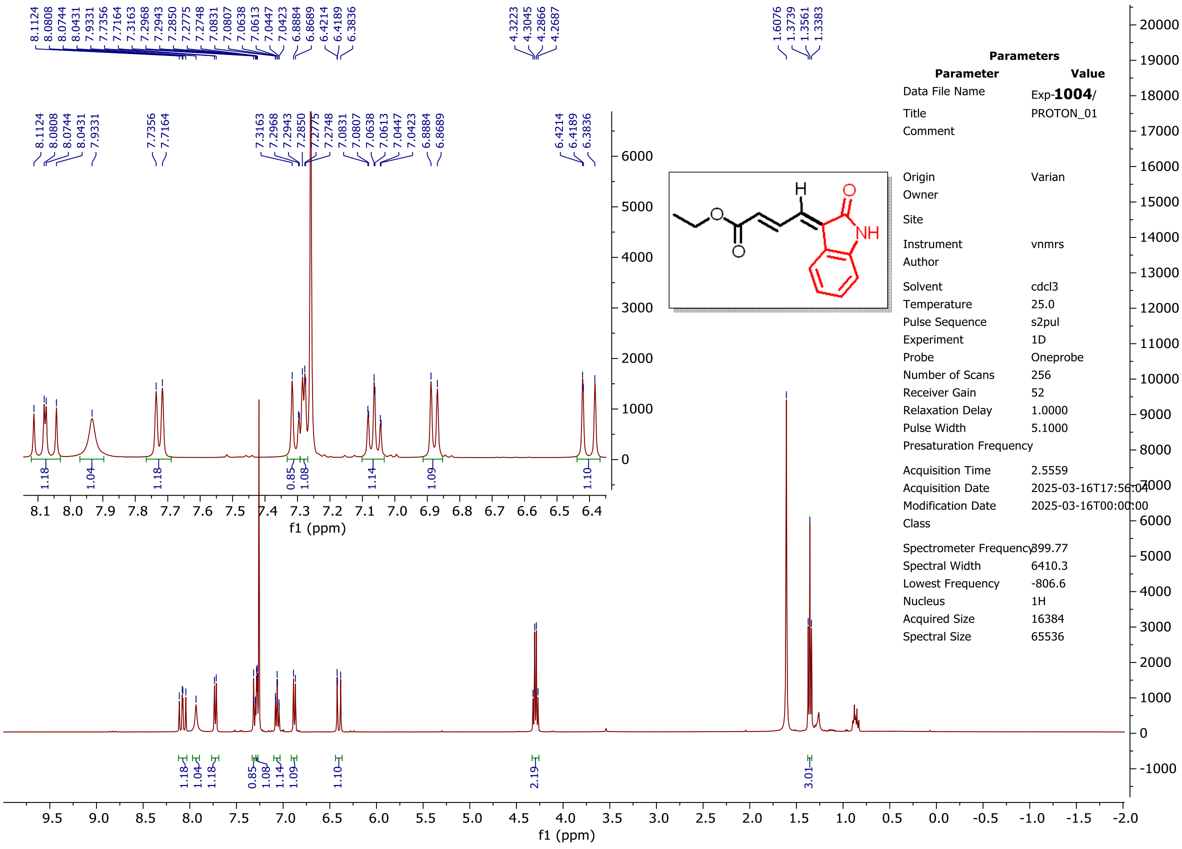

Figure S98. ^{1}H NMR (400 MHz, $CDCl_3$) spectra of compound (6b)

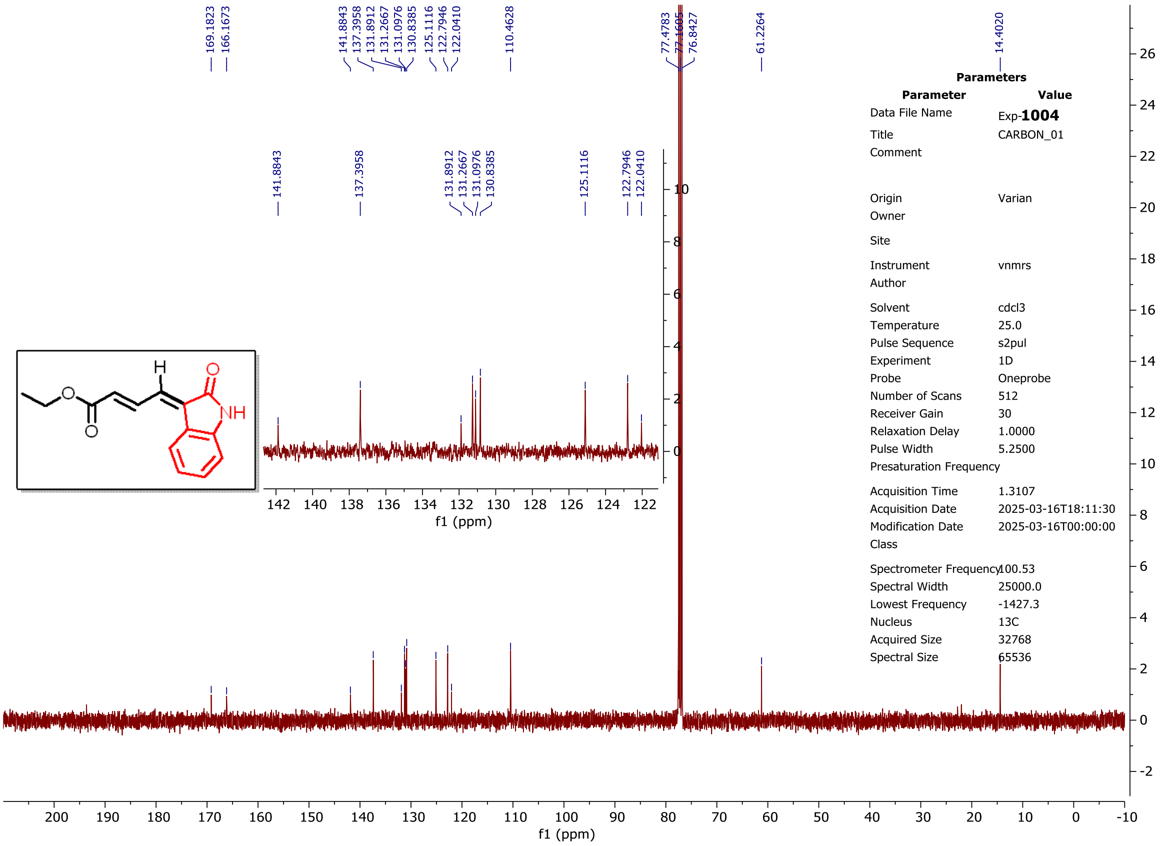

Figure S99. ^{13}C NMR (100 MHz, $CDCl_3$) spectra of compound (6b)

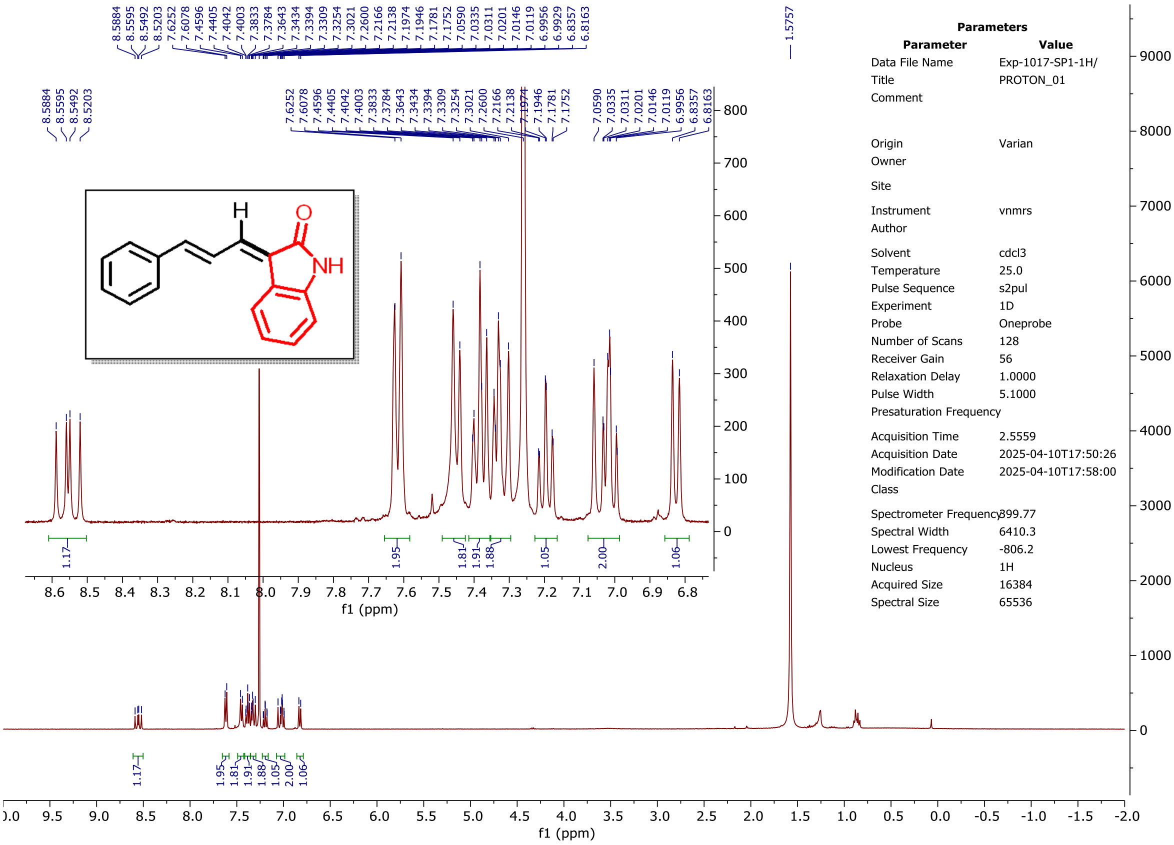

Figure S100. ^{1}H NMR (400 MHz, $CDCl_3$) spectra of compound (6c)

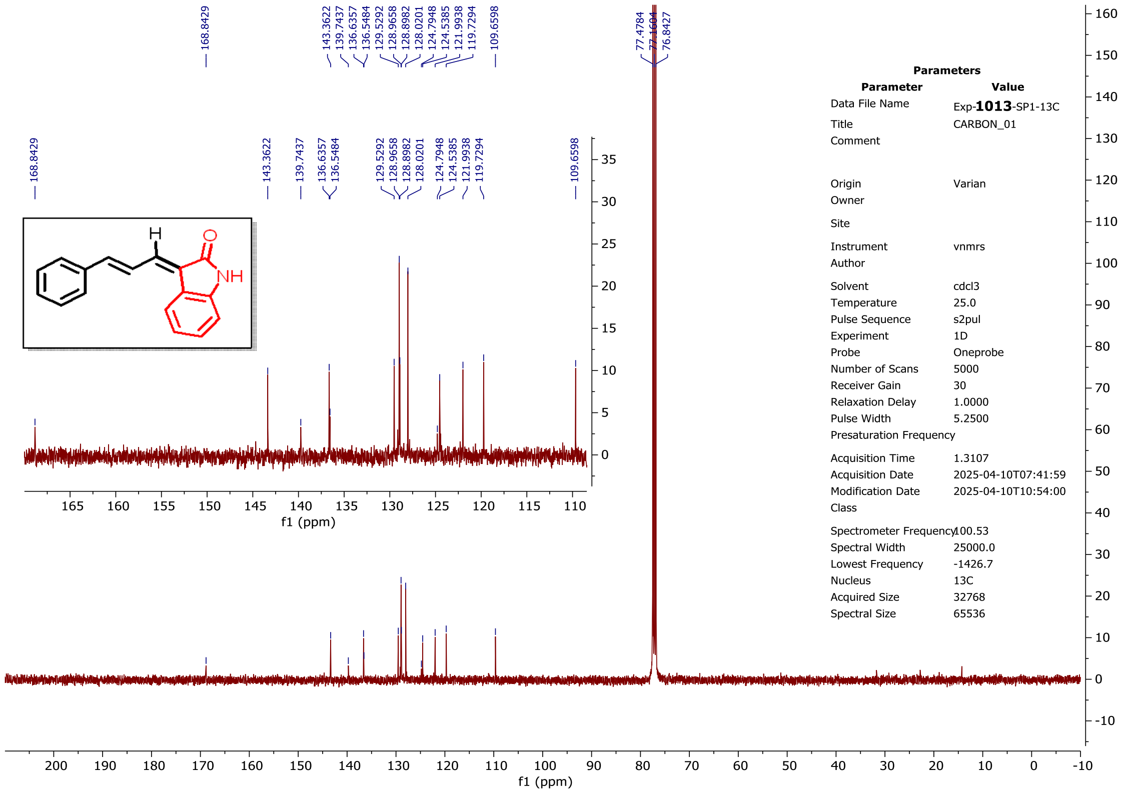

Figure S101. ^{13}C **NMR (100 MHz,** $CDCl_3$**) spectra of compound (6c)**

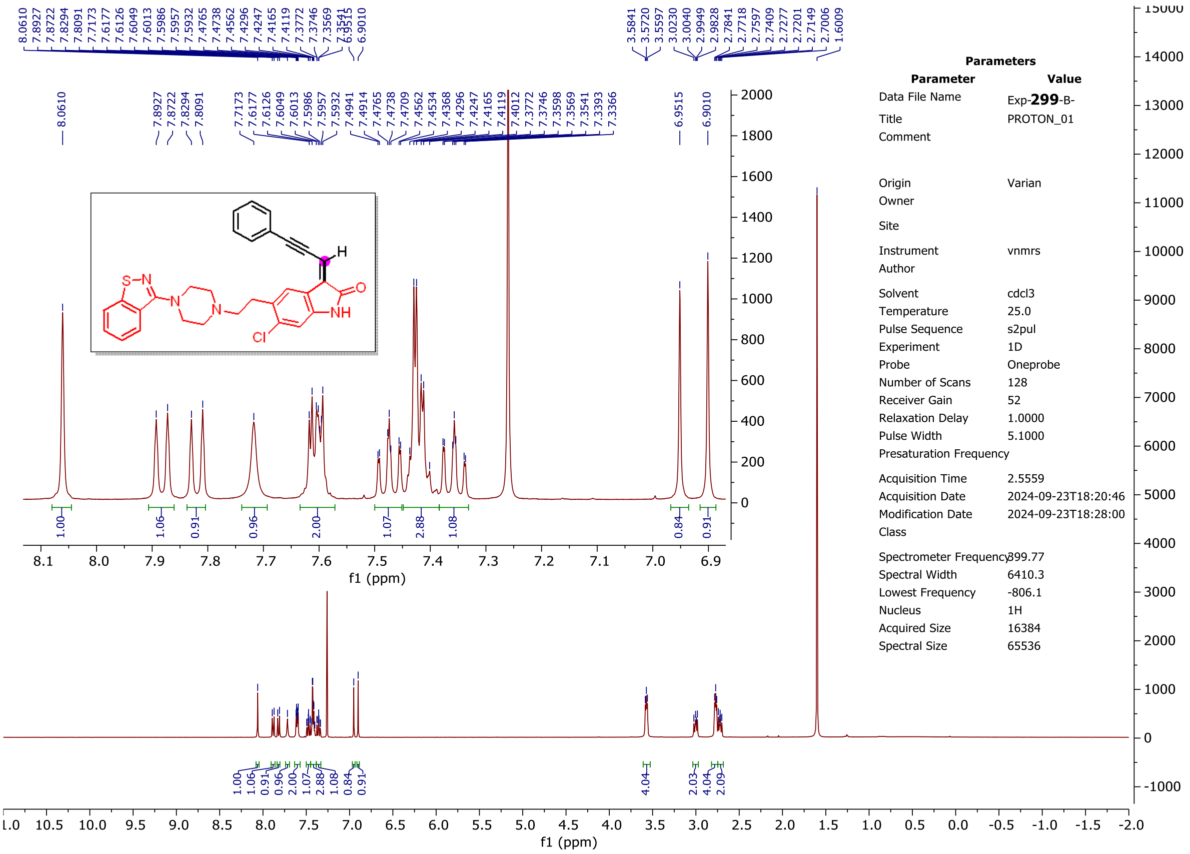

Figure S102. ^{1}H **NMR (400 MHz,** $CDCl_3$**) spectra of compound (7a)**

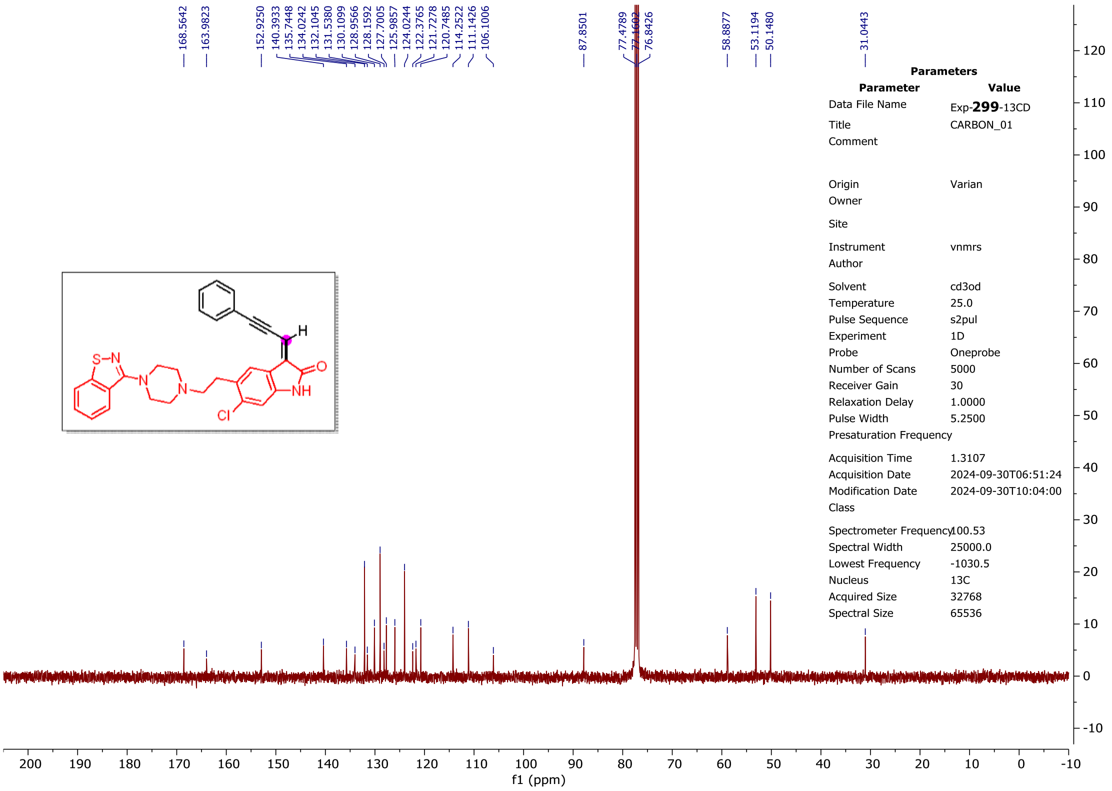

Figure S103. ^{13}C **NMR (100 MHz,** $CDCl_3$**) spectra of compound (7a)**

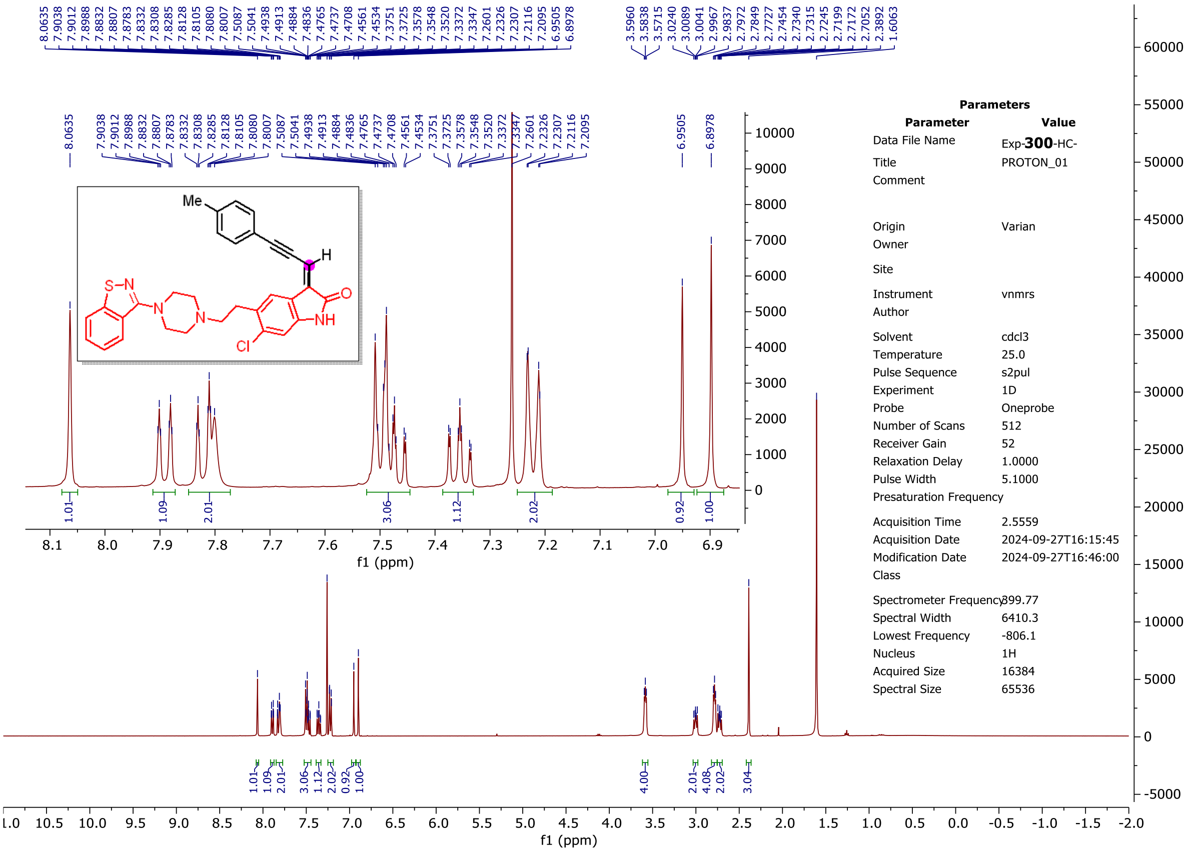

Figure S104. ^{1}H **NMR (400 MHz,** $CDCl_3$**) spectra of compound (7b)**

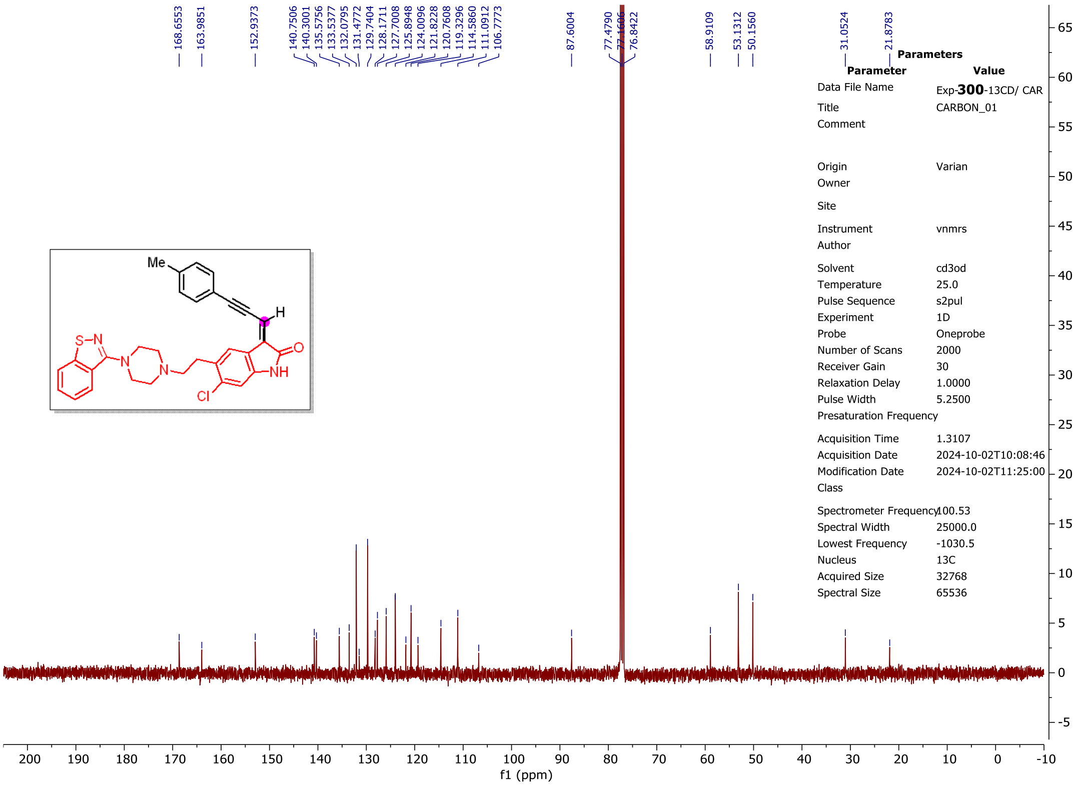

Figure S105. ^{13}C **NMR (100 MHz,** $CDCl_3$**) spectra of compound (7b)**

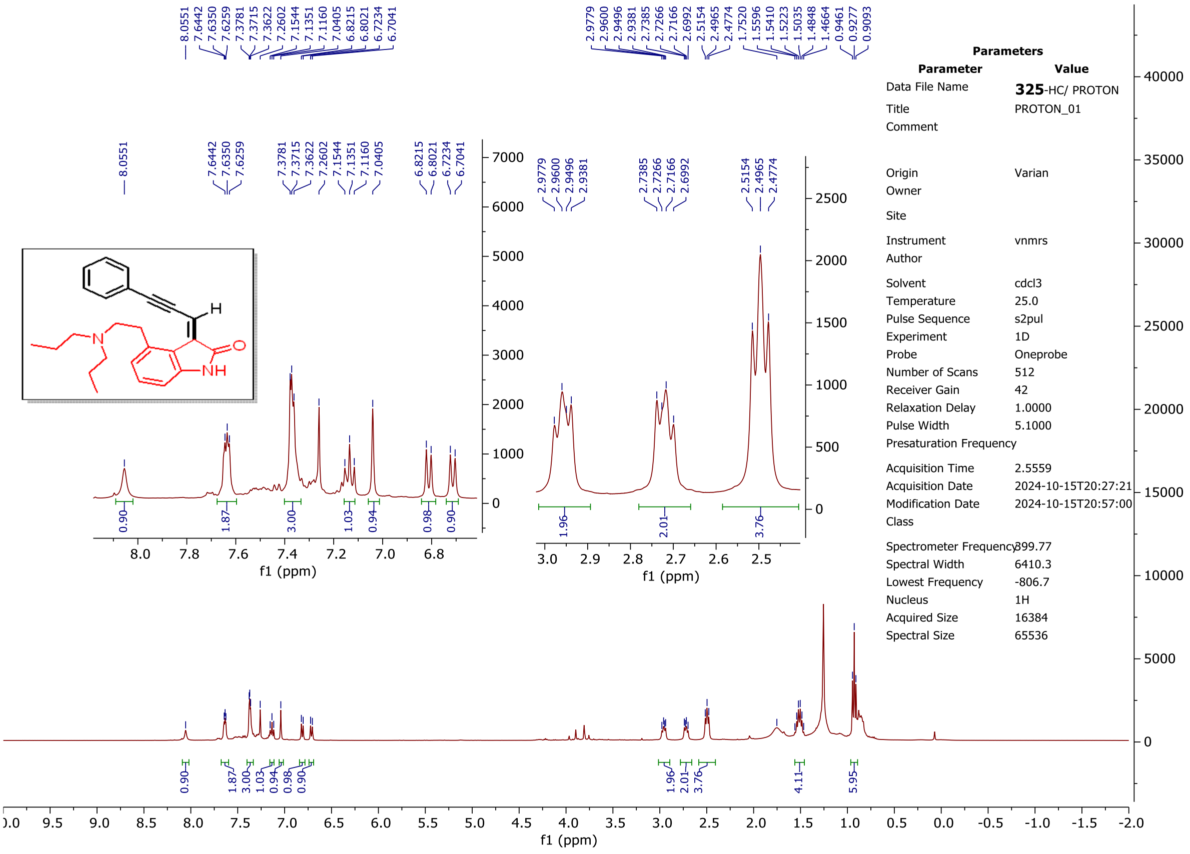

Figure S106. ^{1}H **NMR (400 MHz,** $CDCl_3$**) spectra of compound (7c)**

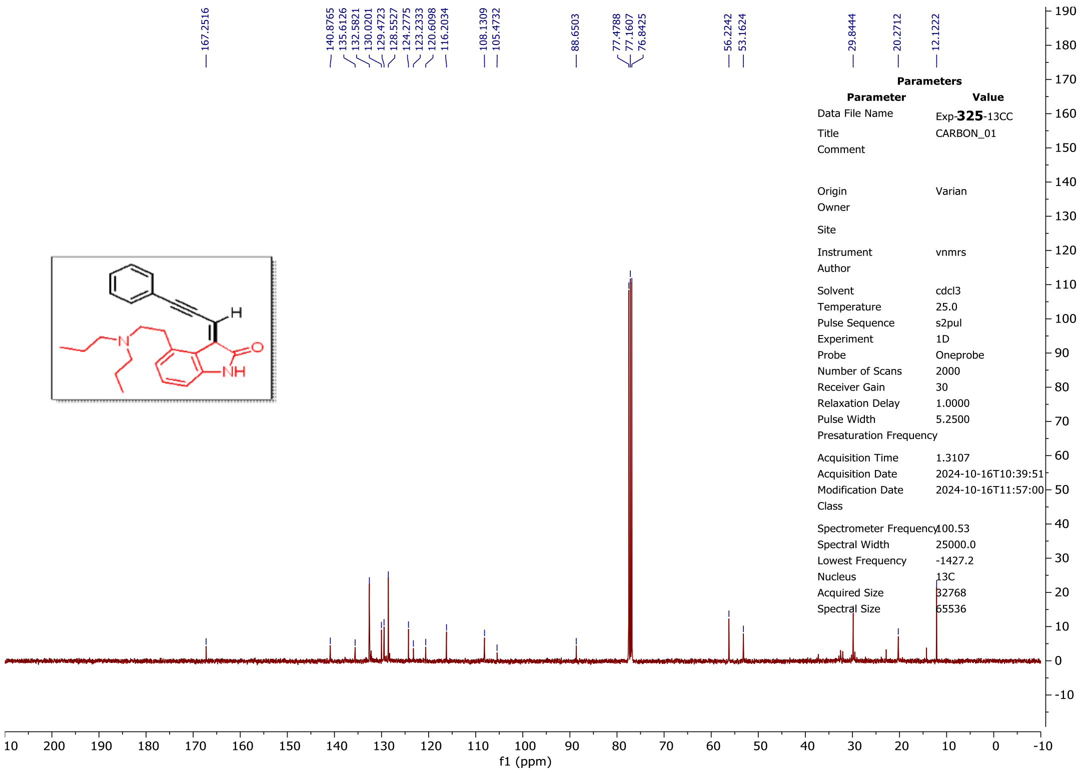

Figure S107. ^{13}C NMR (100 MHz, $CDCl_3$) spectra of compound (7c)

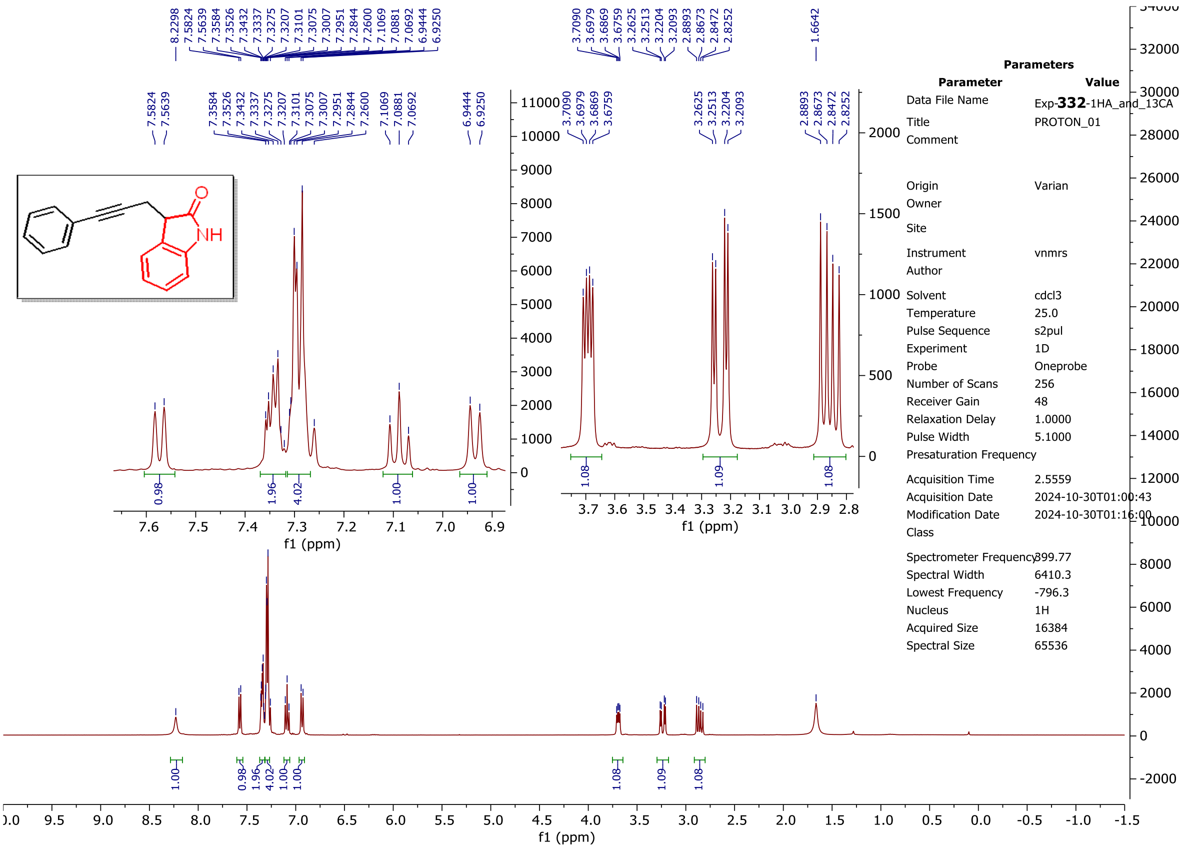

Figure S108. ^{1}H NMR (400 MHz, $CDCl_3$) spectra of compound (8)

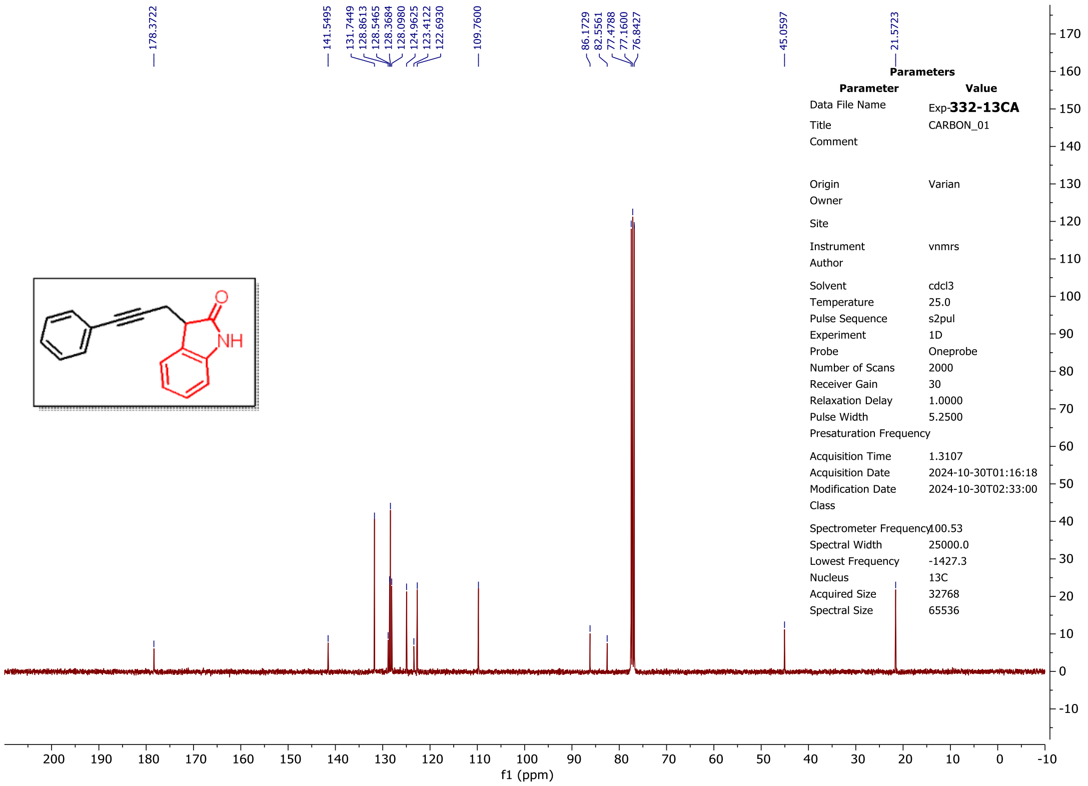

Figure S109. ^{13}C NMR (100 MHz, $CDCl_3$) spectra of compound (8)

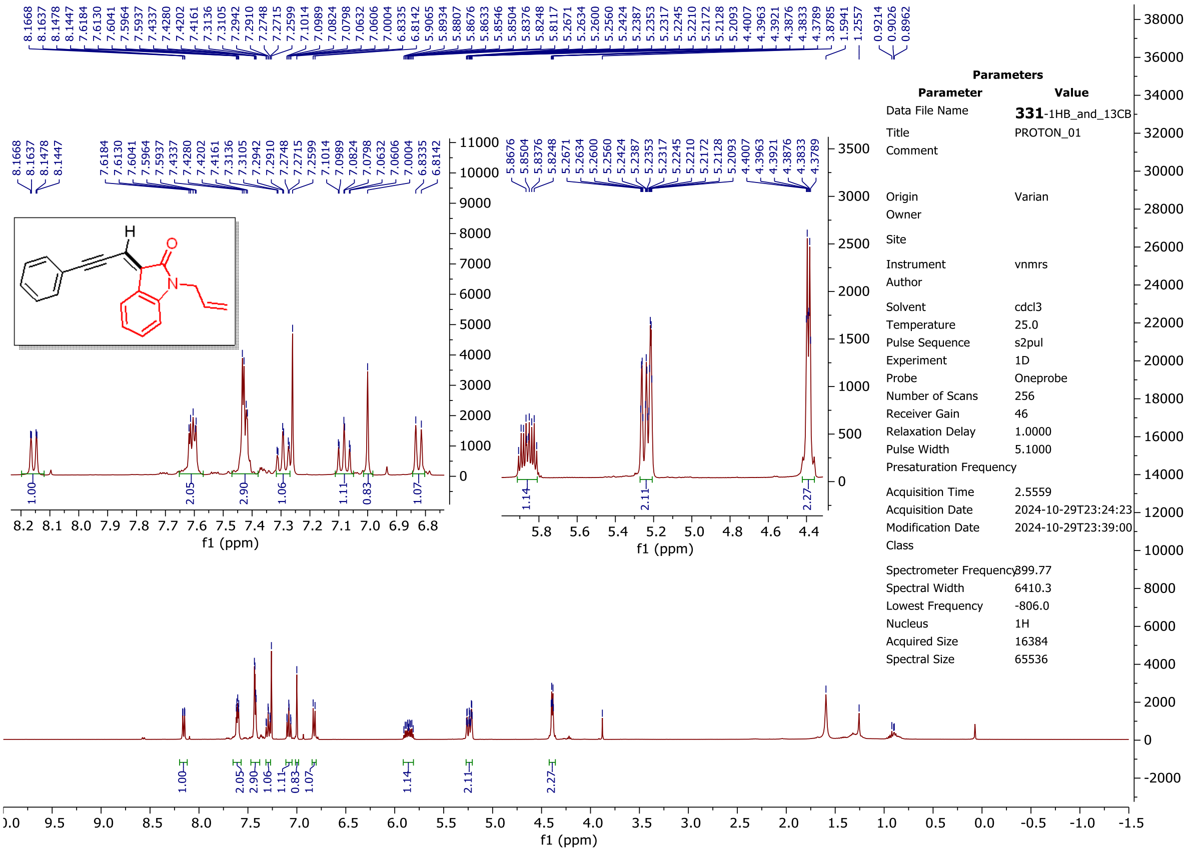

Figure S110. ^{1}H NMR (400 MHz, $CDCl_3$) spectra of compound (9)

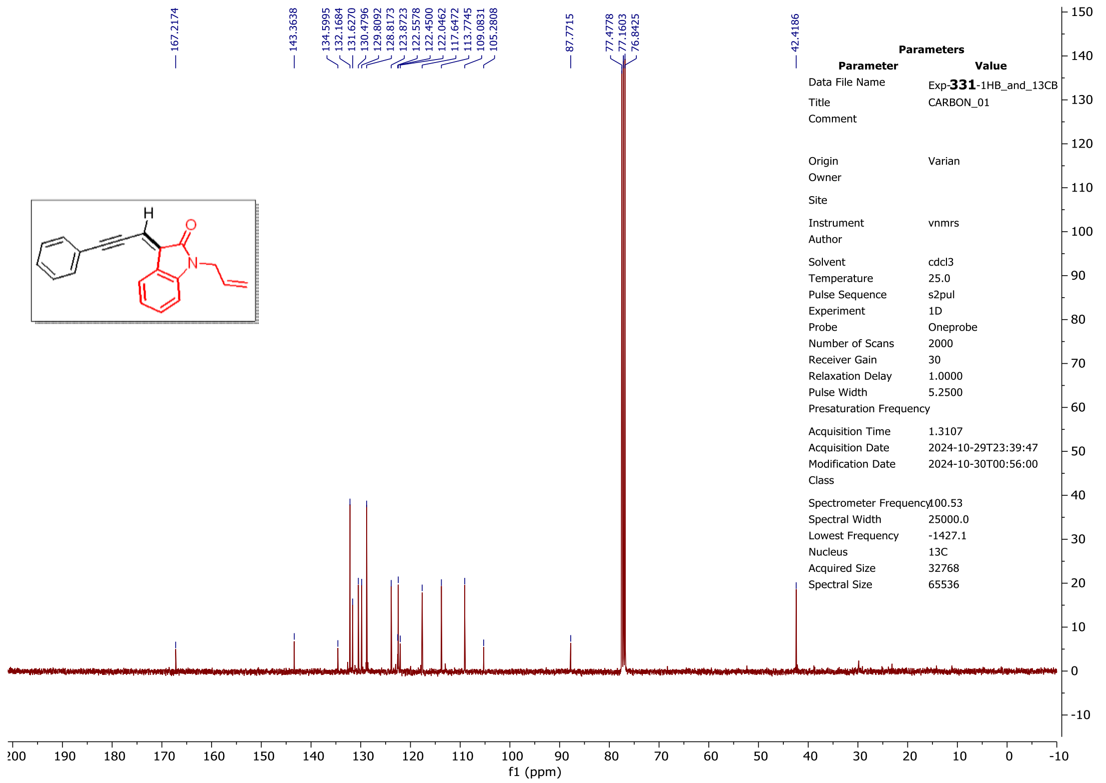

Figure S111. ^{13}C NMR (100 MHz, $CDCl_3$) spectra of compound (9)

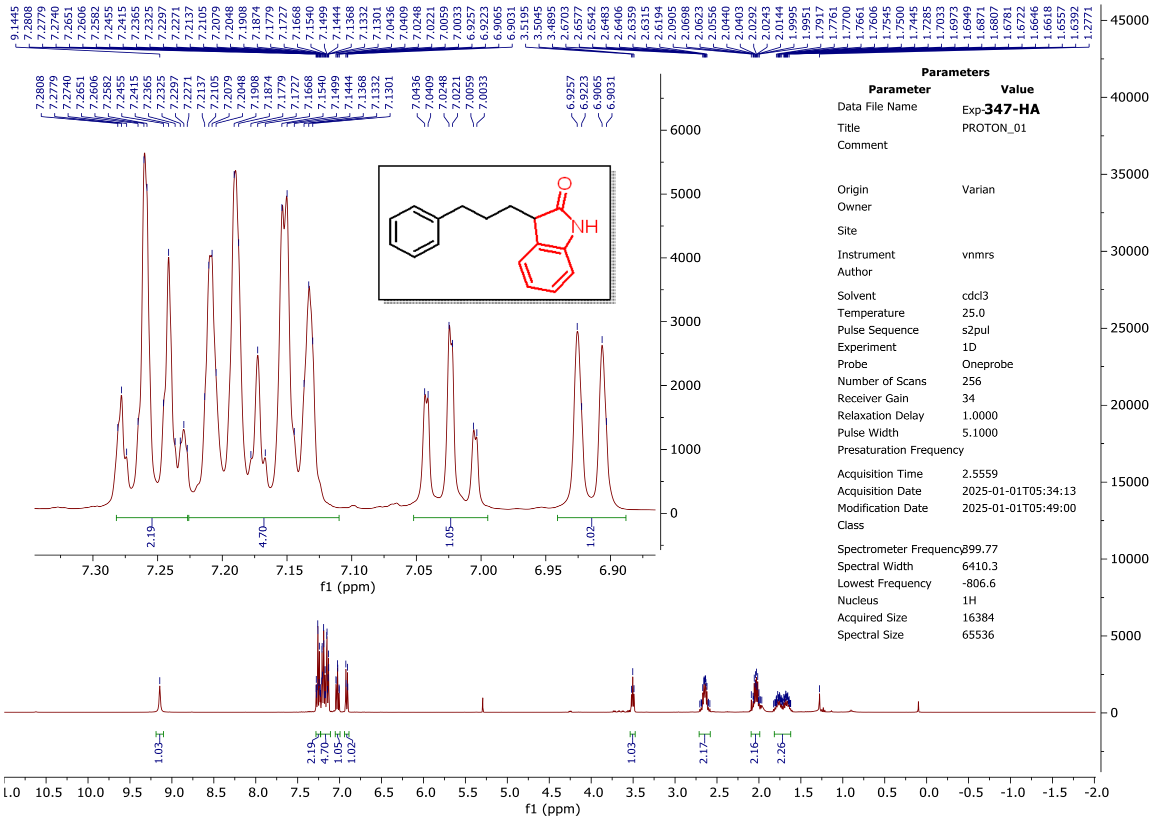

Figure S112. ^{1}H NMR (400 MHz, $CDCl_3$) spectra of compound (10)

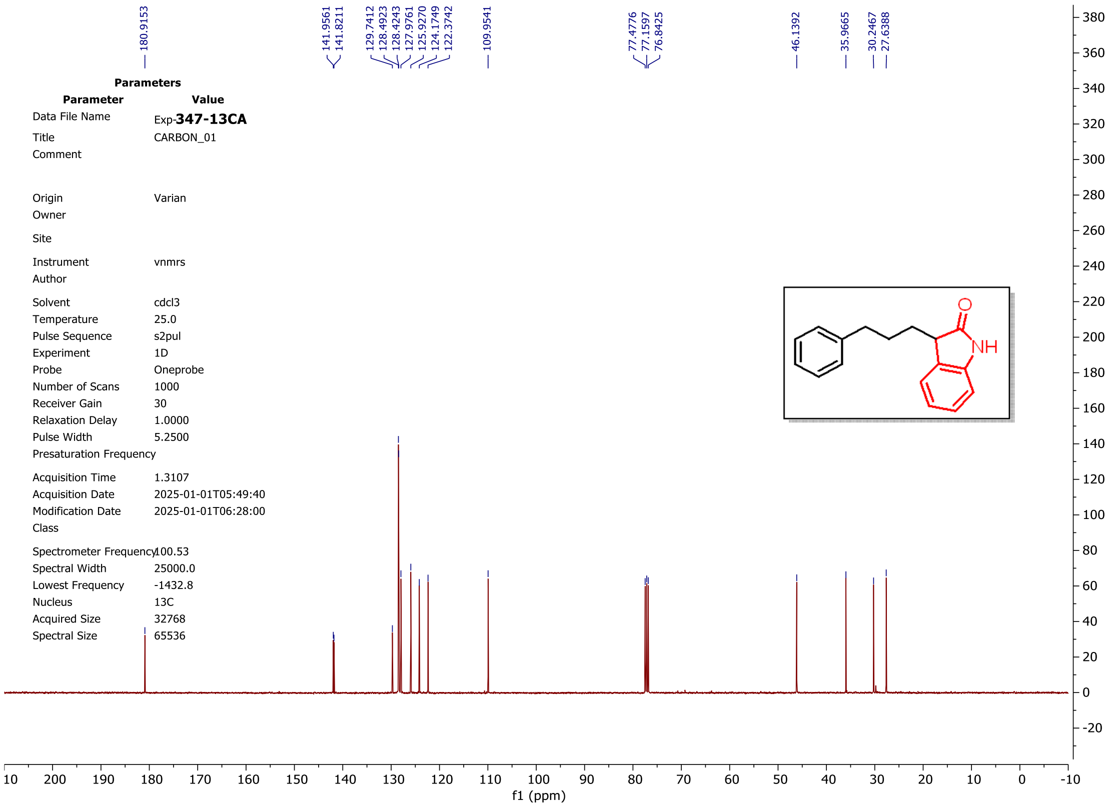

Figure S113. ^{13}C NMR (100 MHz, $CDCl_3$) spectra of compound (10)

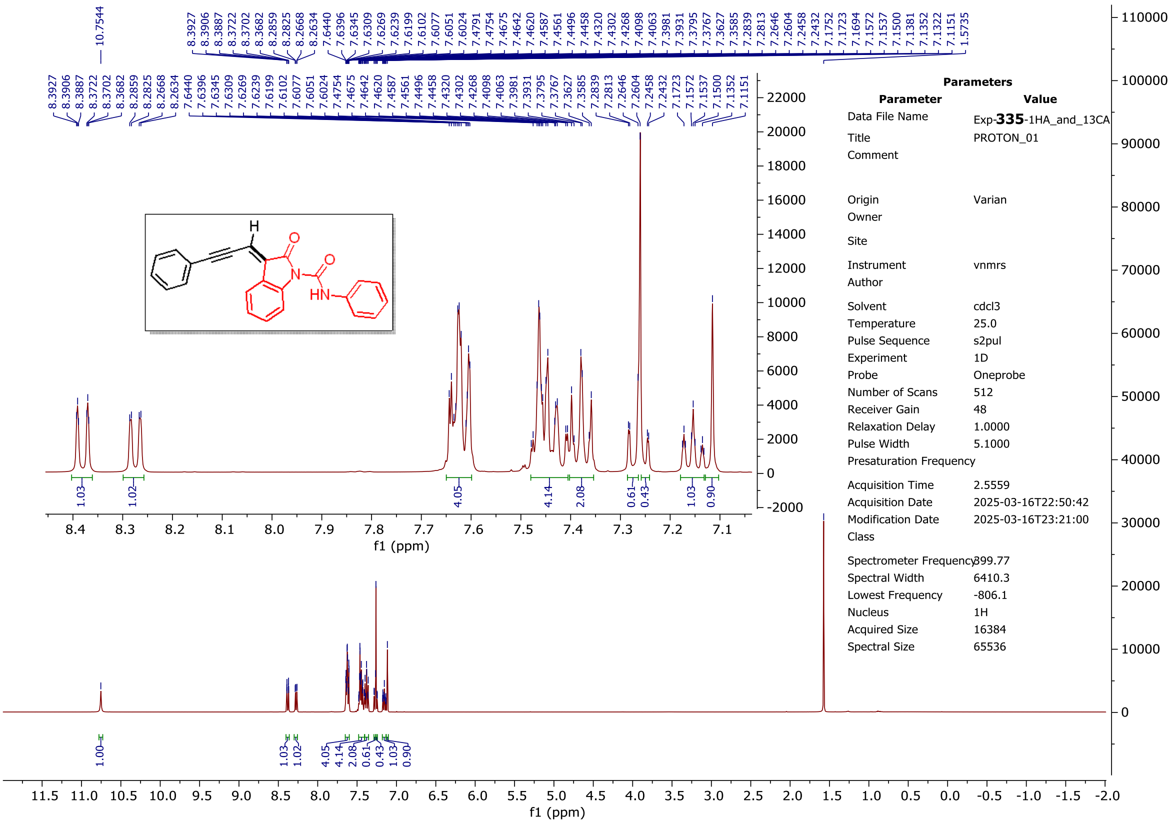

Figure S114. ^{1}H NMR (400 MHz, $CDCl_3$) spectra of compound (11a)

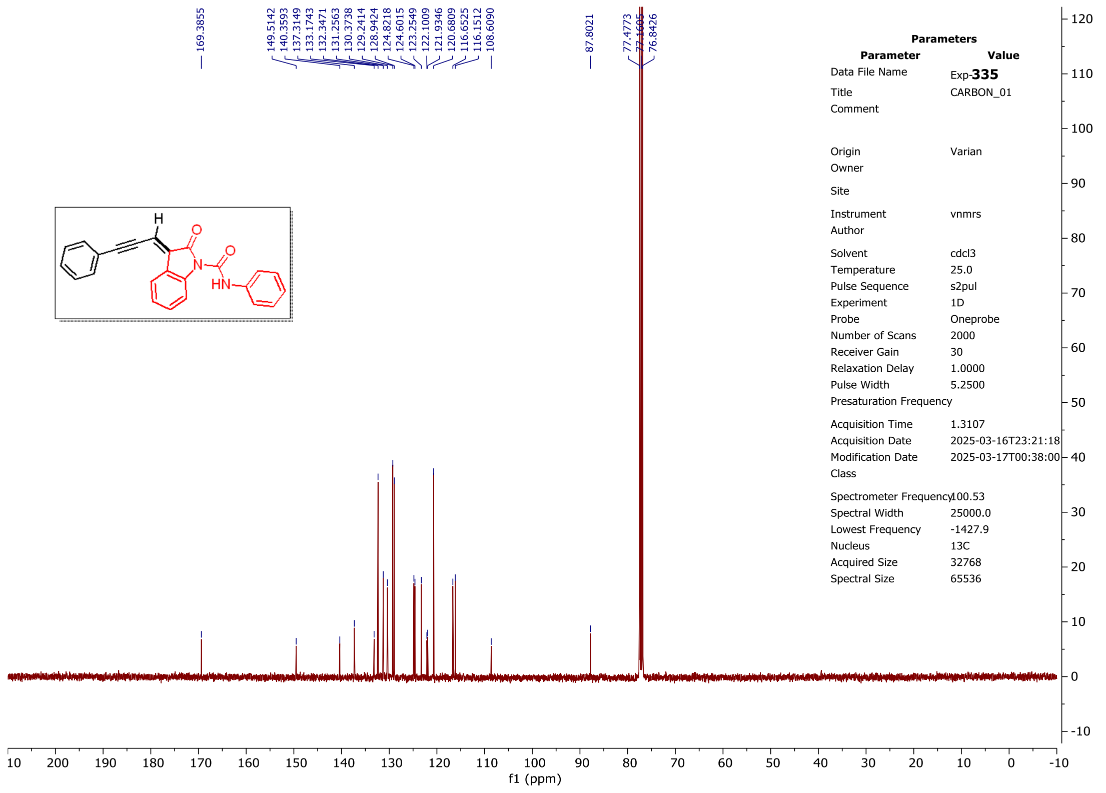

Figure S115. ^{13}C **NMR (100 MHz,** $CDCl_3$**) spectra of compound (11a)**

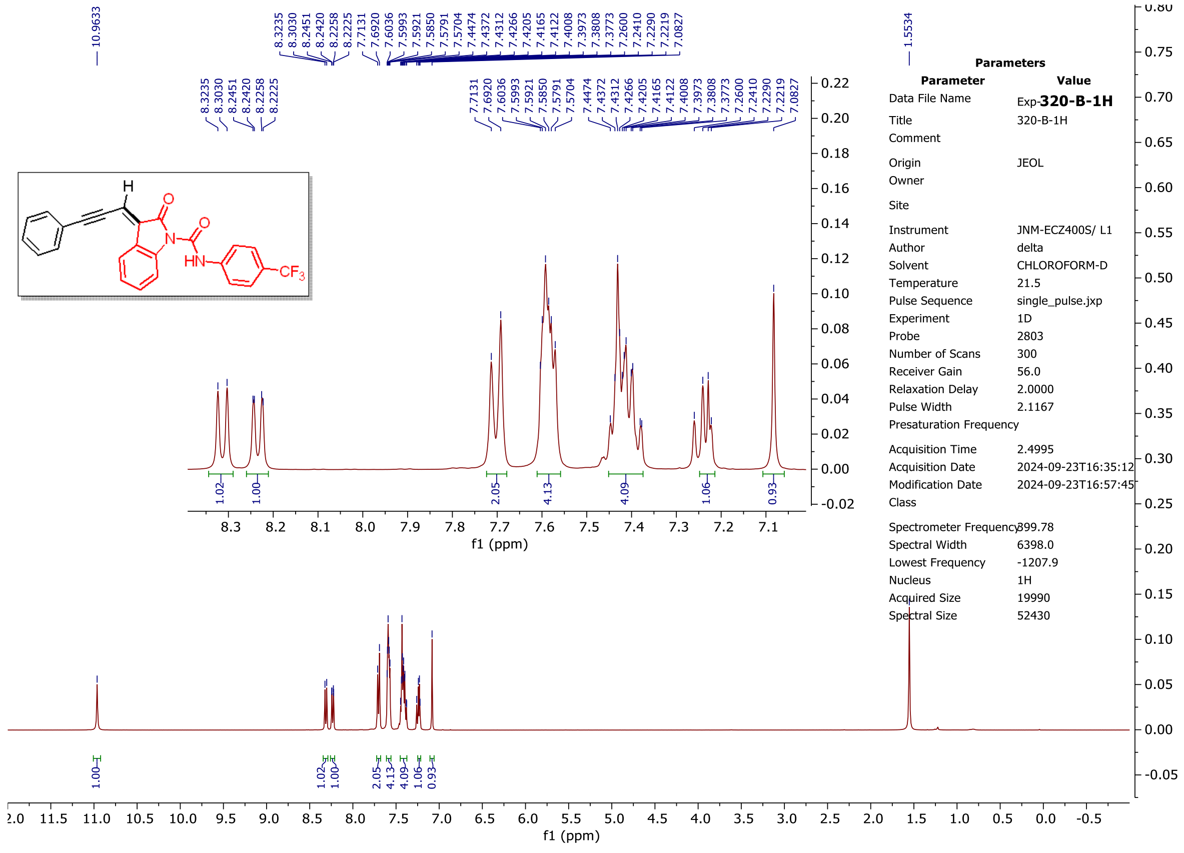

Figure S116. ^{1}H **NMR (400 MHz,** $CDCl_3$**) spectra of compound (11b)**

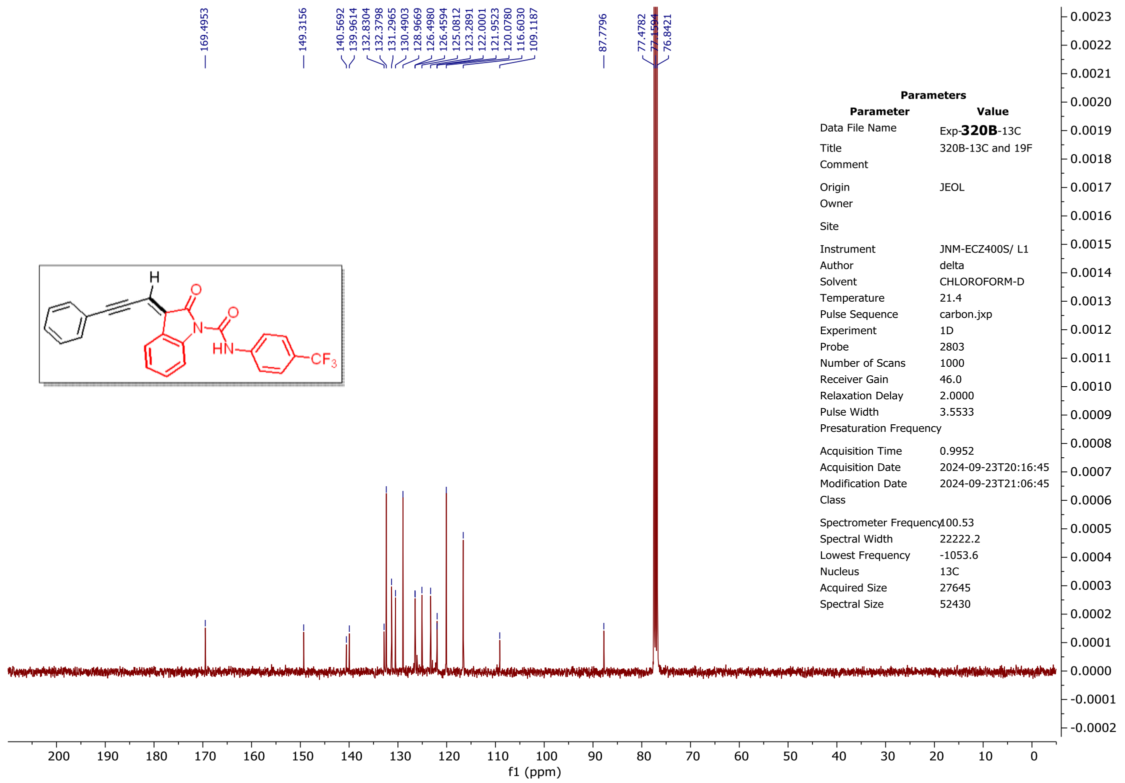

Figure S117. ^{13}C **NMR (100 MHz, $CDCl_3$) spectra of compound (11b)**

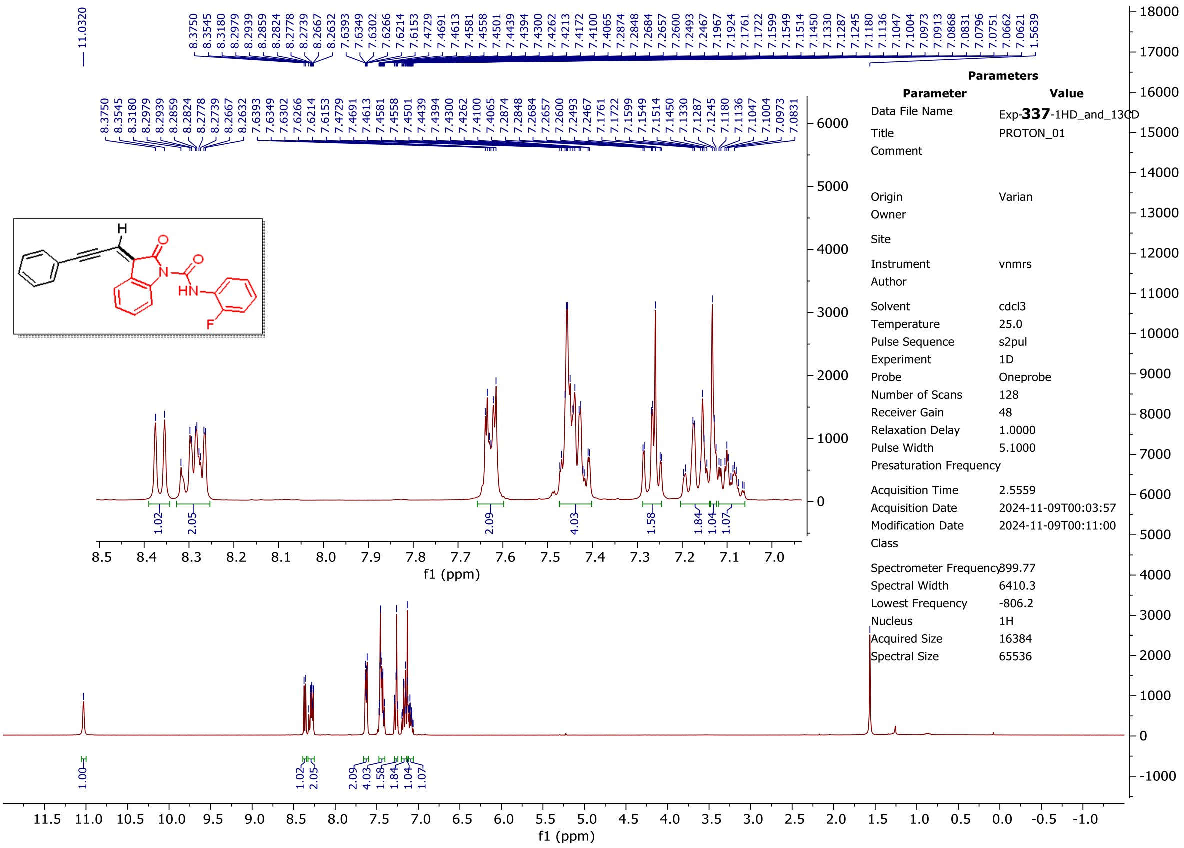

Figure S118. ^{1}H **NMR (400 MHz, $CDCl_3$) spectra of compound (11c)**

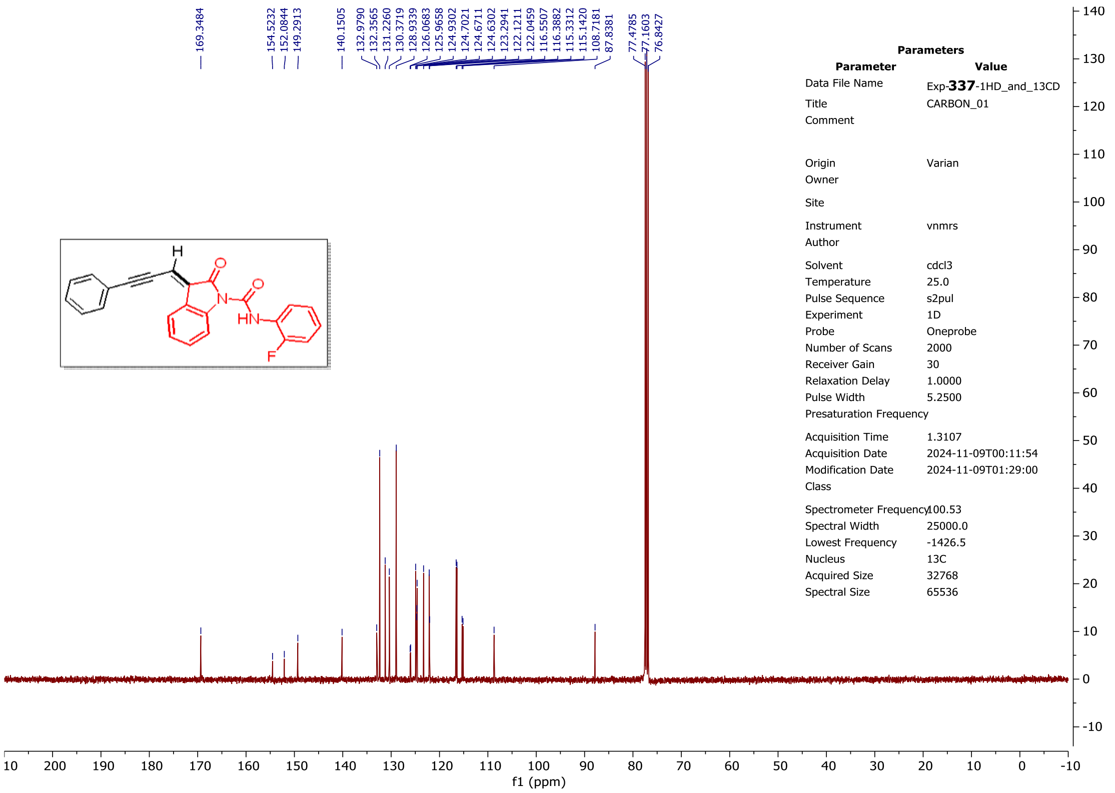

Figure S119. ^{13}C **NMR (100 MHz,** $CDCl_3$**) spectra of compound (11c)**

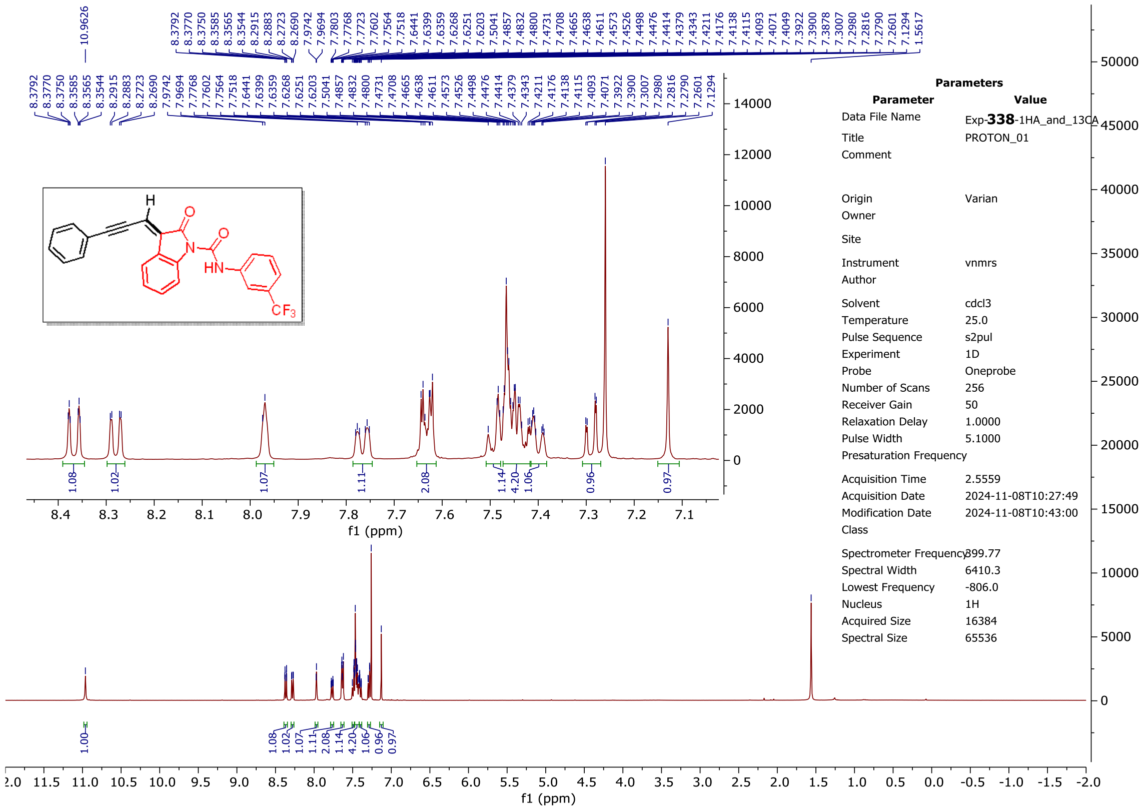

Figure S120. ^{1}H **NMR (400 MHz,** $CDCl_3$**) spectra of compound (11d)**

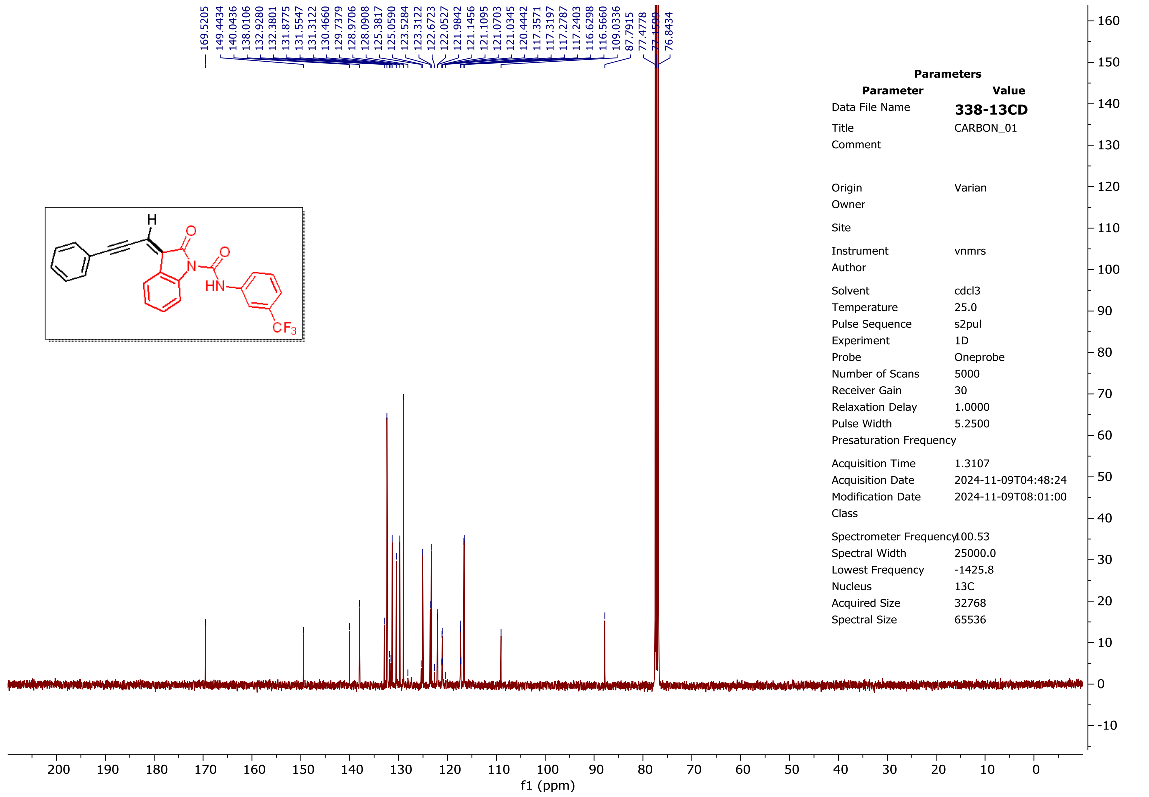

Figure S121. ^{13}C **NMR (100 MHz,** $CDCl_3$**) spectra of compound (11d)**

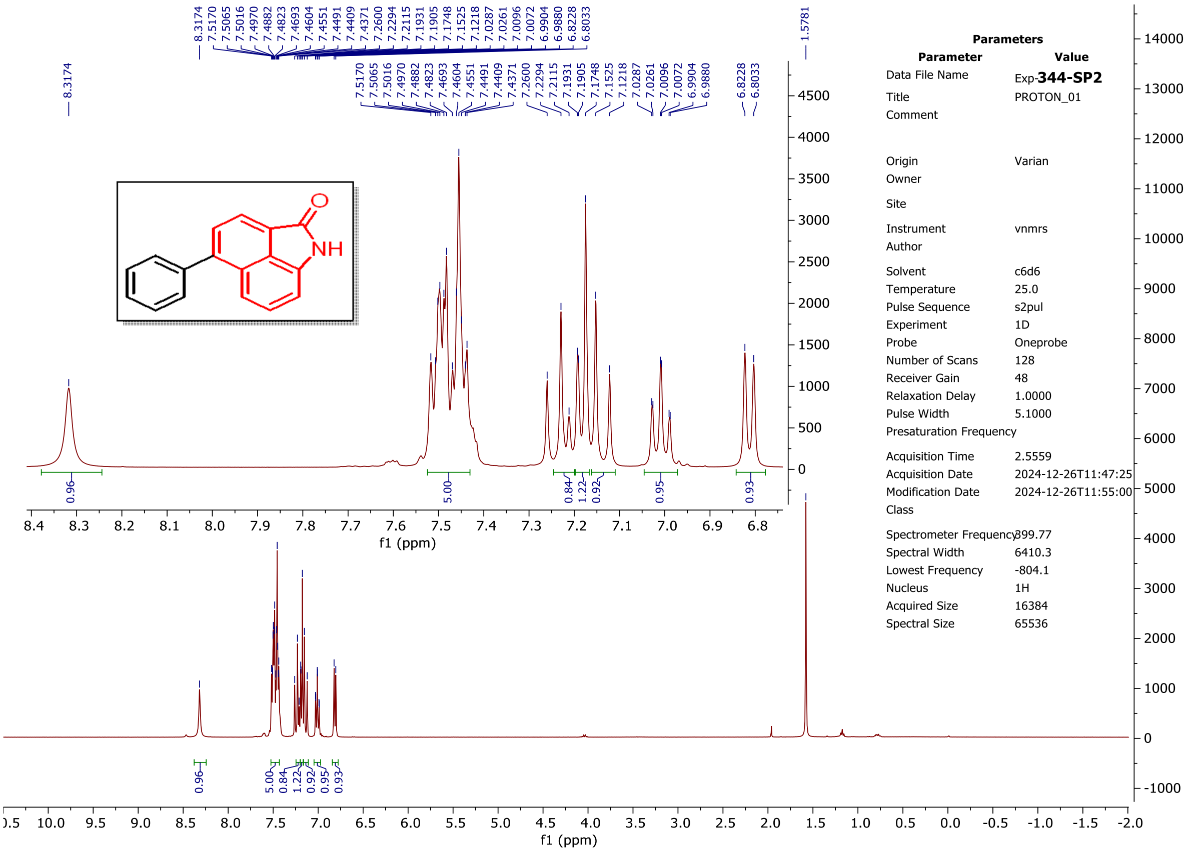

Figure S122. ^{1}H **NMR (400 MHz,** $CDCl_3$**) spectra of compound (12)**

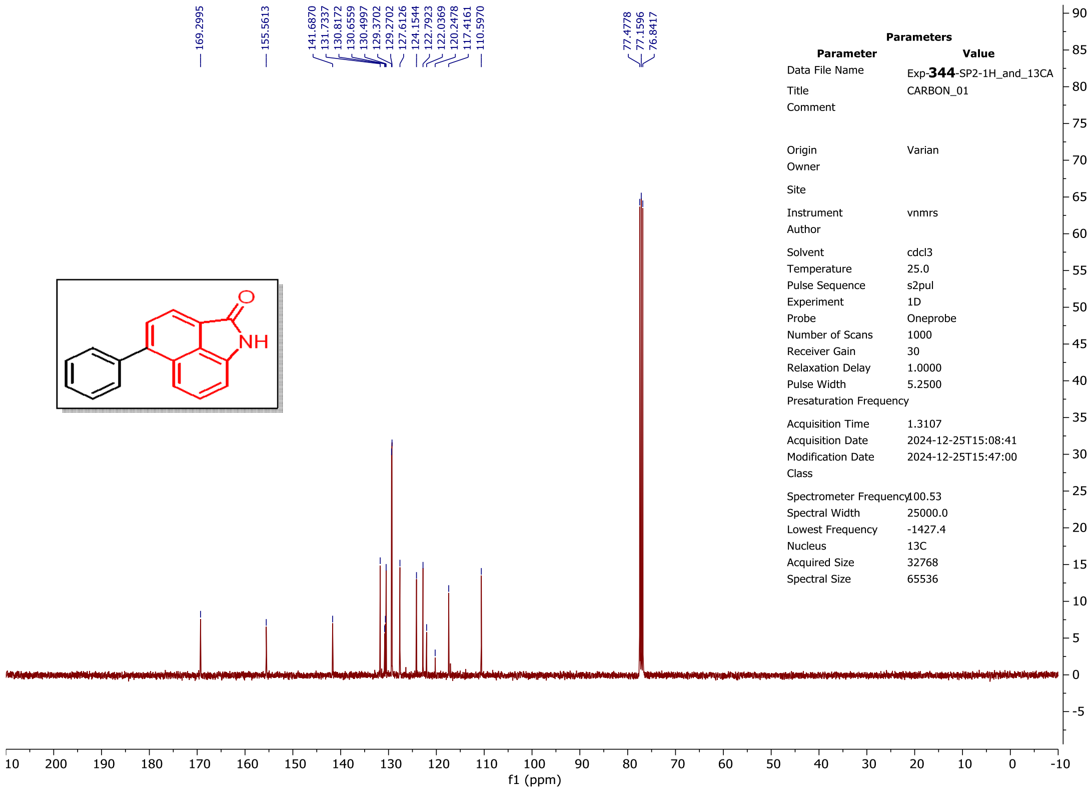

Figure S123. ^{13}C NMR (100 MHz, $CDCl_3$) spectra of compound (12)

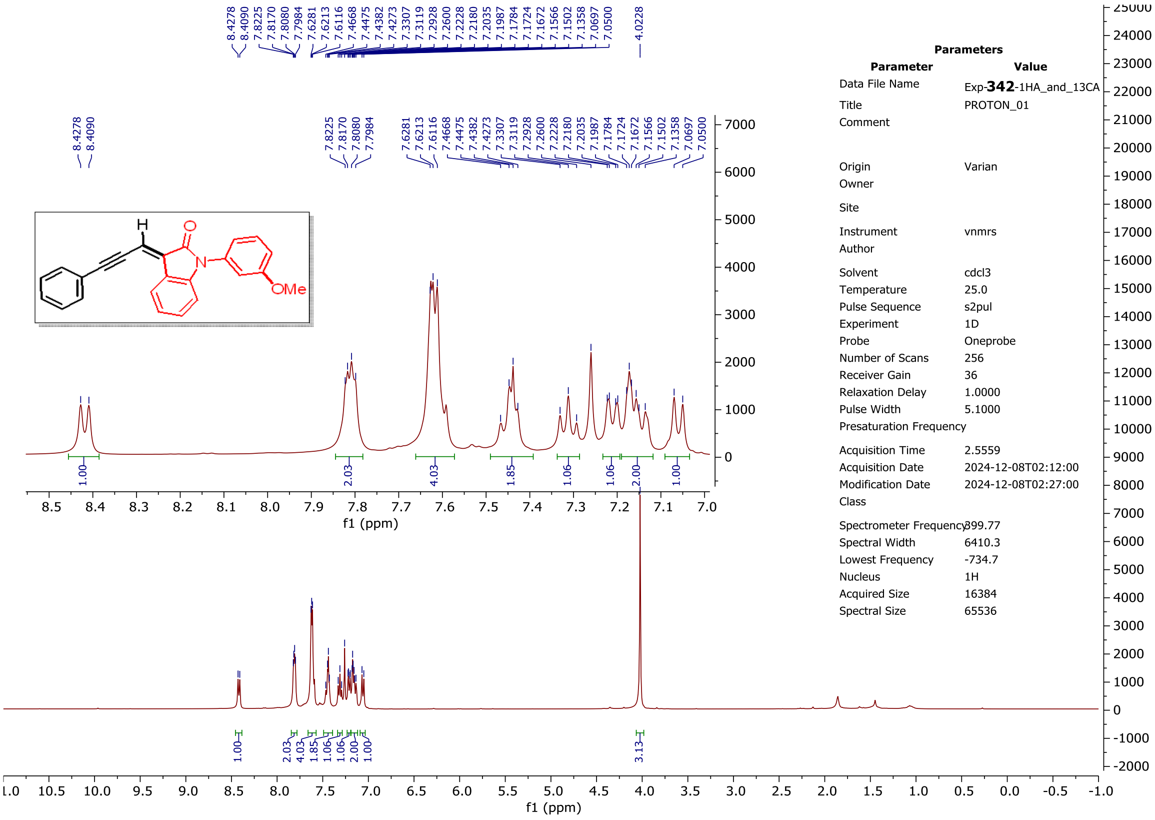

Figure S124. ^{1}H NMR (400 MHz, $CDCl_3$) spectra of compound (13a)

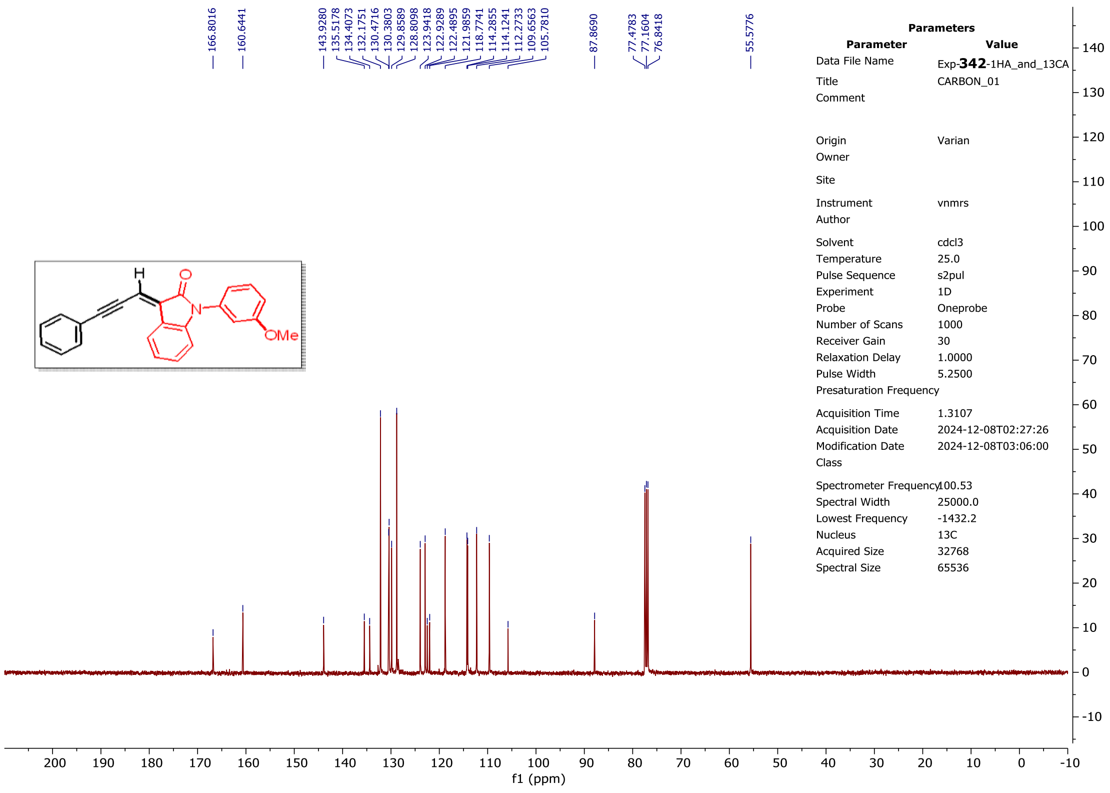

Figure S125. ^{13}**C NMR (100 MHz, CDCl$_3$) spectra of compound (13a)**

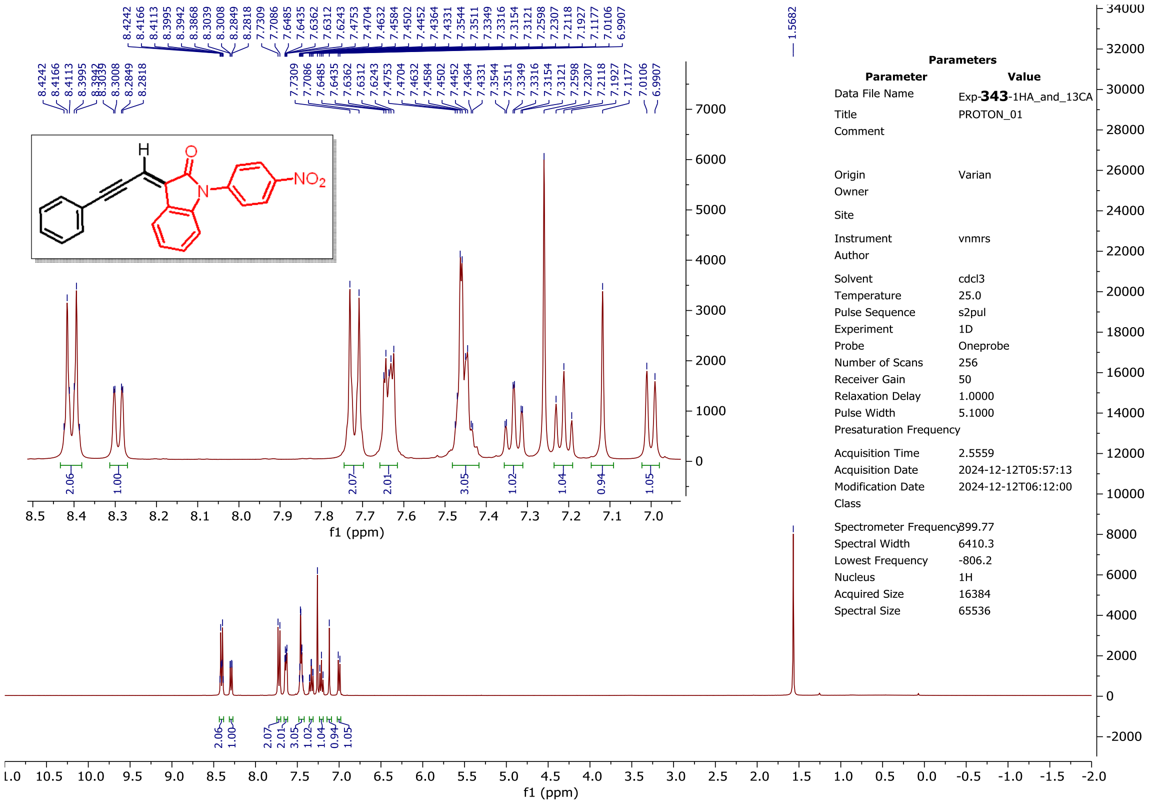

Figure S126. ^{1}**H NMR (400 MHz, CDCl$_3$) spectra of compound (13b)**

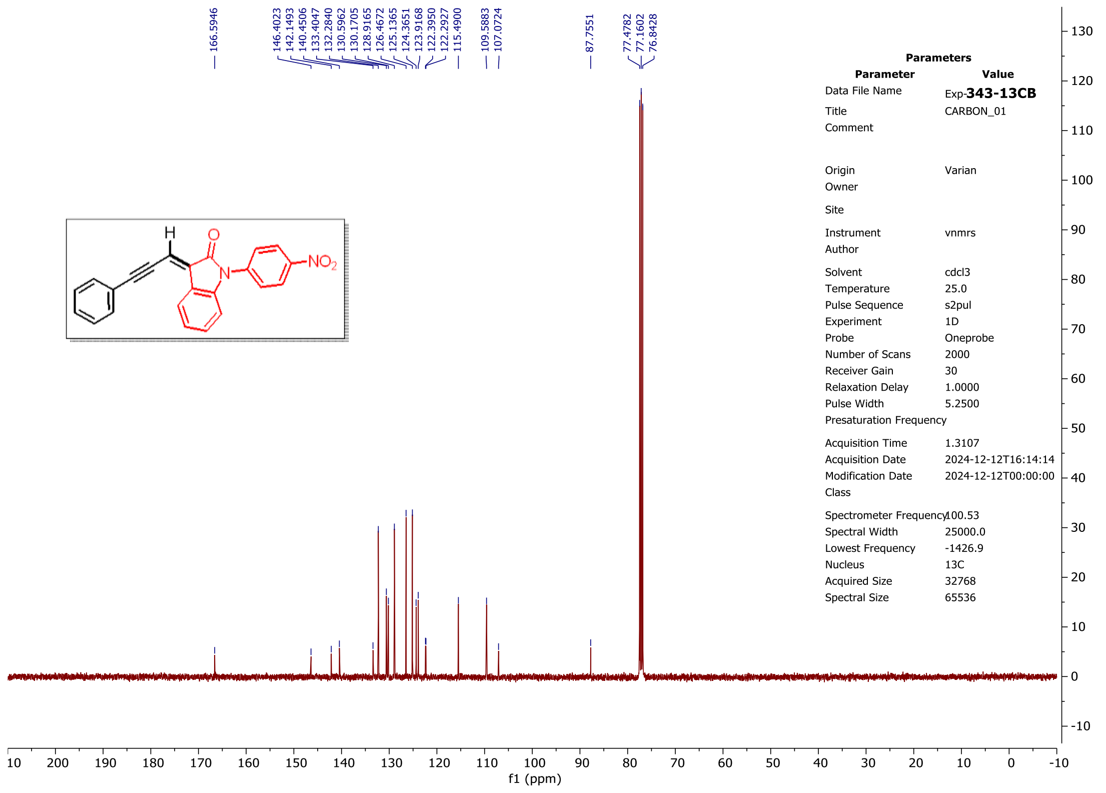

Figure S127. ^{13}C NMR (100 MHz, $CDCl_3$) spectra of compound (13b)

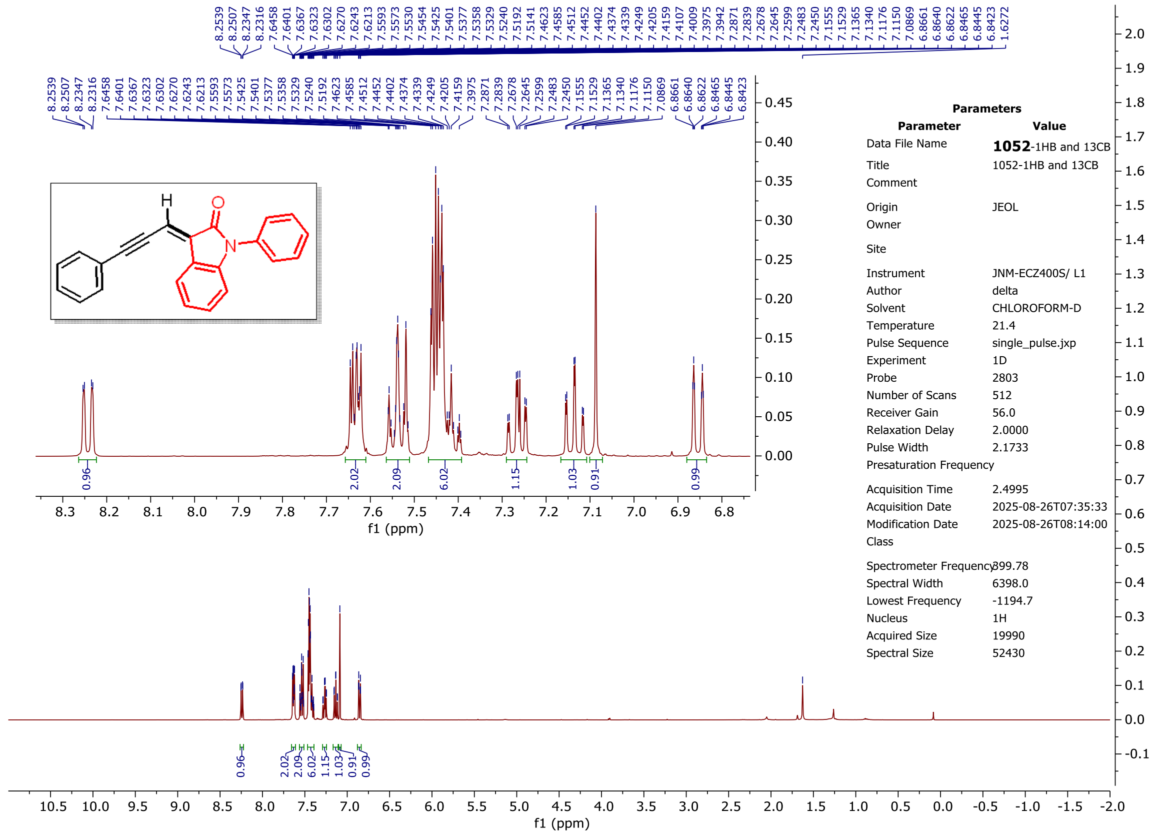

Figure S128. ^{1}H NMR (400 MHz, $CDCl_3$) spectra of compound (14a)

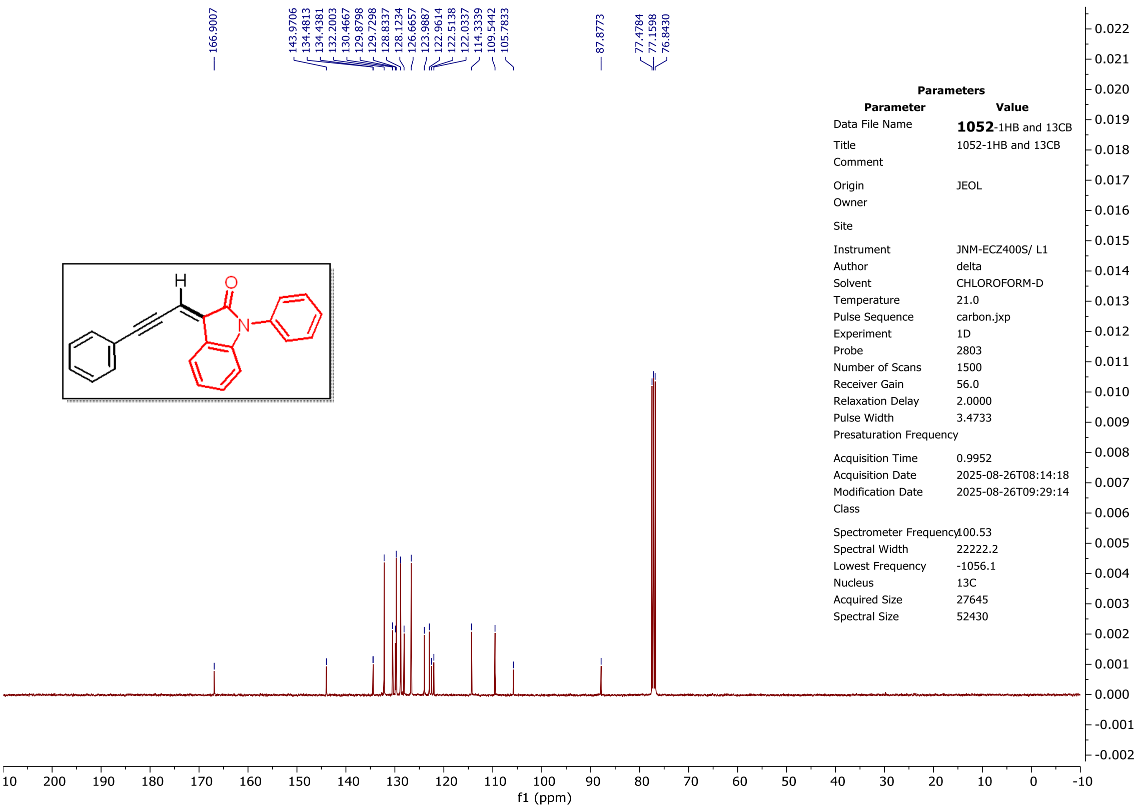

Figure S129. ^{13}**C NMR (100 MHz, CDCl$_3$) spectra of compound (14a)**

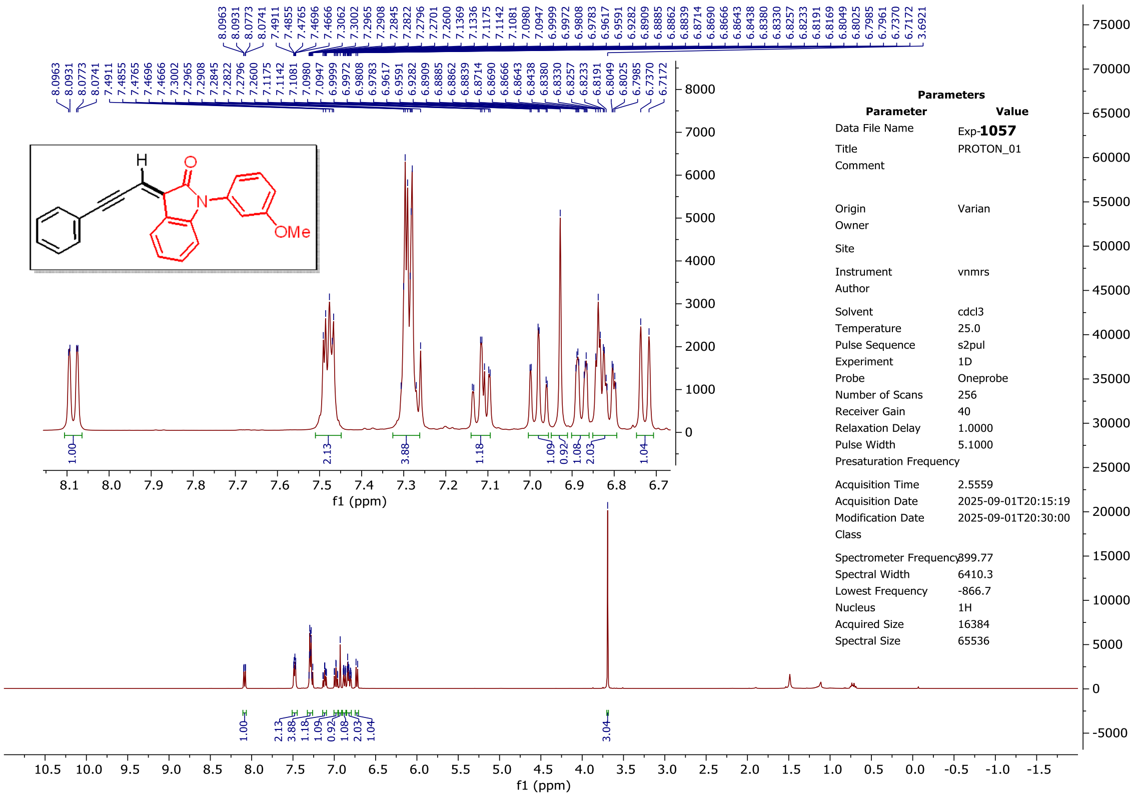

Figure S130. ^{1}**H NMR (400 MHz, CDCl$_3$) spectra of compound (14b)**

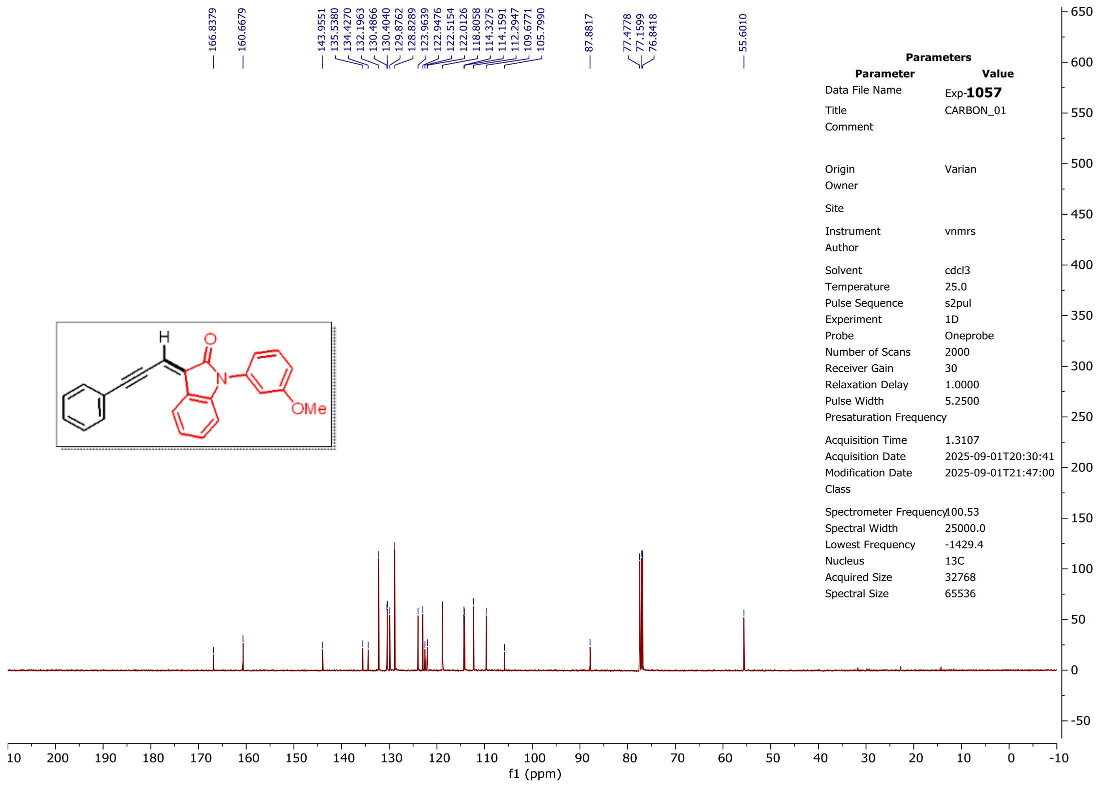

Figure S131. ^{13}C NMR (100 MHz, $CDCl_3$) spectra of compound (14b)

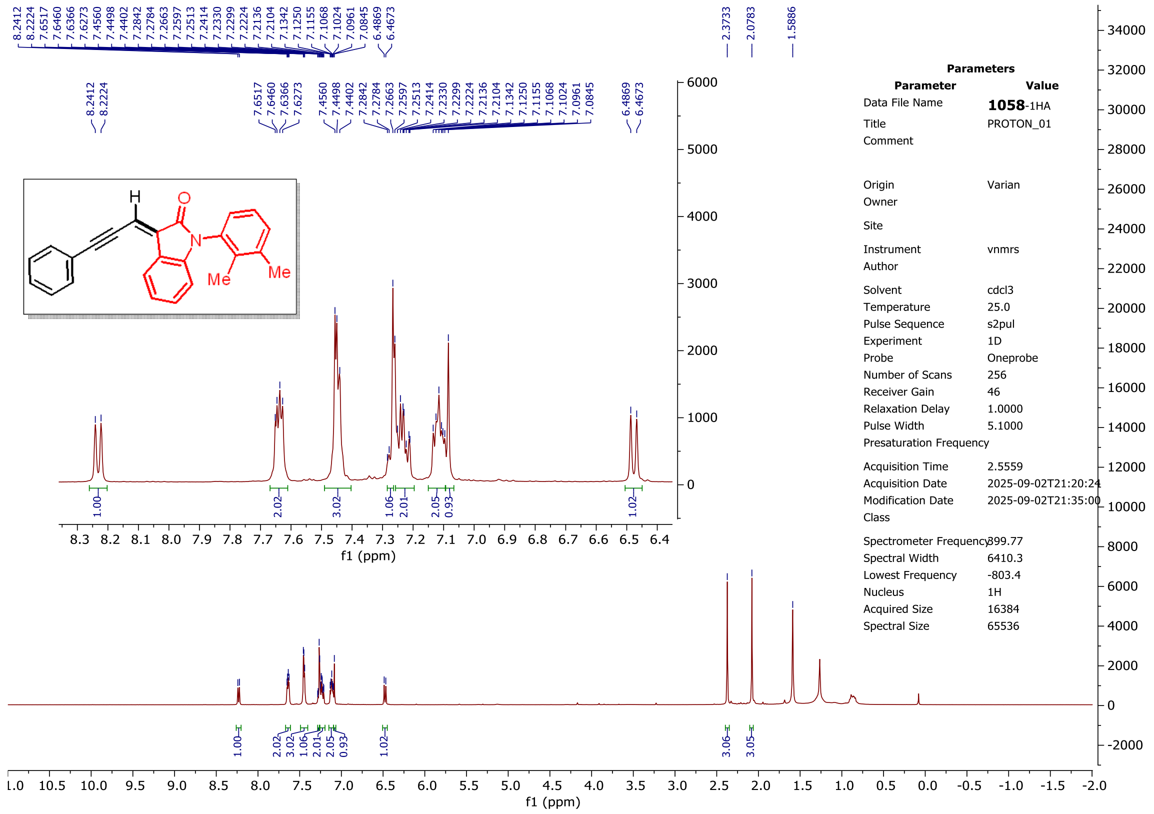

Figure S132. ^{1}H NMR (400 MHz, $CDCl_3$) spectra of compound (14c)

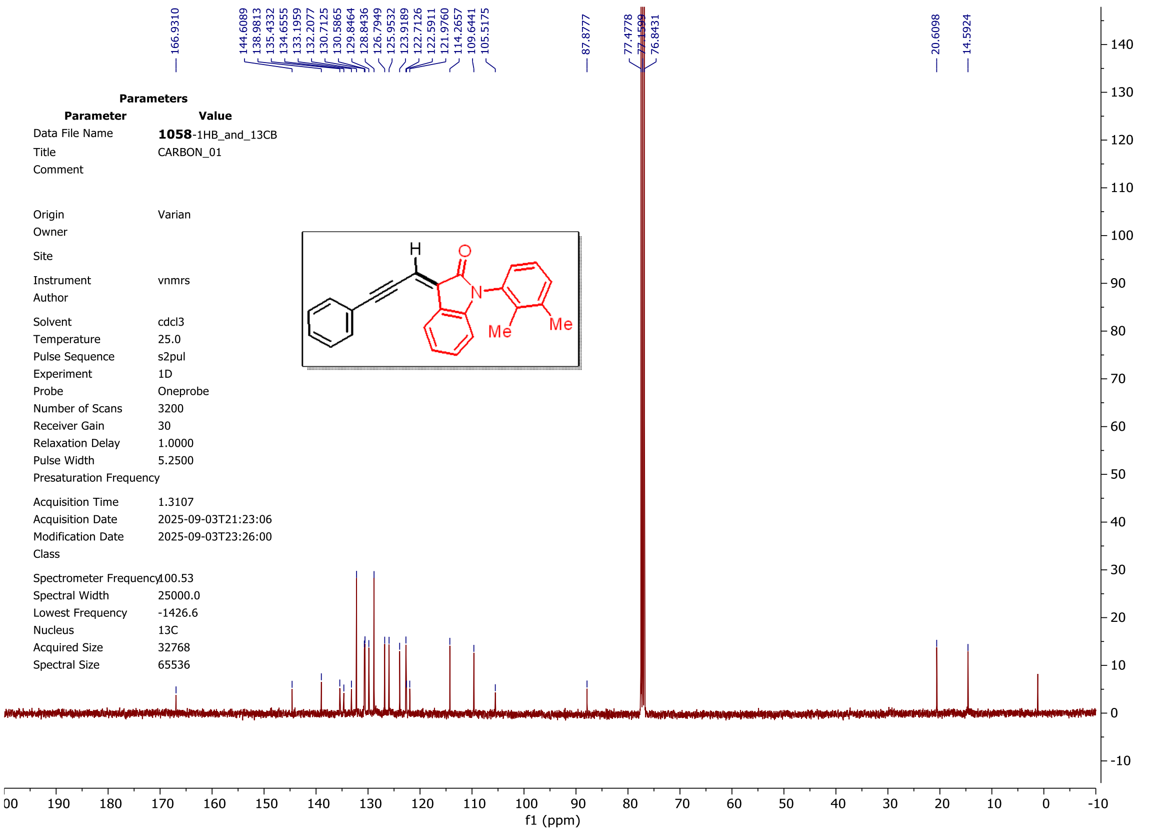

Figure S133. ^{13}C NMR (100 MHz, $CDCl_3$) spectra of compound (14c)

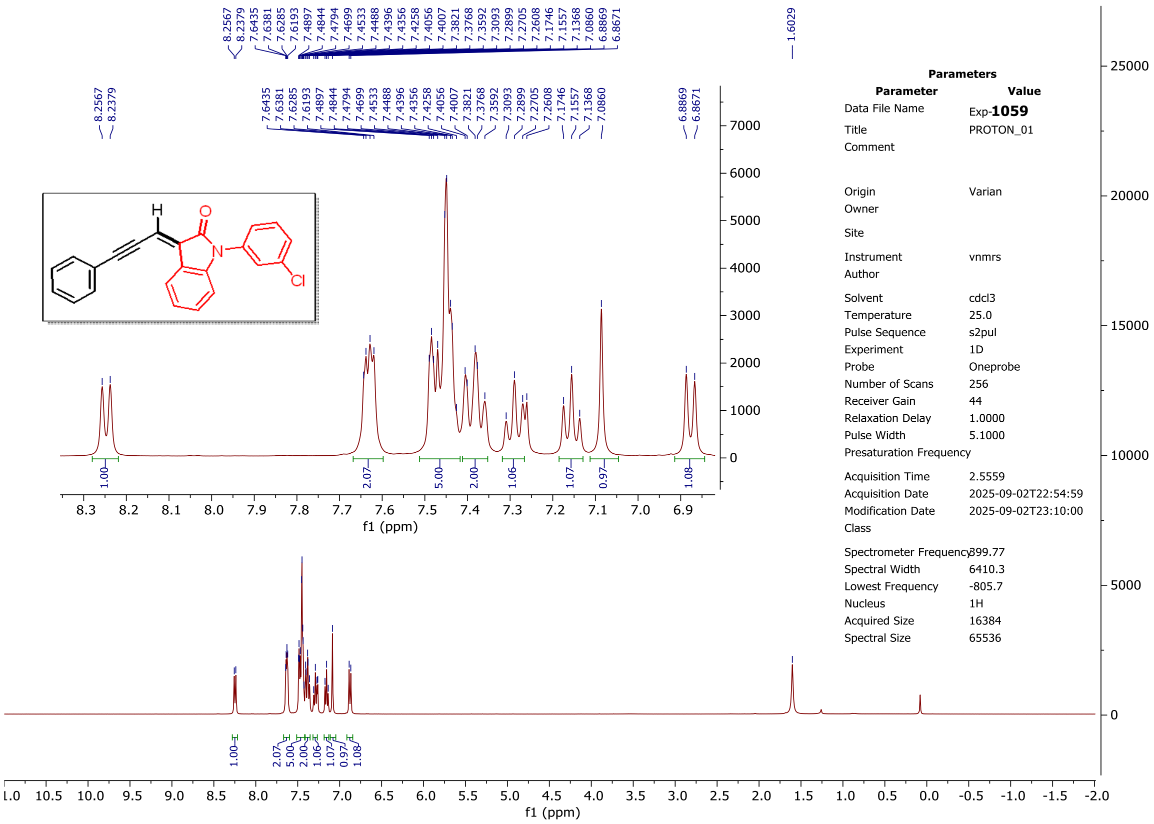

Figure S134. ^{1}H NMR (400 MHz, $CDCl_3$) spectra of compound (14d)

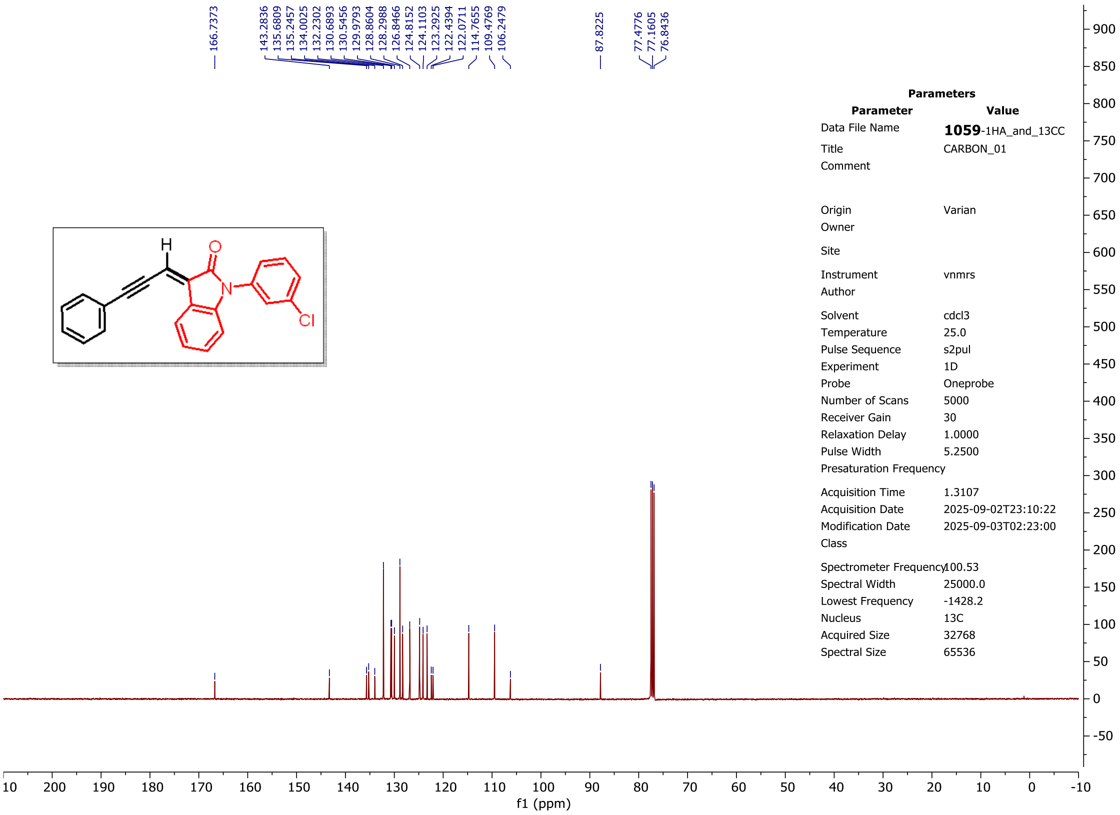

Figure S135. ^{13}C NMR (100 MHz, $CDCl_3$) spectra of compound (14d)

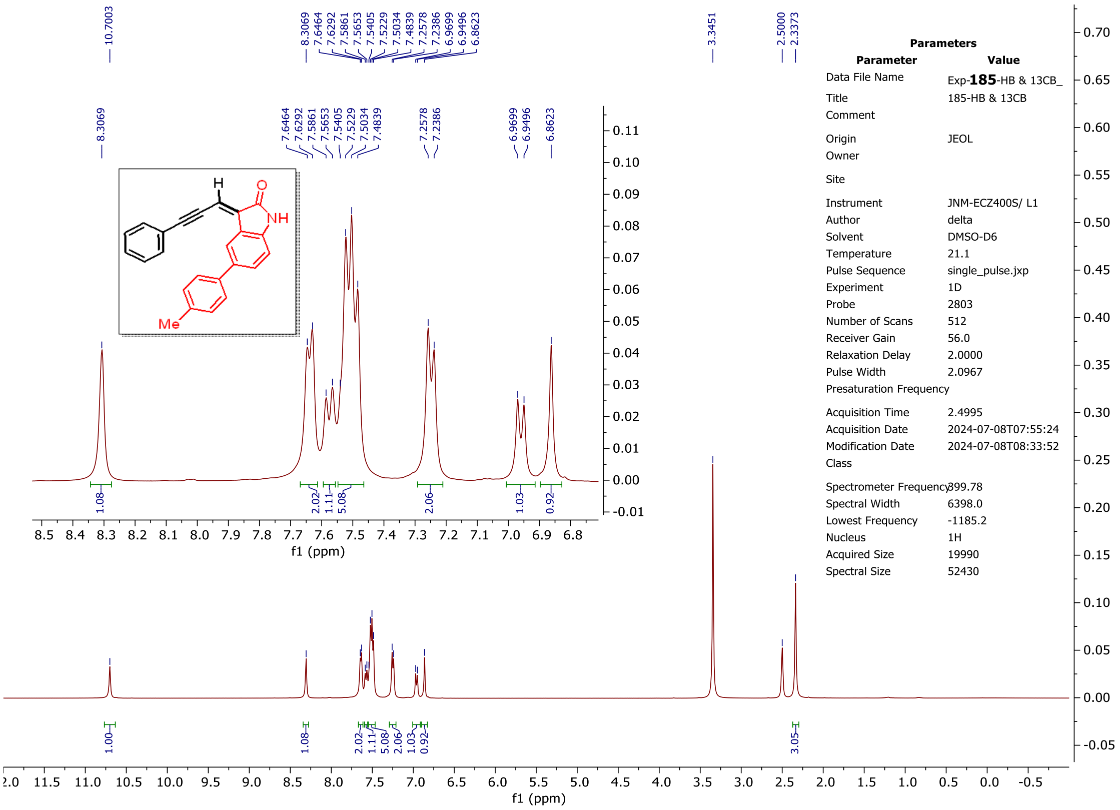

Figure S136. ^{1}H NMR (400 MHz, DMSO-d_6) spectra of compound (15)

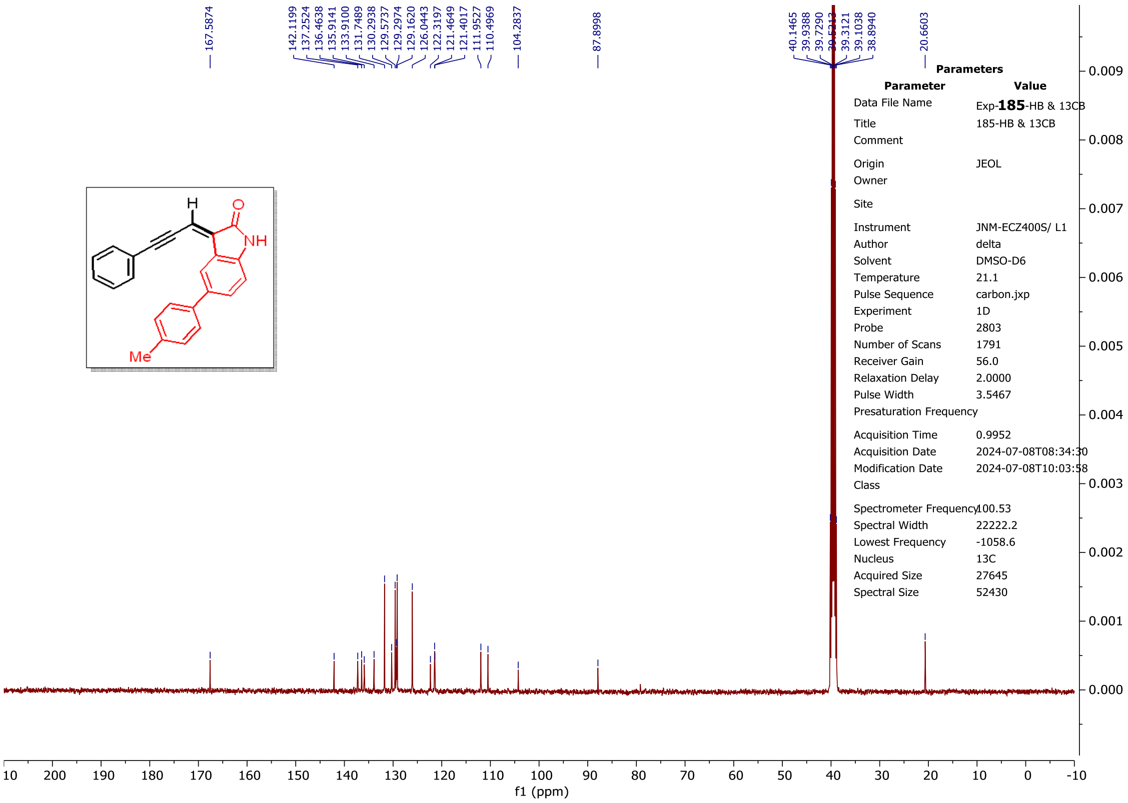

Figure S137. ^{13}C NMR (100 MHz, DMSO-d_6) spectra of compound (15)

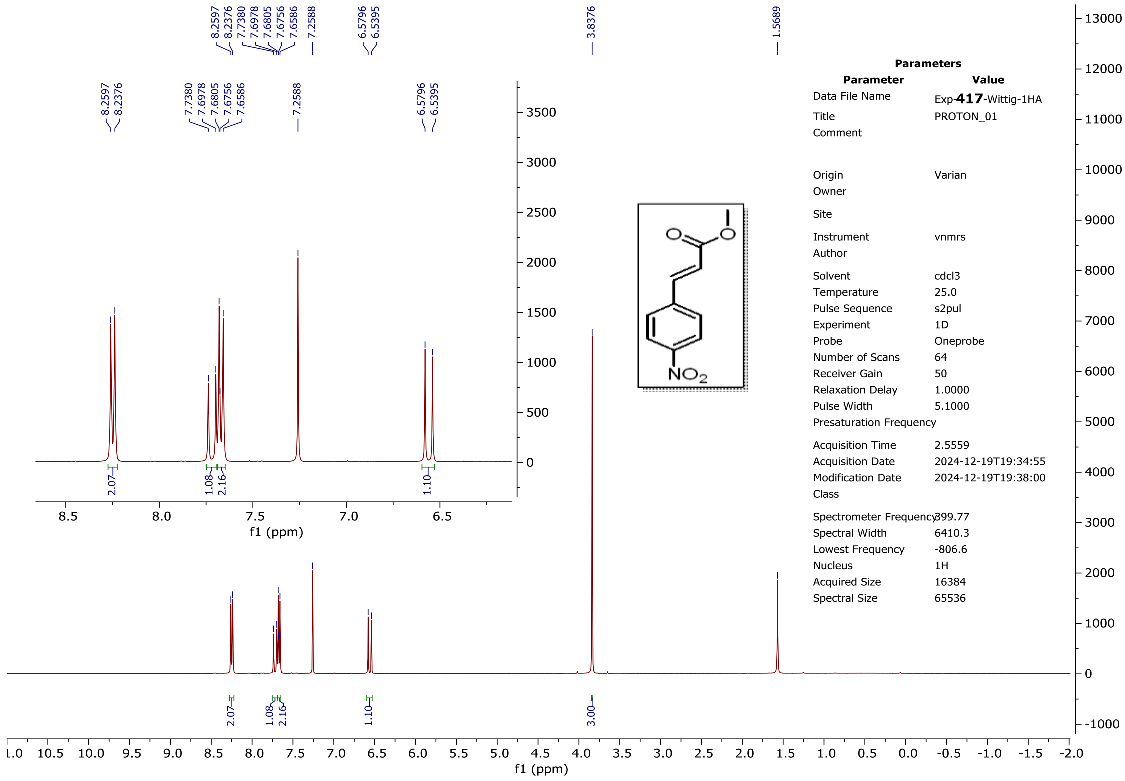

Figure S138. ^{1}H NMR (400 MHz, DMSO-d_6) spectra of compound (4a'')

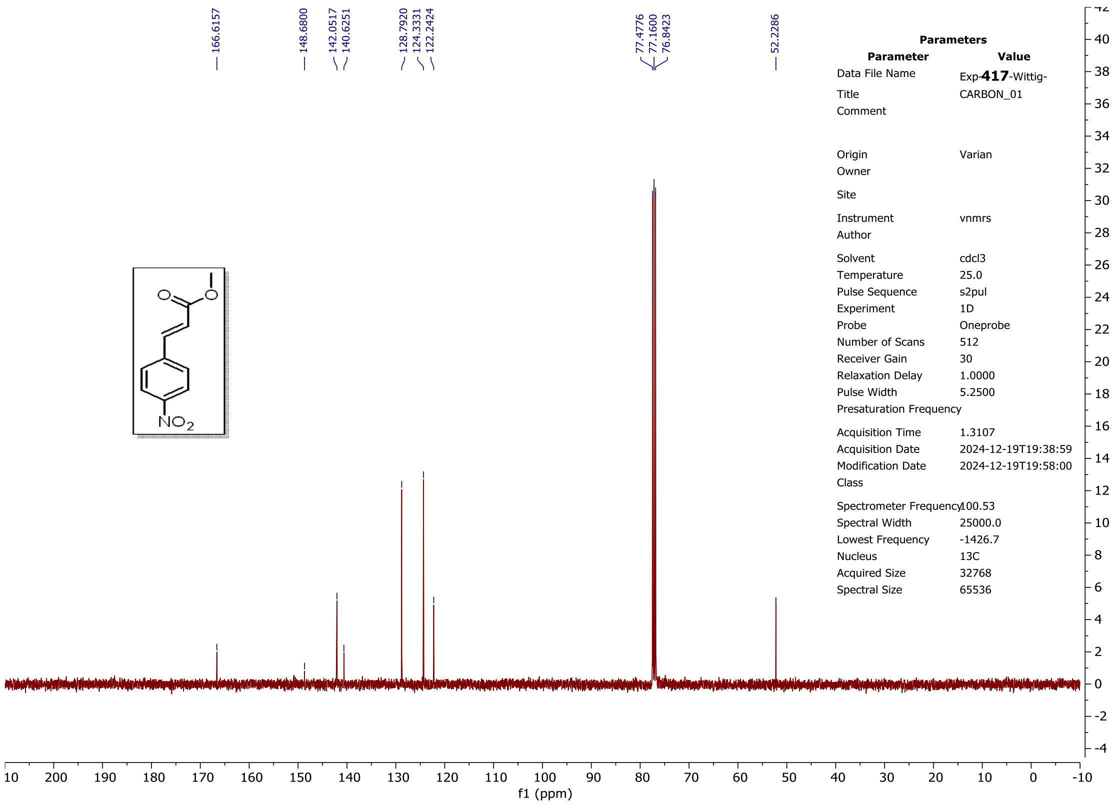

Figure S139. ^{13}C **NMR (100 MHz, DMSO-*d*$_6$) spectra of compound (4a'')**

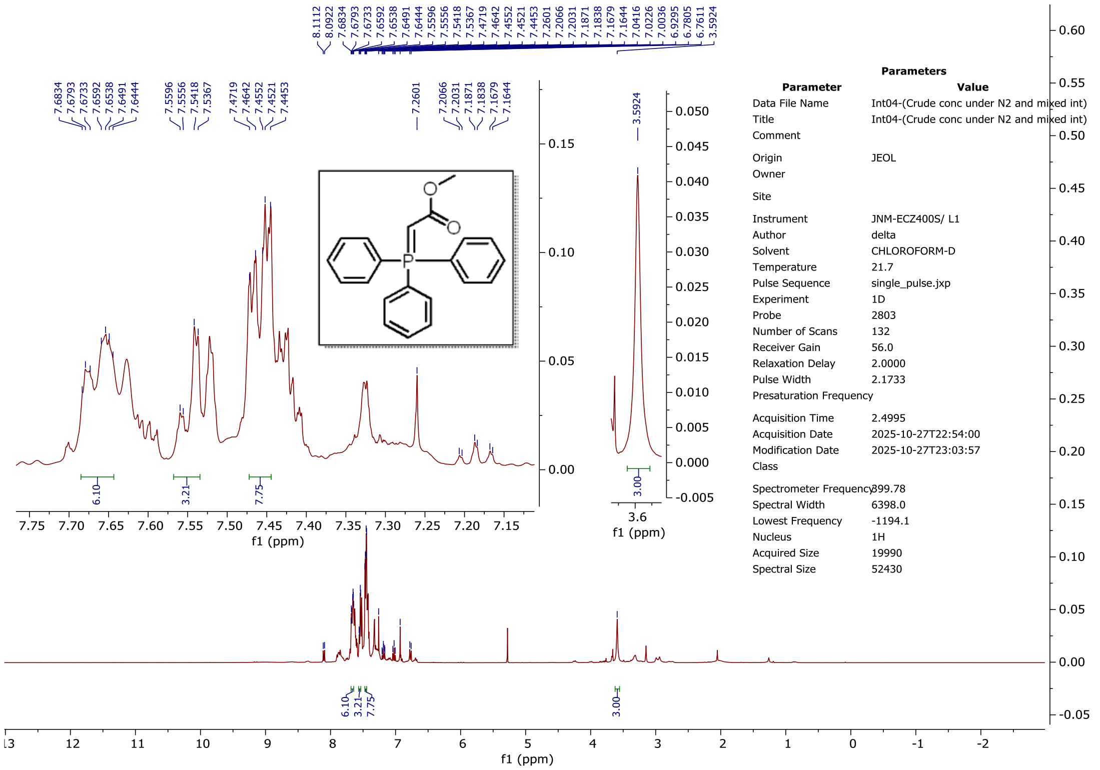

Figure S140. ^{1}H **NMR (400 MHz, CDCl$_3$) spectra of crude (4')**

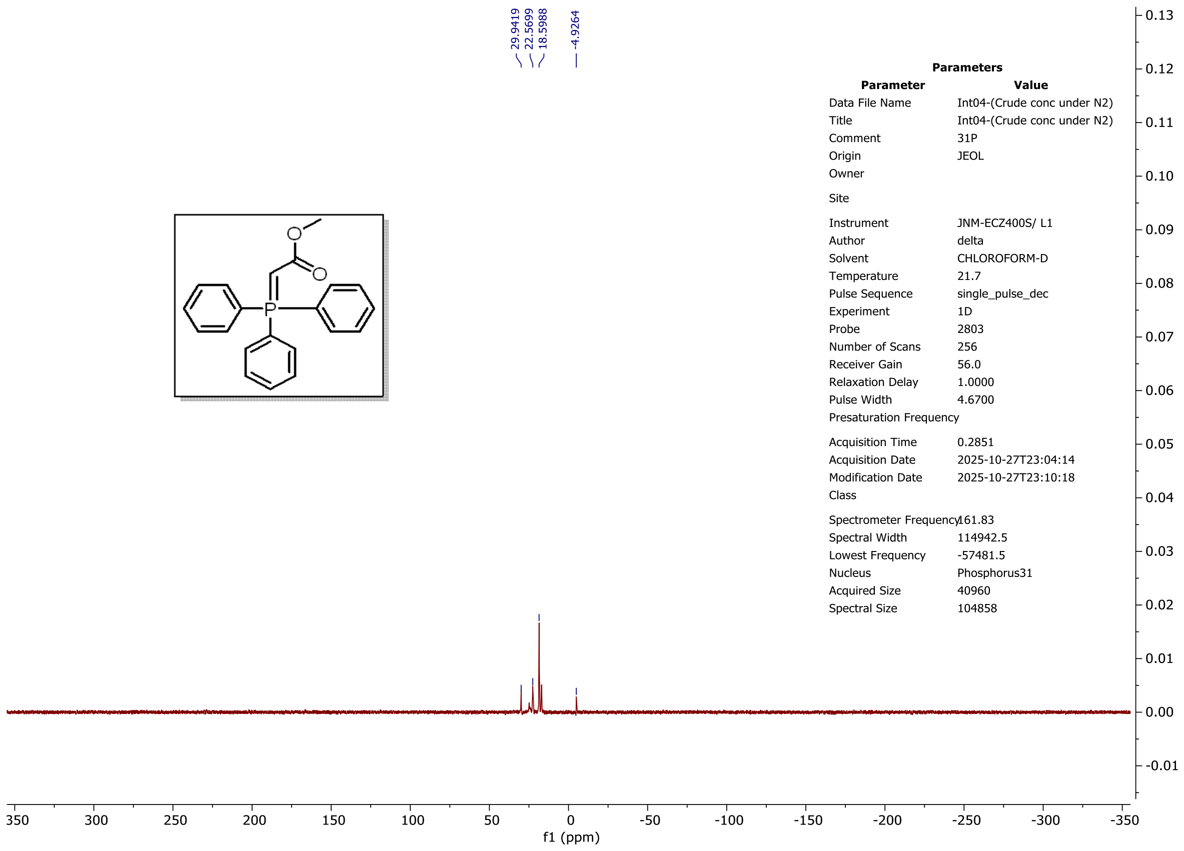

Figure S141. ^{31}P NMR (242.5 Hz, $CDCl_3$) spectra of crude (4')

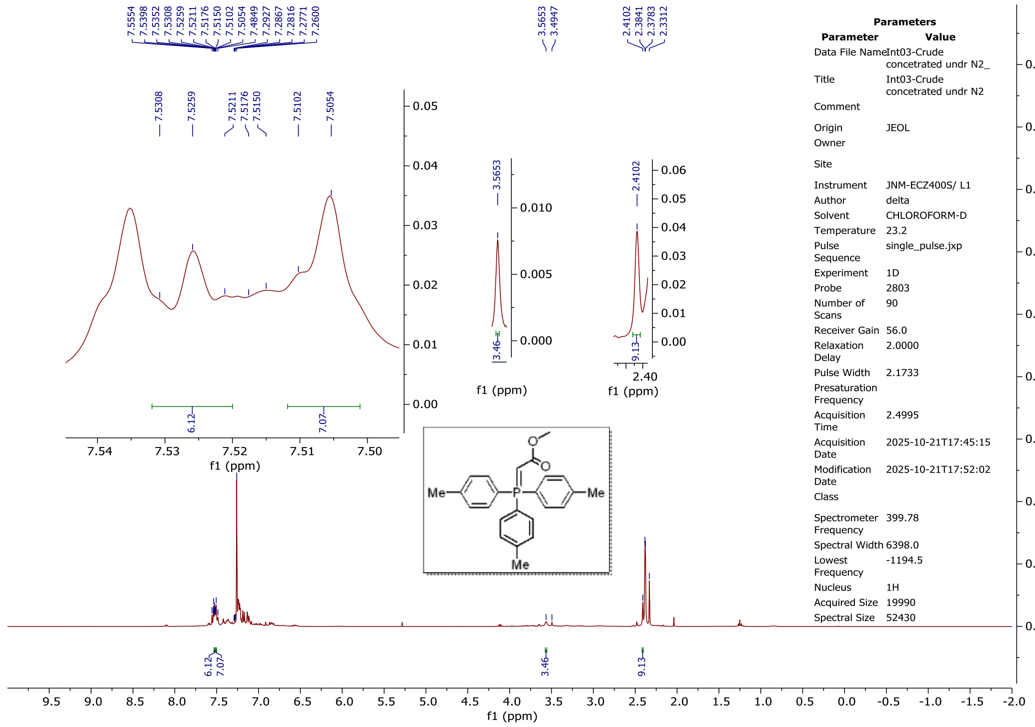

Figure S142. ^{1}H NMR (400 MHz, $CDCl_3$) spectra of crude (5')

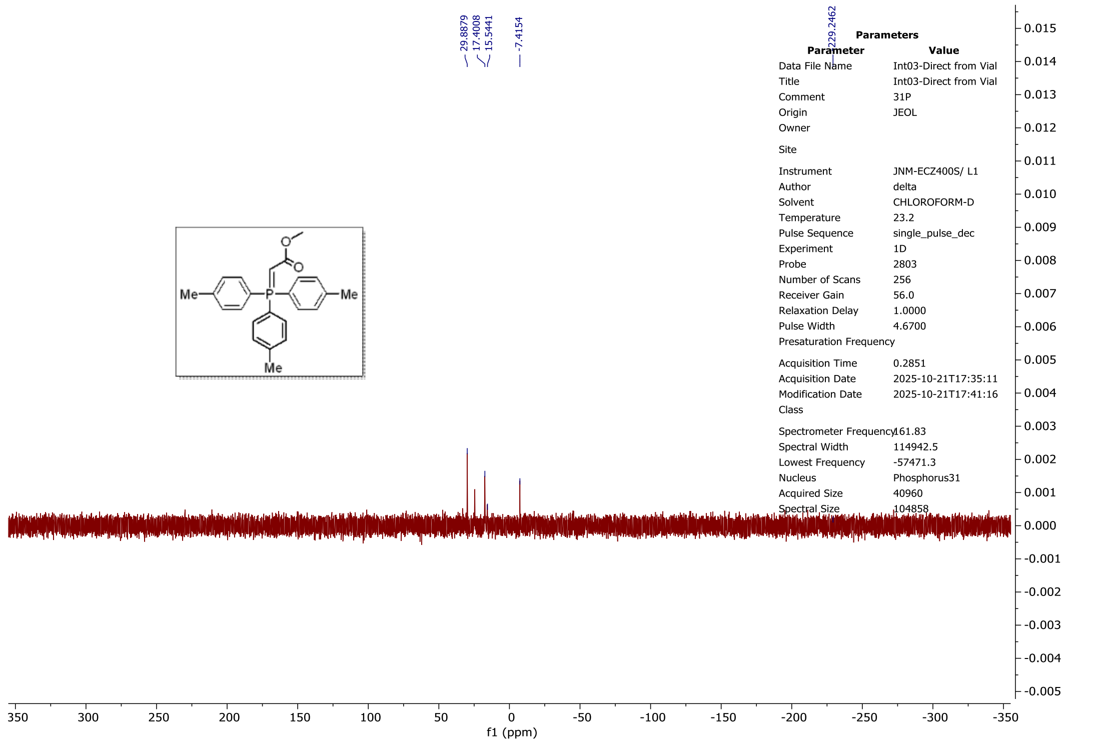

Figure S143. ^{31}**P NMR (242.5 Hz, CDCl$_3$) spectra of crude (5')**

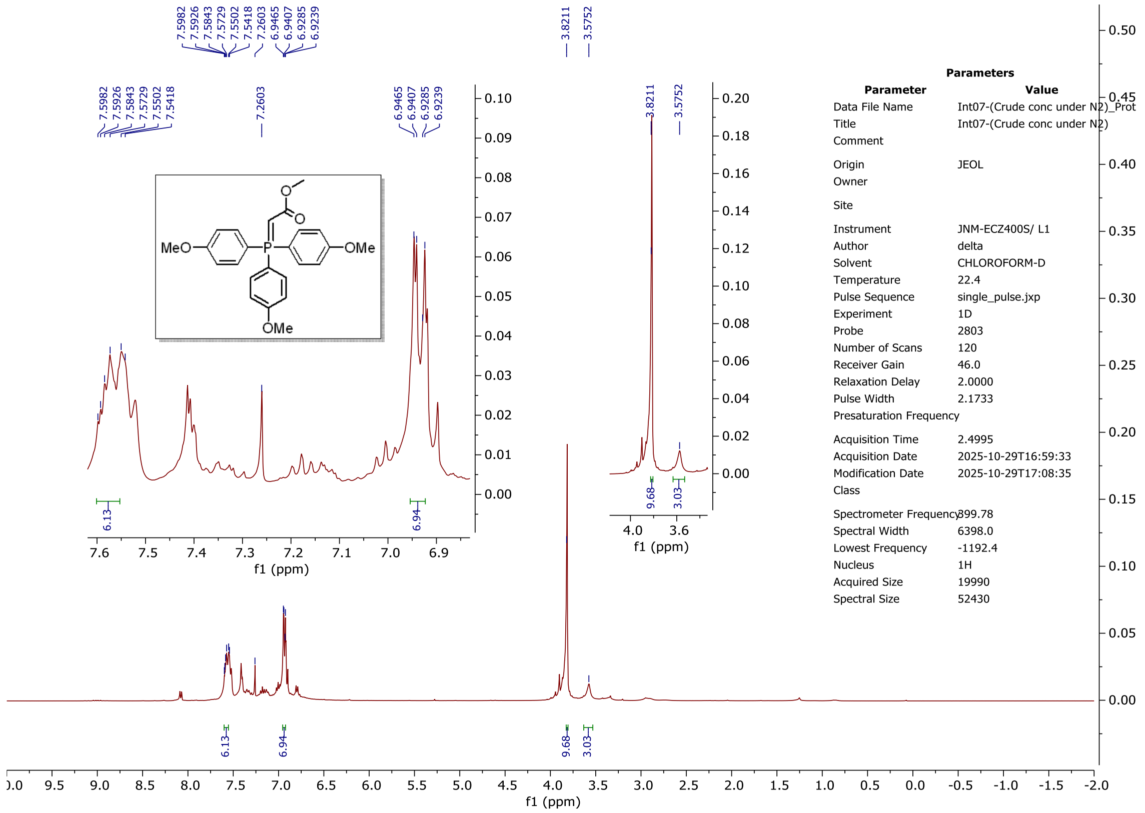

Figure S144. ^{1}**H NMR (400 MHz, CDCl$_3$) spectra of crude (6')**

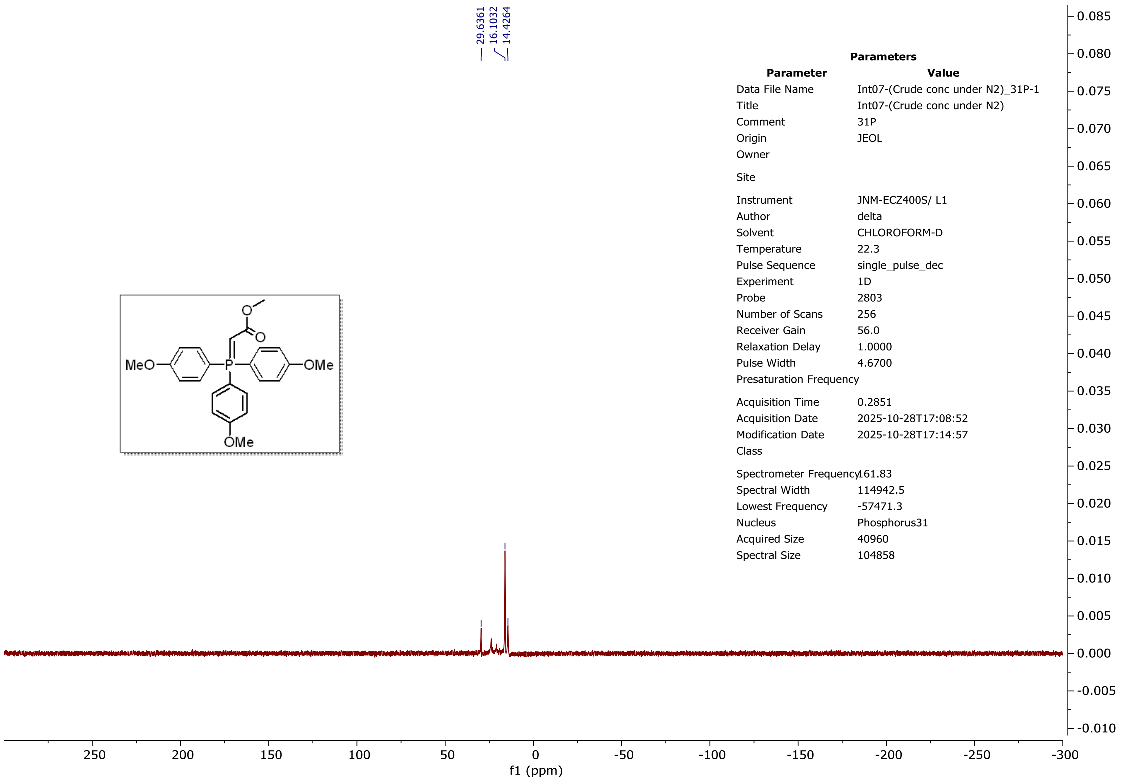

Figure S145. ^{31}P NMR (242.5 Hz, CDCl$_3$) spectra of crude (6')

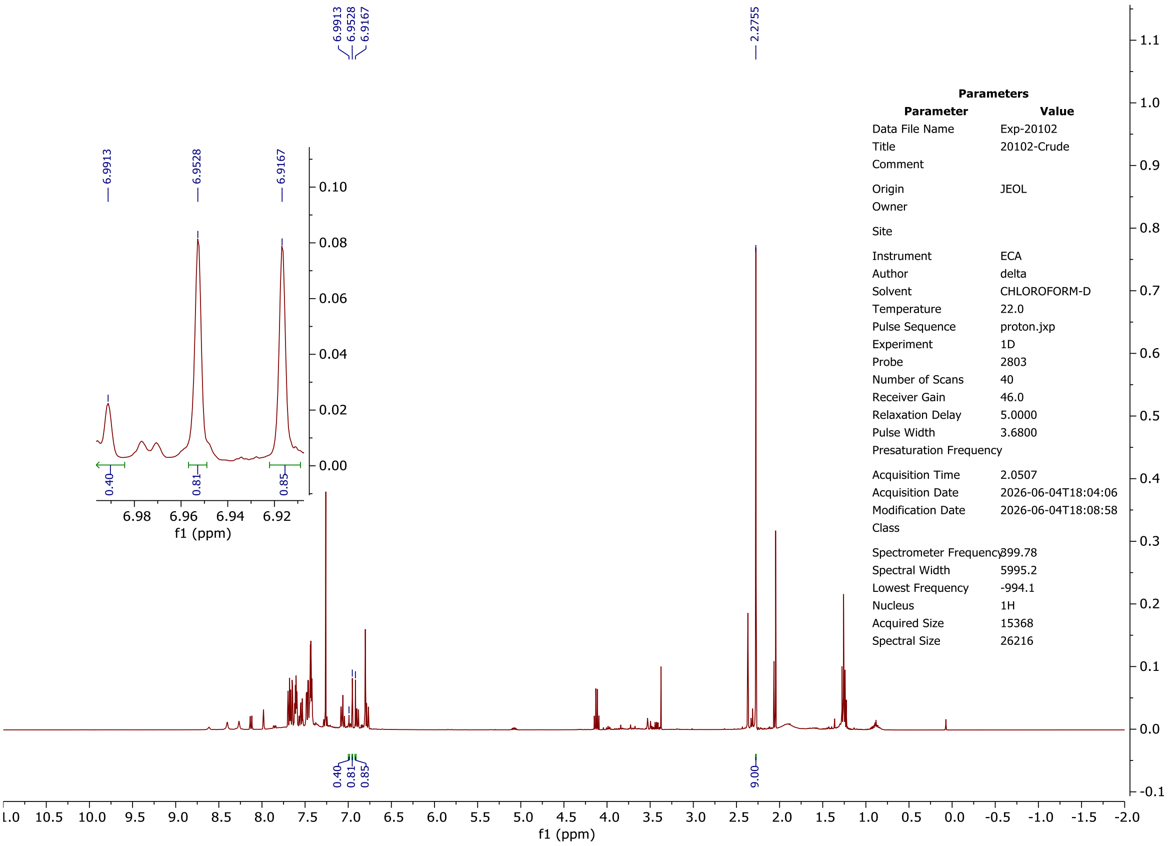

Figure S146. ^{1}H NMR (400 MHz, CDCl$_3$) spectra of crude crossover experiment

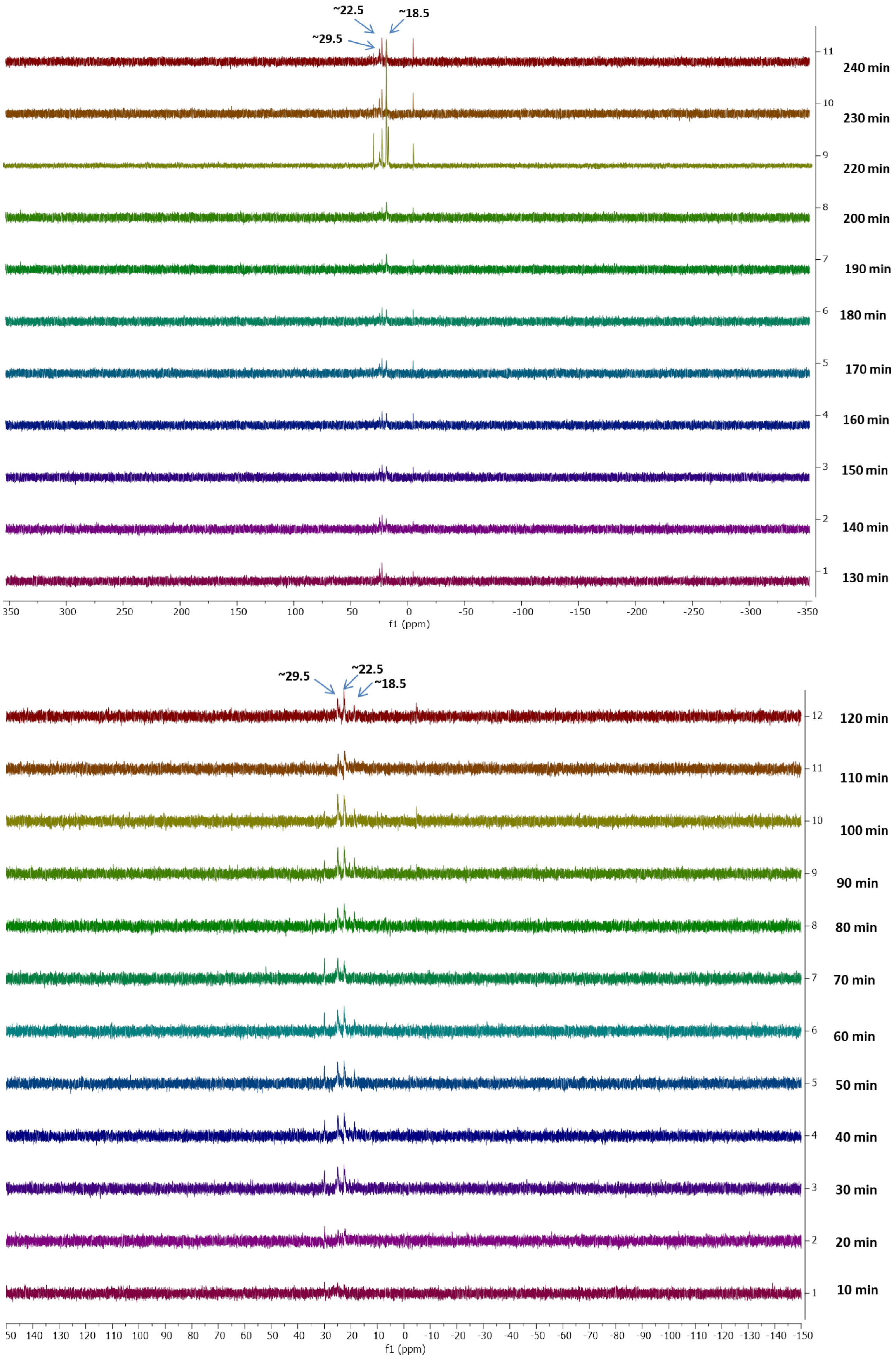

Figure S147. Time-dependent ^{31}P NMR (242.5 Hz, $CDCl_3$) spectra of model reaction showing evolution of ylide byproduct (4′)

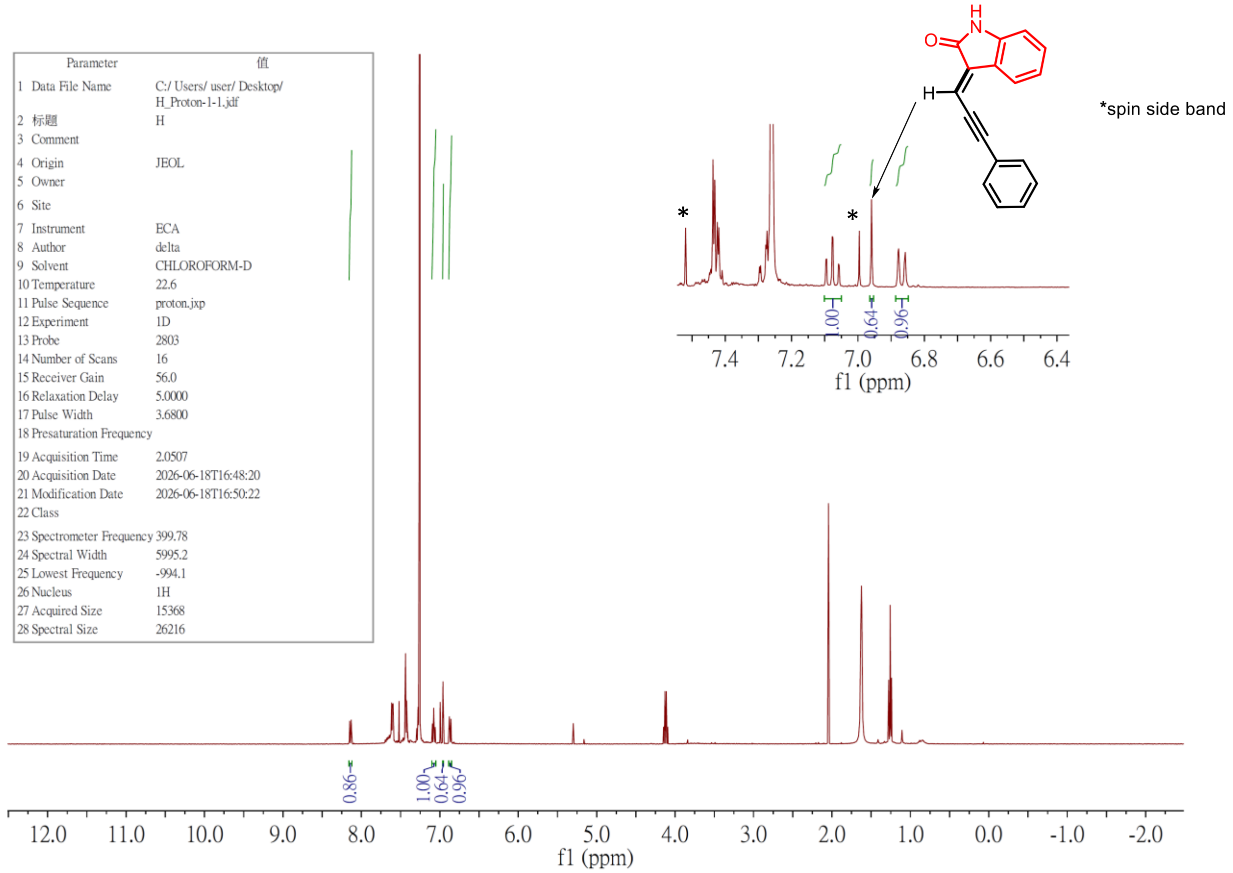

Figure S148. ^{1}H NMR of the reaction using 1a, D_3-2a, and PPh_3 in 3 mL DCM for 4 h.

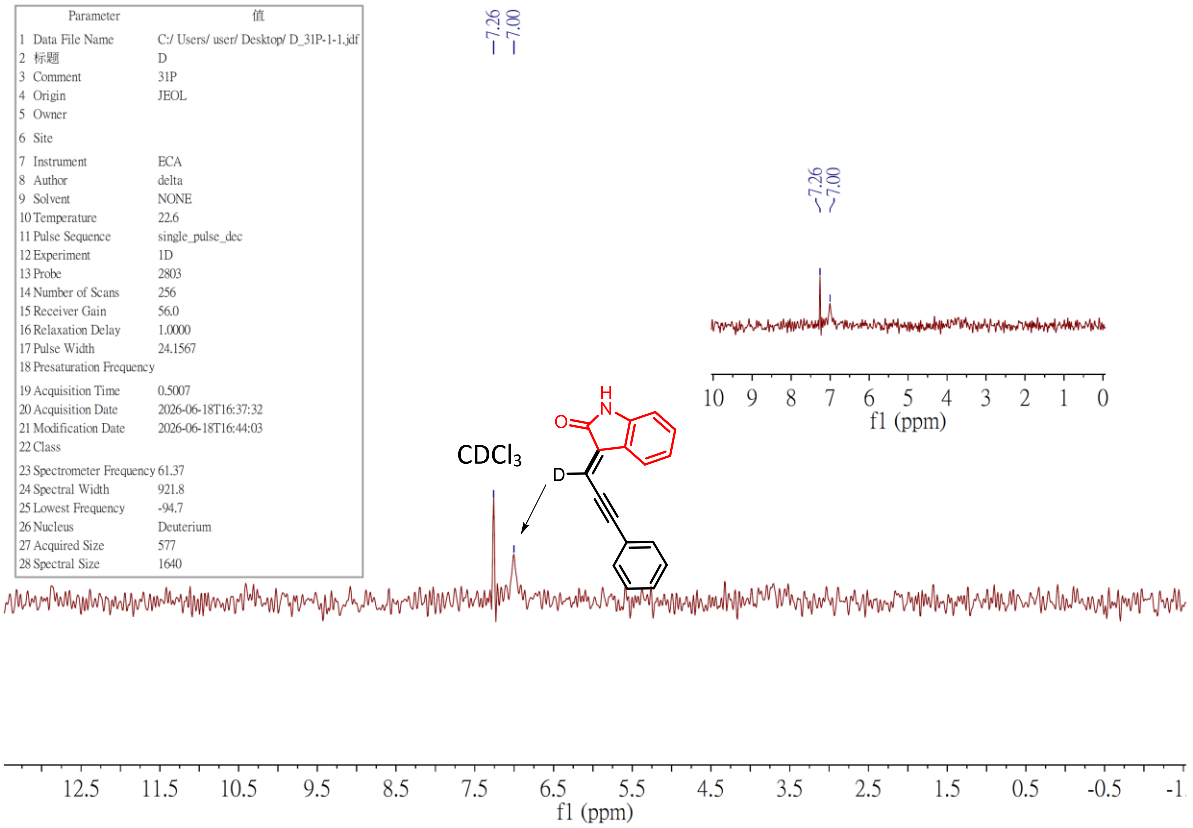

Figure S149. D NMR of the reaction using 1a, D_3-2a, and PPh_3 in 3 mL DCM for 4 h.

X-ray Crystallographic Analysis

General Crystal Growing Conditions: X-ray quality single crystals of **4e** were grown from a dichloromethane/hexane solvent system (7:3, v/v) at room temperature by slow evaporation.

X-ray structure for compound (4e)

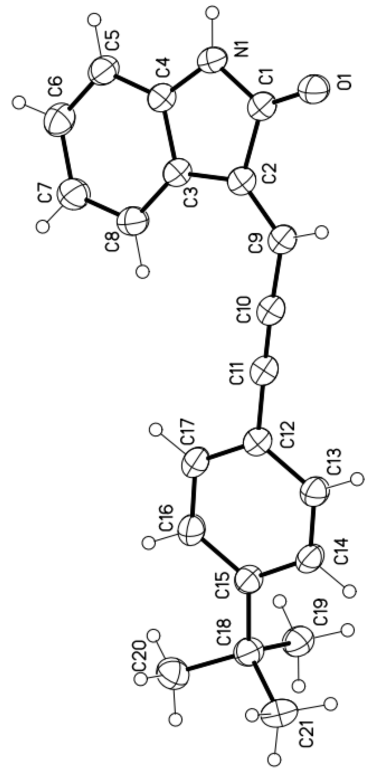

Structure of **4e** with 50% ellipsoid probability for non-H atoms.

X-Ray Data Collection and Structure Refinement Details:

Single crystals of compound **4e** ($C_{21}H_{19}NO$) were obtained and used for X-ray crystallographic analysis. A suitable crystal was selected and mounted on a loop using oil. Single-crystal X-ray diffraction data were collected on a Rigaku XtaLAB Synergy R (DW system) equipped with a HyPix-Arc 150 area detector using Mo Kα radiation. The crystal was kept at 100.00(12) K during data collection. Using Olex2 [1], the structure was solved with the SHELXT [2] structure solution program using Intrinsic Phasing and refined with the SHELXL [3] refinement package using Least Squares minimization.

1. Dolomanov, O.V., Bourhis, L.J., Gildea, R.J, Howard, J.A.K. & Puschmann, H. (2009), J. Appl. Cryst. 42, 339-341.
2. Sheldrick, G.M. (2015). Acta Cryst. A71, 3-8.
3. Sheldrick, G.M. (2015). Acta Cryst. C71, 3-8.

Table S2. Crystal data and structure refinement for compound **4e** (CCDC:2478849)

Identification code	2507091LT_auto
Empirical formula	$C_{21}H_{19}NO$
Formula weight	301.37
Temperature/K	100.00(12)
Crystal system	triclinic
Space group	P-1
a/Å	7.8940(3)
b/Å	9.1046(3)
c/Å	12.6388(5)
α/°	70.017(4)
β/°	75.411(4)
γ/°	75.706(3)
Volume/Å^3	813.13(6)
Z	2
ρ_{calc}g/cm^3	1.231
μ/mm^{-1}	0.585
F(000)	320.0
Crystal size/mm^3	0.21 × 0.19 × 0.14
Radiation	Cu Kα (λ = 1.54184)
2Θ range for data collection/°	7.572 to 146.12
Index ranges	$-8 \leq h \leq 9$, $-10 \leq k \leq 11$, $-14 \leq l \leq 15$
Reflections collected	8490
Independent reflections	3079 [R_{int} = 0.0215, R_{sigma} = 0.0258]
Data/restraints/parameters	3079/0/212
Goodness-of-fit on F^2	1.086
Final R indexes [I>=2σ (I)]	R_1 = 0.0424, wR_2 = 0.1172
Final R indexes [all data]	R_1 = 0.0475, wR_2 = 0.1208
Largest diff. peak/hole / e Å^{-3}	0.21/-0.18

Optimized coordinates of computed species

Optimized atomic coordinates of **Ia** (B3LYP/def2tzvp)

(N_{imag} = 0, Gibbs free energy = -1648.857830)

0 1

C 6.53652300 -0.26716300 -1.12307600

C 5.21013600 0.05048900 -1.36447000

C 4.40970900 0.62354000 -0.35221600

C 5.00923300 0.86473800 0.90306400

C 6.33958300 0.54905500 1.12445400

C 7.11605600 -0.02167200 0.11918100

H 7.12780500 -0.70602700 -1.91798800

H 4.77453600 -0.13478500 -2.33751400

H 4.41562400 1.30427400 1.69389800

H 6.77543400 0.74932700 2.09603100

H 8.15436500 -0.26762500 0.29950300

C 3.06412900 0.94488500 -0.58842700

C 1.87843400 1.21589800 -0.79654600

C 0.65070600 1.66512600 -1.07439700

C -0.64299300 1.38528500 -0.92773700

P -1.24233800 -0.10534100 -0.00015000

C -2.80952200 -0.91027700 -0.48720500

C -2.80309800 -2.06763800 -1.27376400

C -4.04049400 -0.35376900 -0.11411200

C -3.99705700 -2.65302500 -1.67806800

H -1.86930700 -2.52586600 -1.56514400

C -5.22768100 -0.94974800 -0.51218700

H -4.06641100 0.55415300 0.46806800

C -5.21184200 -2.09810200 -1.29794300

H -3.97219400 -3.54863700 -2.28578600

H -6.17003700 -0.50815400 -0.21381100

H -6.14152000 -2.55628300 -1.61100800

C 0.04282400 -1.37165500 -0.20595700

C 0.49010700 -2.13329600 0.87380300

C 0.56225100 -1.62073700 -1.48040200
C 1.43636900 -3.13313200 0.68193900
H 0.11373100 -1.94259300 1.86917000
C 1.50258700 -2.62311400 -1.66791700
H 0.24617300 -1.01927200 -2.32217000
C 1.94142700 -3.38094400 -0.58755600
H 1.78400300 -3.71065000 1.52877800
H 1.90480000 -2.80139500 -2.65679300
H 2.68501200 -4.15386800 -0.73426700
C -1.37154500 0.27437600 1.77364000
C -0.65906000 1.35798400 2.29337900
C -2.14250500 -0.51400100 2.63421000
C -0.71815200 1.64616200 3.65083700
H -0.06431300 1.97815500 1.63661700
C -2.19953200 -0.21960100 3.99028300
H -2.70420200 -1.35363500 2.24754100
C -1.48774300 0.86040900 4.50077400
H -0.16448900 2.49001900 4.04187500
H -2.80257800 -0.83386300 4.64681700
H -1.53582100 1.09049400 5.55761700
C -1.74011400 2.31764200 -1.24805300
O -2.84414100 2.24043900 -0.73677600
O -1.41391900 3.26509600 -2.13466800
C -2.43292200 4.23000500 -2.42439700
H -2.72787000 4.76278200 -1.52039500
H -1.98670300 4.91501400 -3.14044000
H -3.31137100 3.74796000 -2.85362000

Optimized atomic coordinates of **Ia′** (B3LYP/def2tzvp)

(N_{imag} = 0, Gibbs free energy = -1648.848246)

0 1
C 6.26166400 0.17932300 -0.71990400
C 4.87460100 0.15974900 -0.71016800

C 4.15059100 1.19706500 -0.10110400

C 4.86105800 2.25391500 0.49126500

C 6.24758300 2.26710500 0.47399100

C 6.95457700 1.23099900 -0.12876000

H 6.80503200 -0.62782400 -1.19557600

H 4.33588700 -0.65304800 -1.17894800

H 4.31018000 3.05850800 0.95982100

H 6.77969400 3.09053600 0.93383000

H 8.03702500 1.24471900 -0.14009700

C 2.72832900 1.18758300 -0.08349500

C 1.51912300 1.21818900 -0.05827900

C 0.09572000 1.26518000 -0.03772600

C -0.61812100 2.38314000 0.01494900

P -0.71651100 -0.40268100 0.03887300

C -1.96031500 2.76575400 0.18120800

O -2.54265200 2.82953700 1.26464900

O -2.54666700 3.21516800 -0.97446900

C -3.84456700 3.79052800 -0.81936500

H -4.55441600 3.06744800 -0.41540800

H -3.81188400 4.65484500 -0.15512200

H -4.14949200 4.09773900 -1.81785800

C -2.25824100 -0.36203000 -0.90957300

C -2.28781500 0.28922100 -2.14597200

C -3.39755600 -1.02912100 -0.45352000

C -3.43929500 0.25618900 -2.92060300

H -1.42282600 0.83817000 -2.49163100

C -4.54805200 -1.05424900 -1.23123200

H -3.39236900 -1.51930500 0.51037700

C -4.56816800 -0.41636000 -2.46630900

H -3.45685900 0.76952400 -3.87313900

H -5.42939000 -1.56701800 -0.86811100

H -5.46692100 -0.43428900 -3.06990000

C -1.10645100 -0.85392200 1.75481500

C -1.07069100 -2.18890100 2.17556700

C -1.48505800 0.14584200 2.65757500
C -1.39712300 -2.51746000 3.48437500
H -0.78436300 -2.97237400 1.48754800
C -1.81287100 -0.19590000 3.96431300
H -1.55437400 1.18113500 2.34502500
C -1.76570800 -1.52057800 4.38148900
H -1.36178200 -3.55193000 3.80165500
H -2.10657400 0.58388500 4.65504600
H -2.01666100 -1.77766900 5.40302100
C 0.30031400 -1.75197500 -0.63482400
C -0.03325200 -2.39308000 -1.83094300
C 1.47973500 -2.11663100 0.02720900
C 0.79279300 -3.38254200 -2.35132700
H -0.93978000 -2.12798600 -2.35658900
C 2.29694900 -3.10907100 -0.49386800
H 1.76148300 -1.62572800 0.94864400
C 1.95683200 -3.74346800 -1.68484800
H 0.52060900 -3.87300400 -3.27716700
H 3.20327100 -3.38398100 0.03030000
H 2.59722800 -4.51675000 -2.08986800

1a (N_{imag} = 0; Gibbs free energy = -612.348513)

0 1
C 4.80620600 -1.11552000 0.00005000
C 3.41619200 -1.18370500 0.00008400
C 2.65395500 0.00370300 -0.00002400
C 3.30982200 1.25317100 -0.00016800
C 4.70050000 1.30633800 -0.00020500
C 5.45004300 0.12580000 -0.00009600
H 5.38914800 -2.03143500 0.00013400
H 2.90903000 -2.14291300 0.00019500
H 2.72129000 2.16474700 -0.00025000
H 5.20148400 2.26951000 -0.00031800
H 6.53494100 0.17315000 -0.00012400

C 1.23537600 -0.05840100 0.00000800

C 0.01484500 -0.11033600 0.00003600

C -1.34150500 -0.16609600 0.00007300

C -2.56047200 -0.20610500 0.00011200

C -3.99498000 -0.35107800 0.00019800

O -4.56661600 -1.42666600 -0.00021900

O -4.60550700 0.84474400 0.00022600

C -6.05174100 0.80220500 0.00000600

H -6.37065000 1.84411200 -0.00003200

H -6.41871500 0.28918000 -0.89255200

H -6.41898000 0.28916700 0.89244900

PPh_3 (N_{imag} = 0; Gibbs free energy = -1036.149135)

0 1

P 0.00083500 0.00068000 -1.23873400

C -1.04525600 1.29589600 -0.42799700

C -1.06320200 2.56886900 -1.02340600

C -1.81320100 1.08040800 0.72699600

C -1.81596200 3.60490900 -0.46922400

H -0.48365800 2.75094500 -1.92537400

C -2.57537400 2.11499200 1.27477400

H -1.81582200 0.10488800 1.20267200

C -2.57661300 3.37919300 0.68100200

H -1.81513100 4.58423300 -0.93949100

H -3.16590600 1.93240300 2.16838000

H -3.17001900 4.18222900 1.10884400

C -0.59870800 -1.55286200 -0.42894700

C -0.03145800 -2.10848400 0.72841200

C -1.69019100 -2.20582500 -1.02675500

C -0.54781200 -3.28506900 1.27634500

H 0.81334500 -1.62244200 1.20594500

C -2.21281200 -3.37489400 -0.47236200

H -2.13507400 -1.79612200 -1.93057400

C -1.64039300 -3.91911500 0.68037900

H -0.09703100 -3.70387700 2.17189100

H -3.05987400 -3.86440700 -0.94468100

H -2.04049600 -4.83380400 1.10848700

C 1.64546400 0.25770100 -0.42752300

C 2.75805700 -0.35462900 -1.02922200

C 1.84125400 1.01992000 0.73482400

C 4.03169000 -0.22238000 -0.47451000

H 2.62694500 -0.93952400 -1.93655600

C 3.11811000 1.16117100 1.28320100

H 0.99679200 1.50336600 1.21552200

C 4.21499300 0.53882700 0.68279900

H 4.88045900 -0.70582800 -0.95003900

H 3.25431400 1.75595800 2.18223300

H 5.20710700 0.65001500 1.11103300

Ia (N_{imag} = 0; Gibbs free energy = -1648.475471)

0 1

C -6.14967300 0.38182200 -1.05556100

C -4.94222000 -0.13169400 -1.52151000

C -4.09285100 -0.86686700 -0.65983800

C -4.51970000 -1.08859600 0.67212400

C -5.73766200 -0.58320100 1.11724700

C -6.55949400 0.16095600 0.26343000

H -6.77904100 0.95512500 -1.73125900

H -4.63332800 0.04144300 -2.54774200

H -3.87881700 -1.64789700 1.34673100

H -6.04367800 -0.76352000 2.14449900

H -7.50535100 0.55823400 0.61933100

C -2.83961400 -1.34418000 -1.11330300

C -1.60944500 -1.43108500 -1.27662100

C -0.32564200 -1.70258300 -1.59526800

C 0.87369500 -1.19437900 -1.24284100

P 1.11166600 0.14359700 0.01675800

C 2.45139400 1.35243500 -0.29764000

C 2.17903600 2.57290700 -0.93612000

C 3.77077000 1.05682700 0.08904200

C 3.21103100 3.47722700 -1.19083000

H 1.16858600 2.83337300 -1.22703600

C 4.79280400 1.97024300 -0.15902900

H 3.99575600 0.11633900 0.57454600

C 4.51783900 3.17982600 -0.80282100

H 2.98678700 4.41735200 -1.68570800

H 5.80680600 1.73376400 0.14917400

H 5.31842800 3.88746900 -0.99711100

C -0.44569500 1.08175700 0.10681000

C -1.07431600 1.32072200 1.33643900

C -1.02767000 1.56686900 -1.07769400

C -2.26999900 2.04025500 1.38003200

H -0.64636400 0.94022900 2.25694600

C -2.21694500 2.28963000 -1.02468700

H -0.56771100 1.35753400 -2.03787300

C -2.84093000 2.52501800 0.20343300

H -2.75795600 2.21091300 2.33460900

H -2.66666600 2.65145300 -1.94392000

H -3.77936300 3.06974700 0.23881100

C 1.41967800 -0.60063400 1.64859400

C 1.07723500 -1.94305800 1.86549200

C 1.95077100 0.16534700 2.69944200

C 1.26705400 -2.51353200 3.12406200

H 0.66792800 -2.53820100 1.05580100

C 2.13725100 -0.41299200 3.95448500

H 2.21897000 1.20456500 2.53978100

C 1.79682400 -1.75140400 4.16745600

H 1.00414500 -3.55433900 3.28618700

H 2.54972900 0.18164800 4.76385200

H 1.94617300 -2.19962500 5.14530300

C 2.16193500 -1.81224800 -1.61595400

O 3.20789300 -1.59969900 -1.00943100

O 2.09075900 -2.64441700 -2.66888700

C 3.32281200 -3.30397000 -3.01822400

H 3.68660600 -3.91457400 -2.18701100

H 3.08325900 -3.93588800 -3.87397300

H 4.09125600 -2.57468000 -3.28965300

2a (N_{imag} = 0; Gibbs free energy = -439.001623)

0 1

C -1.30719500 -1.43078900 -0.00006800

C -0.15077100 -0.65895000 -0.00002000

C -0.18957500 0.74741100 0.00007200

C -1.41256300 1.40148000 0.00010900

C -2.59302100 0.64113900 0.00005400

C -2.53530300 -0.75505500 -0.00003400

H -1.25914500 -2.51521100 -0.00013500

H -1.45653900 2.48711600 0.00017500

H -3.55631800 1.14208800 0.00007800

H -3.45582300 -1.33174800 -0.00007600

C 1.22783900 1.25712200 0.00012000

H 1.47913900 1.85718200 -0.88159200

H 1.47913800 1.85691500 0.88202000

C 2.07236700 -0.02455200 -0.00004900

N 1.18461600 -1.08076900 -0.00002300

O 3.28994600 -0.11894200 -0.00014000

H 1.48699800 -2.04626100 -0.00029100

Ic (N_{imag} = 0; Gibbs free energy = -1648.977237)

1 1

C 6.47190900 1.29700800 0.04935400

C 5.16984700 1.78645200 0.05323400

C 4.08120900 0.88776800 0.04973700

C 4.32286400 -0.50322400 0.04578200

C 5.62973600 -0.97869100 0.04166700

C 6.70396500 -0.08250700 0.04299300

H 7.30784600 1.98958700 0.05050400

H 4.97966300 2.85475200 0.05663500

H 3.48285200 -1.18862100 0.03903300

H 5.81362200 -2.04861500 0.03586800

H 7.72243800 -0.45904100 0.03868700

C 2.75104000 1.37592800 0.04255000

C 1.59211300 1.75684700 0.01878800

C 0.30026900 2.29350200 0.02006800

C -0.89798700 1.63837800 0.02712400

P -1.08559100 -0.17145300 -0.00240100

C -2.70032500 -0.64713300 -0.69264900

C -2.82875600 -0.87634600 -2.07164300

C -3.81664200 -0.78508900 0.14932300

C -4.06864500 -1.23287400 -2.60184800

H -1.97472200 -0.78810500 -2.73305100

C -5.04685400 -1.15254400 -0.38980900

H -3.72887900 -0.59865900 1.21212500

C -5.17561700 -1.37360600 -1.76360400

H -4.16333300 -1.40813200 -3.66881200

H -5.90570000 -1.26182500 0.26482300

H -6.13774600 -1.65796000 -2.17892300

C 0.20267700 -0.86427900 -1.07792500

C 0.81738900 -2.07797700 -0.73731000

C 0.53359100 -0.21952200 -2.28169800

C 1.75471400 -2.64300700 -1.60250000

H 0.57813500 -2.57900000 0.19402500

C 1.47470400 -0.79069400 -3.13542000

H 0.07101600 0.72614200 -2.54506500

C 2.08516400 -2.00118000 -2.79752500

H 2.23142400 -3.58097500 -1.33596000

H 1.73410000 -0.28607800 -4.06059200

H 2.82074800 -2.44101100 -3.46385500

C -0.92551700 -0.83995100 1.67262600

C -0.28158900 -0.08964700 2.66755900

C -1.39947900 -2.13313600 1.95504900

C -0.11296100 -0.63423300 3.93987200

H 0.08187700 0.91002100 2.45787800

C -1.22343800 -2.66662900 3.23034100

H -1.90114400 -2.71697300 1.19033000

C -0.58208400 -1.91897800 4.22136700

H 0.38145000 -0.05176000 4.71070900

H -1.59061400 -3.66423100 3.44918500

H -0.45104700 -2.33745900 5.21461200

C -2.14896200 2.41517300 0.24785900

O -3.11273400 1.96624300 0.84426700

O -2.08310400 3.65582700 -0.25083700

C -3.22631200 4.50200100 0.02126600

H -3.35359200 4.63467500 1.09846300

H -3.00119700 5.45395900 -0.45805700

H -4.13133300 4.06212300 -0.40384900

H 0.24855900 3.38155300 0.05609300

Id (N_{imag} = 0; Gibbs free energy = -438.493438)

-1 1

C -1.29068600 -1.41985100 0.00001200

C -0.12366800 -0.66626800 -0.00001100

C -0.13429900 0.77664400 -0.00001400

C -1.38980700 1.41678400 -0.00000700

C -2.56652400 0.65448200 0.00001400

C -2.52748000 -0.74686800 0.00002600

H -1.24652200 -2.50680400 0.00001800

H -1.44605800 2.50367000 -0.00000500

H -3.52858800 1.16284900 0.00002300

H -3.45115200 -1.31945200 0.00004500

C 1.21986200 1.21163600 -0.00000600

H 1.57406700 2.23482700 0.00001000

C 2.07708200 0.07463900 -0.00002400

N 1.19876900 -1.05516300 -0.00005800

O 3.32277900 -0.06131200 0.00002800

H 1.53775900 -2.00564900 0.00015300

Id′ (Oxindole N anion) (N_{imag} = 0; Gibbs free energy = -438.497783)

-1 1

C 1.27714000 -1.42919300 -0.00001000

C 0.08419500 -0.68429300 -0.00000400

C 0.16605200 0.74036800 0.00000800

C 1.38626900 1.39346200 -0.00000700

C 2.57692300 0.63913100 -0.00001800

C 2.50709300 -0.75764800 -0.00001300

H 1.23800200 -2.51591400 -0.00000900

H 1.43097800 2.48141100 -0.00001200

H 3.54116000 1.14038000 -0.00002800

H 3.42757300 -1.33839800 -0.00001900

C -1.25294700 1.22551800 0.00001400

H -1.52011200 1.82225100 -0.88109100

C -2.04892600 -0.11618000 0.00004100

N -1.20518700 -1.18310600 0.00001200

O -3.29199700 -0.14965300 -0.00001500

H -1.52009900 1.82224600 0.88112700

Ie (N_{imag} = 0; Gibbs free energy = -2087.506185)

0 1

C -5.77757700 -3.42718600 0.81838000

C -4.60115900 -2.75040500 1.13568500

C -4.18835600 -1.64490900 0.36638000

C -4.98202800 -1.23645600 -0.72564900

C -6.15708600 -1.91831900 -1.03385300

C -6.55912300 -3.01467400 -0.26467400

H -6.08546200 -4.27870500 1.41848700

H -3.99379100 -3.06908400 1.97724600

H -4.66257600 -0.38593700 -1.31994600

H -6.76037300 -1.59426300 -1.87709200

H -7.47546300 -3.54452100 -0.50817700

C -2.98765300 -0.93375100 0.67372100

C -1.98777100 -0.27480600 0.87413900

C -0.74985200 0.48364700 1.09003100

C 0.21452200 0.33767700 -0.08031700

P 1.70899700 -0.55240400 0.03521400

C 3.24149300 0.40065600 -0.35191700

C 3.91389300 1.11382600 0.65231600

C 3.70870500 0.48332200 -1.67464300

C 5.03246200 1.88764900 0.34012000

H 3.57543700 1.07732700 1.67890900

C 4.82849500 1.25580500 -1.98091900

H 3.19820300 -0.05267500 -2.46442000

C 5.49349400 1.96074500 -0.97530400

H 5.54296200 2.43061000 1.13007200

H 5.17895400 1.30654800 -3.00770200

H 6.36546900 2.56222600 -1.21560400

C 1.91597800 -1.26231100 1.71630300

C 2.06961800 -2.64897700 1.87682500

C 1.91595800 -0.43903000 2.85887100

C 2.23319700 -3.19876200 3.14951400

H 2.05965800 -3.30887900 1.01822300

C 2.08040200 -0.99762000 4.12628800

H 1.76443000 0.63175200 2.76407700

C 2.24269400 -2.37645000 4.27628500

H 2.35013200 -4.27318000 3.25508600

H 2.07675000 -0.34949100 4.99771800

H 2.36993800 -2.80696500 5.26527700

C 1.73702500 -1.99395700 -1.10368200

C 0.51752200 -2.48586700 -1.58742900

C 2.93139400 -2.64970300 -1.44027800

C 0.49329300 -3.61392000 -2.40788400

H -0.40785900 -1.98428200 -1.32166200

C 2.90228300 -3.77889600 -2.25963800

H 3.88175700 -2.28344000 -1.06561100

C 1.68433800 -4.26039400 -2.74644600

H -0.45581800 -3.98556800 -2.78251100

H 3.83046000 -4.28019300 -2.51775400

H 1.66430800 -5.13750900 -3.38682100

C -0.17920800 0.80673000 -1.37037800

O -1.25723900 1.30051100 -1.69742400

O 0.82597300 0.63395100 -2.31011800

C 0.48417400 0.95592200 -3.65871700

H -0.31795700 0.30928700 -4.03006100

H 0.16538600 1.99868700 -3.74886200

H 1.39037300 0.79261300 -4.24547000

H -0.27617400 0.04145900 1.96784900

C -1.05742700 1.96810300 1.53874300

H -1.38203500 1.91924500 2.58476400

C 0.23487700 2.80226200 1.47637700

O 1.29993400 2.57902700 2.04246400

N -0.00950300 3.86558200 0.64892800

H 0.69397700 4.56114200 0.43636200

C -1.34226400 3.90702400 0.21166900

C -2.02671100 2.79336200 0.72411300

C -1.96168300 4.86525100 -0.57847600

C -3.38268400 2.65091000 0.47156700

C -3.32654200 4.69220100 -0.85030000

H -1.41093500 5.71628200 -0.96692900

C -4.03134500 3.60502500 -0.32831600

H -3.93017900 1.80527000 0.87284000

H -3.84066400 5.42280700 -1.46838800

H -5.09078500 3.49618600 -0.54101800

Ie (N_{imag} = 0; Gibbs free energy = -2088.103108; B3LYP-D3 6-31G(d,p))

0 1

C 4.64057700 4.09393000 -0.21264900

C 4.10368500 2.82985600 -0.41015500

C 2.71460600 2.65098000 -0.48561400

C 1.88162300 3.77425800 -0.36179500

C 2.42652300 5.03465700 -0.16408700

C 3.80597900 5.20071200 -0.08736700

H 5.71500800 4.21657000 -0.15723600

H 4.75224200 1.96942400 -0.51074400

H 0.80991000 3.64992700 -0.43132900

H 1.76983400 5.89079300 -0.07175300

H 4.22842000 6.18560200 0.06602400

C 2.14749300 1.35723900 -0.66142600

C 1.61616400 0.28008600 -0.75969000

C 0.89036200 -0.98615000 -0.84546100

C -0.60660800 -0.76188400 -0.96313100

P -1.49462100 0.20589500 0.14115700

C -3.18902800 -0.41267700 0.40089200

C -3.39599700 -1.79504800 0.42587100

C -4.26752100 0.44932500 0.59496900

C -4.66837100 -2.30039000 0.65362500

H -2.56300700 -2.46498500 0.26048300

C -5.53985100 -0.06436800 0.82059100

H -4.13126300 1.52048300 0.55243100

C -5.74254200 -1.43848900 0.85260400

H -4.81983800 -3.37230300 0.66916400

H -6.37242600 0.61273300 0.96422700

H -6.73464500 -1.83672300 1.02516500

C -0.69033100 0.19817300 1.77895100

C 0.25937800 1.16109700 2.12859500

C -0.95705600 -0.85032000 2.66459100

C 0.93309700 1.07503400 3.34020300

H 0.49214300 1.96930400 1.45273700

C -0.28233000 -0.93143300 3.87504100

H -1.66549400 -1.62102600 2.40041200
C 0.66499000 0.02874000 4.21495300
H 1.67372800 1.82317200 3.59198800
H -0.49252400 -1.75227900 4.54851400
H 1.19382400 -0.04015900 5.15728300
C -1.64991900 1.97105800 -0.31723700
C -1.44118000 2.31567100 -1.65247000
C -1.96134800 2.97055300 0.60897000
C -1.54875400 3.64020200 -2.05815600
H -1.18326000 1.54249500 -2.36322900
C -2.06041800 4.29540500 0.20258700
H -2.11118700 2.71892500 1.65104000
C -1.85619700 4.63116400 -1.13237000
H -1.37910400 3.89894900 -3.09534700
H -2.29118800 5.06495000 0.92831500
H -1.92840600 5.66446200 -1.44764900
C -1.22404900 -1.31786300 -2.12477900
O -0.73514200 -2.14192600 -2.88342900
O -2.48459200 -0.80628400 -2.35830200
C -3.25654500 -1.47719100 -3.35315800
H -3.40456600 -2.52573700 -3.08851400
H -4.21386700 -0.96061600 -3.38224400
H -2.76744200 -1.42921700 -4.32649800
H 1.16325800 -1.46568700 -1.79016900
C 1.39635200 -1.97086300 0.27996500
C 0.58363900 -3.27382700 0.28350500
C 2.79786600 -2.45779900 0.01527000
H 1.28805600 -1.48826000 1.24925600
C 2.74818500 -3.82757800 -0.27145100
N 1.43088000 -4.27132600 -0.14038100
H 1.12408000 -5.22589600 -0.22769000
O -0.57230400 -3.43585700 0.61161200
C 3.87947800 -4.55326700 -0.60039700
H 3.82477200 -5.61132500 -0.82279800

C 4.01211000 -1.79909000 -0.01481100
H 4.06177400 -0.74165000 0.20567600
C 5.09757200 -3.87244000 -0.63286200
H 5.99953000 -4.41442200 -0.88762700
C 5.16788400 -2.51504100 -0.34016800
H 6.12398700 -2.00862500 -0.36530800

Ie′ (N_{imag} = 0; Gibbs free energy = -2087.982179)

1 1

C -5.96721100 -2.16869900 1.11877200

C -4.79989600 -1.51762800 1.51357200

C -3.90458700 -1.03010300 0.54366200

C -4.19958300 -1.20529700 -0.82433500

C -5.36749100 -1.86098100 -1.20700000

C -6.25311900 -2.34354500 -0.23832900

H -6.65489900 -2.54206700 1.87174000

H -4.57267900 -1.38286900 2.56637300

H -3.50494100 -0.82623300 -1.56670500

H -5.58855300 -1.99303700 -2.26210300

H -7.16329900 -2.85334900 -0.54000000

C -2.69021100 -0.37358800 0.90973700

C -1.62014500 0.16341800 1.09894400

C -0.34214400 0.82466200 1.32969700

C 0.67114100 0.68877200 0.14368300

P 1.39819500 -1.03365500 -0.07457200

C 3.15292500 -0.81042300 -0.47316700

C 4.06694800 -1.79330400 -0.05909900

C 3.59001900 0.28125300 -1.24101100

C 5.41153500 -1.67571100 -0.40664800

H 3.73666100 -2.63960100 0.53361100

C 4.93803400 0.38806500 -1.58049600

H 2.89663700 1.04410900 -1.57616200

C 5.84770700 -0.58662600 -1.16452500

H 6.11693000 -2.43331400 -0.08060600

H 5.27523000 1.23527200 -2.16920600

H 6.89666900 -0.49696000 -1.42994900

C 1.30123000 -1.92845000 1.49641700

C 0.68170900 -3.18207800 1.59399100

C 1.91495500 -1.35001800 2.62404100

C 0.66675200 -3.84742500 2.82055300

H 0.22093100 -3.64279900 0.72858500

C 1.88933100 -2.02287700 3.84241500

H 2.39811400 -0.37980600 2.55599000

C 1.26541200 -3.27025700 3.94114900

H 0.18721500 -4.81811200 2.89544800

H 2.35702900 -1.57331500 4.71235700

H 1.24943500 -3.79279100 4.89266400

C 0.59892100 -1.95132500 -1.41470300

C -0.75496200 -2.31772200 -1.32020700

C 1.33892600 -2.27722500 -2.56209800

C -1.35607300 -3.01273700 -2.36541300

H -1.33410000 -2.05710600 -0.44402700

C 0.72313700 -2.96927900 -3.60543000

H 2.38258200 -1.99649100 -2.64688000

C -0.61958700 -3.33721300 -3.50847000

H -2.40235300 -3.28976800 -2.28671700

H 1.29670600 -3.21997600 -4.49210600

H -1.09404900 -3.87550400 -4.32337800

C 0.10807900 1.21358100 -1.17929600

O -0.87538500 0.77731300 -1.73397200

O 0.85635300 2.22635400 -1.63964000

C 0.39574500 2.84056400 -2.86879900

H 0.43524600 2.11703800 -3.68667300

H -0.62417400 3.20635300 -2.74292600

H 1.08114800 3.66677200 -3.05178900

H 0.13488000 0.36049500 2.19781500

C -0.52049900 2.31181400 1.75514100

H -1.15863900 2.29724300 2.64748300

C 0.83857700 2.90617300 2.18213200

O 1.66704100 2.37442100 2.90876900

N 0.95763900 4.11991500 1.55712100

H 1.77367400 4.71008700 1.66034400

C -0.11927100 4.38439000 0.69665500

C -1.03464200 3.31925400 0.75459700

C -0.31953400 5.48870400 -0.12124100

C -2.18739200 3.35608400 -0.01744700

C -1.48428800 5.51020700 -0.90063000

H 0.39814900 6.30206000 -0.15415300

C -2.40699800 4.46140900 -0.85386500

H -2.89753600 2.53704500 0.01562700

H -1.66876700 6.35977700 -1.55144300

H -3.30180500 4.50080900 -1.46703700

H 1.54741400 1.28171100 0.41360900

TS$_{\mathbf{IeIe'}}$ (N_{imag} = 1, f_{imag} = -1045.01; Gibbs free energy = -2526.486157)

0 1

C 6.41081700 0.48046200 -2.38363500

C 5.04995100 0.77954200 -2.37650500

C 4.25352700 0.42953400 -1.26887600

C 4.85136700 -0.22408100 -0.17182100

C 6.21256100 -0.51964000 -0.19023000

C 6.99651000 -0.16969800 -1.29339700

H 7.01538000 0.75364700 -3.24359700

H 4.59194400 1.27966500 -3.22400800

H 4.24296400 -0.49517500 0.68316300

H 6.66203500 -1.02435300 0.66016800

H 8.05738100 -0.40199500 -1.30394800

C 2.85303900 0.70865200 -1.25995500

C 1.65232300 0.87745300 -1.22454200

C 0.19843600 1.07214500 -1.17875400

C -0.48101600 0.22081500 -0.03808000

P 0.38484900 -1.28380700 0.51455000

C -0.83852500 -2.46413700 1.19104900

C -1.60558900 -3.24807100 0.31608300

C -1.05138700 -2.55995100 2.57550100

C -2.58386300 -4.10359300 0.82292400

H -1.46041400 -3.18643600 -0.75449200

C -2.02471200 -3.42383600 3.07442000

H -0.46794700 -1.95758000 3.26072100

C -2.79597000 -4.19279800 2.19933500

H -3.18015100 -4.69711400 0.13706300

H -2.18255400 -3.49102000 4.14655200

H -3.55922000 -4.85940600 2.58974600

C 1.15365300 -2.10392800 -0.92612700

C 2.40071100 -2.73719300 -0.81095000

C 0.48030600 -2.11506200 -2.15910800

C 2.96288000 -3.37794900 -1.91450900

H 2.95313100 -2.71684600 0.11943800

C 1.05344700 -2.75892700 -3.25557500

H -0.46832600 -1.60829200 -2.30399300

C 2.29166200 -3.39226700 -3.13840300

H 3.93352300 -3.85423500 -1.81576500

H 0.52643200 -2.74979500 -4.20479500

H 2.73597900 -3.88655700 -3.99728200

C 1.64395000 -1.00632500 1.80006300

C 2.01486400 0.30108800 2.13964500

C 2.24682900 -2.09395800 2.45311200

C 3.00000200 0.52026100 3.10096700

H 1.53945900 1.14643800 1.66113800

C 3.23804100 -1.86842200 3.40864200

H 1.94211500 -3.11029000 2.22385800

C 3.61919800 -0.56291300 3.72902300

H 3.28126400 1.53760200 3.35497800

H 3.70610800 -2.71260400 3.90532800

H 4.39046200 -0.39111500 4.47387600

C -1.19445900 0.94374700 1.04487200

O -1.90670600 1.91674100 0.88162900

O -1.06933100 0.34426200 2.25321300

C -1.89625000 0.86836400 3.31024000

H -1.62092500 1.90308700 3.52842700

H -2.95089000 0.82238600 3.02884600

H -1.70351000 0.23336300 4.17483900

H -0.21956400 0.69601000 -2.12253400

C -0.06692800 2.60847500 -1.23574000

H 0.47033900 2.92528300 -2.14246900

C -1.52083700 3.04181200 -1.51270300

O -2.31839900 2.52749300 -2.27617700

N -1.71538700 4.21652100 -0.81241400

H -2.62798200 4.64928000 -0.75519400

C -0.67142100 4.47380600 0.07788200

C 0.35308400 3.52600200 -0.10998900

C -0.57506600 5.48480900 1.02672900

C 1.51293200 3.61266200 0.64697100

C 0.58963600 5.54004300 1.80525000

H -1.37781900 6.20313600 1.16153200

C 1.62352500 4.61997100 1.62020200

H 2.32501000 2.91201100 0.48961800

H 0.68683400 6.31708600 2.55821900

H 2.52346600 4.68569500 2.22438500

H -1.50449500 -0.29389800 -0.64781300

C -3.91171400 -0.54499700 -0.71663900

C -2.83294300 -0.42088600 -2.68007500

C -4.94264800 -0.18745900 -1.61876400

C -4.30527400 -0.06409900 -2.97580900

H -4.72499500 -0.73500800 -3.73390100

H -4.33708000 0.95450400 -3.37805900

N -2.67778200 -0.67845100 -1.34914000

O -1.93399900 -0.46904900 -3.53124300

C -6.24295000 -0.01711100 -1.17036300

H -7.03521500 0.25975600 -1.86231600

C -6.52985600 -0.20473500 0.19418300

H -7.54528700 -0.07338400 0.55726200

C -4.19214800 -0.74285400 0.63948000

H -3.40650000 -1.03268400 1.33000400

C -5.51016900 -0.56361800 1.08085900

H -5.74114300 -0.71293700 2.13295600

TS$_{IeIf}$ (N$_{imag}$ = 1, *f*$_{imag}$ = -1734.02; Gibbs free energy = -2088.028431; B3LYP-D3/def2tzvp)
0 1
C -5.69351400 -1.41805900 -0.30076100
C -4.59818900 -0.66188400 -0.69248300
C -3.38475800 -1.28774600 -1.01532300
C -3.30005300 -2.68612700 -0.93560000
C -4.40003500 -3.43369900 -0.54018800
C -5.59987700 -2.80468800 -0.22140800
H -6.62464600 -0.92292800 -0.05519300
H -4.66443100 0.41670200 -0.74809600
H -2.36599400 -3.17349300 -1.18031800
H -4.32066500 -4.51216000 -0.48197000
H -6.45698700 -3.39083700 0.08480600
C -2.24284400 -0.51349000 -1.35850600
C -1.25431100 0.15040600 -1.53829600
C -0.04606900 0.93071500 -1.70218000

C 1.26470000 0.38316700 -1.00599600
P 1.12622800 -0.74048300 0.33165800
C 2.73245500 -0.85426800 1.17495600
C 3.45651700 0.31617100 1.42829300
C 3.24455300 -2.07854000 1.60723400
C 4.65518900 0.26019100 2.12181000
H 3.10729000 1.26340100 1.04468600
C 4.45048400 -2.12962600 2.29737300
H 2.72054000 -2.99931000 1.39743300
C 5.15361400 -0.96200900 2.56237000
H 5.20773300 1.17241800 2.30612800
H 4.83920700 -3.08614900 2.62221100
H 6.09229600 -1.00326600 3.10016500
C -0.07568700 -0.14027300 1.56273000
C -1.31168300 -0.76092200 1.75450800
C 0.24413000 0.98840800 2.32379700
C -2.20099500 -0.27316900 2.70278400
H -1.60275500 -1.60726200 1.15318900
C -0.64984000 1.47595800 3.26441000
H 1.18190300 1.50329400 2.17842000
C -1.87099900 0.84252300 3.46124200
H -3.16006300 -0.75874100 2.82795700
H -0.39992100 2.36486000 3.82768700
H -2.57119600 1.23199400 4.18885000
C 0.62290800 -2.42101300 -0.14855400
C 0.61922600 -2.75192400 -1.50554100
C 0.26990300 -3.38899200 0.79894800
C 0.26982500 -4.03691600 -1.90486100
H 0.87733200 -2.01285000 -2.25192300
C -0.07985400 -4.66901600 0.39296100
H 0.25743300 -3.14235600 1.85253200
C -0.07871200 -4.99465600 -0.96026800
H 0.26343800 -4.28257300 -2.95871000
H -0.35713500 -5.40979000 1.13183000

H -0.35513700 -5.99253700 -1.27643600

C 2.37381100 0.16004200 -1.97369600

O 2.21472800 -0.17424800 -3.12743900

O 3.59233100 0.39967900 -1.44703200

C 4.69756400 0.25259000 -2.34946100

H 4.61200500 0.96970900 -3.16522400

H 5.58607200 0.45046000 -1.75486700

H 4.72925700 -0.75625100 -2.76200700

H 0.19053300 0.99042200 -2.76824400

C -0.04679900 2.34153300 -1.10405800

C 0.75112300 3.35621200 -1.79736200

C -1.05077700 3.05157100 -0.33038100

H 1.10635100 1.67653200 -0.46126800

C -0.73287100 4.43287400 -0.40827200

N 0.36317700 4.58116400 -1.24464500

H 0.74994100 5.45411300 -1.55934000

O 1.62779400 3.20885000 -2.64201000

C -1.45556400 5.39397200 0.27586600

H -1.19241400 6.44272200 0.20721800

C -2.14546400 2.66292600 0.43399500

H -2.41361800 1.61972100 0.50964400

C -2.54600000 4.97663700 1.04373600

H -3.13163900 5.71217000 1.58075200

C -2.88776800 3.62858800 1.11447600

H -3.73923900 3.32202600 1.70983800

If (N_{imag} = 0; Gibbs free energy = -2087.500679)

0 1

C -3.56035900 -4.91047600 1.33907000

C -2.98065500 -3.66235100 1.55777900

C -2.65551600 -2.83130800 0.46838400

C -2.92703000 -3.27823500 -0.84038300

C -3.50605600 -4.52821700 -1.04891900

C -3.82354000 -5.34864300 0.03780400

H -3.80591800 -5.54390500 2.18665600

H -2.76808100 -3.32337400 2.56673500

H -2.66910900 -2.64103500 -1.68033100

H -3.70899000 -4.86350800 -2.06200600

H -4.27410400 -6.32285500 -0.12813400

C -2.02037700 -1.56963700 0.67195200

C -1.43006400 -0.51821300 0.79729900

C -0.73972000 0.76020700 0.96612900

C 0.37764800 1.01617900 -0.15029000

P 1.88329600 -0.04960800 -0.01305200

C 2.82821800 0.08980000 -1.55631500

C 3.54841100 -1.00018500 -2.06633100

C 2.88083400 1.33535100 -2.20470700

C 4.30623300 -0.84448100 -3.22736600

H 3.51499800 -1.96392000 -1.57084800

C 3.63768000 1.47826600 -3.36580100

H 2.33347700 2.18827800 -1.81616500

C 4.34913900 0.38997100 -3.87799200

H 4.85925100 -1.69067800 -3.62294600

H 3.67019100 2.43928200 -3.86922700

H 4.93629300 0.50506900 -4.78403600

C 2.91325300 0.58972100 1.33793700

C 4.19637300 0.02818100 1.47450900

C 2.48139600 1.59365100 2.21894200

C 5.03955100 0.46485200 2.49218200

H 4.53605200 -0.74266100 0.78871900

C 3.34173000 2.02193800 3.23339300

H 1.50004000 2.06058200 2.15033900

C 4.61182600 1.46278600 3.37361900

H 6.02903200 0.03031500 2.59453900

H 3.00671200 2.79867900 3.91391500

H 5.27129800 1.80398900 4.16611400

C 1.48213100 -1.78399200 0.31292400

C 1.62294600 -2.29972400 1.61012900

C 0.96602000 -2.58981000 -0.71693000

C 1.24847800 -3.61753600 1.87276800

H 2.01324600 -1.68176900 2.41115700

C 0.60452100 -3.90603400 -0.44337200

H 0.82437900 -2.18259500 -1.71049900

C 0.74161200 -4.41959400 0.84930100

H 1.35195200 -4.01292500 2.87830500

H 0.19668000 -4.52499900 -1.23572300

H 0.44395100 -5.44248300 1.05872600

C -0.21762100 0.93243700 -1.54340000

O -0.44158200 -0.10370100 -2.14172600

O -0.48616800 2.15237800 -2.01655900

C -1.17399800 2.19901600 -3.28756100

H -0.58919200 1.68705900 -4.05559800

H -2.15854700 1.73569200 -3.19350900

H -1.27588100 3.25787900 -3.52222300

H -0.19242000 0.72240700 1.91670800

C -1.65623200 1.93757300 1.04013500

C -1.28460700 3.07490700 1.80799500

O -0.27955300 3.27799100 2.53975900

N -2.29476300 4.03827400 1.59050800

H -2.27518000 4.96020400 2.00066700

C -3.23274200 3.57366900 0.68450300

C -2.84277500 2.24259800 0.30709000

C -4.36022100 4.19389500 0.16113300

C -3.63584000 1.56677900 -0.63898000

C -5.13517600 3.49143100 -0.77752500

H -4.62943400 5.20213700 0.46699600

C -4.76890500 2.19625100 -1.17025800

H -3.36692200 0.56357900 -0.95546600

H -6.01969300 3.95964200 -1.20079200

H -5.37671600 1.66615300 -1.90012800

H 0.73557300 2.03249300 0.02540600

TS$_{\mathbf{If4a}}$ (N$_{imag}$ = 1, f_{imag} = -415.80; Gibbs free energy = -2087.493085)

0 1

C -2.75431800 5.21172500 -1.54500300

C -2.47780800 3.86621900 -1.78078400

C -2.10302200 3.02636800 -0.71567200

C -2.01129100 3.55928600 0.58690400

C -2.29043700 4.90564000 0.81050900

C -2.66146700 5.73567300 -0.25226900

H -3.04112300 5.85365700 -2.37293000

H -2.54206300 3.45790300 -2.78434900

H -1.70501800 2.90707300 1.39877800

H -2.21500500 5.30966400 1.81604400

H -2.87643100 6.78527700 -0.07403700

C -1.77459900 1.65445700 -0.92471100

C -1.44536800 0.48979300 -1.01498900

C -1.03766700 -0.88696700 -1.01404700

C 0.29079200 -0.99378700 0.46731600

P 1.89825000 -0.28698400 0.08335000

C 2.85497200 0.07460300 1.59330700

C 3.78475800 1.12381800 1.63617400

C 2.69208800 -0.76216000 2.70825000

C 4.54115000 1.33384500 2.79016100

H 3.91703900 1.77788400 0.78131600

C 3.45085500 -0.54500700 3.85677900

H 1.97128300 -1.57408800 2.68595000

C 4.37457400 0.50274900 3.89942400

H 5.25649500 2.15003400 2.82072200

H 3.31717600 -1.19135300 4.71870400

H 4.96136500 0.67217300 4.79730700

C 2.84752200 -1.49593300 -0.88744400

C 4.23391800 -1.29583700 -1.01155200

C 2.23318500 -2.57922400 -1.53435300

C 4.99779900 -2.17199600 -1.77828400

H 4.71789500 -0.46454600 -0.50888500

C 3.01266900 -3.45215400 -2.29760100

H 1.16576900 -2.77254800 -1.46200000

C 4.38753100 -3.25216100 -2.42297900

H 6.06803700 -2.01349700 -1.86869700

H 2.53311300 -4.29092500 -2.79303600

H 4.98513000 -3.93597000 -3.01865700

C 1.77518000 1.24466300 -0.88673100

C 1.99351400 1.21354800 -2.27189600

C 1.41639200 2.44716100 -0.25608600

C 1.85034800 2.38218200 -3.02088900

H 2.27123700 0.28856400 -2.76575000

C 1.28732300 3.60994700 -1.01184300

H 1.21305400 2.46054400 0.80772400

C 1.50120400 3.57878800 -2.39250100

H 2.01551300 2.35501900 -4.09353200

H 0.99822400 4.53550400 -0.52444800

H 1.38964600 4.48662700 -2.97784500

C -0.38027200 -0.35062300 1.60494800

O -0.24992600 0.82359200 1.94008300

O -1.23129700 -1.20101000 2.21198400

C -2.07773900 -0.62665500 3.22455400

H -1.47827600 -0.21726800 4.04244600

H -2.70378800 0.16212600 2.80057000

H -2.70283000 -1.44491700 3.58175400

H -0.33143000 -1.13456800 -1.80672300

C -2.03139400 -1.89446300 -0.81613900

C -1.74881100 -3.26915400 -1.15382000

O -0.72282700 -3.78004000 -1.65174400

N -2.88436300 -4.00306300 -0.78815600

H -2.95259000 -5.00432200 -0.89841000

C -3.83802500 -3.18615700 -0.19517300

C -3.32759600 -1.85141200 -0.18240400

C -5.08285300 -3.51406200 0.32772100

C -4.12201900 -0.84517600 0.38660300

C -5.85456700 -2.48735300 0.89297400

H -5.44245300 -4.53934600 0.30307600

C -5.37503400 -1.17144200 0.92060600

H -3.76554500 0.17845900 0.41707000

H -6.83006000 -2.71960000 1.31122600

H -5.98418600 -0.38745800 1.36339400

H 0.35009400 -2.07668600 0.53309900

4a (N_{imag} = 0; Gibbs free energy = -784.253784)

0 1

C 4.63375700 1.54084100 -0.00027800

C 3.33863900 1.03123800 -0.00002400

C 3.12687500 -0.36434000 0.00015000

C 4.24062400 -1.23122300 0.00007700

C 5.53104500 -0.70955500 -0.00017700

C 5.73161700 0.67445700 -0.00035700

H 4.78879700 2.61559500 -0.00041400

H 2.48356800 1.69990900 0.00004100

H 4.07897700 -2.30437900 0.00021900

H 6.38297700 -1.38287900 -0.00023600

H 6.74036400 1.07659700 -0.00055500

C 1.80622100 -0.88918700 0.00042600

C 0.66650800 -1.32574300 0.00059600

C -0.63649400 -1.84792400 0.00027300

H -0.74630200 -2.93235500 0.00020200

C -1.78104900 -1.11646200 0.00008700

C -3.12279800 -1.79784500 -0.00020300

C -2.04148100 0.31611700 0.00012500

C -3.44876300 0.47932600 -0.00017600

N -4.05699100 -0.77878800 -0.00042700

H -5.05428000 -0.94482500 -0.00051700

O -3.36376800 -2.99873600 -0.00033700

C -4.04258400 1.73385800 -0.00023000

H -5.12231100 1.84360900 -0.00046700

C -1.21676100 1.44215200 0.00037800

H -0.13842100 1.32909500 0.00060700

C -3.19680300 2.85208300 0.00003900

H -3.63611400 3.84538100 0.00001000

C -1.80424700 2.71214100 0.00033800

H -1.17401000 3.59605100 0.00053900

TS$_{\mathbf{If4a'}}$ (N_{imag} = 1, f_{imag} = -409.83; Gibbs free energy = -2087.484494)

0 1

C -4.81686700 -3.62426300 -1.71061500

C -3.48738500 -3.21761100 -1.80944500

C -2.80598900 -2.74653800 -0.67151400

C -3.48206200 -2.69381700 0.56497300
C -4.81068000 -3.10288800 0.65230000
C -5.48299000 -3.56741400 -0.48286100
H -5.33522400 -3.98462600 -2.59465200
H -2.97020800 -3.25220000 -2.76308400
H -2.95346800 -2.31235800 1.43285100
H -5.32515800 -3.05577400 1.60795400
H -6.51959700 -3.88370300 -0.41089000
C -1.46255800 -2.27625600 -0.74582800
C -0.33455400 -1.83083000 -0.71680300
C 0.96444400 -1.22005500 -0.63754800
C 0.71044800 0.24441700 0.67926300
P -0.19674900 1.65822500 0.04935600
C -0.67492600 2.81626400 1.37621000
C -1.69565100 3.75597400 1.16231800
C 0.03611900 2.81708500 2.58461700
C -2.00435600 4.68231300 2.15751800
H -2.25396100 3.76021600 0.23198000
C -0.27973800 3.74687900 3.57517100
H 0.82877900 2.09637600 2.75811300
C -1.29880500 4.67784600 3.36355500
H -2.79837200 5.40393000 1.99156600
H 0.26914900 3.73984500 4.51172100
H -1.54447400 5.39821600 4.13804300
C 0.88137500 2.57741300 -1.09416500
C 0.31411900 3.59530400 -1.88104800
C 2.26197900 2.34352900 -1.15490600
C 1.11925900 4.35229200 -2.72896000
H -0.75247900 3.79142000 -1.83913900
C 3.06164600 3.10423100 -2.00954300
H 2.72862400 1.57734900 -0.54960000
C 2.49345900 4.10553500 -2.79756000
H 0.67261200 5.13299400 -3.33676800
H 4.12850600 2.90830900 -2.05396000

H 3.11827100 4.69506000 -3.46169900
C -1.70570800 1.19411800 -0.84949500
C -1.67153300 1.01125400 -2.24036900
C -2.89385700 0.95887200 -0.13969800
C -2.82274900 0.60566800 -2.91471800
H -0.75646900 1.17981700 -2.79723500
C -4.04080300 0.56266900 -0.82426600
H -2.91464300 1.06287300 0.93796600
C -4.00704500 0.38457900 -2.20907900
H -2.79089000 0.46058200 -3.99003000
H -4.95537400 0.37266500 -0.27197500
H -4.90050900 0.06298800 -2.73546400
C 0.07465700 -0.39625700 1.84078500
O -1.13267400 -0.44678300 2.05407100
O 0.99836200 -0.98157000 2.62848000
C 0.49062300 -1.87294200 3.64006300
H -0.27655200 -1.38152200 4.24338800
H 0.09167900 -2.76809700 3.15763300
H 1.35095200 -2.13427100 4.25728800
H 1.17802000 -0.57166700 -1.48955500
C 2.07796000 -2.02073700 -0.23250500
C 2.02035900 -3.15631300 0.66981800
O 1.06322900 -3.76456000 1.17665800
N 3.36396400 -3.51957500 0.90219400
H 3.62015900 -4.31185100 1.47310200
C 4.24302200 -2.69430200 0.22224200
C 3.46521000 -1.73508500 -0.49598800
C 5.63156700 -2.70747000 0.17286000
C 4.13462500 -0.78736100 -1.28219700
C 6.27707300 -1.73831800 -0.61306000
H 6.20041800 -3.44832100 0.72840200
C 5.53547300 -0.79324600 -1.33256600
H 3.57266300 -0.06295600 -1.86431900
H 7.36234900 -1.72915900 -0.66371300

H 6.05114700 -0.05668700 -1.94357000
H 1.75523900 0.49026300 0.84456000

4a′ (N_{imag} = 0; Gibbs free energy = -784.251409)

0 1

C 5.29936100 1.26870000 0.00007700

C 3.93802600 0.97981000 0.00000600

C 3.49947800 -0.36233200 -0.00003400

C 4.45449100 -1.40095500 -0.00000400

C 5.81351400 -1.09926000 0.00006800

C 6.23937700 0.23279100 0.00010900

H 5.62954500 2.30318000 0.00010800

H 3.20061700 1.77613600 -0.00001900

H 4.11850700 -2.43297400 -0.00003600

H 6.54288100 -1.90384600 0.00009100

H 7.30072500 0.46297300 0.00016400

C 2.10878200 -0.65152900 -0.00011200

C 0.90136900 -0.82671200 -0.00015400

C -0.47059900 -1.12476800 -0.00008200

H -0.73107100 -2.18313200 -0.00006100

C -1.48668800 -0.22112700 -0.00007900

C -1.32018300 1.27500700 -0.00018400

C -2.92198500 -0.47667300 -0.00003400

C -3.57168800 0.77990300 0.00000800

N -2.60667800 1.78871100 -0.00007700

H -2.79985600 2.78107700 0.00018400

O -0.29035600 1.93689400 -0.00005300

C -4.95613000 0.89081800 0.00011000

H -5.44122600 1.86166100 0.00014700

C -3.68099400 -1.64690600 0.00002500

H -3.19404300 -2.61761900 0.00001500

C -5.70141900 -0.29617800 0.00015900

H -6.78595900 -0.23798600 0.00023700

C -5.07653500 -1.54950300 0.00011600

H -5.67959200 -2.45212600 0.00016500

Ylide 4′ (N_{imag} = 0; Gibbs free energy = -1303.269126)

0 1

C -1.02178200 0.01500800 -1.64810600

P 0.05145800 -0.00495900 -0.30305900

C -0.42023900 -1.19624100 1.00242100

C -1.20344500 -2.30344200 0.64779200

C 0.02168000 -1.05022600 2.32551000

C -1.53652200 -3.25885000 1.60752700

H -1.55194200 -2.40580500 -0.37516600

C -0.31084000 -2.01127000 3.28106100

H 0.61991800 -0.19141300 2.61241400

C -1.08947400 -3.11474700 2.92353200

H -2.14767700 -4.11229100 1.32916100

H 0.03389800 -1.89475600 4.30425700

H -1.35085400 -3.85889100 3.67030800

C 1.68729400 -0.49377000 -0.94148100

C 2.24445400 0.24489900 -1.99905800

C 2.39504100 -1.57447100 -0.39930600

C 3.49268700 -0.10469000 -2.51128500

H 1.70516500 1.08838700 -2.41922700

C 3.64645200 -1.91747500 -0.91605100

H 1.97465200 -2.14856800 0.41939300

C 4.19508800 -1.18567100 -1.97028200

H 3.91775500 0.46787000 -3.33018000

H 4.18942900 -2.75750300 -0.49340900

H 5.16835400 -1.45494300 -2.37005200

C 0.30703100 1.59377800 0.56072600

C -0.78498100 2.45945600 0.73731100

C 1.57246400 1.96219300 1.04285800

C -0.60507200 3.67858500 1.39053600

H -1.76491500 2.16750000 0.37390300

C 1.74321100 3.18400300 1.69678600

H 2.42405900 1.30343300 0.90605800

C 0.65620200 4.04301600 1.87017500

H -1.45192200 4.34539100 1.52452100

H 2.72567900 3.46427500 2.06530300

H 0.79141800 4.99453100 2.37636800

C -2.41430600 0.18970800 -1.46035700

O -3.00524400 0.40871300 -0.38970700

O -3.11881100 0.08204800 -2.63920000

C -4.53475200 0.24906800 -2.51917000

H -4.79073800 1.23579700 -2.11904000

H -4.93464100 0.14581800 -3.53023400

H -4.97553000 -0.51312700 -1.86797600

H -0.60268900 -0.18055400 -2.62609100

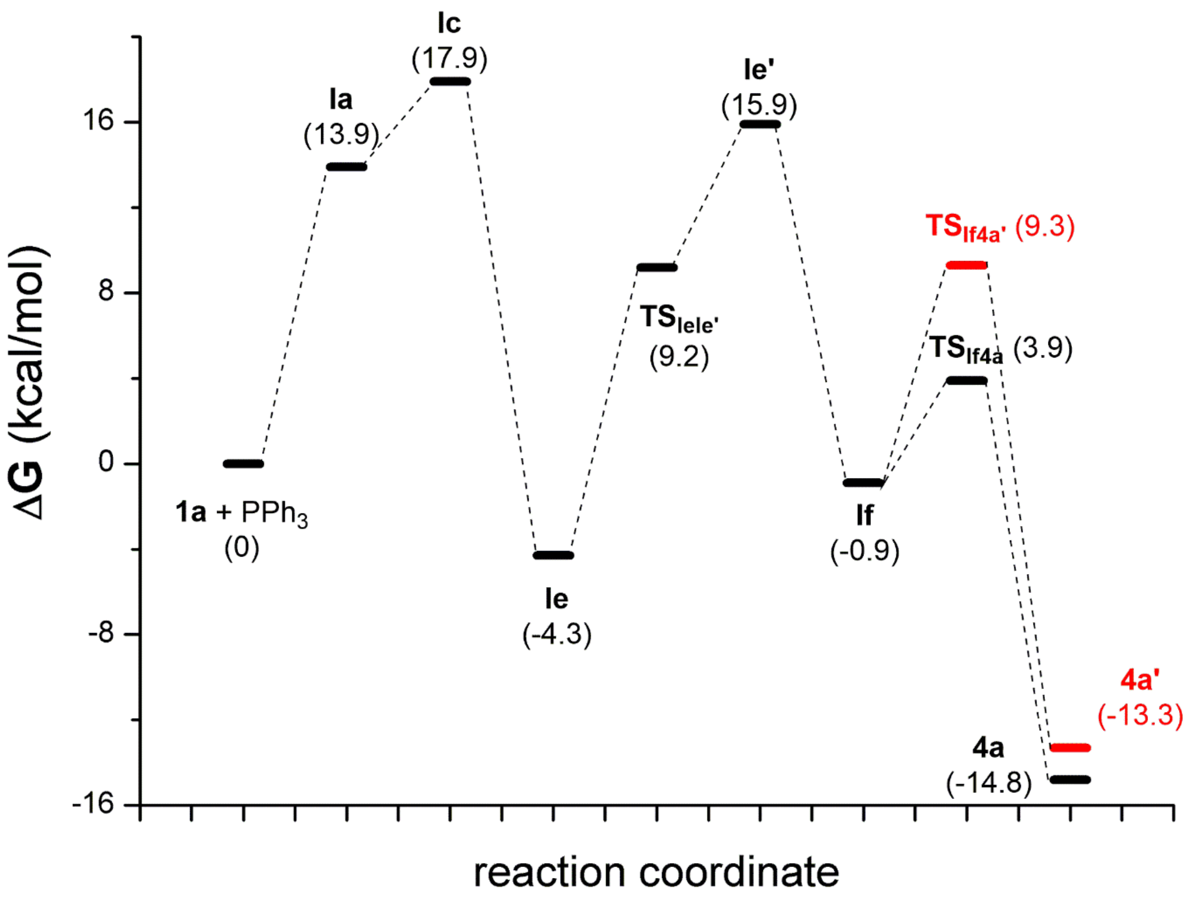

Figure S150. Energy profile of the reaction of 1a, 2a, and PPh_3 (B3LYP-D3/6-31g(d,p))

References

(1) Deng, J.-C.; Kuo, C.-W.; Chuang, S.-C. Nucleophilic conjugate 1, 3-addition of phosphines to oligoynoates. *Chem. Commun.* **2014**, *50*, 10580-10583.

(2) Nallapati, S.; Tseng, M.-F.; Chen, P.-L.; Chuang, S.-C. Phosphine-Mediated [4+ 3] Annulation of Diynoates and 2-Arylidene Indane-1, 3-diones: Access of Indeno [1, 2-b] oxepin-4-ylidenes and Beyond. *Org. Lett.* **2022**, *24*, 2993-2997.

(3) Marti, C.; Carreira, E. M. Total synthesis of (−)-spirotryprostatin B: synthesis and related studies. *J. Am. Chem. Soc.* **2005**, *127*, 11505-11515.

(4) Xia, A.-B.; Huang, L.-S.; Li, C.-P.; Hu, Q.-B.; Zhu, J.-Y.; Bai, L.; Xu, D.-Q. Enantioselective construction of spirodihydrofuran oxindoles via one-pot organo-/iodine sequential catalysis. *Org. Chem. Front.* **2023**, *10*, 4848-4853.

(5) Zhang, L.; Ren, W.; Wang, X.; Zhang, J.; Liu, J.; Zhao, L.; Zhang, X. Discovery of novel polycyclic spiro-fused carbocyclicoxindole-based anticancer agents. *Chem. - Eur. J.* **2017**, *126*, 1071-1082.

(6) Aghazadeh, M. Formation of indole trimers in Vilsmeier type reactions. *Arkivoc.* **2019**, 141-148.

(7) Chen, D.-Z.; Xiao, W.-J.; Chen, J.-R. Synthesis of spiropyrazoline oxindoles by a formal [4+ 1] annulation reaction between 3-bromooxindoles and in situ-derived 1, 2-diaza-1, 3-dienes. *Org. Chem. Front.* **2017**, *4*, 1289-1293.

(8) Kumar, N.; Kumar, A.; Sahoo, S. C.; Chimni, S. S. Candida antarctica lipase-B-catalyzed kinetic resolution of 1, 3-dialkyl-3-hydroxymethyl oxindoles. *Chirality.* **2020**, *32*, 1377-1394.

(9) Zhao, J.-b.; Ren, X.; Zheng, B.-q.; Ji, J.; Qiu, Z.-b.; Li, Y. Cinchona-alkaloid-catalyzed enantioselective hydroxymethylation of 3-fluorooxindoles with paraformaldehyde. *J. Fluor. Chem.* **2018**, *215*, 44-51.

(10) Basak, S.; Alvarez-Montoya, A.; Winfrey, L.; Melen, R. L.; Morrill, L. C.; Pulis, A. P. B (C6F5) 3-catalyzed direct C3 alkylation of indoles and oxindoles. *ACS Catal.* **2020**, *10*, 4835-4840.

(11) Babu, B.; Nagarsenkar, A.; Naidu, V.; Lalita, G.; Guggilapu, S.; Prajapti, S. 3-((7-hydroxy-4-methyl-2-oxo-2 H-chromen-8-yl) methylene) indolin-2-one derivatives as anticancer agents. *Indian Patent Application No# 201741003441* **2017**.

(12) Zhao, P.; Li, Y.; Gao, G.; Wang, S.; Yan, Y.; Zhan, X.; Liu, Z.; Mao, Z.; Chen, S.; Wang, L. Design, synthesis and biological evaluation of N-alkyl or aryl substituted isoindigo derivatives as potential dual cyclin-dependent kinase 2 (CDK2)/glycogen synthase kinase 3β (GSK-3β) phosphorylation inhibitors. *Chem. - Eur. J.* **2014**, *86*, 165-174.

(13) Ma, Y.; Fan, C.; Jia, B.; Cheng, P.; Liu, J.; Ma, Y.; Qiao, K. Total synthesis and biological evaluation of spirotryprostatin A analogs. *Chirality.* **2017**, *29*, 737-746.

(14) Deng, H.; Konopelski, J. P. Aryllead (IV) reagents in synthesis: formation of the C11 quaternary center of N-methylwelwitindolinone C isothiocyanate. *Org. Lett.* **2001**, *3*, 3001-3004.

(15) Ölgen, S.; Götz, C.; Jose, J. Synthesis and biological evaluation of 3-(substituted-benzylidene)-1, 3-dihydro-indolin derivatives as human protein kinase CK2 and p60c-Src tyrosine kinase inhibitors. *Biol. Pharm. Bull.* **2007**, *30*, 715-718.

(16) Thanigaimalai, P.; Lee, K.-C.; Sharma, V. K.; Sharma, N.; Roh, E.; Kim, Y.; Jung, S.-H. Identification of indoline-2-thione analogs as novel potent inhibitors of α-melanocyte stimulating hormone induced melanogenesis. *Chem. Pharm. Bull.* **2011**, *59*, 1285-1288.

(17) Lozinskaya, N. A.; Babkov, D. A.; Zaryanova, E. V.; Bezsonova, E. N.; Efremov, A. M.; Tsymlyakov, M. D.; Anikina, L. V.; Zakharyascheva, O. Y.; Borisov, A. V.; Perfilova, V. N. Synthesis and biological evaluation of 3-substituted 2-oxindole derivatives as new glycogen synthase kinase 3β inhibitors. *Bioorg. Med. Chem.* **2019**, *27*, 1804-1817.

(18) Mohammadinezhada, A.; Akhlaghinia, B. Fe3O4@Boehmite-NH_2-Co^{II} NPs: an inexpensive and highly efficient heterogeneous magnetic nanocatalyst for the Suzuki–Miyaura and Heck–Mizoroki cross-coupling reactions. *Green Chem*. 2017, *19,* 5625-5641.
